# Second-Line Pharmaceutical Treatments for Patients with Type 2 Diabetes

**DOI:** 10.1001/jamanetworkopen.2023.36613

**Published:** 2023-10-02

**Authors:** Rohit Vashisht, Ayan Patel, Lisa Dahm, Cora Han, Kathryn E. Medders, Robert Mowers, Carrie L. Byington, Suneil K. Koliwad, Atul J. Butte

**Affiliations:** 1 Bakar Computational Health Sciences Institute, University of California, San Francisco; 2 Center for Data-driven Insights and Innovation, University of California Health, Oakland; 3 Department of Pharmacy, UC San Diego Health, San Diego, California; 4 Managed Care Pharmacy Services, University of California, Davis School of Medicine, Davis; 5 Department of Pediatrics, University of California, San Francisco; 6 Division of Endocrinology and Metabolism, Department of Medicine, and Diabetes Center, University of California, San Francisco

## Abstract

**Importance:**

Assessing the relative effectiveness and safety of additional treatments when metformin monotherapy is insufficient remains a limiting factor in improving treatment choices in type 2 diabetes.

**Objective:**

To determine whether data from electronic health records across the University of California Health system could be used to assess the comparative effectiveness and safety associated with 4 treatments in diabetes when added to metformin monotherapy.

**Design, Setting, and Participants:**

This multicenter, new user, multidimensional propensity score–matched retrospective cohort study with leave-one-medical-center-out (LOMCO) sensitivity analysis used principles of emulating target trial. Participants included patients with diabetes receiving metformin who were then additionally prescribed either a sulfonylurea, dipeptidyl peptidase-4 inhibitor (DPP4I), sodium-glucose cotransporter-2 inhibitor (SGLT2I), or glucagon-like peptide-1 receptor agonist (GLP1RA) for the first time and followed-up over a 5-year monitoring period. Data were analyzed between January 2022 and April 2023.

**Exposure:**

Treatment with sulfonylurea, DPP4I, SGLT2I, or GLP1RA added to metformin monotherapy.

**Main Outcomes and Measures:**

The main effectiveness outcome was the ability of patients to maintain glycemic control, represented as time to metabolic failure (hemoglobin A_1c_ [HbA_1c_] ≥7.0%). A secondary effectiveness outcome was assessed by monitoring time to new incidence of any of 28 adverse outcomes, including diabetes-related complications while treated with the assigned drug. Sensitivity analysis included LOMCO.

**Results:**

This cohort study included 31 852 patients (16 635 [52.2%] male; mean [SD] age, 61.4 [12.6] years) who were new users of diabetes treatments added on to metformin monotherapy. Compared with sulfonylurea in random-effect meta-analysis, treatment with SGLT2I (summary hazard ratio [sHR], 0.75 [95% CI, 0.69-0.83]; *I*^2^ = 37.5%), DPP4I (sHR, 0.79 [95% CI, 0.75-0.84]; *I*^2^ = 0%), GLP1RA (sHR, 0.62 [95% CI, 0.57-0.68]; *I*^2^ = 23.6%) were effective in glycemic control; findings from LOMCO sensitivity analysis were similar. Treatment with SGLT2I showed no significant difference in effectiveness compared with GLP1RA (sHR, 1.26 [95% CI, 1.12-1.42]; *I*^2^ = 47.3%; no LOMCO) or DPP4I (sHR, 0.97 [95% CI, 0.90-1.04]; *I*^2^ = 0%). Patients treated with DPP4I and SGLT2I had fewer cardiovascular events compared with those treated with sulfonylurea (DPP4I: sHR, 0.84 [95% CI, 0.74-0.96]; *I*^2^ = 0%; SGLT2I: sHR, 0.78 [95% CI, 0.62-0.98]; *I*^2^ = 0%). Patients treated with a GLP1RA or SGLT2I were less likely to develop chronic kidney disease (GLP1RA: sHR, 0.75 [95% CI 0.6-0.94]; *I*^2^ = 0%; SGLT2I: sHR, 0.77 [95% CI, 0.61-0.97]; *I*^2^ = 0%), kidney failure (GLP1RA: sHR, 0.69 [95% CI, 0.56-0.86]; *I*^2^ = 9.1%; SGLT2I: sHR, 0.72 [95% CI, 0.59-0.88]; *I*^2^ = 0%), or hypertension (GLP1RA: sHR, 0.82 [95% CI, 0.68-0.97]; *I*^2^ = 0%; SGLT2I: sHR, 0.73 [95% CI, 0.58-0.92]; *I*^2^ = 38.5%) compared with those treated with a sulfonylurea. Patients treated with an SGLT2I, vs a DPP4I, GLP1RA, or sulfonylurea, were less likely to develop indicators of chronic hepatic dysfunction (sHR vs DPP4I, 0.68 [95% CI, 0.49-0.95]; *I*^2^ = 0%; sHR vs GLP1RA, 0.66 [95% CI, 0.48-0.91]; *I*^2^ = 0%; sHR vs sulfonylurea, 0.60 [95% CI, 0.44-0.81]; *I*^2^ = 0%), and those treated with a DPP4I were less likely to develop new incidence of hypoglycemia (sHR, 0.48 [95% CI, 0.36-0.65]; *I*^2^ = 22.7%) compared with those treated with a sulfonylurea.

**Conclusions and Relevance:**

These findings highlight familiar medication patterns, including those mirroring randomized clinical trials, as well as providing new insights underscoring the value of robust clinical data analytics in swiftly generating evidence to help guide treatment choices in diabetes.

## Key Points

**Question:**

Can the comparative effectiveness and safety associated with second-line pharmaceutical interventions in type 2 diabetes be assessed using clinical data across a multicenter health care system?

**Findings:**

This cohort study including 31 852 patients with diabetes monitored for 5 years and using clinical data analysis found that treatment with either a glucagon-like peptide-1 receptor agonist, sodium-glucose cotransporter-2 inhibitor, or dipeptidyl peptidase-4 inhibitor added to metformin monotherapy was effective compared with a sulfonylurea in maintaining glycemic control, with glucagon-like peptide-1 receptor agonist being more effective than dipeptidyl peptidase-4 inhibitor. These add-on treatments were associated with fewer diabetes-related cardiovascular and renal complications compared with a sulfonylurea and were safe, avoiding hypoglycemia.

**Meaning:**

These findings suggest that clinical data across a multisite health care system could be used to assess the comparative effectiveness and safety associated with treatments in diabetes and could help guide medical decisions.

## Teaser

This cohort study assesses the comparative effectiveness and safety associated with 4 treatments in diabetes when added to metformin monotherapy across a multisite health care system.

## Introduction

The annual prevalence of type 2 diabetes remains high, impacting an estimated 11.3% of the US population and 9.3% of people worldwide.^1,2^ Current clinical treatment guidelines recommend targeting glycated hemoglobin A_1c_ (HbA_1c_) less than 7% for most adults (to convert to proportion of total hemoglobin, multiply by 0.01),^3^ although there is heterogeneity across recommendations, ranging between 6.5% and 8%, depending on the target population and risk of various microvascular and macrovascular complications.^4,5^ Moderate glycemic control, defined by HbA_1c_ within 7% to 8%, has been shown to improve macrovascular and microvascular outcomes in individuals with diabetes.^6,7^ Metformin is a preferred glucose-lowering drug and is often recommended at treatment initiation for patients with diabetes due to its tolerability, efficacy, and low cost.^3,8,9^ However, intensification of treatment with an additional agent is often needed to maintain glycemic levels in the recommended range. Indeed, an estimated one-third of the US population with HbA_1c_ greater than 7% is prescribed an additional drug to maintain glycemic control.^10^ However, the choice of such an add-on treatment is ambiguous, owing to the lack of direct comparisons of currently available agents.^3,8,9^ Additionally, evidence emerging from the characterization of clinical data suggests that the actual choices clinicians make in adding another agent onto metformin is highly variable across health systems, both in the US and worldwide.^11,12,13^ Therefore, to help inform clinical decision-making, it is imperative to fill this knowledge gap by comparing the effectiveness and safety of agents added on to metformin in a head-to-head fashion. However, such rigorous comparison across multiple therapeutic options is simply not feasible in the context of classical randomized clinical trials (RCTs) for myriad reasons, including but not limited to cost, time, patient follow-ups, and the combinatorial nature of diabetes treatment options.

We systematically evaluated the comparative effectiveness and safety associated with 4 categories of diabetes drugs: sulfonylurea, dipeptidyl peptidase-4 inhibitor (DPP4I), glucagon-like peptide-1 receptor agonist (GLP1RA), and sodium-glucose cotransporter-2 inhibitor (SGLT2I) when added onto existing metformin therapy using a clinical database covering 8 million patients across 5 University of California (UC) academic health centers that have been linked to form UC Health (eMethods in Supplement 1). Our objective did not involve the emulation of any specific trial; rather, we endeavored to conduct an analysis following the conventions observed within a customary clinical trial framework under the purview of target trial emulation from observational data.^14,15,16^ Our study involved identifying new users of a given diabetes treatment, creating matched cohorts of patients prescribed a specific drug and a comparator agent, evaluating their effectiveness and safety in maintaining glycemic control, and assessing adverse outcomes over 5 years at each site. We performed meta-analysis of evidence gathered from each site, followed by leave-1-medical-center-out (LOMCO) influence analysis to assess the replicability and robustness (eMethods in Supplement 1).

## Methods

For this cohort study, the institutional review boards across the UC Health system have determined the research use of the deidentified clinical database was not human participants research; therefore, this study was exempt from further approval and informed consent. The study is reported following the Strengthening the Reporting of Observational Studies in Epidemiology (STROBE) reporting guideline. The analysis was conducted between January 2022 to April 2023 using data from electronic health records (EHR) starting January 2012 to April 2023. Patient demographic data were extracted from EHR. Race and ethnicity were not included in analysis because of incompleteness in the EHR. The patients were followed up for a 5-year monitoring period after treatment initiation. Detailed methods are provided in the eMethods in Supplement 1.

### Statistical Analysis

The clinical data from EHRs of more than 8 million individuals across UC Health were extracted, transformed, and loaded into a standardized Observational Medical Outcome Partnership Common Data Model (OMOP-CDM) with an overall data quality score of 95% based on plausibility, conformance, and completeness.^17,18^ New users of diabetes treatments were identified using predefined inclusion and exclusion criteria. Patients from each treatment cohort were 1:1 matched with patients in a comparator cohort based on high-dimensional propensity scores estimated using adaptive least absolute shrinkage and selection operator,^19^ leveraging their extensive pretreatment clinical history at each site individually. The Cox proportional hazards model was used to calculate hazard ratios (HRs) assessing the comparative effectiveness and safety of treatments in each of the matched comparator-treatment pair at each site independently. The evidence from each site were summarized using random-effects meta-analysis and further assessed for stability and robustness by implementing LOMCO influence analysis approach. *P* values were corrected for multiple hypotheses using false discovery rate correction. The summary HR (sHR) was considered reliable, stable, and significant if its 95% CI did not span 1, had *I*^2^ less than 60%, had corrected *P* < .10, and remained stable based on LOMCO analysis. All the calculations were performed using R statistical software version 3.6.3 (R Project for Statistical Computing). A detailed description of the methods is provided in the eMethods in Supplement 1.

## Results

### Population Characteristics

A total of 31 852 patients (16 635 [52.2%] male; mean [SD] age, 61.4 [12.6] years) who were new users of sulfonylurea, DPP4I, SGLT2I, and GLP1RA ages 18 years and older and who satisfied the study inclusion and exclusion criteria were identified across all of UC Health (Table; eMethods and eAppendix 1 in Supplement 1). Among these, the sulfonylurea group was the largest, with 14 029 new users, followed by DPP4I (7434 new users), GLP1RA (5472 new users), and SGLT2I (4917 new users) distributed across the sites. There was no statistical difference in age of females (mean [SD] age, 61.6 [3.6] years) and males (mean [SD] age, 61.4 [2.5] years) at the time of add-on treatment initiation across the sites, which is also in accordance with the known predominance of relatively older individuals receiving care across UC Health. Mean HbA_1c_ at the time of treatment initiation varied from 7.4% (GLP1RA) to 8.3% (sulfonylurea) (Table).

**Table.** Characteristics of the New Users of Diabetes Treatments in Addition to Metformin Considered for Comparative Effectiveness and Safety Analysis

Characteristic	Patients, No. (%)				
	UC-1	UC-2	UC-3	UC-4	UC-5
**Sulfonylureas**					
New users, No.	2409	1991	3140	1754	4735
Sex					
Female	1223 (50.8)	886 (44.5)	1449 (46.2)	766 (43.7)	2069 (43.7)
Male	1186 (49.2)	1105 (55.5)	1691 (53.9)	988 (56.3)	2666 (56.3)
Age, mean (SD), y					
Female	56.8 (13.6)	60.8 (14.3)	61.8 (12.4)	62.6 (13.4)	63.9 (12.8)
Male	59.1 (13.1)	62.0 (11.9)	60.2 (11.8)	62.5 (12.2)	63.0 (12.5)
HbA_1c_, mean (SD), %^a^	8.74 (2.1)	7.57 (1.8)	8.60 (2.4)	8.40 (2.0)	8.24 (2.2)
Baseline comorbidity^b^					
Cardiovascular disease	191 (7.9)	256 (12.9)	283 (9)	164 (9.4)	351 (7.4)
Stroke	69 (2.9)	68 (3.4)	69 (2.2)	43 (2.5)	115 (2.4)
Myocardial infarction	54 (2.2)	70 (3.5)	69 (2.2)	31 (1.8)	126 (2.7)
Heart failure	95 (3.9)	148 (7.4)	181 (5.8)	105 (6.0)	171 (3.6)
Chronic kidney disease	105 (4.4)	157 (7.9)	186 (5.9)	164 (9.4)	433 (9.1)
Baseline health care visits, mean (SD), No.^c^	27.86 (41.39)	29.99 (55.51)	38.44 (56.69)	29.16 (51.47)	23.46 (41.55)
**Dipeptidyl peptidase-4 inhibitor**					
New users, No.	1033	1226	1077	696	3402
Sex					
Female	518 (50.2)	621 (50.7)	531 (49.3)	352 (50.6)	1679 (49.4)
Male	515 (49.9)	605 (49.4)	546 (50.7)	344 (49.4)	1723 (50.7)
Age, mean (SD), y					
Female	64.7 (14.0)	65.5 (12.6)	62.9 (13.1)	65.8 (14.6)	65.9 (12.8)
Male	64.6 (12.9)	64.1 (12.7)	62.0 (12.4)	65.1 (12.3)	63.9 (12.6)
HbA_1c_, mean (SD), %^a^	7.9 (2.1)	7.0 (1.4)	7.9 (1.6)	7.6 (1.8)	7.5 (1.9)
Baseline comorbidity^b^					
Cardiovascular disease	122 (11.8)	161 (13.1)	110 (10.2)	84 (12.1)	262 (7.7)
Stroke	56 (5.4)	42 (3.4)	26 (2.4)	28 (4.0)	86 (2.5)
Myocardial infarction	30 (2.9)	37 (3.0)	36 (3.3)	17 (2.4)	63 (1.9)
Heart failure	57 (5.5)	109 (8.9)	73 (6.8)	46 (6.6)	140 (4.1)
Chronic kidney disease	98 (9.5)	143 (11.7)	77 (7.2)	76 (10.9)	270 (7.9)
Baseline health care visits, mean (SD), No.^c^	53.61 (87.68)	42.49 (69.36)	53.97 (81.84)	35.19 (59.66)	34.13 (73.05)
**Sodium-glucose cotransporter-2 inhibitor**					
New users, No.	754	923	531	486	2223
Sex					
Female	337 (44.7)	381 (41.3)	208 (39.2)	190 (39.1)	865 (38.9)
Male	417 (55.3)	542 (58.7)	323 (60.8)	296 (60.9)	1358 (61.1)
Age, mean (SD), y					
Female	59.9 (14.1)	63.3 (13.8)	61.9 (14.4)	64.2 (12.2)	60.6 (12.9)
Male	60.5 (12.6)	64.6 (12.5)	62.2 (12.2)	62.9 (12.2)	61.9 (12.3)
HbA_1c_, mean (SD), %^a^	8.3 (2.0)	7.3 (1.8)	8.1 (2.1)	7.9 (2.7)	7.5 (1.7)
Baseline comorbidity^b^					
Cardiovascular disease	120 (15.9)	224 (24.3)	135 (25.4)	104 (21.4)	261 (11.7)
Stroke	36 (4.8)	38 (4.1)	26 (4.9)	13 (2.7)	28 (1.3)
Myocardial infarction	40 (5.3)	60 (6.5)	45 (8.5)	34 (7.0)	92 (4.1)
Heart failure	80 (10.6)	178 (19.3)	112 (21.1)	82 (16.9)	192 (8.6)
Chronic kidney disease	67 (8.9)	148 (16.0)	68 (12.8)	55 (11.3)	171 (7.7)
Baseline health care visits, mean (SD), No.^c^	58.86 (92.29)	75.78 (114.83)	95.86 (121.38)	70.91 (95.83)	51.56 (79.74)
**Glucagon-like peptide-1 receptor agonist**					
New users, No.	487	1088	809	462	2626
Sex					
Female	291 (59.8)	618 (56.8)	464 (57.4)	268 (58.0)	1501 (57.2)
Male	196 (40.3)	470 (43.2)	345 (42.7)	194 (42.0)	1125 (42.8)
Age, mean (SD), y					
Female	55.4 (13.7)	58.0 (14.0)	56.2 (13.2)	55.0 (14.6)	56.1 (13.4)
Male	57.5 (13.7)	58.9 (13.0)	58.2 (12.8)	58.0 (13.8)	57.3 (12.8)
HbA_1c_, mean (SD), %^a^	7.9 (1.9)	6.8 (1.4)	7.7 (1.9)	8.0 (2.1)	7.1 (3.8)
Baseline comorbidity^b^					
Cardiovascular disease	36 (7.4)	116 (10.7)	67 (8.3)	27 (5.8)	133 (5.1)
Stroke	14 (2.9)	18 (1.7)	11 (1.4)	3 (0.7)	43 (1.6)
Myocardial infarction	12 (2.5)	30 (2.8)	18 (2.2)	8 (1.7)	42 (1.6)
Heart failure	18 (3.7)	80 (7.4)	48 (5.9)	17 (3.7)	64 (2.4)
Chronic kidney disease	31 (6.4)	99 (9.1)	47 (5.8)	37 (8)	121 (4.6)
Baseline health care visits, mean (SD), No.^c^	58.93 (73.64)	90.84 (125.47)	92.62 (107.31)	70.72 (100.36)	66.92 (86.28)

Abbreviations: HbA_1c_, glycated hemoglobin A_1c_; UC, University of California.

SI conversion factor: To convert HbA_1c_ to proportion of total hemoglobin, multiply by 0.01.

^a^ Means are calculated from HbA_1c_ values measured on or any time before the treatment assignment.

^b^ Baseline comorbidity is the prevalence of relevant conditions at the baseline calculated within 365 days of treatment initiation, including day 0.

^c^ Calculated as the mean of baseline number of clinical encounters on or before treatment assignment.

HbA_1c_ values were highly variable in our patient population viewed across UC Health. This included cohorts receiving each of the add-on medications, except for GLP1RA, which was not as variable (Table). We also saw a significant difference in the HbA_1c_ levels of females vs males at the time of add-on treatment initiation irrespective of which agent was being prescribed.

### Matched Cohorts

**Figure 1.**
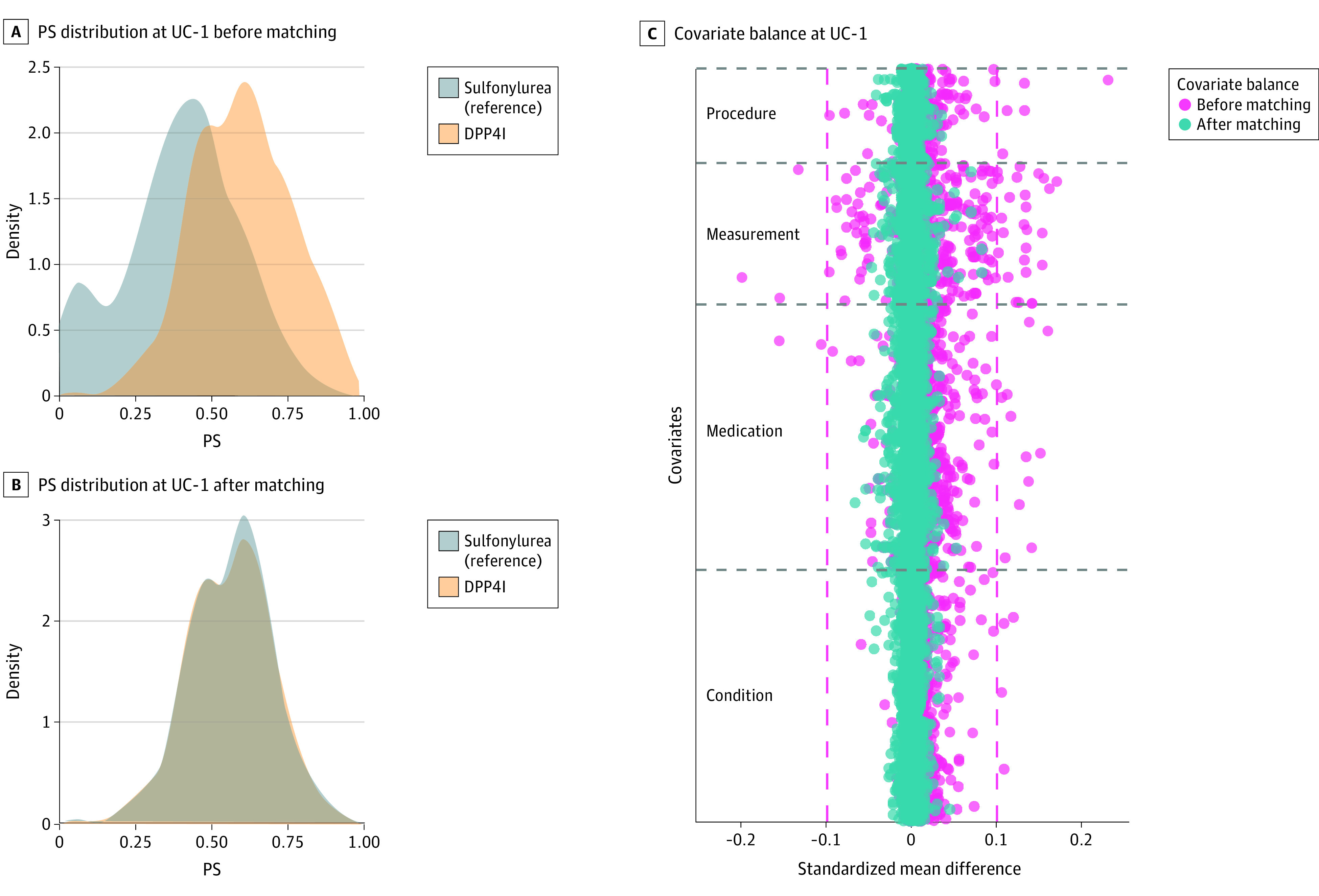
Propensity Score (PS) Matching and Covariate Balance Diagnostics C, The pink lines represent the standardize mean difference (−0.10 to 0.10), and the covariates outside of pink lines are considered imbalanced before and after matching. The PS distributions before and after matching along with covariate balance diagnostic of each other drug pair at each of the University of California (UC) Health site is illustrated in eAppendix 2 in Supplement 1.

We analyzed 30 pairs of comparator and treatment cohorts in which each pair represented a combination of 6 drug pairs from 4 categories of individual drugs prescribed as an add-on to metformin across the 5 sites. Patients from each treatment cohort were matched 1:1 with patients in a comparator cohort based on their estimated propensity scores at each site independently. Propensity score was estimated by accounting for a mean (SD) 4688.2 (643.2) pretreatment clinical covariates, along with age and sex of patients in each comparator-treatment pair (eAppendix 2 in Supplement 1). For instance, Figure 1 illustrates an example of propensity score matching for 1033 patients treated with a DPP4I (treatment cohort) and 2409 patients treated with a sulfonylurea (comparator cohort) at UC-1. In this example, the propensity scores were estimated by adjusting for 4429 clinical covariates, including medical procedures (556 covariates), laboratory measurements (832 covariates), unique orders of various drugs (1561 covariates), and medical conditions (1480 covariates) observed on or at least 365 days prior to the treatment initiation, in addition to age and sex (Figure 1C). After matching, all clinical features (100%) of the 4429 covariates were balanced (absolute SMD < 0.10), resulting in a final cohort of 1756 matched patients (Figure 1C). Detailed illustrations of the estimated propensity score distribution before and after matching along with the covariate balance diagnostic of each comparator-treatment pair for each site is provided in eAppendix 2 in Supplement 1.

### Comparative Effectiveness

**Figure 2.**
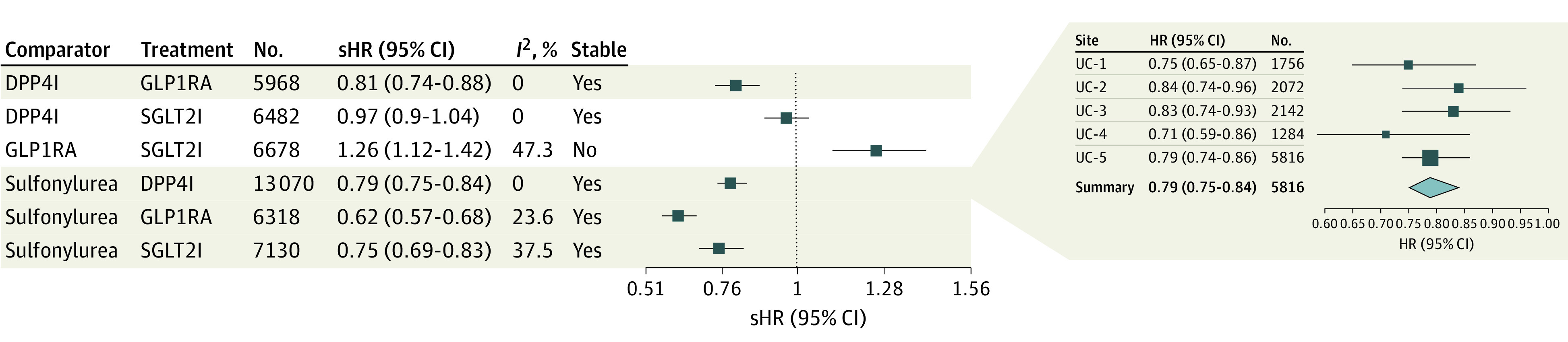
Comparative Effectiveness of Diabetes Treatments for the Ability of Patients to Maintain Glycemic Control Across the University of California (UC) Health System Glycemic control was defined as time to HbA_1c_ of 7% or greater (to convert to proportion of total hemoglobin, multiply by 0.01). Summary hazard ratios (sHRs) and 95% CIs were obtained from random-effect meta-analysis across the UC Health system, the heterogeneity (*I*^2^) of the sHRs and the stability of the sHRs are based on leave-1-medical-center-out influence analysis. The sub–forest plot shows the HRs of the comparative effectiveness of patients treated with DPP4I compared with sulfonylureas at each UC Health site along with the sHR obtained from random effect meta-analysis across UC Health as an example. Size of square indicates sample size. Detailed forest plots of each drug pair comparison at each UC Health site along with summary estimates across UC Health, including leave-1-medical-center-out influence analysis, are provided in eAppendix 3 in Supplement 1.

We analyzed the effectiveness in each matched comparator-treatment pair independently at each UC Health site in terms of a given patient’s ability to maintain glycemic control and then summarized the results using random-effect meta-analysis across all UCs with LOMCO influence analysis. The patients who received a DPP4I (summary hazard ratio [sHR], 0.79 [95% CI, 0.75-0.84]; *I*^2^ = 0%), GLP1RA (sHR, 0.62 [95% CI, 0.57-0.68]; *I*^2^ = 23.6%), or SGLT2I (sHR, 0.75 [95% CI, 0.69-0.83]; *I*^2^ = 37.5%) were significantly less likely to experience suboptimal glycemic control compared with those who were treated with a sulfonylurea with metformin during the 5-year posttreatment follow-up (Figure 2). The summary estimates of DPP4I, GLP1RA, and SGLT2I compared with sulfonylurea were found to be reliable based on the LOMCO influence analysis, indicating their robustness. There was no significant difference in the effectiveness associated with SGLT2I compared with DPP4I (sHR, 0.97 [95% CI, 0.90-1.04]; *I*^2^ = 0%). Across UCs, we observed better effectiveness of GLP1RA compared with DPP4I (sHR, 0.81 [95% CI, 0.74-0.88]; *I*^2^ = 0%).

Patients treated with a SGLT2I were more likely to experience metabolic failure than those treated with a GLP1RA (sHR, 1.26 [95% CI, 1.12-1.42]; *I*^2^ = 47.3%). However, this estimated summary difference between SGLT2I and GLP1RA was considered inconclusive because it was not stable based on the LOMCO analysis (Figure 2). A detailed illustration of the hazard ratios of each matched comparator-treatment pair at each site along with their summary estimates across all 5 UC Health sites, including LOMCO metric, is provided in eAppendix 3 in Supplement 1.

### Comparative Analysis of Diabetes Complications and Adverse Outcomes

We also analyzed each matched comparator and treatment pair independently at each UC Health site to evaluate the time it took for a new adverse outcome to occur within the 5-year follow-up period after treatment initiation, and then summarized the results using random-effect meta-analysis across all UCs with LOMCO influence analysis (eAppendix 4 and the eFigure in Supplement 1). Compared with patients treated with a sulfonylurea, patients treated with DPP4I were less likely to develop all-cause cardiovascular disease (sHR, 0.84 [95% CI, 0.74-0.96]; *I*^2^ = 0%) and specifically myocardial infarction (sHR, 0.75 [95% CI, 0.61-0.92]; *I*^2^ = 0%) (eFigure in Supplement 1). Compared with patients treated with a sulfonylurea, patients treated with SGLT2I also had lower hazards for all-cause cardiovascular disease (sHR, 0.78 [95% CI, 0.62-0.98]; *I*^2^ = 0%) and specifically stroke (hemorrhagic and ischemic combined: sHR, 0.55 [95% CI, 0.35-0.86]; *I*^2^ = 0%) (eFigure in Supplement 1). Patients treated with GLP1RA or SGLT2I, compared with those treated with a sulfonylurea, had a lower hazard for kidney failure (GLP1RA: sHR, 0.69 [95% CI, 0.56-0.86]; *I*^2^ = 9.1%; SGLT2I: sHR, 0.72 [95% CI, 0.59-0.88]; *I*^2^ = 0%) as well as chronic kidney disease (GLP1RA: sHR, 0.75 [95% CI 0.6-0.94]; *I*^2^ = 0%; SGLT2I: sHR, 0.77 [95% CI, 0.61-0.97]; *I*^2^ = 0%) (eFigure in Supplement 1). There were no differences in the renal outcomes of among patients treated with a GLP1RA vs a DPP4I, nor when comparing individuals treated with an SGLT2I vs a GLP1RA. However, patients treated with an SGLT2I had lower hazards for chronic kidney disease and kidney failure compared with those treated with a DPP4I. Patients treated with an SGLT2I had lower hazards of chronic liver disease after treatment compared with those treated with a DPP4I, GLP1RA, or sulfonylurea (eFigure in Supplement 1).

Hypoglycemia is an important concern whenever diabetes treatments are intensified to maintain strict glycemic control.^20,21^ We found that patients treated with a DPP4I had a lower hazard for hypoglycemia compared with those treated with a sulfonylurea (sHR, 0.48 [95% CI, 0.36-0.65]; *I*^2^ = 22.7%). Separately, we found that patients treated with a GLP1RA or SGLT2I had lower hazards for hypertension compared with those receiving a sulfonylurea (GLP1RA: sHR, 0.82 [95% CI, 0.68-0.97]; *I*^2^ = 0%; SGLT2I: sHR, 0.73 [95% CI, 0.58-0.92]; *I*^2^ = 38.5%) (eFigure in Supplement 1). There was no conclusive evidence regarding other acute adverse outcomes, such as abdominal pain, among all the comparator-treatment pairs, except for the GLP1RA group compared with the sulfonylurea group (sHR, 1.24 [95% CI, 1.04-1.49]; *I*^2^ = 0%) (eFigure in Supplement 1). We found that both treatment with an SGLT2I (compared with GLP1RA), and treatment with a GLP1RA (compared with sulfonylurea) had lower hazards for development of acute adverse outcomes, such as vomiting and nausea (eFigure in Supplement 1). With respect to bone fracture, we noted a lower hazard among patients treated with a SGLT2I compared with a DPP4I (sHR, 0.64 [95% CI, 0.46-0.88]; *I*^2^ = 0%) while no conclusive differences were found for other treatment comparisons (eFigure in Supplement 1). A detailed illustration of the hazard ratios of each matched comparator-treatment pair at each UC Health site along with their summary estimates across UCs, including LOMCO metrics, is provided in eAppendix 3 in Supplement 1.

## Discussion

**Figure 3.**
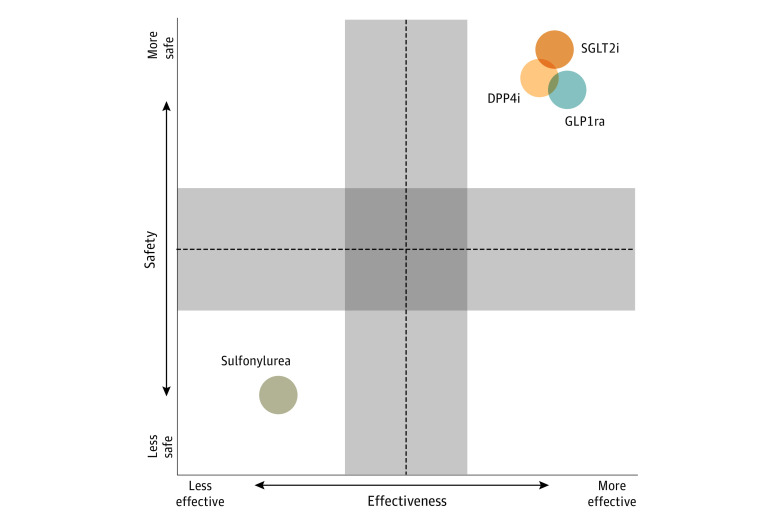
Visual Summary of the Findings From the Comparative Effectiveness and Safety Study Across the University of California Health System Shaded areas indicate low confidence in distinguishing difference in treatment effectiveness and safety.

The findings of this cohort study are meaningful with respect to guiding clinical care, especially when compared with RCT data and traditional observational studies. First, our analysis found that compared with sulfonylurea, treatment with either a GLP1RA, SGLT2I, or DPP4I was associated with effectively maintaining glycemic control in patients with diabetes when added to metformin monotherapy over a 5-year monitoring period (Figure 3). This finding is important because the drugs in question are not similarly priced. Moreover, whereas the concept of secondary failure, including in a 5-year period, is established for sulfonylurea, clinicians are grappling with whether there is differential effectiveness between the other classes as adjunctive agents to metformin. Our data-driven findings suggest more equivalency across classes with respect to HbA_1c_ control than may have been found from separate RCTs without a head-to-head element across classes.

We also found that treatment with a GLP1RA was associated with better glycemic control than a DPP4I. This mirrors what is expected based on the complementary mechanisms of action for each of these classes and validates findings from prior RCTs. By contrast, the comparison between SGLT2I and DPP4I yielded inconclusive results. We also observed a higher risk of metabolic failure among patients treated with a SGLT2I compared with those treated with a GLP1RA, although the stability and robustness of this evidence were weakened once our LOMCO influence analysis was conducted.

Our study found that adding DPP4I or SGLT2I to metformin monotherapy was associated with a significantly lower risk of new cardiovascular disease compared with sulfonylurea. At least for SGLT2I, this finding again mirrors what might be found in RCTs, which are increasingly showing that SGLT2Is are protective against congestive heart failure.^22,23^ Additionally, patients treated with a DPP4I had lower risk of myocardial infarction, and patients treated with an SGLT2I had lower risk of stroke. This too, aligns with findings from RCTs, which consistently show that GLP1RA and SGLT2I have potential for cardiovascular (GLP1RA) and cardiac (SGLT2I) benefit.^23^ The comparative evaluation of a specific DPP4I, linagliptin, vs a specific sulfonylurea, glimepiride, when added to metformin using a noninferiority design in the CAROLINA trial did not reveal a cardiovascular advantage to DPP4I.^24^ The trial’s findings suggested that when supplementary glucose-lowering therapy is needed, the DPP4I’s favorable attributes, including its lower risk of hypoglycemia and weight gain, make it a potentially suitable choice.^24^ In contrast, our analysis indicated not only that addition of a DPP4I was associated with cardiovascular benefits compared with addition of an sulfonylurea following metformin, but also that DPP4I treatment vs sulfonylurea was associated with superior glycemic control and lower likelihood of hypoglycemia, observations that align with CAROLINA, emphasizing DPP4I as a viable second-line option.

Adding a GLP1RA or SGLT2I to metformin also showed lower risks of new chronic kidney disease and kidney failure compared with a sulfonylurea.^25,26,27,28,29^ Once more, this comports with accumulated RCT findings, at least for SGLT2I. SGLT2I were associated with a lower risk of new chronic liver disease compared with DPP4I. Moreover, when added to metformin, SGLT2I, DPP4I, and GLP1RA were associated with lower risks of new nausea, abdominal pain, hypertension, and hypoglycemia compared with sulfonylurea. These findings contrast with those from RCTs evaluating GLP1RAs against placebo, in which nausea is highlighted as a class-wide adverse effect. Overall, our analysis highlights the effectiveness of diabetes medications, including SGLT2I, DPP4I, and GLP1RA, in glycemic control and accurately assesses impacts with respect to diabetes complications and potential adverse effects for patients with diabetes. Whereas some of our findings with respect to effectiveness and ancillary benefits mirror what has been found in much more costly RCTs, they extend across the entire class (whereas RCTs usually focus on only 1 drug) and highlight associations that either have not yet been probed by RCTs or that were not included in prior primary or secondary prespecified RCT outcomes.

The understanding that the cardioprotective effects of newer classes of antidiabetic drugs are primarily attributed to their pleiotropic properties rather than only to their glucose-lowering abilities has played a significant role in the broader management strategies.^26,30^ Sulfonylurea is a preferred add-on treatment to metformin monotherapy, potentially due to its low cost and long track record.^5^ This choice is widely practiced, including in health care systems like UC Health.^11,12^ However, sulfonylurea can cause relatively higher incidence of hypoglycemia compared with newer diabetes medications, which may limit their use.^31^ Our analysis showed that GLP1RA, SGLT2I, and DPP4I, the newer diabetes medications, were significantly associated with more effective glycemic control maintenance when added to metformin monotherapy compared with a sulfonylurea. Additionally, treatment with a DPP4I, compared with a sulfonylurea, was not only associated with effectively maintaining glycemic control but also a lower risk of hypoglycemia and adverse cardiac outcomes.

The GRADE trial comparing glargine (insulin), glimepiride (sulfonylurea), liraglutide (GLP1RA), and sitagliptin (DPP4I) pairwise indicated a similar efficacy of these 4 drugs in reducing HbA_1c_, with better efficacy of glargine and liraglutide when added to metformin monotherapy and no significant difference in the incidence of macrovascular or microvascular outcomes.^32,33^ Like the evidence from the GRADE trial, our findings indicated better effectiveness associated with a GLP1RA compared with a sulfonylurea when added to metformin monotherapy in maintaining glycemic control, in addition to their protective associations against cardiac, renal, and liver disorders as well as hypertension. Additionally, our analysis showed better effectiveness associated with use of a SGLT2I, which was not considered in the GRADE trial, in maintaining glycemic control along with their protective associations against cardiac, renal, and liver disorders, as well as hypertension. Thus, our study underscores the significance of evidence in not only reaffirming anticipated results but also generating evidence for drugs that were not included in the GRADE RCT.^32,33^

Previous investigations using clinical data have improved our understanding of the effectiveness and safety of treatments for diabetes.^27,34,35^ Recent evidence from OptumLabs, a nationwide claims database in the US, has provided similar findings to the GRADE trial, highlighting the importance of generating timely evidence.^36^ Initiatives such as RCT-Duplicate^37^ underscore the importance of cautiously evaluating clinical data, such as from EHR, owing to various types of underlying bias,^38,39,40^ as well as the need of further research to build frameworks to better emulate target trials. Our investigation presents a comprehensive framework for generating meaningful evidence using clinical data, incorporating the principles of emulating target trials in alignment with efforts, such as those represented by RCT-Duplicate.^37^ The reliability of evidence is bolstered through independent analyses of each UC Health campus, accounting for unique medical practices as well as correcting for multiple hypothesis testing. To assess evidence credibility, we performed LOMCO influence analysis, discerning the comparative strength of data from different health systems. The framework introduced in this study holds promise for informing future studies, facilitating the generation of meaningful evidence to drive effective medical decision-making.

### Limitations

This study has some limitations. Our study did not consider race and ethnicity of patients due to incompleteness of capturing such data elements in EHR, especially in outpatient and ambulatory encounters, as well as other factors, such as their socioeconomic backgrounds or the cost of treatment. We binarized (ie, present or absent) medical procedures, diagnoses, medications, and laboratory measurements to use as surrogate markers of the clinical state of a patient to adjust for potentially observed confounders. This approach has the potential to obscure the underlying rationale for treatment decisions established at the baseline.

The study population represents patients receiving care across California in any of the UC Health sites. Patients from outside of UC Health may occasionally receive care at a facility within UC Health, but we estimate that the proportion of such patients is small. However, this concept of fragmented care delivery with components of care delivered across multiple unconnected systems is both a potential limitation of our study and a potential limitation to the interpretation of our results by health systems located outside of California. However, UC Health represents a large number of patients, and California is a diverse state, suggesting that our results may be broadly applicable. Moreover, the issue of fragmented care delivery is a weakness of the US health care system as a whole and highlights the importance of building electronic health record tools with increasing capacity to capture care delivered within and outside of any given health system through interoperability. Given all of this, we acknowledge that determining the true status of new users of any given medication added on to metformin is challenging, given current tools and the limitations. We discuss limitations further in eAppendix 5 in Supplement 1.

## Conclusions

This cohort study used clinical data from 5 academic health centers within UC Health to assess the comparative effectiveness and safety associated with 4 pharmaceutical treatments added to metformin monotherapy for diabetes using the principles of emulating target trial. Findings from this study indicated that GLP1RAs, SGLT2Is, or DPP4Is, compared with sulfonylureas, were each effective in maintaining glycemic control in individuals with diabetes when added to metformin. Moreover, the study found that clinical data could be used to define drug-specific patterns with respect to the prevention of diabetes-associated complications, as well as potential drug-related adverse effects. Some of these patterns mirrored those found in dedicated RCTs, whereas others were new and likely would not have been possible to identify without access to data from so many patients. As such, statistically rigorous clinical data analytics could be used to rapidly generate evidence for both effectiveness and safety associated with pharmaceutical interventions to aid in enforcing beneficial shifts in clinical decision-making.

## Notes

Supplement 1.
eMethods.
**eAppendix 1.** Cohort Selection **eAppendix 2.** Patient Matching **eAppendix 3.** Comparative Effectiveness and Safety **eFigure.** Forest Plot of Summary Estimates of Secondary Outcomes **eAppendix 4.** Secondary Outcomes **eAppendix 5.** Discussion and Limitations

Supplement 2.
Data Sharing Statement

## References

[1] Centers for Disease Control and Prevention. National Diabetes Statistics Report. Accessed April 5, 2023. https://www.cdc.gov/diabetes/data/statistics-report/index.html

[2] Saeedi P, Petersohn I, Salpea P, ; IDF Diabetes Atlas Committee. Global and regional diabetes prevalence estimates for 2019 and projections for 2030 and 2045: Results from the International Diabetes Federation Diabetes Atlas, 9^th^ edition. Diabetes Res Clin Pract. 2019;157:107843. doi:10.1016/j.diabres.2019.107843 31518657

[3] American Diabetes Association. 6. Glycemic targets: *Standards of Medical Care in Diabetes—2020.* Diabetes Care. 2020;43(suppl 1):S66-S76. doi:10.2337/dc20-S006 31862749

[4] International Diabetes Federation. Recommendations for managing type 2 diabetes in primary care. Accessed April 5, 2023. http://www.idf.org/managing-type2-diabetes

[5] Davies MJ, Aroda VR, Collins BS, . Management of hyperglycaemia in type 2 diabetes, 2022: a consensus report by the American Diabetes Association (ADA) and the European Association for the Study of Diabetes (EASD). Diabetologia. 2022;65(12):1925-1966. doi:10.1007/s00125-022-05787-2 36151309 PMC9510507

[6] Hemmingsen B, Lund SS, Gluud C, . Intensive glycaemic control for patients with type 2 diabetes: systematic review with meta-analysis and trial sequential analysis of randomised clinical trials. BMJ. 2011;343:d6898. doi:10.1136/bmj.d6898 22115901 PMC3223424

[7] Rodriguez-Gutierrez R, Gonzalez-Gonzalez JG, Zuñiga-Hernandez JA, McCoy RG. Benefits and harms of intensive glycemic control in patients with type 2 diabetes. BMJ. 2019;367:l5887. doi:10.1136/bmj.l5887 31690574

[8] Garber AJ, Abrahamson MJ, Barzilay JI, . Consensus statement by the American Association of Clinical Endocrinologists and American College of Endocrinology on the comprehensive type 2 diabetes management algorithm—2019 executive summary. Endocr Pract. 2019;25(1):69-100. doi:10.4158/CS-2018-0535 30742570

[9] Conlin PR, Colburn J, Aron D, Pries RM, Tschanz MP, Pogach L. Synopsis of the 2017 U.S. Department of Veterans Affairs/U.S. Department of Defense Clinical practice guideline: management of type 2 diabetes mellitus. Ann Intern Med. 2017;167(9):655-663. doi:10.7326/M17-1362 29059687

[10] Fang M, Wang D, Coresh J, Selvin E. Trends in diabetes treatment and control in U.S. adults, 1999-2018. N Engl J Med. 2021;384(23):2219-2228. doi:10.1056/NEJMsa2032271 34107181 PMC8385648

[11] Hripcsak G, Ryan PB, Duke JD, . Characterizing treatment pathways at scale using the OHDSI network. Proc Natl Acad Sci U S A. 2016;113(27):7329-7336. doi:10.1073/pnas.1510502113 27274072 PMC4941483

[12] Peterson TA, Fontil V, Koliwad SK, Patel A, Butte AJ. Quantifying variation in treatment utilization for type 2 diabetes across five major University of California health systems. Diabetes Care. 2021;44(4):908-914. doi:10.2337/dc20-0344 33531419 PMC7985428

[13] McCoy RG, Van Houten HK, Deng Y, . Comparison of diabetes medications used by adults with commercial insurance vs Medicare Advantage, 2016 to 2019. JAMA Netw Open. 2021;4(2):e2035792. doi:10.1001/jamanetworkopen.2020.35792 33523188 PMC7851726

[14] Matthews AA, Danaei G, Islam N, Kurth T. Target trial emulation: applying principles of randomised trials to observational studies. BMJ. 2022;378:e071108. doi:10.1136/bmj-2022-071108 36041749

[15] Hernán MA, Wang W, Leaf DE. Target trial emulation: a framework for causal inference from observational data. JAMA. 2022;328(24):2446-2447. doi:10.1001/jama.2022.21383 36508210

[16] Franklin JM, Patorno E, Desai RJ, . Emulating randomized clinical trials with nonrandomized real-world evidence studies: first results from the RCT DUPLICATE initiative. Circulation. 2021;143(10):1002-1013. doi:10.1161/CIRCULATIONAHA.120.051718 33327727 PMC7940583

[17] Dixon BE, Wen C, French T, Williams JL, Duke JD, Grannis SJ. Extending an open-source tool to measure data quality: case report on Observational Health Data Science and Informatics (OHDSI). BMJ Health Care Inform. 2020;27(1). doi:10.1136/bmjhci-2019-100054 PMC7254131 32229499

[18] Hripcsak G, Duke JD, Shah NH, . Observational Health Data Sciences and Informatics (OHDSI): Opportunities for observational researchers. Stud Health Technol Inform. 2015;216:574-578. 26262116 PMC4815923

[19] Hastie T, Tibshirani R, Friedman J. The Elements of Statistical Learning. 2nd ed. Springer; 2017.

[20] Anderson M, Powell J, Campbell KM, Taylor JR. Optimal management of type 2 diabetes in patients with increased risk of hypoglycemia. Diabetes Metab Syndr Obes. 2014;7:85-94. 24623984 10.2147/DMSO.S48896 PMC3949696

[21] Karter AJ, Lipska KJ, O’Connor PJ, ; SUPREME-DM Study Group. High rates of severe hypoglycemia among African American patients with diabetes: the surveillance, prevention, and Management of Diabetes Mellitus (SUPREME-DM) network. J Diabetes Complications. 2017;31(5):869-873. doi:10.1016/j.jdiacomp.2017.02.009 28319006 PMC5491095

[22] McGuire DK, Shih WJ, Cosentino F, . Association of SGLT2 inhibitors with cardiovascular and kidney outcomes in patients with type 2 diabetes: a meta-analysis. JAMA Cardiol. 2021;6(2):148-158. doi:10.1001/jamacardio.2020.4511 33031522 PMC7542529

[23] Giugliano D, Longo M, Signoriello S, . The effect of DPP-4 inhibitors, GLP-1 receptor agonists and SGLT-2 inhibitors on cardiorenal outcomes: a network meta-analysis of 23 CVOTs. Cardiovasc Diabetol. 2022;21(1):42. doi:10.1186/s12933-022-01474-z 35296336 PMC8925229

[24] Rosenstock J, Kahn SE, Johansen OE, ; CAROLINA Investigators. Effect of linagliptin vs glimepiride on major adverse cardiovascular outcomes in patients with type 2 diabetes: the CAROLINA randomized clinical trial. JAMA. 2019;322(12):1155-1166. doi:10.1001/jama.2019.13772 31536101 PMC6763993

[25] Standl E, Schnell O, McGuire DK, Ceriello A, Rydén L. Integration of recent evidence into management of patients with atherosclerotic cardiovascular disease and type 2 diabetes. Lancet Diabetes Endocrinol. 2017;5(5):391-402. doi:10.1016/S2213-8587(17)30033-5 28131656

[26] Cosentino F, Grant PJ, Aboyans V, ; ESC Scientific Document Group. 2019 ESC guidelines on diabetes, pre-diabetes, and cardiovascular diseases developed in collaboration with the EASD. Eur Heart J. 2020;41(2):255-323. doi:10.1093/eurheartj/ehz486 31497854

[27] Xie Y, Bowe B, Gibson AK, McGill JB, Maddukuri G, Al-Aly Z. Comparative effectiveness of sodium-glucose cotransporter 2 inhibitors vs sulfonylureas in patients with type 2 diabetes. JAMA Intern Med. 2021;181(8):1043-1053. doi:10.1001/jamainternmed.2021.2488 34180939 PMC8240007

[28] Zelniker TA, Wiviott SD, Raz I, . SGLT2 inhibitors for primary and secondary prevention of cardiovascular and renal outcomes in type 2 diabetes: a systematic review and meta-analysis of cardiovascular outcome trials. Lancet. 2019;393(10166):31-39. doi:10.1016/S0140-6736(18)32590-X 30424892

[29] Lo KB, Gul F, Ram P, . The effects of SGLT2 inhibitors on cardiovascular and renal outcomes in diabetic patients: a systematic review and meta-analysis. Cardiorenal Med. 2020;10(1):1-10. doi:10.1159/000503919 31743918

[30] Gerstein HC, Colhoun HM, Dagenais GR, ; REWIND Investigators. Dulaglutide and renal outcomes in type 2 diabetes: an exploratory analysis of the REWIND randomised, placebo-controlled trial. Lancet. 2019;394(10193):131-138. doi:10.1016/S0140-6736(19)31150-X 31189509

[31] Gangji AS, Cukierman T, Gerstein HC, Goldsmith CH, Clase CM. A systematic review and meta-analysis of hypoglycemia and cardiovascular events: a comparison of glyburide with other secretagogues and with insulin. Diabetes Care. 2007;30(2):389-394. doi:10.2337/dc06-1789 17259518

[32] Nathan DM, Lachin JM, Bebu I, ; GRADE Study Research Group. Glycemia reduction in type 2 diabetes—microvascular and cardiovascular outcomes. N Engl J Med. 2022;387(12):1075-1088. doi:10.1056/NEJMoa2200436 36129997 PMC9832916

[33] Nathan DM, Lachin JM, Balasubramanyam A, ; GRADE Study Research Group. Glycemia reduction in type 2 diabetes—glycemic outcomes. N Engl J Med. 2022;387(12):1063-1074. doi:10.1056/NEJMoa2200433 36129996 PMC9829320

[34] Vashisht R, Jung K, Schuler A, . Association of hemoglobin A1c levels with use of sulfonylureas, dipeptidyl peptidase 4 inhibitors, and thiazolidinediones in patients with type 2 diabetes treated with metformin: analysis from the Observational Health Data Sciences and Informatics Initiative. JAMA Netw Open. 2018;1(4):e181755. doi:10.1001/jamanetworkopen.2018.1755 30646124 PMC6324274

[35] Lee KA, Jin HY, Kim YJ, Kim SS, Cho EH, Park TS. Real-world comparison of mono and dual combination therapies of metformin, sulfonylurea, and dipeptidyl peptidase-4 inhibitors using a common data model: a retrospective observational study. Medicine (Baltimore). 2022;101(8):e28823. doi:10.1097/MD.0000000000028823 35212277 PMC8878728

[36] Deng Y, Polley EC, Wallach JD, . Emulating the GRADE trial using real world data: retrospective comparative effectiveness study. BMJ. 2022;379:e070717. doi:10.1136/bmj-2022-070717 36191949 PMC9527635

[37] Wang SV, Schneeweiss S, Franklin JM, ; RCT-DUPLICATE Initiative. Emulation of randomized clinical trials with nonrandomized database analyses: results of 32 clinical trials. JAMA. 2023;329(16):1376-1385. doi:10.1001/jama.2023.4221 37097356 PMC10130954

[38] Agniel D, Kohane IS, Weber GM. Biases in electronic health record data due to processes within the healthcare system: retrospective observational study. BMJ. 2018;361:k1479. doi:10.1136/bmj.k1479 29712648 PMC5925441

[39] Verheij RA, Curcin V, Delaney BC, McGilchrist MM. Possible sources of bias in primary care electronic health record data use and reuse. J Med Internet Res. 2018;20(5):e185. doi:10.2196/jmir.9134 29844010 PMC5997930

[40] Weber GM, Adams WG, Bernstam EV, . Biases introduced by filtering electronic health records for patients with “complete data”. J Am Med Inform Assoc. 2017;24(6):1134-1141. doi:10.1093/jamia/ocx071 29016972 PMC6080680

