# Error in Byline

**DOI:** 10.1001/jamanetworkopen.2023.44113

**Published:** 2023-11-07

In the Original Investigation titled “Second-Line Pharmaceutical Treatments for Patients with Type 2 Diabetes,”^1^ published October 2, 2023, there was an error in degrees provided for coauthor Kathryn E. Medders, PharmD, PhD. This article has been corrected.^1^

## References

[1] Vashisht R, Patel A, Dahm L, . Second-line pharmaceutical treatments for patients with type 2 diabetes. JAMA Netw Open. 2023;6(10):e2336613. doi:10.1001/jamanetworkopen.2023.36613 37782497 PMC10546239

