# Supplementary material for: Second-Line Pharmaceutical Treatments for Patients with Type 2 Diabetes
Source: JAMA Netw Open. 2023 Oct 2;6(10):e2336613. doi: 10.1001/jamanetworkopen.2023.36613 (PMC10546239; doi:10.1001/jamanetworkopen.2023.36613)
Supplement: Supplement 1. — eMethods. eAppendix 1. Cohort Selection eAppendix 2. Patient Matching eAppendix 3. Comparative Effectiveness and Safety eFigure. Forest Plot of Summary Estimates of Secondary Outcomes eAppendix 4. Secondary Outcomes eAppendix 5. Discussion and Limitations [file jamanetwopen-e2336613-s001.pdf]

Supplemental Online Content

Vashisht R, Patel A, Dahm L, et al. Second-line pharmaceutical treatments for patients with type 2 diabetes. *JAMA Netw Open*. 2023;6(10):e2336613. doi:10.1001/jamanetworkopen.2023.36613

eMethods.
eAppendix 1. Cohort Selection
eAppendix 2. Patient Matching
eAppendix 3. Comparative Effectiveness and Safety
eFigure. Forest Plot of Summary Estimates of Secondary Outcomes
eAppendix 4. Secondary Outcomes
eAppendix 5. Discussion and Limitations

This supplemental material has been provided by the authors to give readers additional information about their work.

1 eMethods

1.1 Overall Study Design

We systematically evaluated the comparative effectiveness and safety of four categories of T2D drugs, sulfonylurea (SU), dipeptidyl peptidase-4 inhibitors (DPP4i), sodium-glucose cotransporter-2 inhibitors (SGLT2i) and glucagon-like peptide-1 receptor agonists (GLP1RA) when added onto existing metformin therapy using a clinical database covering 8 million patients across five University of California (UC) academic health centers that have been linked to form “UC Health” (eFigure 1). Our objective did not involve the emulation of any specific trial; rather, we endeavored to conduct an analysis following the conventions observed within a customary clinical trial framework under the purview of target trial emulation from observational data[1-3]. Our study had five main steps. First, we constructed several paired cohorts of individuals, each of which included those newly prescribed a given drug, the “treatment cohort” (Tc), and those prescribed a comparator agent, the “comparator cohort” (Cc), following a set of inclusion and exclusion criteria (eFigure 1B-C) applied at each site independently. Second, we matched patients in the Tc with patients in the Cc based on high-dimensional propensity scores estimation and evaluation using extensive pre-treatment clinical history at the individual patient-level, leveraging data at each site to do so (eFigure 1D). Third, we compared the matched patients in the Tc and Cc with respect to their relative effectiveness in maintaining glycemic control, evaluated as the time to observed metabolic failure defined by HbA1c ≥ 7% for the first time following initiation of a given add-on treatment, and the time to first incidence of any of the 28 clinical adverse outcomes as treatment safety over 5 years post-treatment (eFigure 1E). The adverse outcomes were selected based on commonly known treatment complications in T2D related to cardiovascular, renal, ophthalmological, other organ dysfunction, hypoglycemia, and hypertension[4,5]. Fourth, we followed the same first three steps at each UC Health site independently and obtained consensus estimates of effectiveness and safety using random-effect meta-analysis across the entirety of UC Health to confirm consensus evidence (eFigure 1F-G). Finally, we performed a leave-one-medical-center-out (LOMCO) influence analysis to quantify and assess the stability and robustness of evidence across all of UC Health (eFigure 1H-I).

© 2023 Vashisht R et al. *JAMA Network Open.*

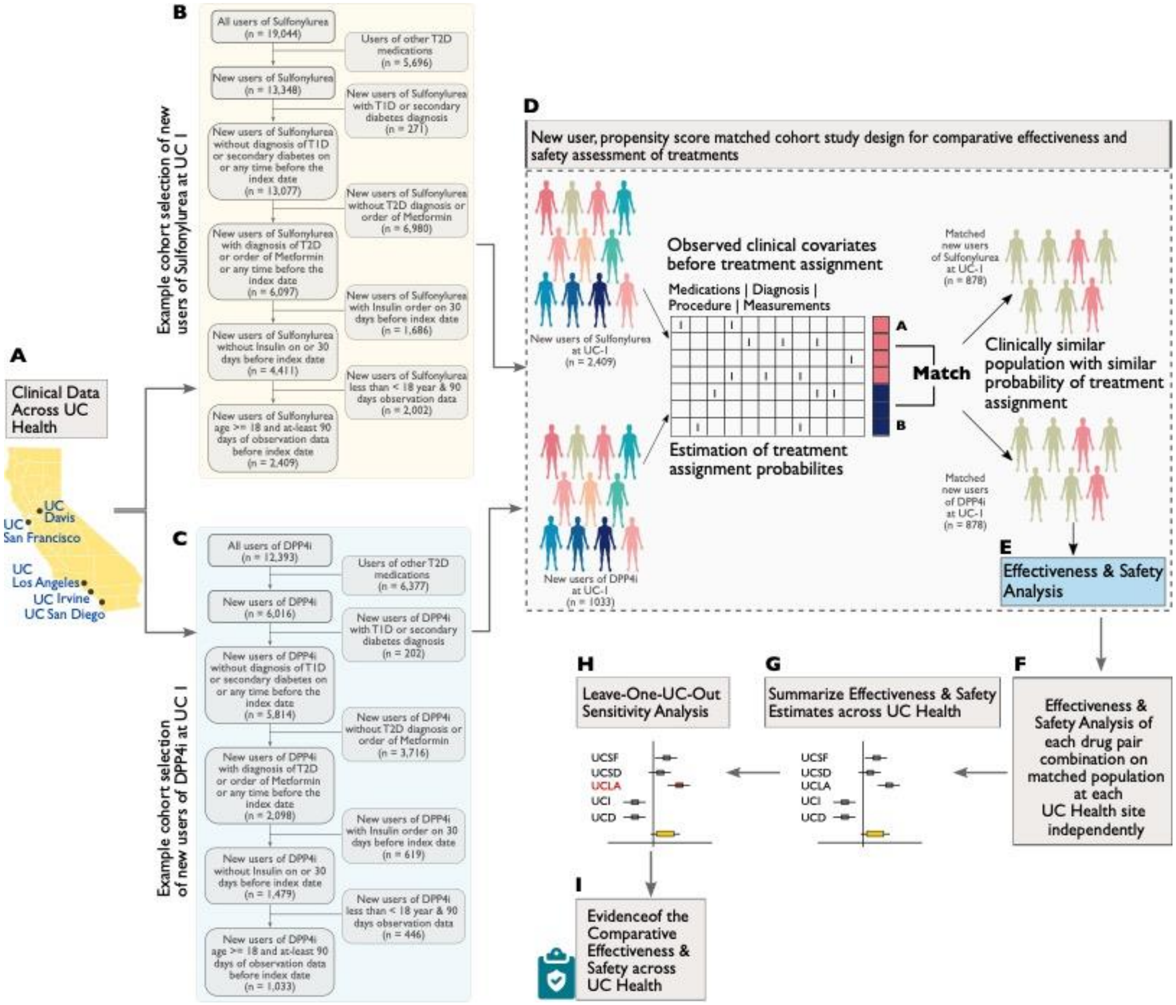

eFigure 1: Overview of the study design and implementation: A) UC Health sites across California considered in this analysis. B-C) Inclusion-exclusion criteria of new user cohort selection at one of the UC (UC-1) for SU (comparator cohort) and DPP4i (treatment cohort) as an example. D) Patient matching- A patient feature design matrix with medication, diagnosis, laboratory measurements and medical procedures as covariates observed on or 365 days before treatment initiation is constructed to estimate propensity scores and 1:1 match patient in treatment cohort to the patients in comparator cohort E) Comparative effectiveness and safety analysis. F) Execution of the study using the process highlighted in B-F for each comparator and treatment cohort pair at each UC Health site. G-H) Meta-analysis of each comparator and treatment cohort across UC Health to obtain summary estimates followed by leave-one-medical-center-out influence analysis to assess the stability of the summary estimate I) Reporting of the summary estimates of effectiveness and safety of second-line treatments in T2D.

© 2023 Vashisht R et al. *JAMA Network Open.*

1.2 Data Source

Data for this study was drawn from the UC Health Data Warehouse (UCHDW), a Health Insurance Portability and Accountability Act (HIPAA) compliant de-identified set of electronic health records (EHRs) of over 8 million patients receiving care over 11 years, across 6 academic health centers and 12 hospitals with approximately 150,000 inpatient and 4 million outpatients visit annually. UCHDW constitutes de-identified medical records from six independent academic health centers: UC San Francisco, UC San Diego, UC Davis, UC Irvine, UC Los Angeles, and UC Riverside. UC Riverside Health was established only 9 years ago, and based on the limited number of current patients with T2D, was excluded from this study. The remaining UC sites were randomly anonymized as UC-1, UC-2, UC-3, UC-4 and UC-5 in the analysis. The de-identified EHR data were extracted, transformed, and loaded into a database using the standardized Observational Medical Outcome Partnership Common Data Model (OMOP-CDM)[6]. We performed a comprehensive quality assessment of the UCHDW OMOP-CDM and achieved an overall data quality score of 95% based on plausibility, conformance and completeness of various data elements metrics using data quality dashboard implemented via observational health data science and informatics[6,7]. The analysis was conducted between January 2022 to April 2023 using data from electronic health records starting January 2012 to April 2023. The patients were followed for 5-year monitoring period post treatment initiation.

1.3 New User Cohort

New user cohorts for T2D drugs used to intensify T2D treatment on top of metformin were identified using pre-specified inclusion and exclusion criteria. As an example, for the SU, first, all users of SU were identified based on the date on which SU were ordered for the first time in a patient's medical record; this date was considered as the index date for SU (eFigure 1B). Next, patients indicated to be new SU users were excluded if they had an order placed for another T2D drug other than metformin before the SU index date, indicating that the SU prescription indicated a switch from this other drug, as opposed to a simple add-on to metformin. Next, new users were excluded if they had a mention of Type 1 diabetes (T1D) or secondary diabetes on or any time prior to the index date. Additionally, new users were excluded if there was no mention of T2D diagnosis and an order of metformin on or any time prior to the index date. Next, new users were excluded if they had an order for insulin on or up to 30 days before the index date. Finally, new users were excluded if they were less than 18 years of age on the index date and lacked at least 90 days of continuous enrollment before the index date. The UC population predominantly comprises individuals whose funding is facilitated by their employers. While a minor proportion of individuals may be referred to UC from external sources, this fraction remains minimal. In order to establish a robust representation of patients primarily under the purview of the UC health plan, we established a prerequisite for patients to possess a minimum of 90 days' worth of observation data prior to the initiation of their treatment. This measure was undertaken to ensure the inclusion of patients whose healthcare funding is predominantly derived from UC. Furthermore, the decision to opt for a 90-day interval was primarily driven by the intention to encompass an adequate observation period for patients within UC Health system. This duration is likely to account for potential prescription refills and ensure a comprehensive assessment. The STROBE flow diagrams of the total number of patients following the study inclusion and exclusion criteria for each T2D second-line drugs at each of the UC sites is provided in eFigure 2-21 of this document.

1.4 Propensity Score Estimation, Matching and Covariate Balance Diagnostics

The propensity scores (PS) – conditional probabilities of treatment assignment – were estimated using adaptive least absolute shrinkage and selection operator approach (adaptive LASSO) with 10 fold cross validation to select the best hyperparameters[8,9]. First, we constructed a high-dimensional binary patient feature design matrix (PFDM) for a pair of Cc-Tc (eFigure-1 D). The rows of the PFDM represented patients in Cc and Tc and the columns were the features such as age at the index date, sex, medical conditions, medication use, laboratory test orders, and medical procedures observed on or 365 days prior to the index date (eFigure-1 D). The PFDM was then subjected to adaptive LASSO with a 10-fold cross validation for

© 2023 Vashisht R et al. *JAMA Network Open.*

hyperparameter tuning and the propensity scores were obtained after fitting the trained model with optimal hyperparameter at λ.1se (largest value of λ such that errors is within 1 standard error of the cross-validation errors for λ.min) on full data8. Next, the patients from the Tc group were 1:1 matched to the patients in the Cc group based on their estimated PS using nearest neighbor matching with a caliper of 0.20 on the logit scale[10]. The quality of the matched cohort was quantified and assessed by calculating covariate balance diagnostics using standardize mean difference (SMD) before and after PS matching. The covariates with an absolute SMD < 0.10 post matching were considered balanced[11]. Patients from the Tc were matched to the patients in the Cc for each Tc-Cc combination independently using data from each UC Health site independently. All calculations were performed using glmnet[12] and R statistical software version 3.6.3[13].

1.5 Treatment Effectiveness and Safety Assessment

The Cox Proportional-Hazard model was used to calculate hazard ratios assessing the comparative effectiveness and safety of T2D second-line treatments in each of the matched Cc-Tc pair at each UC independently. To assess the effectiveness of treatment, the matched Cc and Tc were compared for the patient's ability to maintain glycemic control calculated as time to metabolic failure i.e., time to HbA1c ≥ 7% starting one day after treatment initiation up to 5 years of follow-up. One day after treatment initiation was selected to minimize the day 0 bias where an HbA1c could be reported on the day of treatment assignment. To assess the safety of treatment, the matched cohort were compared for time-to new incidence of 28 adverse outcomes related to cardiovascular-, renal-, liver- and eye-disorders including hypertension and hypoglycemia starting one day after treatment initiation up to 5 years of follow-up. Different levels of phenotypic hierarchy were considered in defining the secondary outcomes. For instance, all-cause cardiovascular disease included acute myocardial infarction, sudden cardiac death, ischemic or hemorrhagic stroke, and heart failure. Details on the phenotypic definitions of 28 adverse outcomes considered in this study is provided in eTables 205-232. The follow-up period for each patient was considered right-censored if a non-metformin treatment was prescribed at any point following treatment initiation, within the 5-year monitoring period. However, post-treatment follow-up for patients who potentially underwent non-metformin treatments beyond the initial 5-year monitoring period was not subject to censorship. In assessing safety, patients who might have experienced the adverse outcome in question prior to treatment assignment were removed from the analysis. The hazard ratios (HR), 95% confidence intervals, and p-values were obtained using the Cox Proportional-Hazard model. The p-values were corrected for multiple hypotheses using false discovery rate correction. An HR was considered significant when its 95% confidence intervals did not span 1 with adjusted p-value < 10%. All the calculations were performed using survival package[14] and R statistical software version 3.6.3[13].

1.6 Summary Estimates, Reliability and Stability Assessment with Leave-One-Medical-Center-Out Influence Analysis

The summary estimates of the comparative effectiveness and safety of a given Cc-Tc across UC Health were obtained using random effect meta-analysis with restricted maximum-likelihood estimator and represented as summary hazard ratio (sHR)[15]. The I^2 statistic describing the percentage of variation across studies due to heterogeneity was calculated and reported as a measure of evidence of reliability. The stability of sHR was assessed using leave-one-medical-center-out (LOMCO) influence analysis[16]. Multiple diagnostic metrics such as a) Hat matrix, b) difference in fits (DFFITS), c) Cook's distance, d) leave-one-out amount of residual heterogeneity, e) leave-one-out test statistic of the test for residual heterogeneity, and f) DFBETAS values were calculated to assess the influence of a given Cc-Tc comparison at one site with respect to the remaining sites. The HR obtained from a given site was considered influential to the pooled sHR if a) the associated absolute DFFITS value was larger than 3 * sqrt (p/(k - p)), where k is the number of UCs (k = 5) and p is the number of model coefficients b) the lower tail area of a chi-square distribution with p degree of freedom cut off by the Cook's distance is > 50% c) the had value > 3 * (p/k) and d) any DFBETAS > 1. The sHR was considered stable if the estimated HR in 4 out of the 5 UC Health sites were non-influential to the pooled sHR. The p-values associated with sHR were corrected for multiple hypothesis using false discovery rate adjustment. The sHR was considered reliable, stable, and significant if it's 95% confidence intervals

© 2023 Vashisht R et al. *JAMA Network Open.*

did not span 1, had I2 < 60%, had corrected p-value < 10%, and if stable based on LOMCO analysis. The strength and direction of sHR was interpreted as real-world evidence of the comparative effectiveness and safety of T2D drugs. All the calculations were performed using R statistical software version 3.6.3[13].

Reference

1. Matthews AA, Danaei G, Islam N, Kurth T. Target trial emulation: applying principles of randomised trials to observational studies. BMJ. 2022;378:e071108.
2. Hernán MA, Wang W, Leaf DE. Target Trial Emulation: A Framework for Causal Inference From Observational Data. JAMA. Published online December 12, 2022. doi:10.1001/jama.2022.21383
3. Franklin Jessica M., Patorno Elisabetta, Desai Rishi J., et al. Emulating Randomized Clinical Trials With Nonrandomized Real-World Evidence Studies. Circulation. 2021;143(10):1002-1013.
4. Harding JL, Pavkov ME, Magliano DJ, Shaw JE, Gregg EW. Global trends in diabetes complications: a review of current evidence. Diabetologia. 2019;62(1):3-16.
5. Deshpande AD, Harris-Hayes M, Schootman M. Epidemiology of diabetes and diabetes-related complications. Phys Ther. 2008;88(11):1254-1264.
6. Hripcsak G, Duke JD, Shah NH, et al. Observational Health Data Sciences and Informatics (OHDSI): Opportunities for observational researchers. Stud Health Technol Inform. 2015;216:574-578.
7. Dixon BE, Wen C, French T, Williams JL, Duke JD, Grannis SJ. Extending an open-source tool to measure data quality: case report on Observational Health Data Science and Informatics (OHDSI). BMJ Health Care Inform. 2020;27(1). doi:10.1136/bmjhci-2019-100054
8. Hastie T, Tibshirani R, Friedman J. The Elements of Statistical Learning. 2nd ed. Springer; 2017.
9. Hui ZOU. The Adaptive Lasso and Its Oracle Properties. doi:10.1198/016214506000000735
10. Austin PC. Optimal caliper widths for propensity-score matching when estimating differences in means and differences in proportions in observational studies. Pharm Stat. Published online 2011. https://onlinelibrary.wiley.com/doi/abs/10.1002/pst.433
11. Austin PC. Balance diagnostics for comparing the distribution of baseline covariates between treatment groups in propensity-score matched samples. Stat Med. Published online 2009. https://onlinelibrary.wiley.com/doi/abs/10.1002/sim.3697
12. Friedman J, Tibshirani R, Hastie T. Regularization Paths for Generalized Linear Models via Coordinate Descent. Journal of Statistical Software. 2010;33(1):1-22. doi:10.18637/jss.v033.i01
13. R Core Team. R: A language and environment for statistical computing. Published online 2020. https://www.R-project.org/
14. Therneau TM, Grambsch PM. Modeling Survival Data: Extending the COx Model. Springer Science & Business Media; 2000.
15. Schwarzer G, Carpenter JR, Rucker G. Meta-Analysis with R. 1st ed. Springer International Publishing; 2015.
16. Viechtbauer W, Cheung MWL. Outlier and influence diagnostics for meta-analysis. Res Synth Methods. 2010;1(2):112-125

© 2023 Vashisht R et al. *JAMA Network Open.*

eAppendix 1. Cohort Selection

Each flow diagram represents a STROBE chart indicating number of patients on a given anti-diabetic drug at a given anonymized UC health site based on the inclusion criteria.

1.7 eFigure: Cohort selection Sulfonylurea

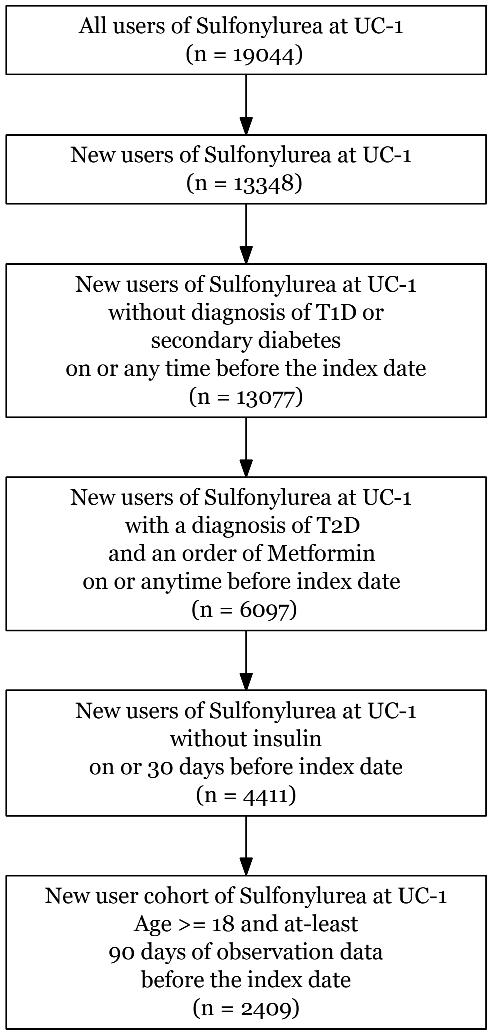

eFigure 2: Sulfonylurea cohort UC-1

© 2023 Vashisht R et al. *JAMA Network Open.*

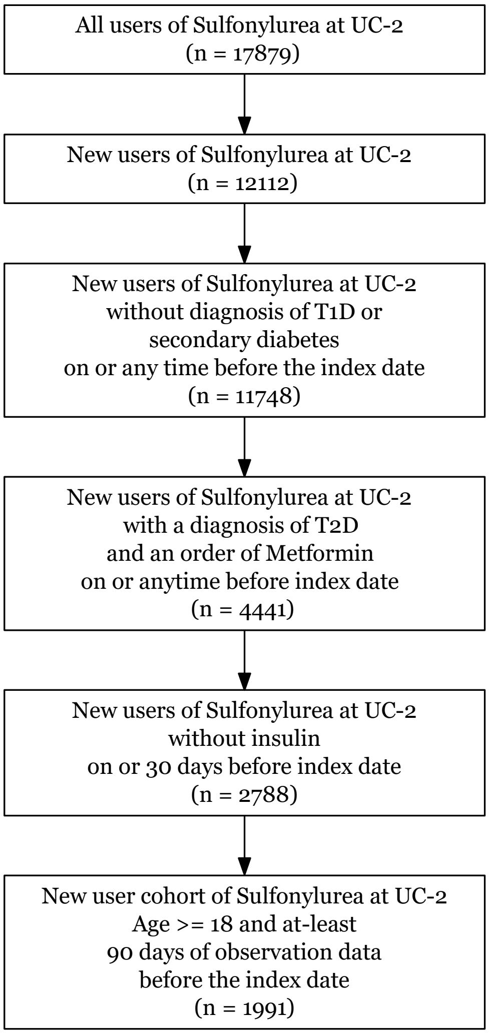

eFigure 3: Sulfonylurea cohort UC-2

© 2023 Vashisht R et al. *JAMA Network Open.*

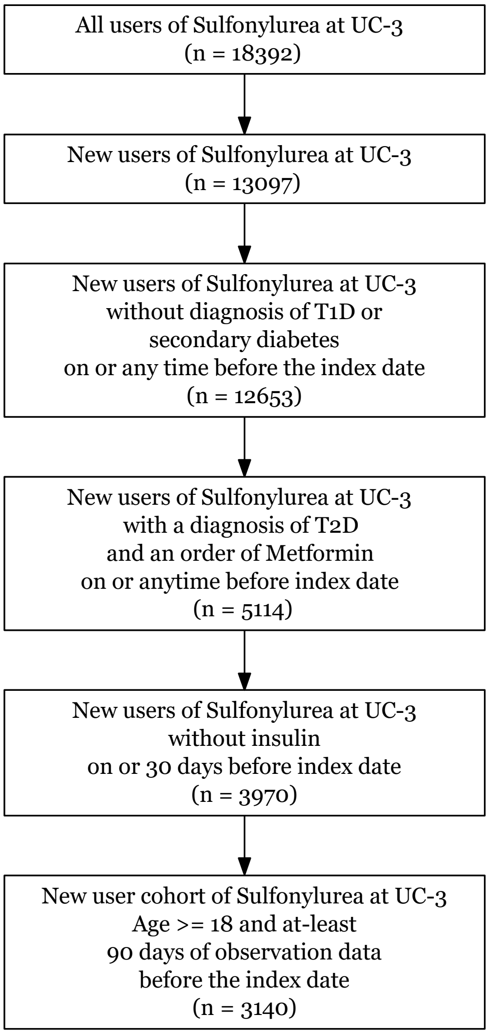

eFigure 4: Sulfonylurea cohort UC-3

© 2023 Vashisht R et al. *JAMA Network Open.*

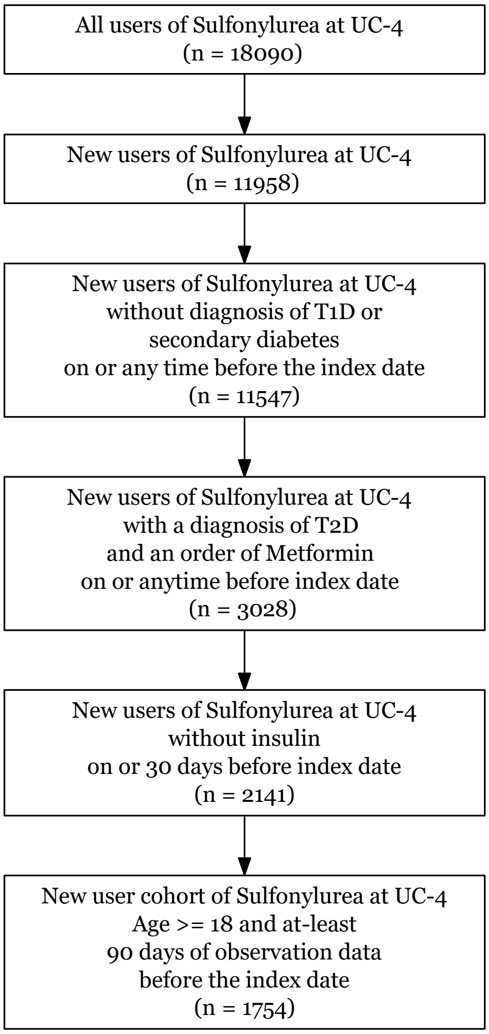

eFigure 5: Sulfonylurea cohort UC-4

© 2023 Vashisht R et al. *JAMA Network Open.*

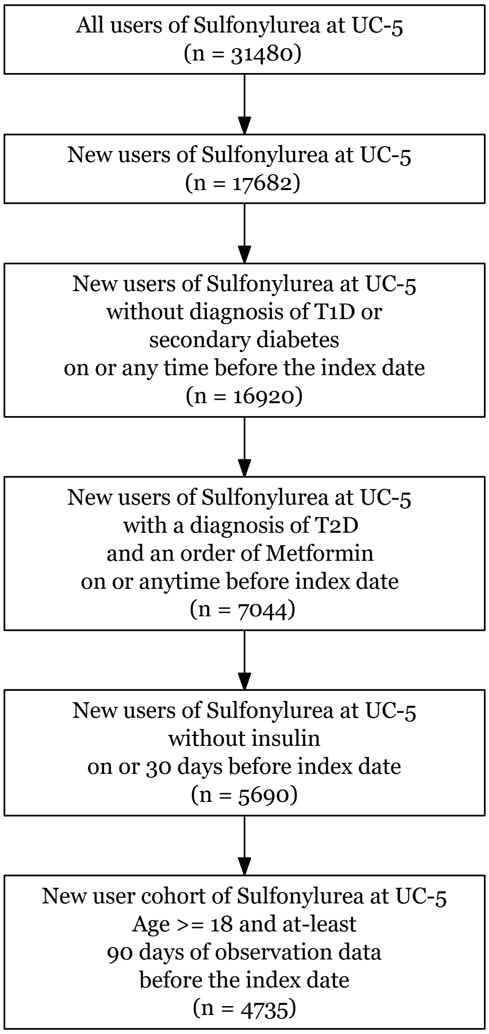

eFigure 6: Sulfonylurea cohort UC-5

© 2023 Vashisht R et al. *JAMA Network Open.*

1.8 eFigure: Cohort selection DPP4-Inhibitors

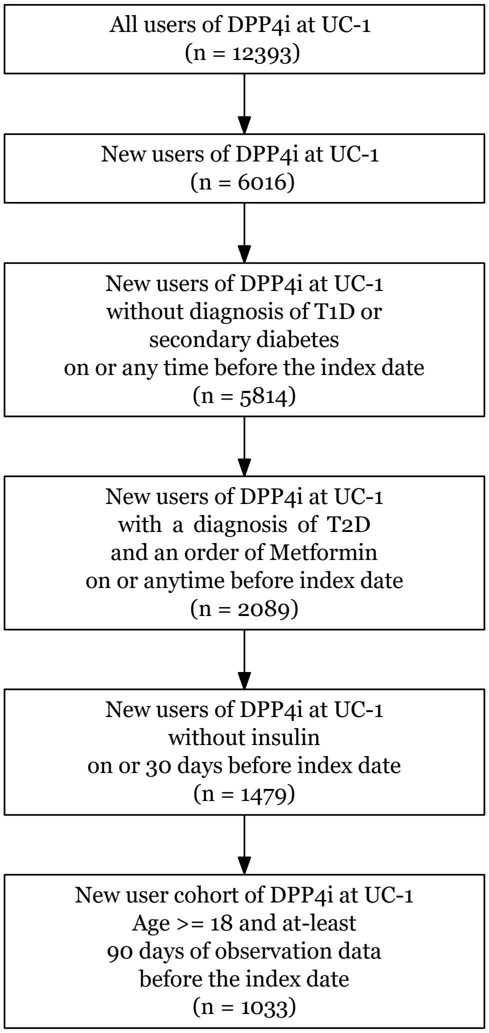

eFigure 7: DPP4i cohort UC-1

© 2023 Vashisht R et al. *JAMA Network Open.*

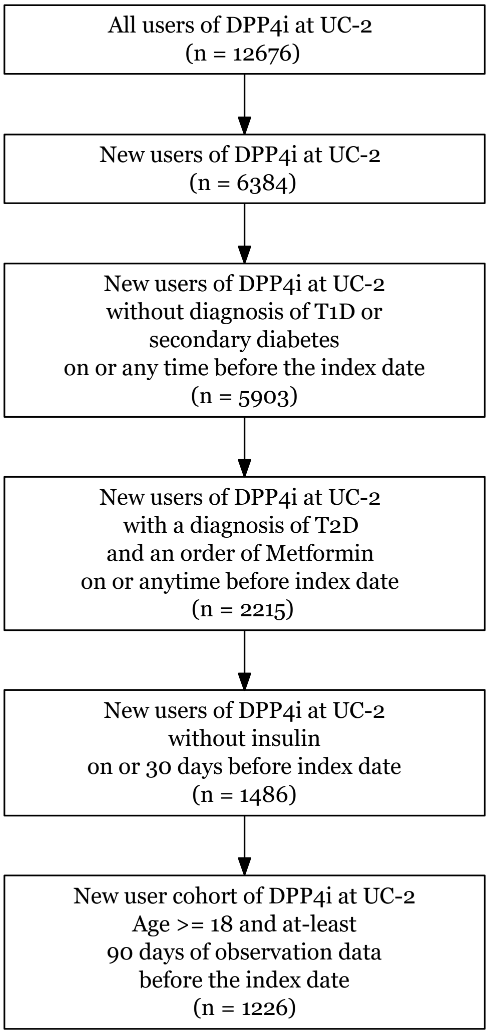

eFigure 8: DPP4i cohort UC-2

© 2023 Vashisht R et al. *JAMA Network Open.*

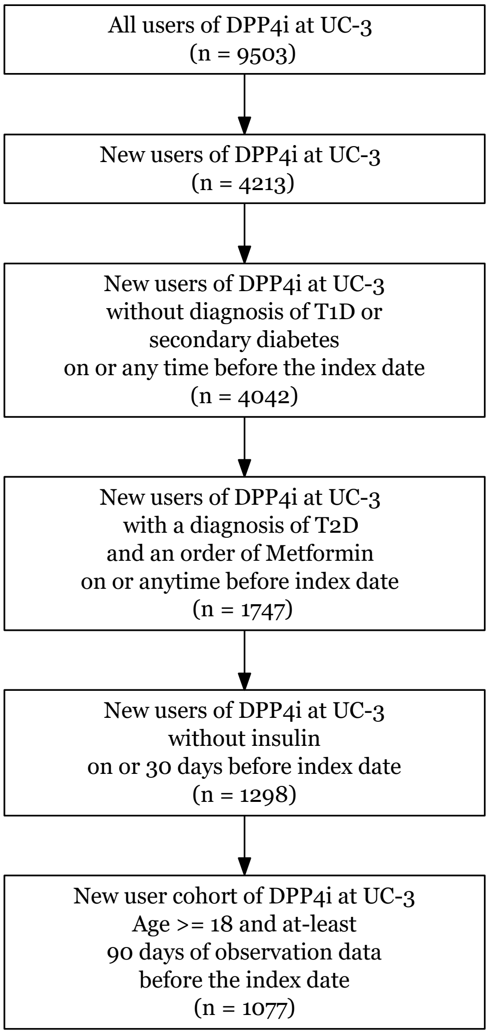

eFigure 9: DPP4i cohort UC-3

© 2023 Vashisht R et al. *JAMA Network Open.*

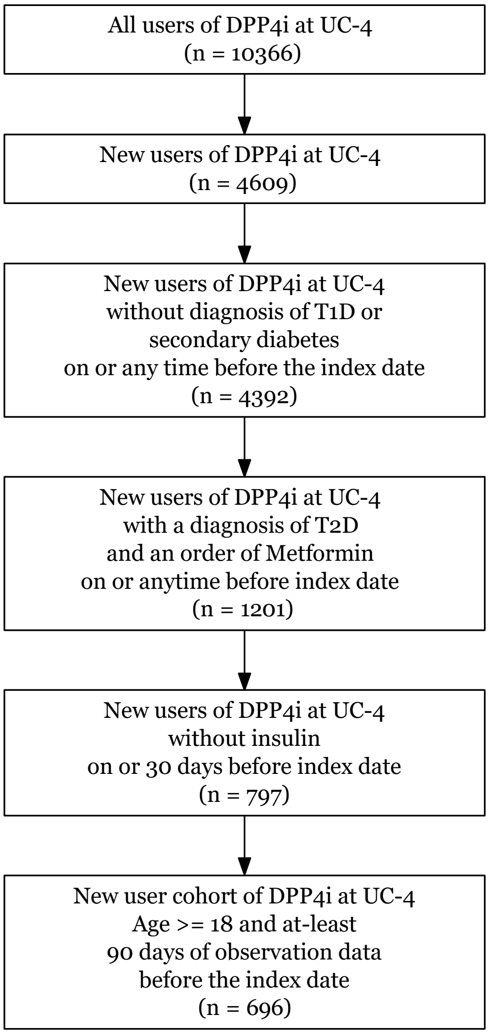

eFigure 10: DPP4i cohort UC-4

© 2023 Vashisht R et al. *JAMA Network Open.*

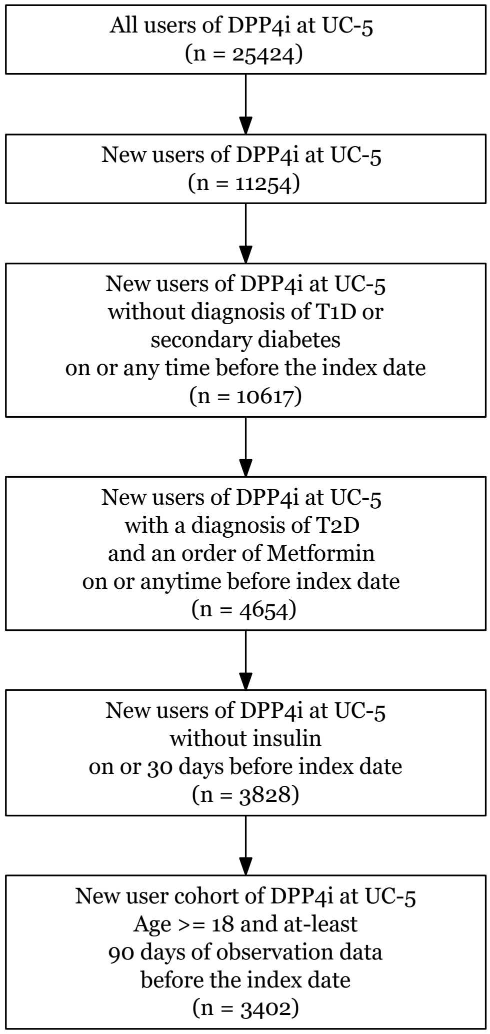

eFigure 11: DPP4i cohort UC-5

© 2023 Vashisht R et al. *JAMA Network Open.*

1.9 eFigure: Cohort selection SGLT2-Inhibitors

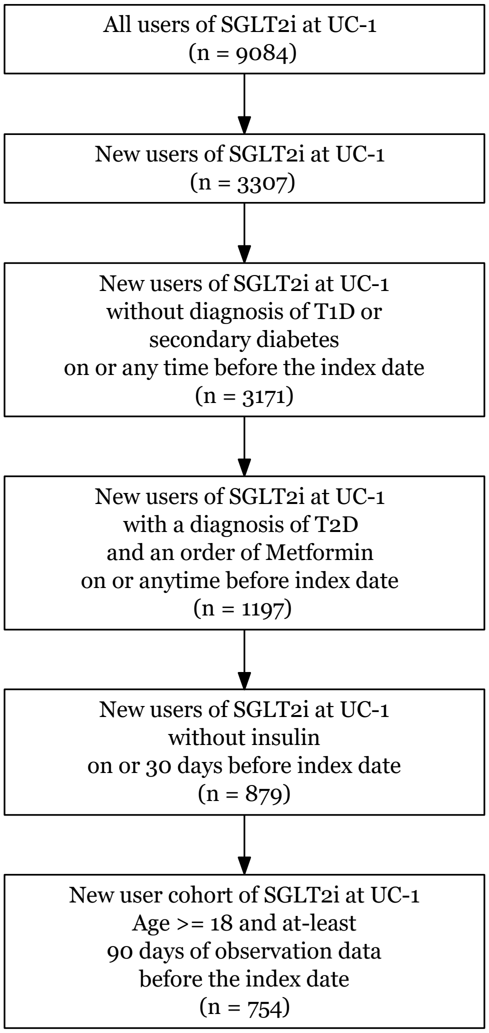

eFigure 12: SGLT2i cohort UC-1

© 2023 Vashisht R et al. *JAMA Network Open.*

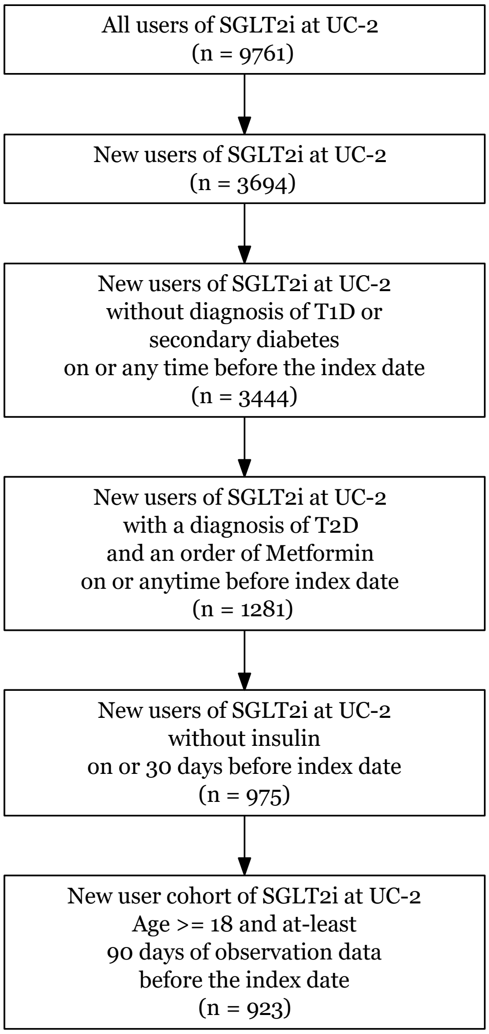

eFigure 13: SGLT2i cohort UC-2

© 2023 Vashisht R et al. *JAMA Network Open.*

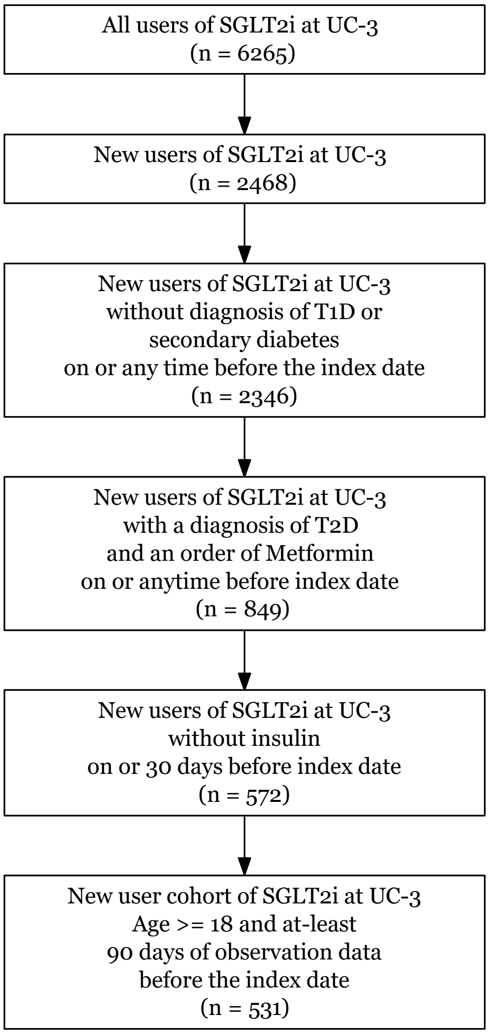

eFigure 14: SGLT2i cohort UC-3

© 2023 Vashisht R et al. *JAMA Network Open.*

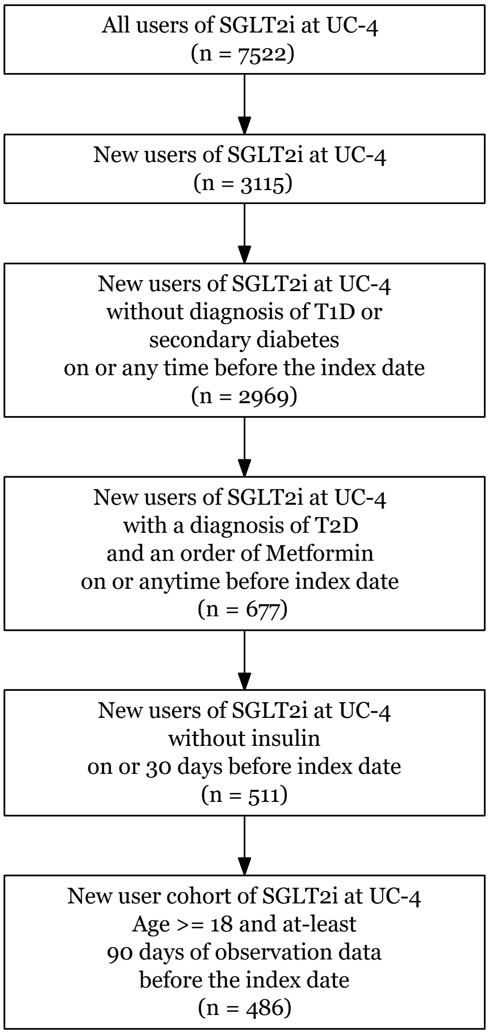

eFigure 15: SGLT2i cohort UC-4

© 2023 Vashisht R et al. *JAMA Network Open.*

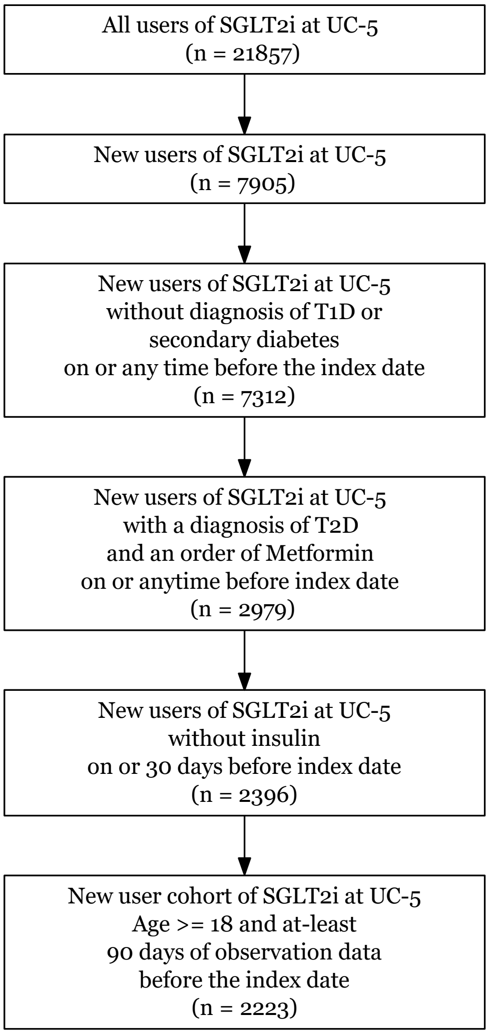

eFigure 16: SGLT2i cohort UC-5

© 2023 Vashisht R et al. *JAMA Network Open.*

1.10 eFigure: Cohort selection Glucagon-Like Peptide 1 Receptor Agonist

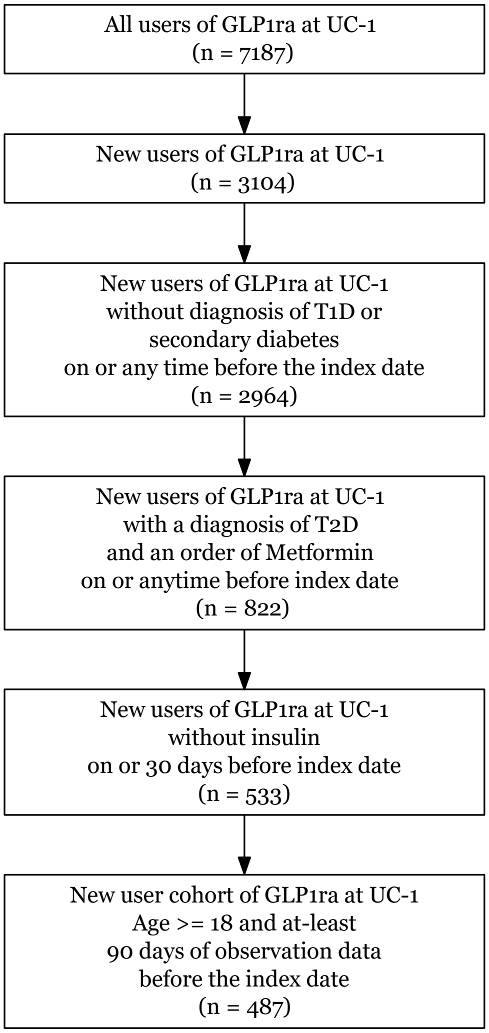

eFigure 17: GLP1ra cohort UC-1

© 2023 Vashisht R et al. *JAMA Network Open.*

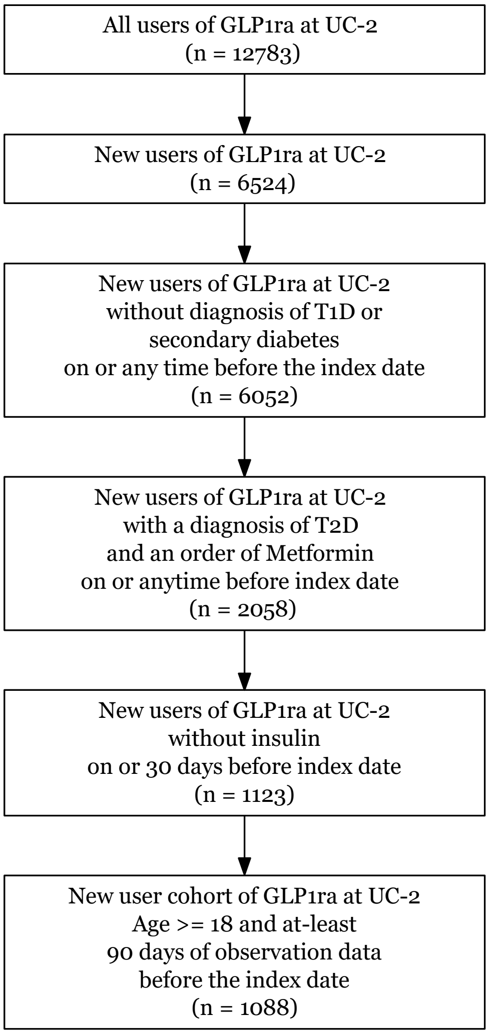

eFigure 18: GLP1ra cohort UC-2

© 2023 Vashisht R et al. *JAMA Network Open.*

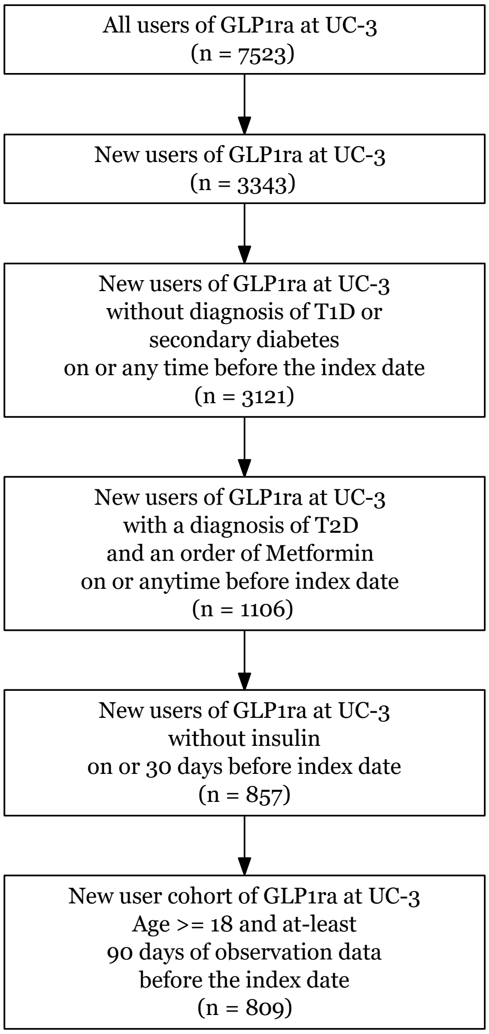

eFigure 19: GLP1ra cohort UC-3

© 2023 Vashisht R et al. *JAMA Network Open.*

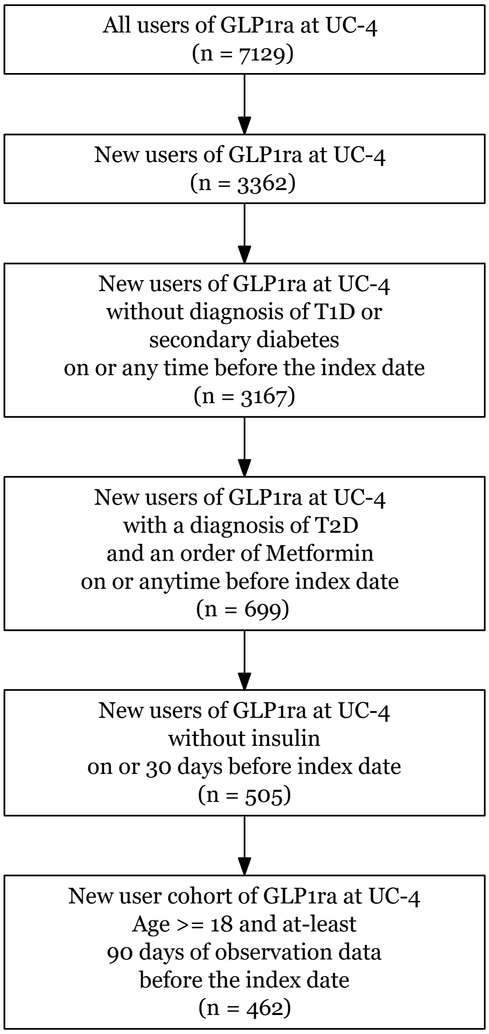

eFigure 20: GLP1ra cohort UC-4

© 2023 Vashisht R et al. *JAMA Network Open.*

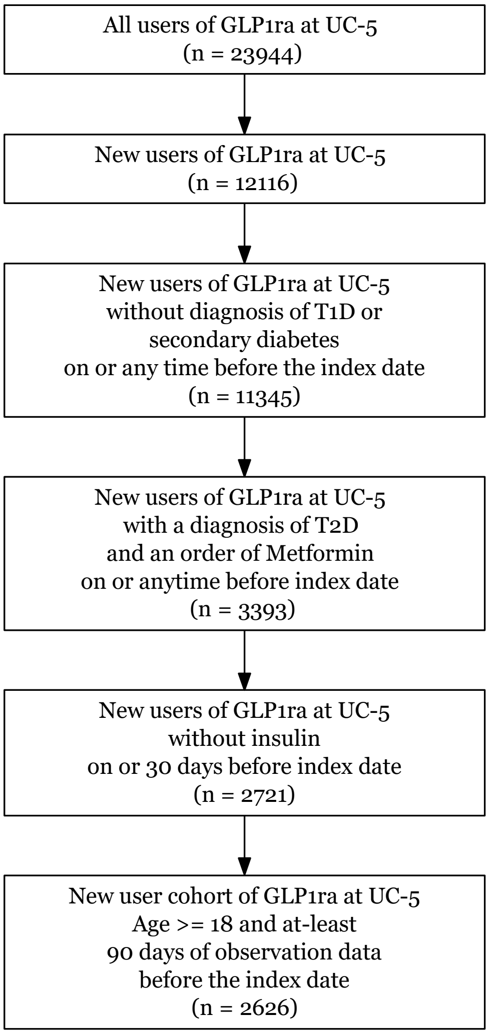

eFigure 21: GLP1ra cohort UC-5

© 2023 Vashisht R et al. *JAMA Network Open.*

eAppendix 2. Patient Matching

The following section illustrate a table and figures. The table summarize the total number of patients before and after matching in each comparator (C) and treatment (T) cohort at each UC health site. The figures illustrate the propensity score distribution before and after matching as well as covariate balance before and after matching for each comparator and treatment cohort at each UC health site. The covariates are segregated into conditions, medication, measurement and procedures for easy visualization and to also represent the total number of covariates in each category that were used to match patients across UC health.

1.11 eTable: Patient Matching Summary Table

eTable 1: Total number of patients in a given comparator and treat- ment cohort before and after propensity score matching at a given UC Health site are tabulated below. The number of clinical Covari- ates including drugs, disease, laboratory measurement and Medical procedures used in matching and the percentage of covariates that achieved balance after matching are tabulated. The covariates with absolute standardize mean difference < 0.10 after matching were considered balanced.

	Number of patients before Matching		Number of patients after Matching			
UC	Comparator (n)	Treatment (n)	Comparator (n)	Treatment (n)	Number of Covariates	Balanced Covariates After Matching
1	DPP4i (1033)	GLP1ra (487)	DPP4i (356)	GLP1ra (356)	5539	99.96
1	DPP4i (1033)	SGLT2i (754)	DPP4i (537)	SGLT2i (537)	5802	99.98
1	GLP1ra (487)	SGLT2i (754)	GLP1ra (362)	SGLT2i (362)	4751	99.92
1	Sulfonylurea (2409)	DPP4i (1033)	Sulfonylurea (878)	DPP4i (878)	4429	100.00
1	Sulfonylurea (2409)	GLP1ra (487)	Sulfonylurea (376)	GLP1ra (376)	4711	99.28
1	Sulfonylurea (2409)	SGLT2i (754)	Sulfonylurea (612)	SGLT2i (612)	4107	99.90
2	DPP4i (1226)	GLP1ra (1088)	DPP4i (549)	GLP1ra (549)	5091	99.88
2	DPP4i (1226)	SGLT2i (923)	DPP4i (544)	SGLT2i (544)	4801	99.94
2	GLP1ra (1088)	SGLT2i (923)	GLP1ra (658)	SGLT2i (658)	4953	100.00
2	Sulfonylurea (1991)	DPP4i (1226)	Sulfonylurea (1036)	DPP4i (1036)	4485	100.00
2	Sulfonylurea (1991)	GLP1ra (1088)	Sulfonylurea (587)	GLP1ra (587)	4679	99.96
2	Sulfonylurea (1991)	SGLT2i (923)	Sulfonylurea (571)	SGLT2i (571)	5220	99.96
3	DPP4i (1077)	GLP1ra (809)	DPP4i (439)	GLP1ra (439)	5087	99.98
3	DPP4i (1077)	SGLT2i (531)	DPP4i (360)	SGLT2i (360)	4655	99.23
3	GLP1ra (809)	SGLT2i (531)	GLP1ra (417)	SGLT2i (417)	4520	99.91
3	Sulfonylurea (3140)	DPP4i (1077)	Sulfonylurea (1071)	DPP4i (1071)	3803	99.97
3	Sulfonylurea (3140)	GLP1ra (809)	Sulfonylurea (549)	GLP1ra (549)	4322	98.38
3	Sulfonylurea (3140)	SGLT2i (531)	Sulfonylurea (495)	SGLT2i (495)	4119	97.40
4	DPP4i (696)	GLP1ra (462)	DPP4i (279)	GLP1ra (279)	4627	99.72
4	DPP4i (696)	SGLT2i (486)	DPP4i (284)	SGLT2i (284)	4658	99.61
4	GLP1ra (462)	SGLT2i (486)	GLP1ra (332)	SGLT2i (332)	7108	99.94
4	Sulfonylurea (1754)	DPP4i (696)	Sulfonylurea (642)	DPP4i (642)	4751	99.79
4	Sulfonylurea (1754)	GLP1ra (462)	Sulfonylurea (332)	GLP1ra (332)	4585	98.69

© 2023 Vashisht R et al. *JAMA Network Open.*

eTable 1: Total number of patients in a given comparator and treatme *(continued)*

UC	Comparator (n)	Treatment (n)	Comparator (n)	Treatment (n)	Number of Co-variates	Balanced Covari-ates After Match-ing
4	Sulfonylurea (1754)	SGLT2i (486)	Sulfonylurea (386)	SGLT2i (386)	4659	98.99
5	DPP4i (3402)	GLP1ra (2626)	DPP4i (1361)	GLP1ra (1361)	4201	100.00
5	DPP4i (3402)	SGLT2i (2223)	DPP4i (1516)	SGLT2i (1516)	4299	99.88
5	GLP1ra (2626)	SGLT2i (2223)	GLP1ra (1570)	SGLT2i (1570)	4609	100.00
5	Sulfonylurea (4735)	DPP4i (3402)	Sulfonylurea (2908)	DPP4i (2908)	3881	99.92
5	Sulfonylurea (4735)	GLP1ra (2626)	Sulfonylurea (1315)	GLP1ra (1315)	4057	100.00
5	Sulfonylurea (4735)	SGLT2i (2223)	Sulfonylurea (1501)	SGLT2i (1501)	4137	99.81

© 2023 Vashisht R et al. *JAMA Network Open.*

1.12 Patient Matching Visualization

Each figure illustrates the propensity score matching of the patients in the treatment cohort to the patients in the comparator cohort along with covariate balance diagnostics. The probability distribution of estimated propensity scores before and after matching is shown (A, B) along with the covariate balance before and after matching (C). The covariates with absolute standardize mean difference < 0.10 after matching are considered balanced. The number of patients in comparator and treatment groups before and after matching are provided. In addition, the balance diagnostic plot illustrates the overall percentage of covariate balance achieved after matching. The balance diagnostic plot also highlights the number of covariates segregated into procedures (medical procedures), measurements (laboratory measurements), medication and condition (medical condition diagnosis) before the index date that were used in propensity score estimation. The drug class abbreviations are DPP4i (Dipeptidyl Peptidase-4 Inhibitor), GLP1ra (Glucagon-like Peptide-1 Receptor Agonists), SGLT2i (Sodium-Glucose Cotransporter-2 Inhibitor). Figures are plotted by the comparator and treatment combination per UC health site similar to that in eTable-1 above.

1.12.1 eFigure: DPP4i (C) and GLP1ra (T) at UC-1

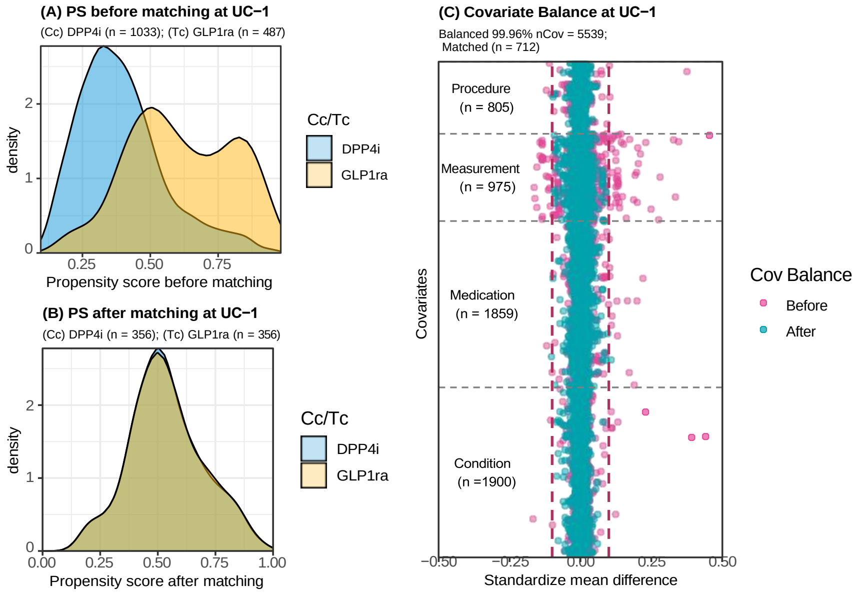

eFigure 22: Propensity score matching DPP4i (C) and GLP1ra (T) along with covariate balance plot at UC-1

1.12.2 eFigure: DPP4i (C) and SGLT2i (T) at UC-1

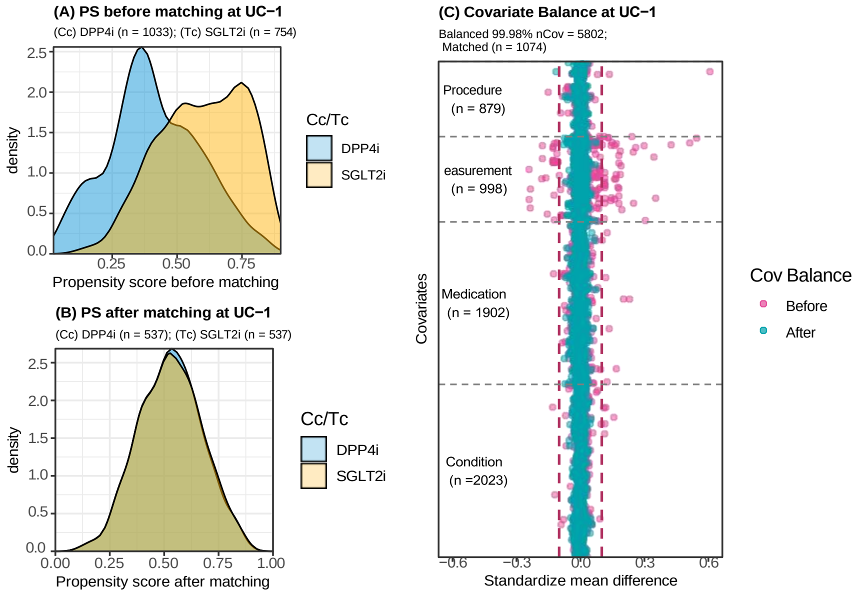

eFigure 23: Propensity score matching DPP4i (C) and SGLT2i (T) along with covariate balance plot at UC-1

© 2023 Vashisht R et al. *JAMA Network Open.*

1.12.3 eFigure: GLP1ra (C) and SGLT2i (T) at UC-1

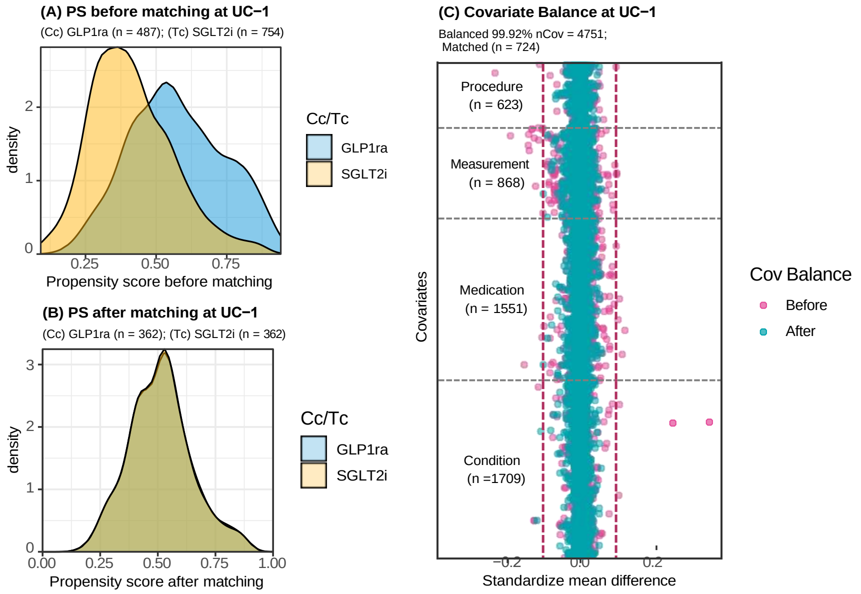

eFigure 24: Propensity score matching GLP1ra (C) and SGLT2i (T) along with covariate balance plot at UC-1

1.12.4 eFigure: Sulfonylurea (C) and DPP4i (T) at UC-1

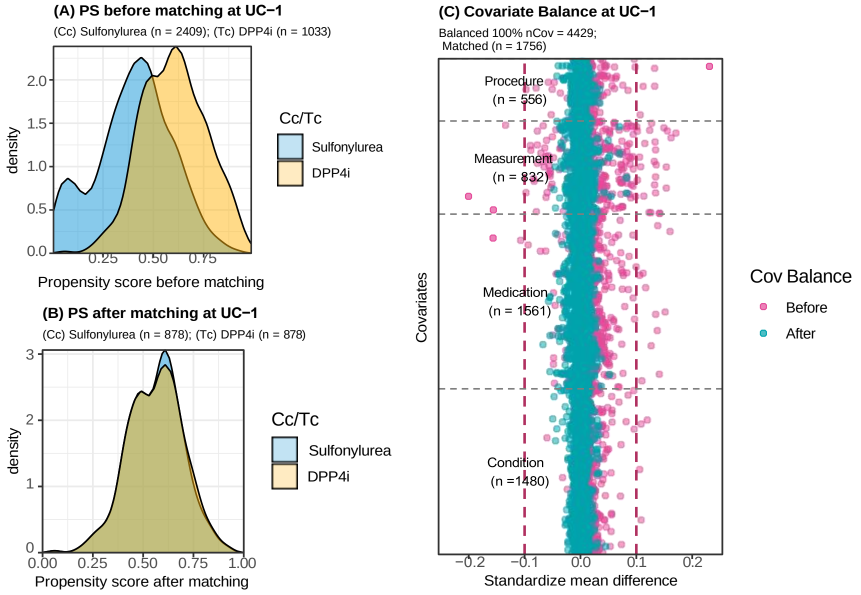

eFigure 25: Propensity score matching Sulfonylurea (C) and DPP4i (T) along with covariate balance plot at UC-1

© 2023 Vashisht R et al. *JAMA Network Open.*

1.12.5 eFigure: Sulfonylurea (C) and SGLT2i (T) at UC-1

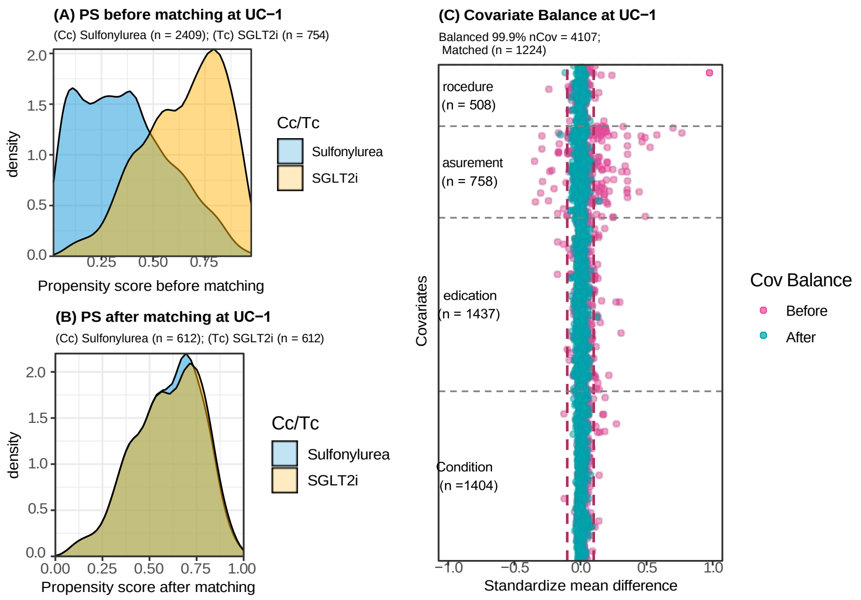

eFigure 26: Propensity score matching Sulfonylurea (C) and SGLT2i (T) along with covariate balance plot at UC-1

© 2023 Vashisht R et al. *JAMA Network Open.*

1.12.6 eFigure: DPP4i (C) and GLP1ra (T) at UC-2

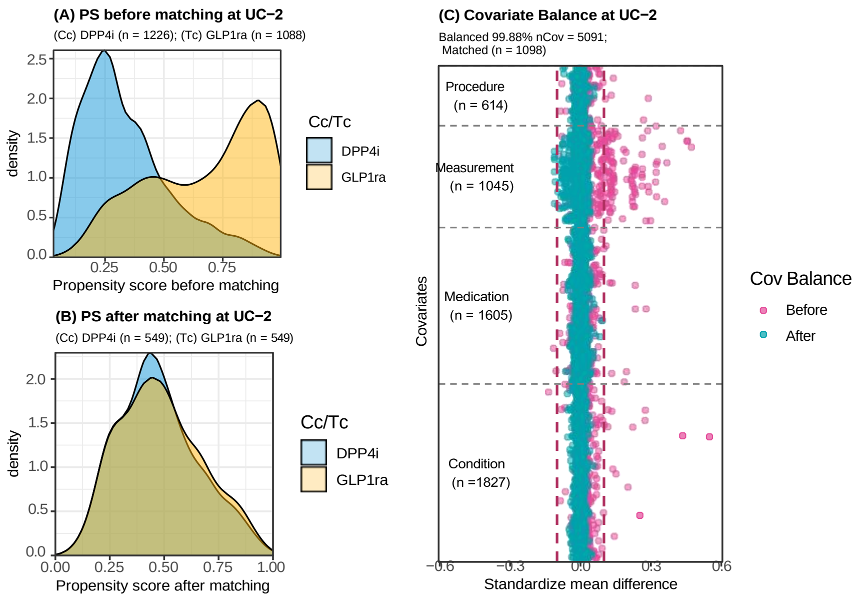

eFigure 27: Propensity score matching DPP4i (C) and GLP1ra (T) along with covariate balance plot at UC-2

1.12.7 eFigure: DPP4i (C) and SGLT2i (T) at UC-2

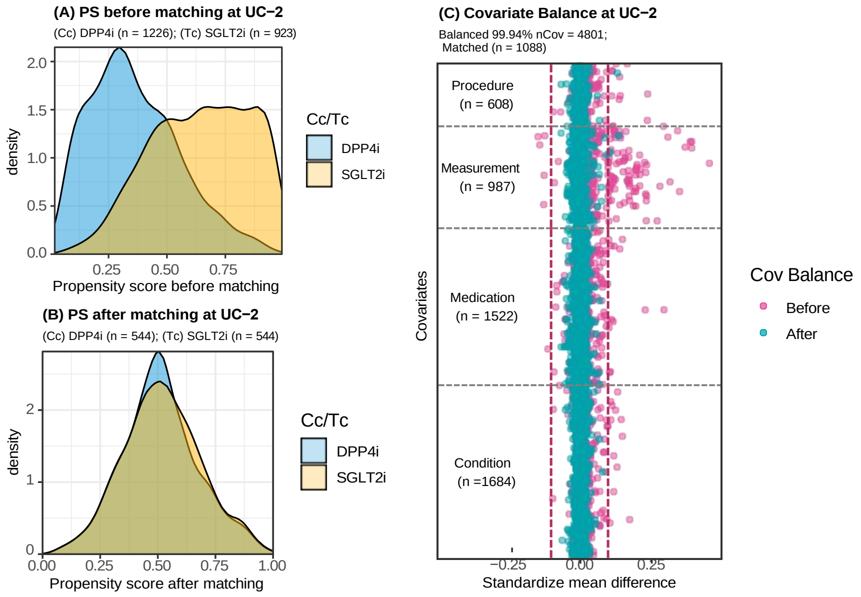

eFigure 28: Propensity score matching DPP4i (C) and SGLT2i (T) along with covariate balance plot at UC-2

© 2023 Vashisht R et al. *JAMA Network Open.*

1.12.8 eFigure: GLP1ra (C) and SGLT2i (T) at UC-2

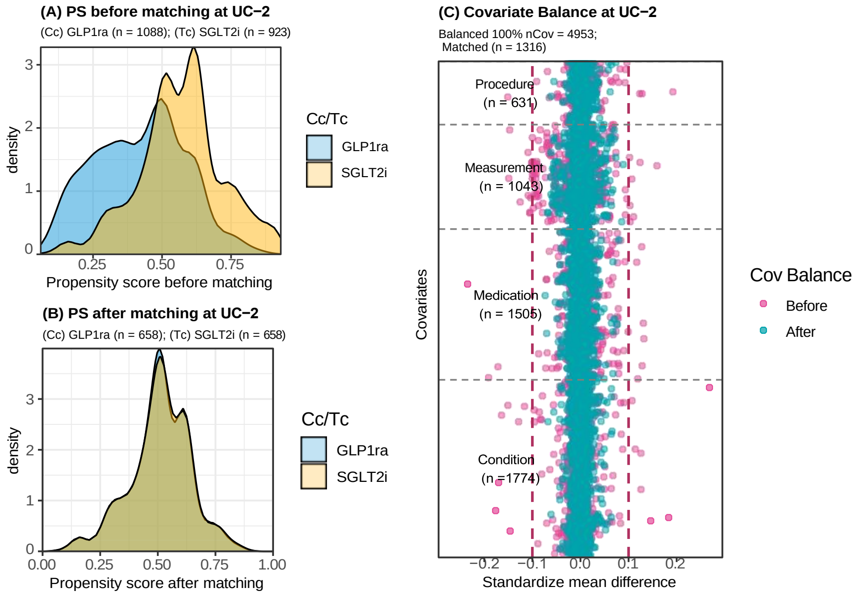

eFigure 29: Propensity score matching GLP1ra (C) and SGLT2i (T) along with covariate balance plot at UC-2

1.12.9 eFigure: Sulfonylurea (C) and DPP4i (T) at UC-2

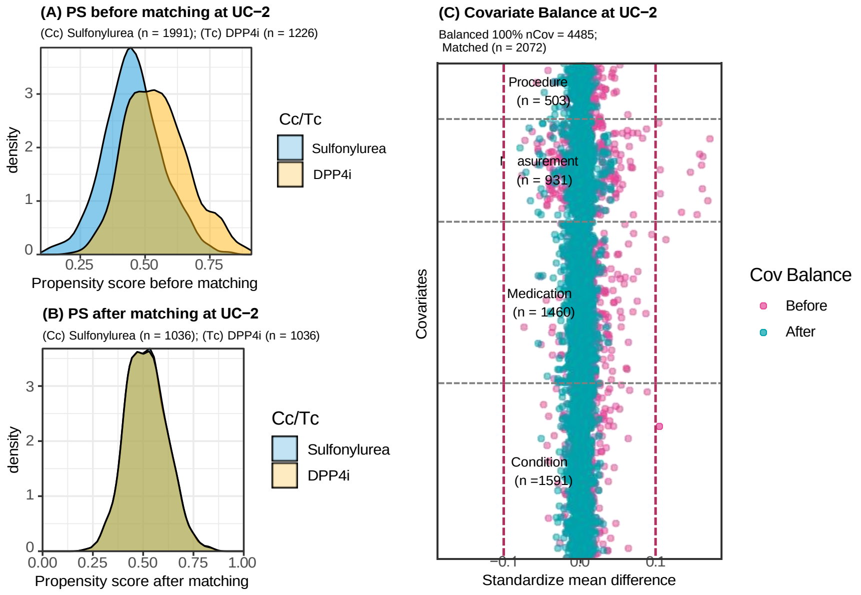

eFigure 30: Propensity score matching Sulfonylurea (C) and DPP4i (T) along with covariate balance plot at UC-2

© 2023 Vashisht R et al. *JAMA Network Open.*

1.12.10 eFigure: Sulfonylurea (C) and SGLT2i (T) at UC-2

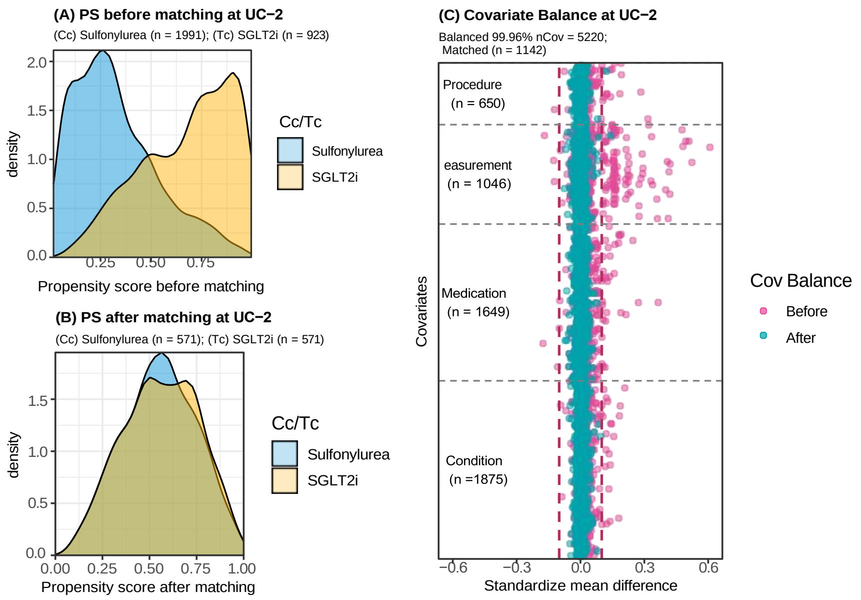

eFigure 31: Propensity score matching Sulfonylurea (C) and SGLT2i (T) along with covariate balance plot at UC-2

© 2023 Vashisht R et al. *JAMA Network Open.*

1.12.11 eFigure: DPP4i (C) and GLP1ra (T) at UC-3

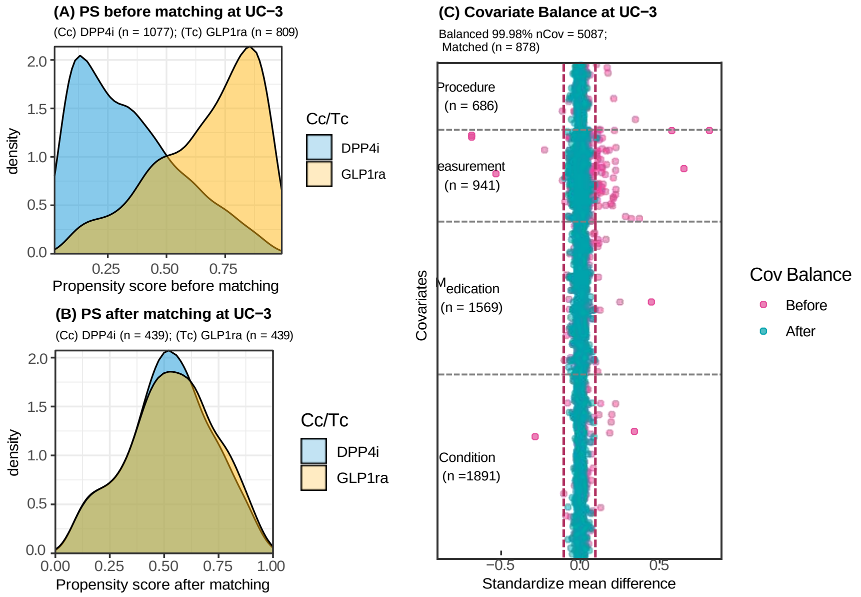

eFigure 32: Propensity score matching DPP4i (C) and GLP1ra (T) along with covariate balance plot at UC-3

1.12.12 eFigure: DPP4i (C) and SGLT2i (T) at UC-3

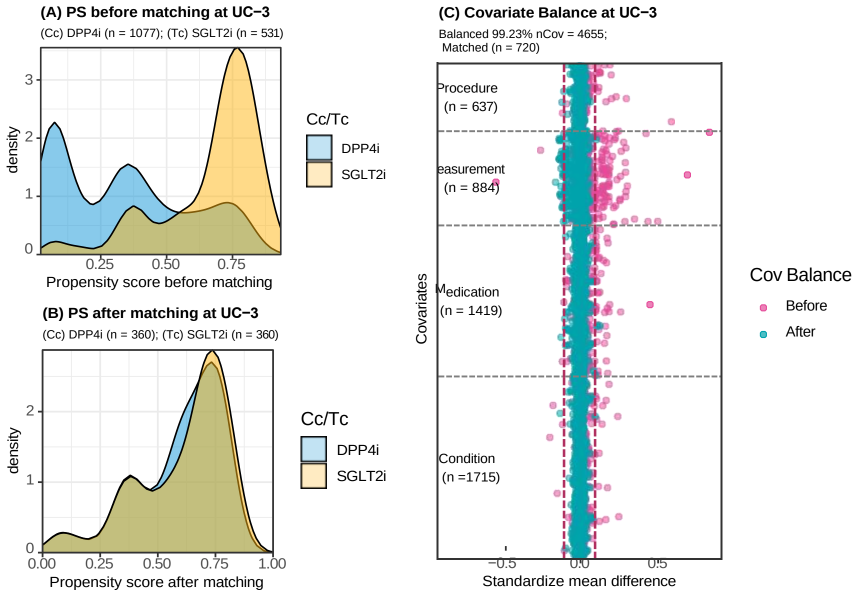

eFigure 33: Propensity score matching DPP4i (C) and SGLT2i (T) along with covariate balance plot at UC-3

© 2023 Vashisht R et al. *JAMA Network Open.*

1.12.13 eFigure: GLP1ra (C) and SGLT2i (T) at UC-3

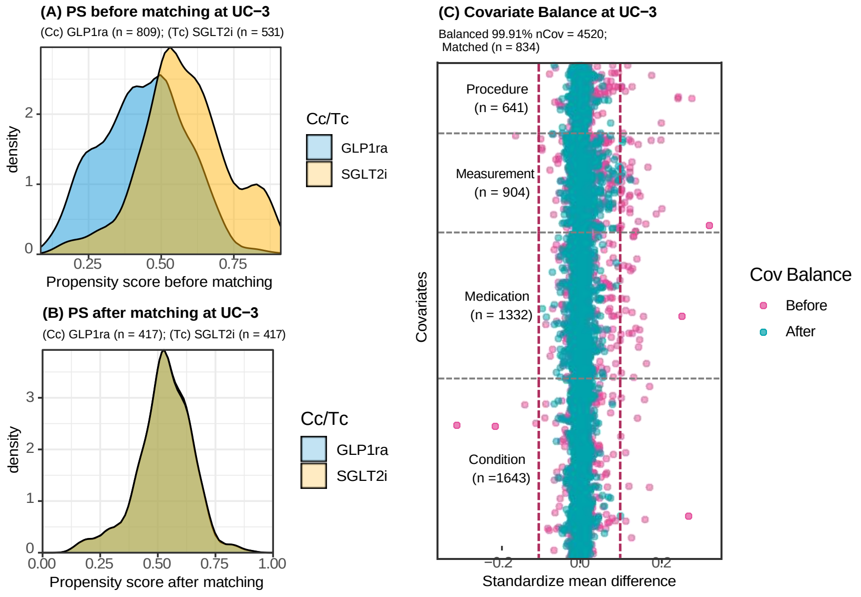

eFigure 34: Propensity score matching GLP1ra (C) and SGLT2i (T) along with covariate balance plot at UC-3

1.12.14 eFigure: Sulfonylurea (C) and DPP4i (T) at UC-3

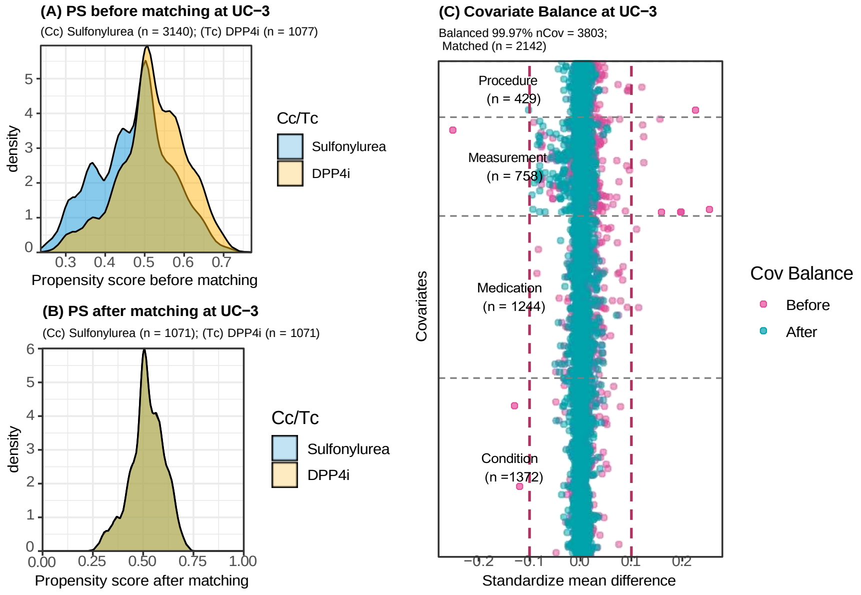

eFigure 35: Propensity score matching Sulfonylurea (C) and DPP4i (T) along with covariate balance plot at UC-3

1.12.15 eFigure: Sulfonylurea (C) and SGLT2i (T) at UC-3

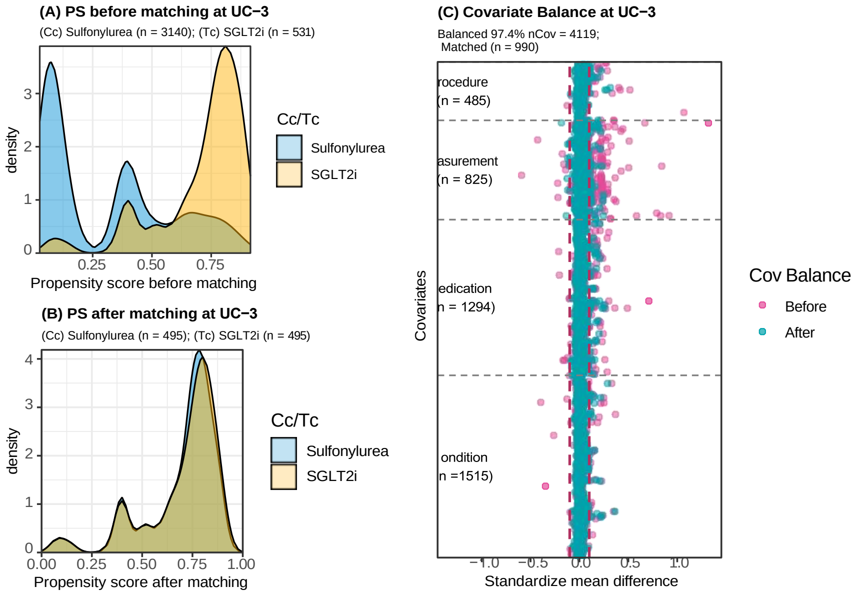

eFigure 36: Propensity score matching Sulfonylurea (C) and SGLT2i (T) along with covariate balance plot at UC-3

© 2023 Vashisht R et al. *JAMA Network Open.*

1.12.16 eFigure: DPP4i (C) and GLP1ra (T) at UC-4

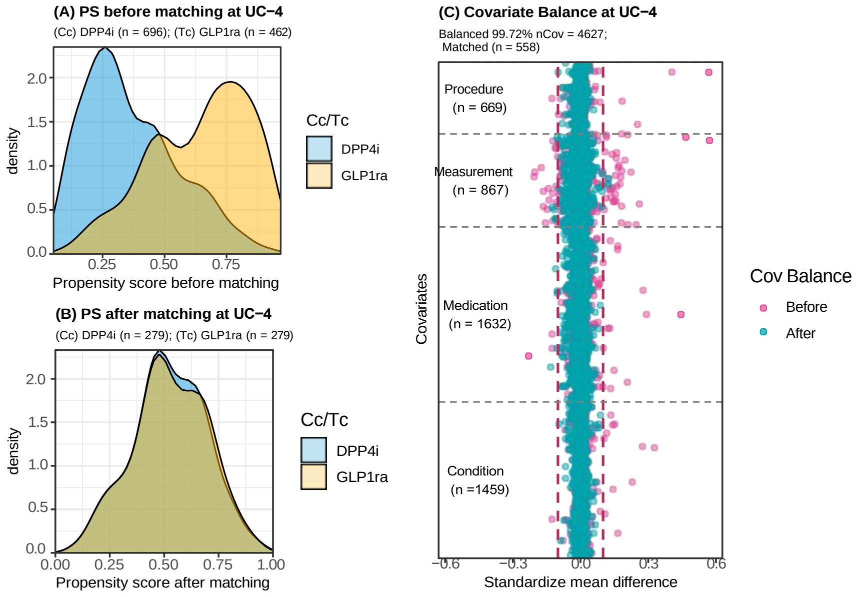

eFigure 37: Propensity score matching DPP4i (C) and GLP1ra (T) along with covariate balance plot at UC-4

1.12.17 eFigure: DPP4i (C) and SGLT2i (T) at UC-4

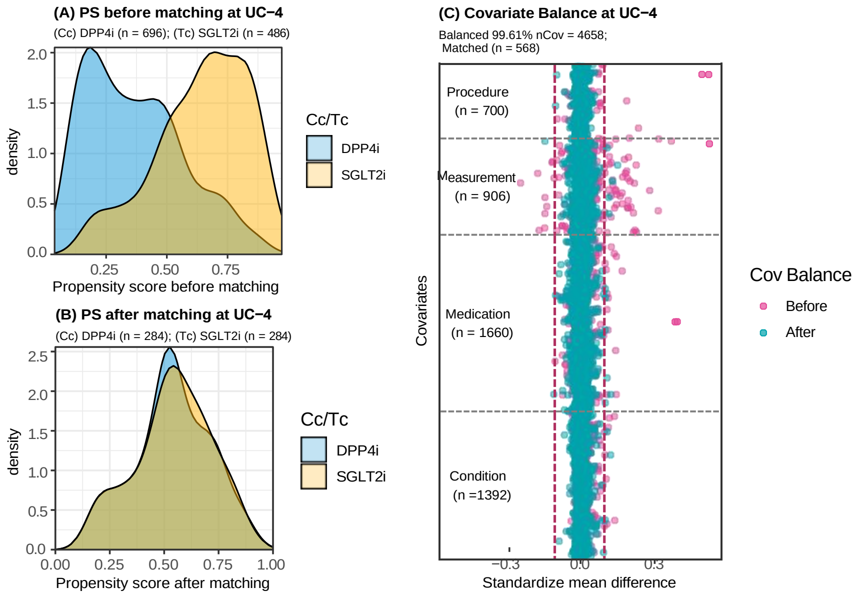

eFigure 38: Propensity score matching DPP4i (C) and SGLT2i (T) along with covariate balance plot at UC-4

© 2023 Vashisht R et al. *JAMA Network Open.*

1.12.18 eFigure: GLP1ra (C) and SGLT2i (T) at UC-4

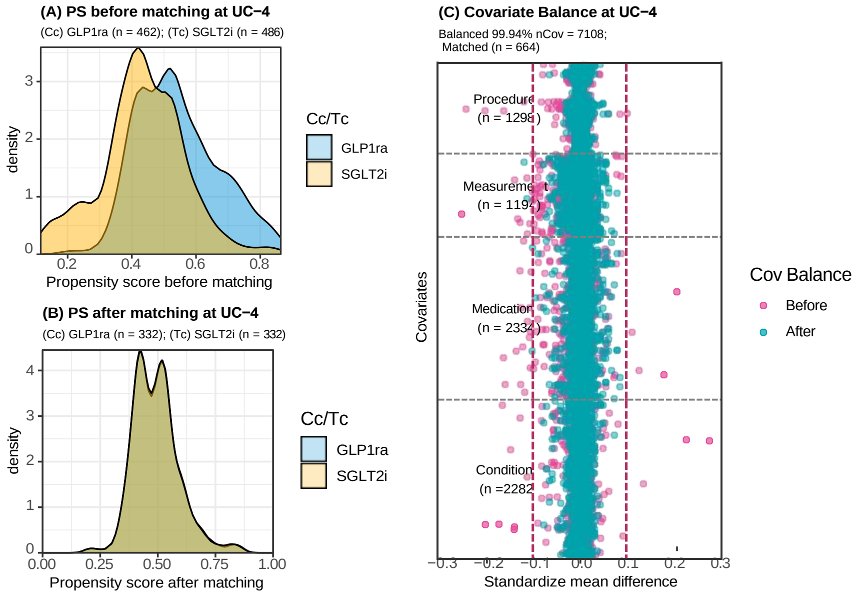

eFigure 39: Propensity score matching GLP1ra (C) and SGLT2i (T) along with covariate balance plot at UC-4

1.12.19 eFigure: Sulfonylurea (C) and DPP4i (T) at UC-4

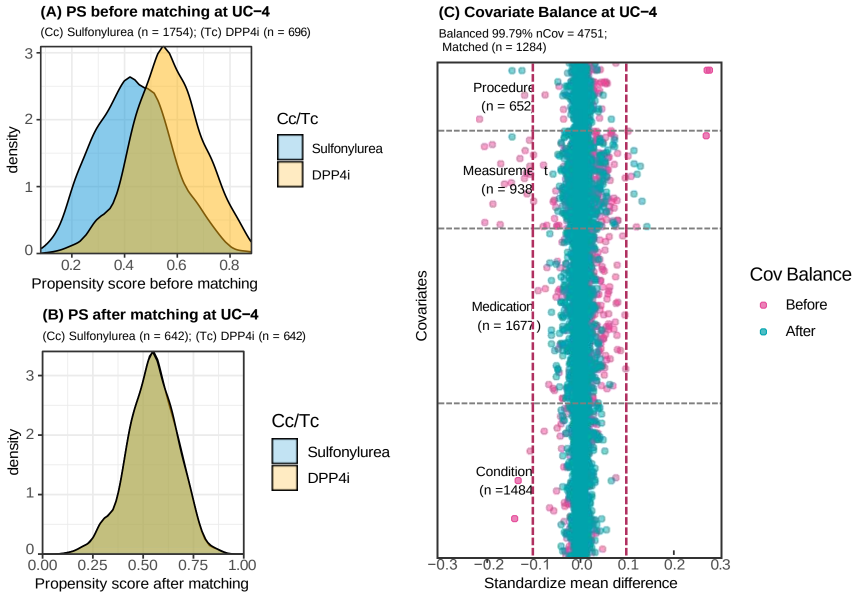

eFigure 40: Propensity score matching Sulfonylurea (C) and DPP4i (T) along with covariate balance plot at UC-4

© 2023 Vashisht R et al. *JAMA Network Open.*

1.12.20 eFigure: Sulfonylurea (C) and SGLT2i (T) at UC-4

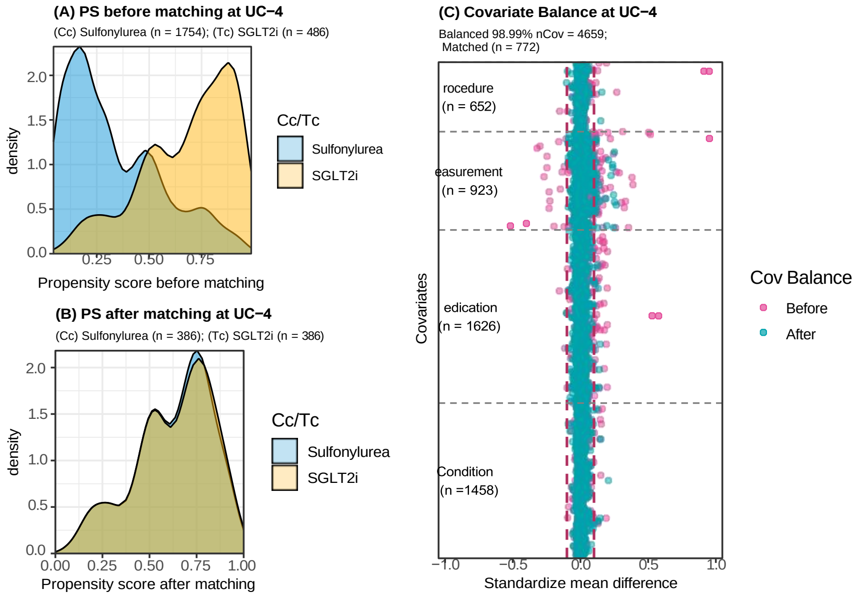

eFigure 41: Propensity score matching Sulfonylurea (C) and SGLT2i (T) along with covariate balance plot at UC-4

© 2023 Vashisht R et al. *JAMA Network Open.*

1.12.21 eFigure: DPP4i (C) and GLP1ra (T) at UC-5

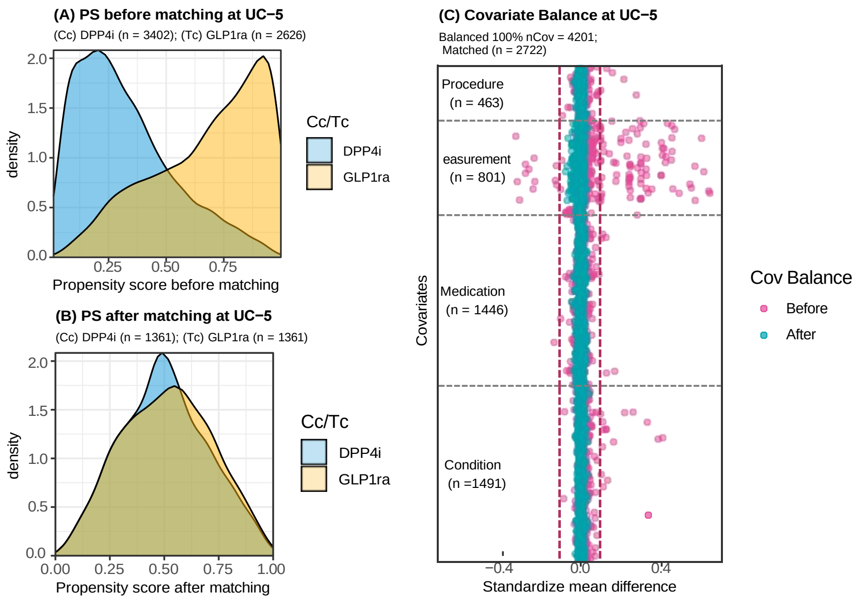

eFigure 42: Propensity score matching DPP4i (C) and GLP1ra (T) along with covariate balance plot at UC-5

1.12.22 eFigure: DPP4i (C) and SGLT2i (T) at UC-5

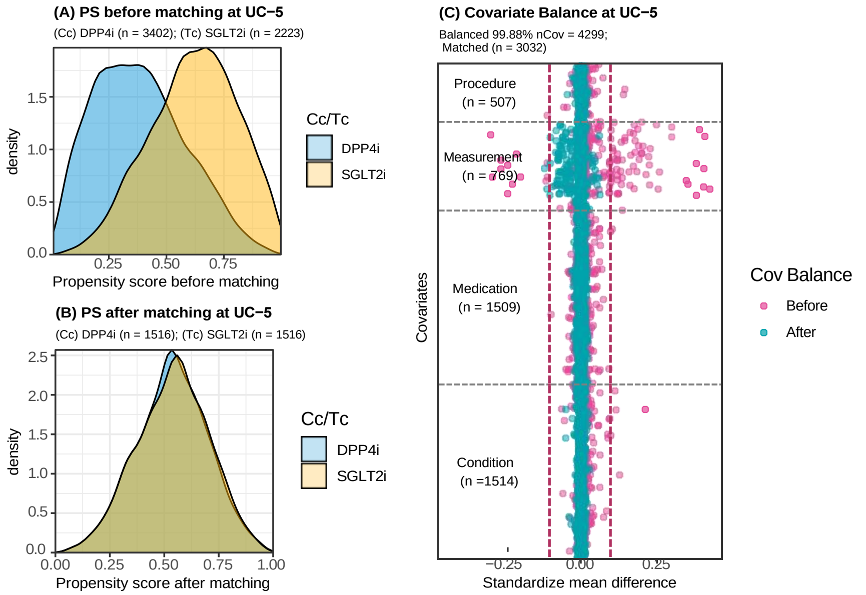

eFigure 43: Propensity score matching DPP4i (C) and SGLT2i (T) along with covariate balance plot at UC-5

© 2023 Vashisht R et al. *JAMA Network Open.*

1.12.23 eFigure: GLP1ra (C) and SGLT2i (T) at UC-5

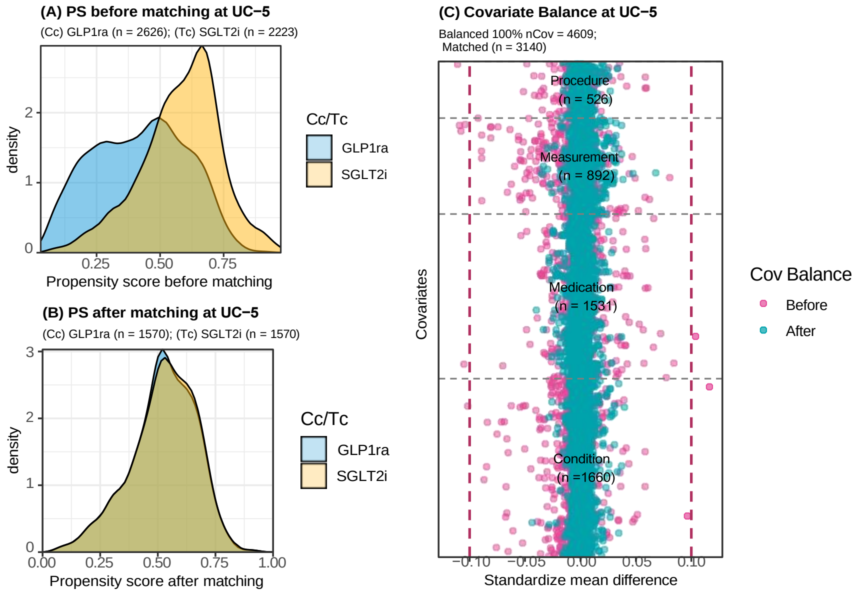

eFigure 44: Propensity score matching GLP1ra (C) and SGLT2i (T) along with covariate balance plot at UC-5

1.12.24 eFigure: Sulfonylurea (C) and DPP4i (T) at UC-5

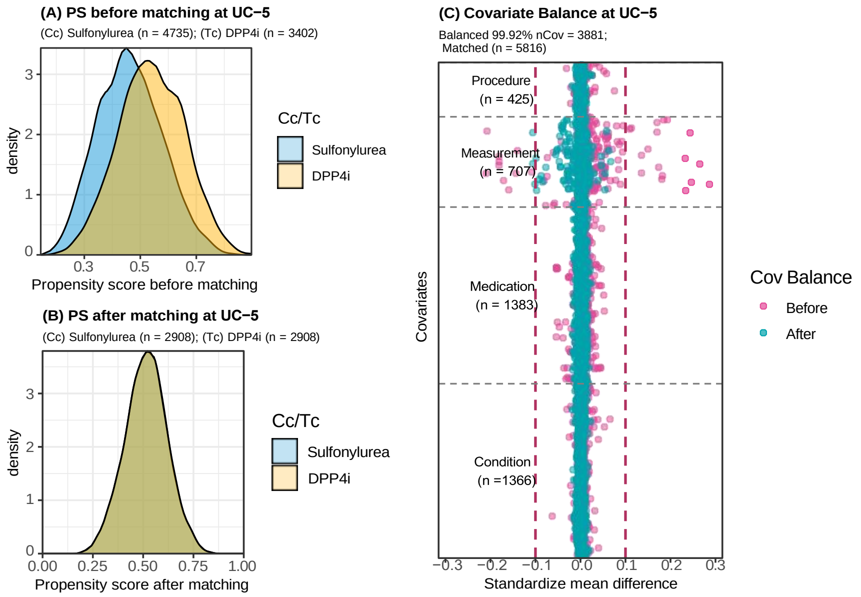

eFigure 45: Propensity score matching Sulfonylurea (C) and DPP4i (T) along with covariate balance plot at UC-5

© 2023 Vashisht R et al. *JAMA Network Open.*

1.12.25 eFigure: Sulfonylurea (C) and SGLT2i (T) at UC-5

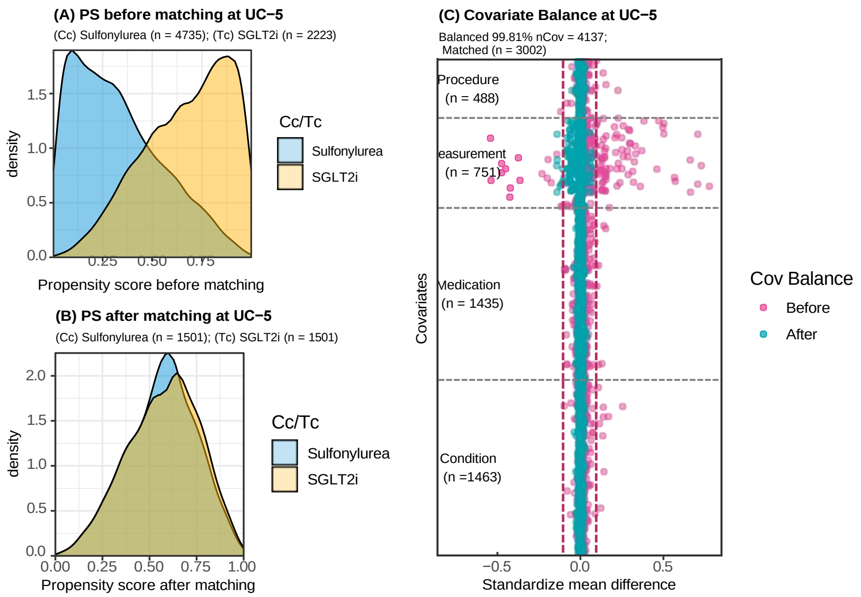

eFigure 46: Propensity score matching Sulfonylurea (C) and SGLT2i (T) along with covariate balance plot at UC-5

© 2023 Vashisht R et al. *JAMA Network Open.*

eAppendix 3. Comparative Effectiveness and Safety

The comparative effectiveness and safety of each drug combination at each of the UC is illustrated. The comparative effectiveness was assessed in terms of time to metabolic failure (HbA1c >= 7%) after treatment initiation. The safety was assessed in term of new incidence of 29 adverse outcomes. For each outcome, the effect size of each drug comparison at each UC is tabulated. The effect size and the summary statistics are visualized using forest plots. The summary estimates were obtained using a random-effect meta-analysis approach where each drug-pair was analyzed across all the 5 UC health sites. Additionally, the leave-one-UC-out sensitivity analysis of each drug pair is tabulated.

4.1 Metabolic Failure HbA1c >= 7%

4.1.1 eTable: Drug Comparison eTable

Effect size of each drug comparison at each UC health site is tabulated.

eTable 2: Hazard ratios of drug class comparison at each UC

Comparator	Treated	UC	N	Hazard Ratio (95% CI)	P-value	Adjusted P-Value
DPP4i	GLP1ra	UC_1	712	0.78 (0.61-1)	5.82688e-02	8.740320e-02
DPP4i	GLP1ra	UC_2	1098	0.9 (0.74-1.1)	2.90125e-01	3.626563e-01
DPP4i	GLP1ra	UC_3	878	0.79 (0.65-0.96)	1.64345e-02	2.594921e-02
DPP4i	GLP1ra	UC_4	558	0.77 (0.56-1.1)	1.27240e-01	1.817714e-01
DPP4i	GLP1ra	UC_5	2722	0.79 (0.7-0.89)	1.38426e-04	4.152780e-04
DPP4i	SGLT2i	UC_1	1074	0.92 (0.75-1.1)	4.12140e-01	4.755462e-01
DPP4i	SGLT2i	UC_2	1088	0.93 (0.77-1.1)	4.83922e-01	5.376911e-01
DPP4i	SGLT2i	UC_3	720	0.95 (0.77-1.2)	6.33439e-01	6.786846e-01
DPP4i	SGLT2i	UC_4	568	1 (0.75-1.4)	8.78179e-01	9.084610e-01
DPP4i	SGLT2i	UC_5	3032	1 (0.89-1.1)	9.69732e-01	9.697320e-01
GLP1ra	SGLT2i	UC_1	724	1.4 (1.1-1.8)	1.47570e-02	2.459500e-02
GLP1ra	SGLT2i	UC_2	1316	1.1 (0.9-1.3)	3.62050e-01	4.344600e-01
GLP1ra	SGLT2i	UC_3	834	1.1 (0.92-1.4)	2.51503e-01	3.280474e-01
GLP1ra	SGLT2i	UC_4	664	1.3 (0.93-1.7)	1.42448e-01	1.942473e-01
GLP1ra	SGLT2i	UC_5	3140	1.4 (1.2-1.5)	5.25293e-08	5.252930e-07
Sulfonylurea	DPP4i	UC_1	1756	0.75 (0.65-0.87)	1.71236e-04	4.670073e-04
Sulfonylurea	DPP4i	UC_2	2072	0.84 (0.74-0.96)	8.67946e-03	1.531669e-02
Sulfonylurea	DPP4i	UC_3	2142	0.83 (0.74-0.93)	1.07591e-03	2.482869e-03
Sulfonylurea	DPP4i	UC_4	1284	0.71 (0.59-0.86)	3.68133e-04	9.203325e-04
Sulfonylurea	DPP4i	UC_5	5816	0.79 (0.74-0.86)	3.12448e-09	4.686720e-08
Sulfonylurea	GLP1ra	UC_1	752	0.55 (0.43-0.69)	4.16807e-07	3.126053e-06
Sulfonylurea	GLP1ra	UC_2	1174	0.69 (0.58-0.83)	7.41291e-05	2.779841e-04
Sulfonylurea	GLP1ra	UC_3	1098	0.68 (0.57-0.8)	5.57184e-06	2.785920e-05
Sulfonylurea	GLP1ra	UC_4	664	0.51 (0.38-0.67)	2.56941e-06	1.541646e-05
Sulfonylurea	GLP1ra	UC_5	2630	0.62 (0.56-0.7)	7.31700e-15	2.195100e-13
Sulfonylurea	SGLT2i	UC_1	1224	0.68 (0.57-0.81)	1.95578e-05	8.381914e-05
Sulfonylurea	SGLT2i	UC_2	1142	0.77 (0.64-0.92)	4.99311e-03	9.362081e-03
Sulfonylurea	SGLT2i	UC_3	990	0.71 (0.6-0.84)	8.50730e-05	2.835767e-04
Sulfonylurea	SGLT2i	UC_4	772	0.7 (0.55-0.9)	4.43296e-03	8.865920e-03
Sulfonylurea	SGLT2i	UC_5	3002	0.85 (0.76-0.95)	3.79867e-03	8.140007e-03

© 2023 Vashisht R et al. *JAMA Network Open.*

4.1.2 eFigure: Individual effect size, meta analysis and sensitivity analysis

The forest plot illustrate the effect size of the comparison between DPP4i and GLP1ra at each UC along with the effect size obtained from the random effect meta-analysis across all the UC for outcome HbA1c > 7%

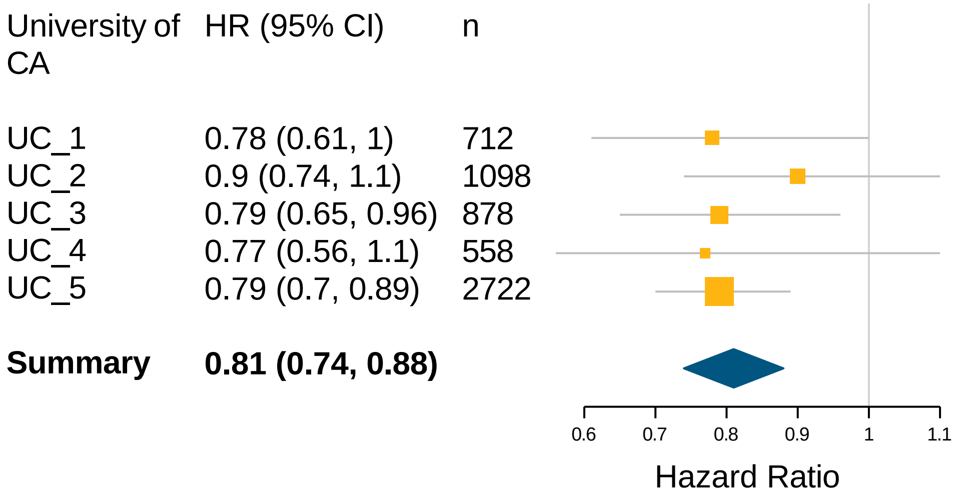

The table below shows the Leave-One-UC-Out diagnostics. The DFFITS value, Cook's distance, Covariance ratio, leave-one-out amount of heteroginity, indicator for influential estimates, comparator and treated groups are provided for each Leave-One-UC-Out analysis. The influential estimate from one UC with respect to pooled estimate are marked as Yes or No, with Yes indicating an influential UC and No otherwise.

eTable 3: Leave-One-UC-Out Sensitivity Analysis

DFFITs	Cook's Dist	Residual Heterogeneity	Influential	Comparator	Treated	UC
-0.0969772	0.0094046	0	No	DPP4i	GLP1ra	UC_1
0.5525578	0.3053202	0	No	DPP4i	GLP1ra	UC_2
-0.1025026	0.0105068	0	No	DPP4i	GLP1ra	UC_3
-0.0686349	0.0047108	0	No	DPP4i	GLP1ra	UC_4
-0.4216388	0.1777793	0	No	DPP4i	GLP1ra	UC_5

© 2023 Vashisht R et al. *JAMA Network Open.*

The forest plot illustrate the effect size of the comparison between DPP4i and SGLT2i at each UC along with the effect size obtained from the random effect meta-analysis across all the UC for outcome HbA1c > 7%

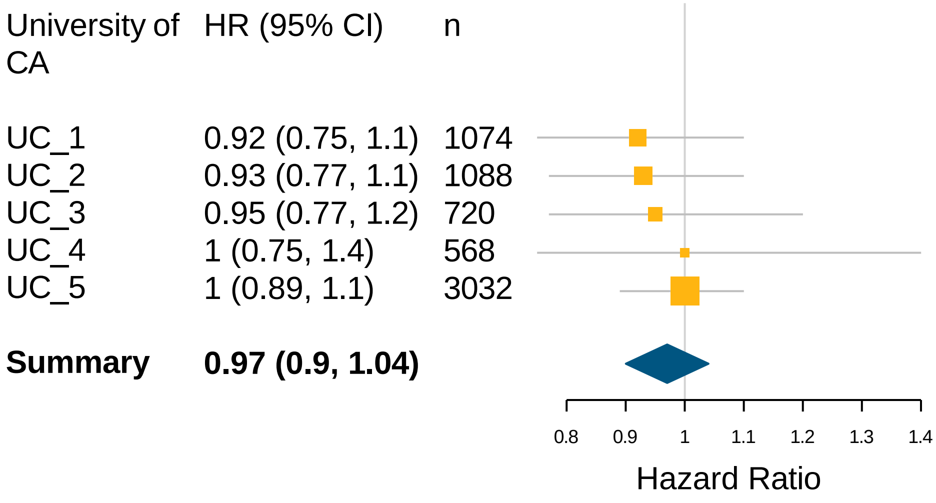

The table below shows the Leave-One-UC-Out diagnostics. The DFFITS value, Cook's distance, Covariance ratio, leave-one-out amount of heteroginity, indicator for influential estimates, comparator and treated groups are provided for each Leave-One-UC-Out analysis. The influential estimate from one UC with respect to pooled estimate are marked as Yes or No, with Yes indicating an influential UC and No otherwise.

eTable 4: Leave-One-UC-Out Sensitivity Analysis

DFFITs	Cook's Dist	Residual Heterogeneity	Influential	Comparator	Treated	UC
-0.2456418	0.0603399	0	No	DPP4i	SGLT2i	UC_1
-0.2307238	0.0532335	0	No	DPP4i	SGLT2i	UC_2
-0.0670242	0.0044922	0	No	DPP4i	SGLT2i	UC_3
0.0501512	0.0025151	0	No	DPP4i	SGLT2i	UC_4
0.8198123	0.6720923	0	Yes	DPP4i	SGLT2i	UC_5

The forest plot illustrate the effect size of the comparison between GLP1ra and SGLT2i at each UC along with the effect size obtained from the random effect meta-analysis across all the UC for outcome HbA1c > 7%

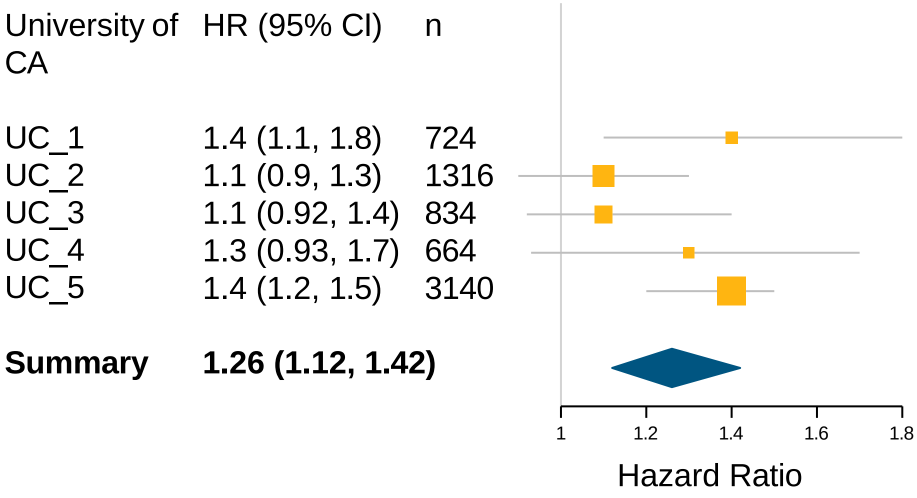

The table below shows the Leave-One-UC-Out diagnostics. The DFFITS value, Cook's distance, Covariance ratio, leave-one-out amount of heteroginity, indicator for influential estimates, comparator and treated groups are provided for each Leave-One-UC-Out analysis. The influential estimate from one UC with respect to pooled estimate are marked as Yes or No, with Yes indicating an influential UC and No otherwise.

eTable 5: Leave-One-UC-Out Sensitivity Analysis

DFFITs	Cook's Dist	Residual Heterogeneity	Influential	Comparator	Treated	UC
0.3519387	0.1415795	0.0120859	No	GLP1ra	SGLT2i	UC_1
-0.8276649	0.5039079	0.0039979	Yes	GLP1ra	SGLT2i	UC_2
-0.5578045	0.2809466	0.0066500	No	GLP1ra	SGLT2i	UC_3
0.1111153	0.0138795	0.0126053	No	GLP1ra	SGLT2i	UC_4
1.5981951	0.9293353	0.0010668	Yes	GLP1ra	SGLT2i	UC_5

© 2023 Vashisht R et al. *JAMA Network Open.*

The forest plot illustrate the effect size of the comparison between Sulfonylurea and DPP4i at each UC along with the effect size obtained from the random effect meta-analysis across all the UC for outcome HbA1c > 7%

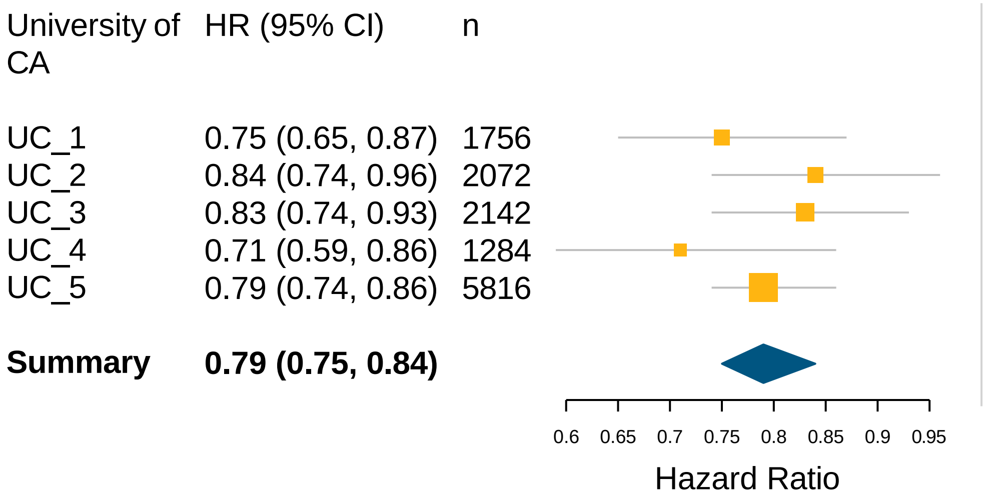

The table below shows the Leave-One-UC-Out diagnostics. The DFFITS value, Cook's distance, Covariance ratio, leave-one-out amount of heteroginity, indicator for influential estimates, comparator and treated groups are provided for each Leave-One-UC-Out analysis. The influential estimate from one UC with respect to pooled estimate are marked as Yes or No, with Yes indicating an influential UC and No otherwise.

eTable 6: Leave-One-UC-Out Sensitivity Analysis

DFFITs	Cook's Dist	Residual Heterogeneity	Influential	Comparator	Treated	UC
-0.3037747	0.0922791	0.0000000	No	Sulfonylurea	DPP4i	UC_1
0.3903836	0.1523993	0.0000000	No	Sulfonylurea	DPP4i	UC_2
0.4211994	0.1774089	0.0000000	No	Sulfonylurea	DPP4i	UC_3
-0.3379431	0.1142055	0.0000000	No	Sulfonylurea	DPP4i	UC_4
-0.1027254	0.0133059	0.0003835	No	Sulfonylurea	DPP4i	UC_5

© 2023 Vashisht R et al. *JAMA Network Open.*

The forest plot illustrate the effect size of the comparison between Sulfonylurea and GLP1ra at each UC along with the effect size obtained from the random effect meta-analysis across all the UC for outcome HbA1c > 7%

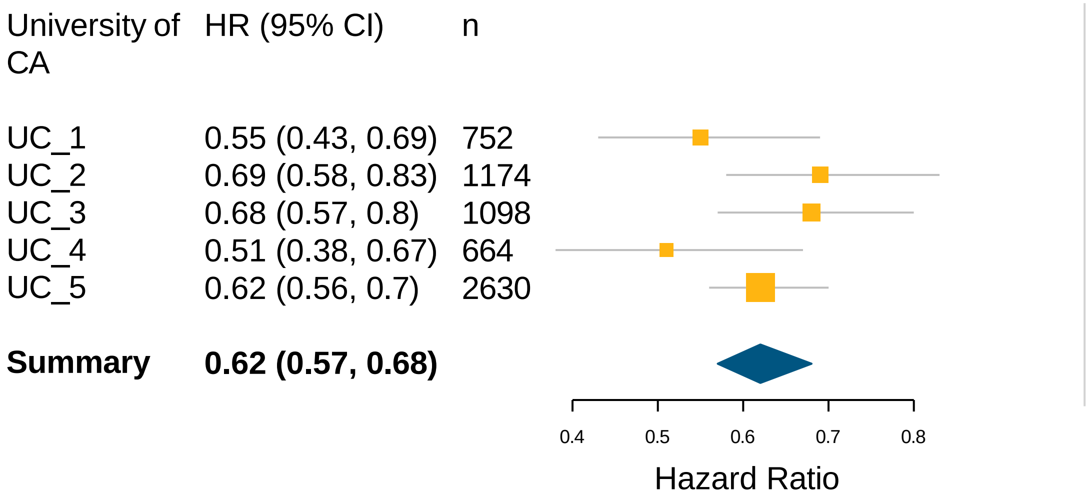

The table below shows the Leave-One-UC-Out diagnostics. The DFFITS value, Cook's distance, Covariance ratio, leave-one-out amount of heteroginity, indicator for influential estimates, comparator and treated groups are provided for each Leave-One-UC-Out analysis. The influential estimate from one UC with respect to pooled estimate are marked as Yes or No, with Yes indicating an influential UC and No otherwise.

eTable 7: Leave-One-UC-Out Sensitivity Analysis

DFFITs	Cook's Dist	Residual Heterogeneity	Influential	Comparator	Treated	UC
-0.4007500	0.1582928	0.0023227	No	Sulfonylurea	GLP1ra	UC_1
0.5254544	0.2774139	0.0026207	No	Sulfonylurea	GLP1ra	UC_2
0.5135165	0.2864116	0.0034341	No	Sulfonylurea	GLP1ra	UC_3
-0.4338276	0.1684358	0.0001003	No	Sulfonylurea	GLP1ra	UC_4
0.0925382	0.0168605	0.0081975	No	Sulfonylurea	GLP1ra	UC_5

© 2023 Vashisht R et al. *JAMA Network Open.*

The forest plot illustrate the effect size of the comparison between Sulfonylurea and SGLT2i at each UC along with the effect size obtained from the random effect meta-analysis across all the UC for outcome HbA1c > 7%

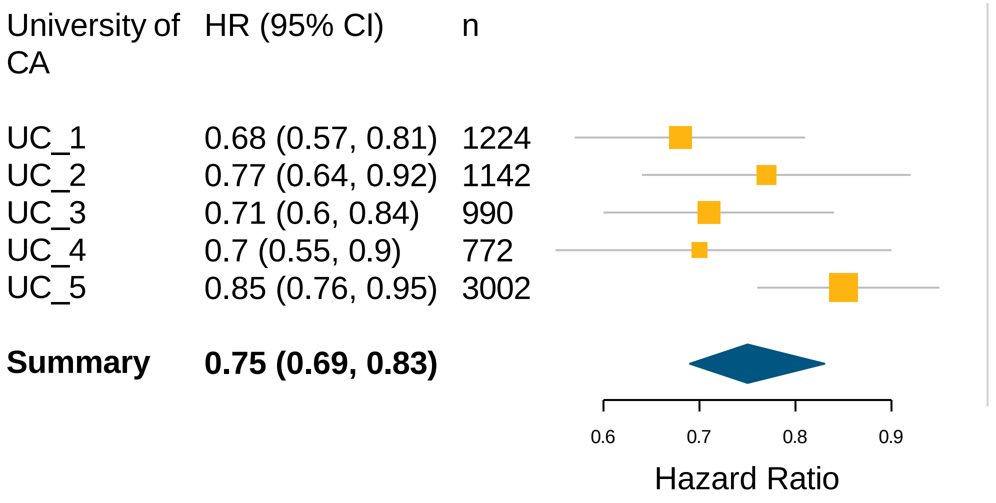

The table below shows the Leave-One-UC-Out diagnostics. The DFFITS value, Cook's distance, Covariance ratio, leave-one-out amount of heteroginity, indicator for influential estimates, comparator and treated groups are provided for each Leave-One-UC-Out analysis. The influential estimate from one UC with respect to pooled estimate are marked as Yes or No, with Yes indicating an influential UC and No otherwise.

eTable 8: Leave-One-UC-Out Sensitivity Analysis

DFFITs	Cook's Dist	Residual Heterogeneity	Influential	Comparator	Treated	UC
-0.6182934	0.3371966	0.0028834	No	Sulfonylurea	SGLT2i	UC_1
0.2106707	0.0571786	0.0080671	No	Sulfonylurea	SGLT2i	UC_2
-0.2316798	0.0608005	0.0058982	No	Sulfonylurea	SGLT2i	UC_3
-0.1481921	0.0236409	0.0058833	No	Sulfonylurea	SGLT2i	UC_4
1.6868432	1.2157931	0.0000000	Yes	Sulfonylurea	SGLT2i	UC_5

© 2023 Vashisht R et al. *JAMA Network Open.*

4.2 Abdominal Pain

4.2.1 eTable: Drug comparision table

Effect size of each drug comparison at each UC health site is tabulated.

eTable 9: Hazard ratios of drug class comparison at each UC

Comparator	Treated	UC	N	Hazard Ratio (95% CI)	P-value	Adjusted P-Value
DPP4i	GLP1ra	UC_1	565	1.1 (0.63-2.1)	6.52615e-01	8.512370e-01
DPP4i	GLP1ra	UC_2	847	0.97 (0.66-1.4)	8.75400e-01	9.055862e-01
DPP4i	GLP1ra	UC_3	638	1.5 (0.97-2.4)	6.50648e-02	6.372343e-01
DPP4i	GLP1ra	UC_4	455	1.1 (0.54-2.4)	7.24848e-01	8.698176e-01
DPP4i	GLP1ra	UC_5	2234	1.1 (0.84-1.4)	5.18336e-01	8.140543e-01
DPP4i	SGLT2i	UC_1	873	0.91 (0.55-1.5)	7.16508e-01	8.698176e-01
DPP4i	SGLT2i	UC_2	891	0.71 (0.45-1.1)	1.41858e-01	6.372343e-01
DPP4i	SGLT2i	UC_3	485	0.75 (0.39-1.4)	3.80759e-01	7.846700e-01
DPP4i	SGLT2i	UC_4	465	1.9 (0.86-4.1)	1.15336e-01	6.372343e-01
DPP4i	SGLT2i	UC_5	2549	0.99 (0.75-1.3)	9.70970e-01	9.709700e-01
GLP1ra	SGLT2i	UC_1	560	0.92 (0.46-1.8)	8.10952e-01	8.956875e-01
GLP1ra	SGLT2i	UC_2	1012	0.6 (0.39-0.92)	2.03492e-02	3.129045e-01
GLP1ra	SGLT2i	UC_3	560	0.53 (0.31-0.91)	2.08603e-02	3.129045e-01
GLP1ra	SGLT2i	UC_4	523	1.4 (0.66-2.9)	3.92335e-01	7.846700e-01
GLP1ra	SGLT2i	UC_5	2489	0.83 (0.63-1.1)	1.75545e-01	6.582938e-01
Sulfonylurea	DPP4i	UC_1	1487	0.91 (0.65-1.3)	5.60362e-01	8.140543e-01
Sulfonylurea	DPP4i	UC_2	1745	1.1 (0.84-1.4)	5.33231e-01	8.140543e-01
Sulfonylurea	DPP4i	UC_3	1636	1.1 (0.86-1.4)	4.28183e-01	8.028431e-01
Sulfonylurea	DPP4i	UC_4	1099	0.8 (0.52-1.2)	3.02585e-01	7.846700e-01
Sulfonylurea	DPP4i	UC_5	5041	1.1 (0.91-1.3)	3.81148e-01	7.846700e-01
Sulfonylurea	GLP1ra	UC_1	597	1.6 (0.85-2.9)	1.48688e-01	6.372343e-01
Sulfonylurea	GLP1ra	UC_2	937	1.2 (0.83-1.8)	3.02739e-01	7.846700e-01
Sulfonylurea	GLP1ra	UC_3	795	1.4 (0.91-2)	1.33052e-01	6.372343e-01
Sulfonylurea	GLP1ra	UC_4	555	0.92 (0.48-1.8)	7.90452e-01	8.956875e-01
Sulfonylurea	GLP1ra	UC_5	2176	1.2 (0.87-1.5)	3.24965e-01	7.846700e-01
Sulfonylurea	SGLT2i	UC_1	1011	1.2 (0.71-2)	4.93420e-01	8.140543e-01
Sulfonylurea	SGLT2i	UC_2	958	0.74 (0.46-1.2)	2.13906e-01	7.130200e-01
Sulfonylurea	SGLT2i	UC_3	696	0.88 (0.51-1.5)	6.27274e-01	8.512370e-01
Sulfonylurea	SGLT2i	UC_4	641	0.93 (0.49-1.8)	8.35975e-01	8.956875e-01
Sulfonylurea	SGLT2i	UC_5	2558	1.1 (0.82-1.4)	5.69838e-01	8.140543e-01

4.2.2 eFigure: Individual effect size, meta analysis and sensitivity analysis

The forest plot illustrate the effect size of the comparison between DPP4i and GLP1ra at each UC along with the effect size obtained from the random effect meta-analysis across all the UC for outcome Abdominal Pain

© 2023 Vashisht R et al. *JAMA Network Open.*

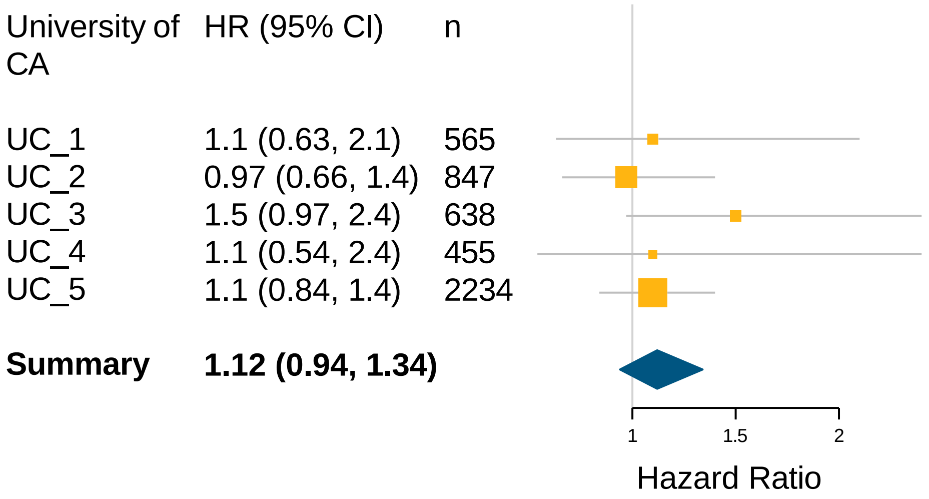

The table below shows the Leave-One-UC-Out diagnostics. The DFFITS value, Cook's distance, Covariance ratio, leave-one-out amount of heteroginity, indicator for influential estimates, comparator and treated groups are provided for each Leave-One-UC-Out analysis. The influential estimate from one UC with respect to pooled estimate are marked as Yes or No, with Yes indicating an influential UC and No otherwise.

eTable 10: Leave-One-UC-Out Sensitivity Analysis

DFFITs	Cook's Dist	Residual Heterogeneity	Influential	Comparator	Treated	UC
-0.0205080	0.0004206	0	No	DPP4i	GLP1ra	UC_1
-0.4590603	0.2107364	0	No	DPP4i	GLP1ra	UC_2
0.5809866	0.3375454	0	No	DPP4i	GLP1ra	UC_3
-0.0129326	0.0001673	0	No	DPP4i	GLP1ra	UC_4
-0.2006840	0.0402740	0	No	DPP4i	GLP1ra	UC_5

The forest plot illustrate the effect size of the comparison between DPP4i and SGLT2i at each UC along with the effect size obtained from the random effect meta-analysis across all the UC for outcome Abdominal Pain

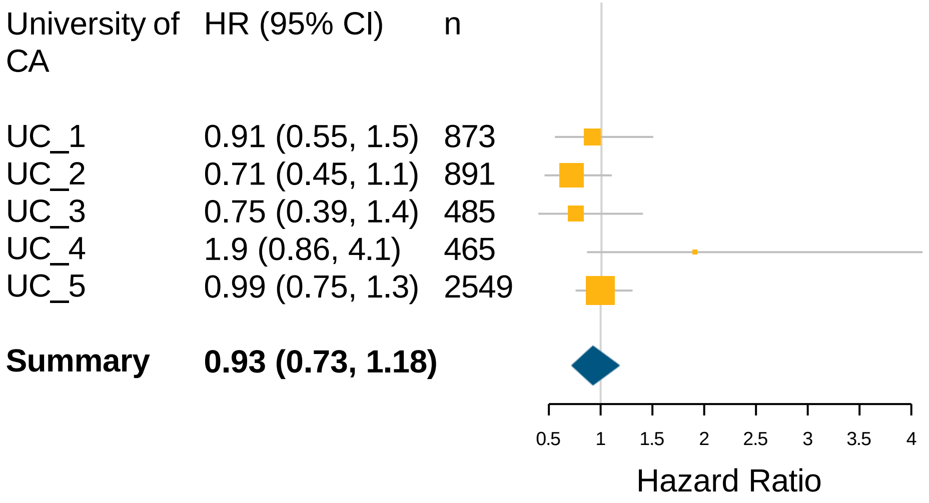

The table below shows the Leave-One-UC-Out diagnostics. The DFFITS value, Cook's distance, Covariance ratio, leave-one-out amount of heteroginity, indicator for influential estimates, comparator and treated groups are provided for each Leave-One-UC-Out analysis. The influential estimate from one UC with respect to pooled estimate are marked as Yes or No, with Yes indicating an influential UC and No otherwise.

eTable 11: Leave-One-UC-Out Sensitivity Analysis

DFFITs	Cook's Dist	Residual Heterogeneity	Influential	Comparator	Treated	UC
-0.0665573	0.0058377	0.0451113	No	DPP4i	SGLT2i	UC_1
-0.6087975	0.3322413	0.0111379	No	DPP4i	SGLT2i	UC_2
-0.2661812	0.0782232	0.0314098	No	DPP4i	SGLT2i	UC_3
0.3775259	0.1276978	0.0000000	No	DPP4i	SGLT2i	UC_4
0.1370782	0.0356226	0.0525831	No	DPP4i	SGLT2i	UC_5

The forest plot illustrate the effect size of the comparison between GLP1ra and SGLT2i at each UC along with the effect size obtained from the random effect meta-analysis across all the UC for outcome Abdominal Pain

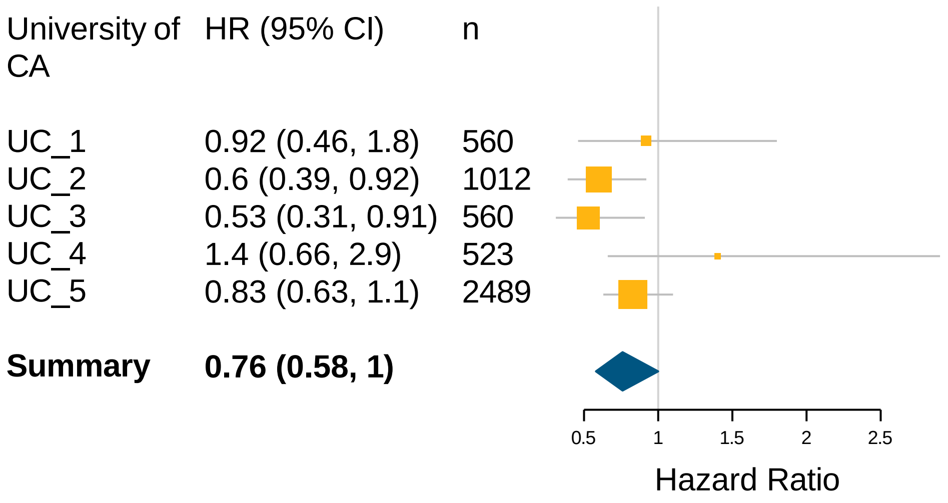

The table below shows the Leave-One-UC-Out diagnostics. The DFFITS value, Cook's distance, Covariance ratio, leave-one-out amount of heteroginity, indicator for influential estimates, comparator and treated groups are provided for each Leave-One-UC-Out analysis. The influential estimate from one UC with respect to pooled estimate are marked as Yes or No, with Yes indicating an influential UC and No otherwise.

eTable 12: Leave-One-UC-Out Sensitivity Analysis

DFFITs	Cook's Dist	Residual Heterogeneity	Influential	Comparator	Treated	UC
0.1727924	0.0334088	0.0510872	No	GLP1ra	SGLT2i	UC_1
-0.5257021	0.2954950	0.0383664	No	GLP1ra	SGLT2i	UC_2
-0.5447179	0.2687698	0.0225829	No	GLP1ra	SGLT2i	UC_3
0.4353612	0.1619221	0.0072257	No	GLP1ra	SGLT2i	UC_4
0.1400163	0.0342480	0.0723626	No	GLP1ra	SGLT2i	UC_5

The forest plot illustrate the effect size of the comparison between Sulfonylurea and DPP4i at each UC along with the effect size obtained from the random effect meta-analysis across all the UC for outcome Abdominal Pain

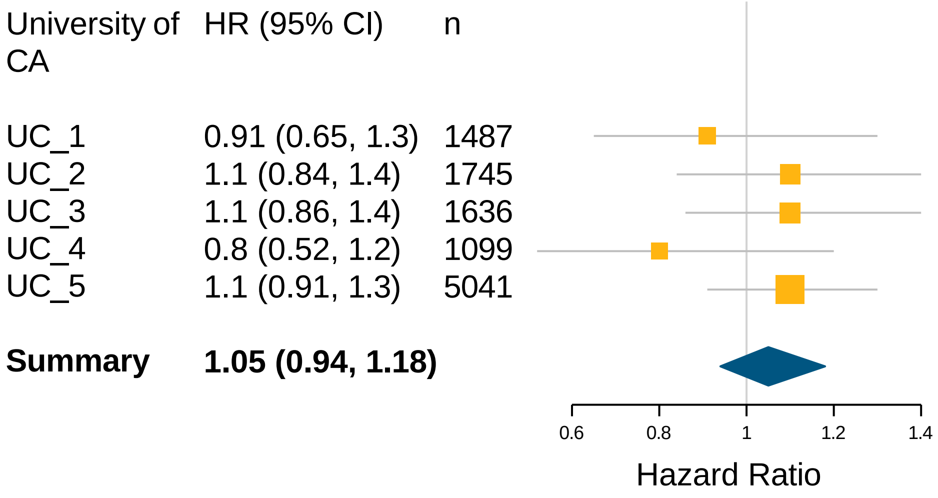

The table below shows the Leave-One-UC-Out diagnostics. The DFFITS value, Cook's distance, Covariance ratio, leave-one-out amount of heteroginity, indicator for influential estimates, comparator and treated groups are provided for each Leave-One-UC-Out analysis. The influential estimate from one UC with respect to pooled estimate are marked as Yes or No, with Yes indicating an influential UC and No otherwise.

eTable 13: Leave-One-UC-Out Sensitivity Analysis

DFFITs	Cook's Dist	Residual Heterogeneity	Influential	Comparator	Treated	UC
-0.3024629	0.0914838	0	No	Sulfonylurea	DPP4i	UC_1
0.1859719	0.0345855	0	No	Sulfonylurea	DPP4i	UC_2
0.2094608	0.0438738	0	No	Sulfonylurea	DPP4i	UC_3
-0.3772011	0.1422807	0	No	Sulfonylurea	DPP4i	UC_4
0.5144446	0.2646532	0	No	Sulfonylurea	DPP4i	UC_5

© 2023 Vashisht R et al. *JAMA Network Open.*

The forest plot illustrate the effect size of the comparison between Sulfonylurea and GLP1ra at each UC along with the effect size obtained from the random effect meta-analysis across all the UC for outcome Abdominal Pain

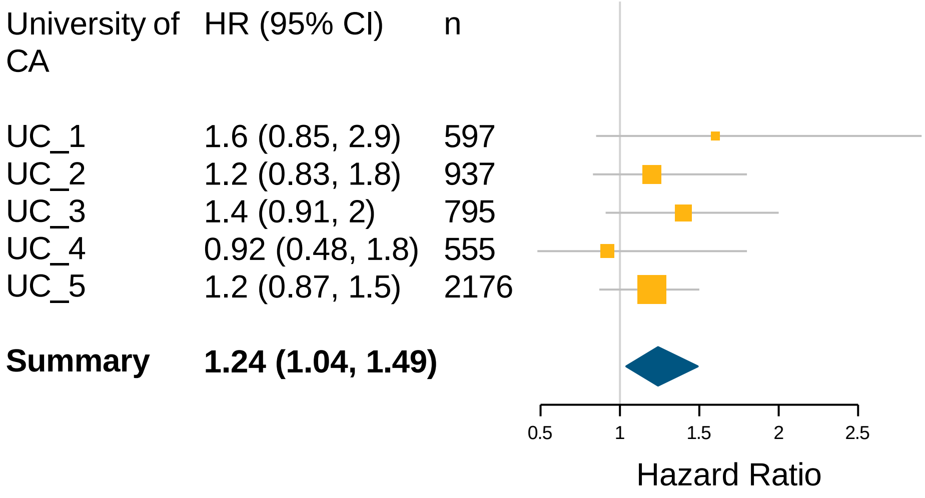

The table below shows the Leave-One-UC-Out diagnostics. The DFFITS value, Cook's distance, Covariance ratio, leave-one-out amount of heteroginity, indicator for influential estimates, comparator and treated groups are provided for each Leave-One-UC-Out analysis. The influential estimate from one UC with respect to pooled estimate are marked as Yes or No, with Yes indicating an influential UC and No otherwise.

eTable 14: Leave-One-UC-Out Sensitivity Analysis

DFFITs	Cook's Dist	Residual Heterogeneity	Influential	Comparator	Treated	UC
0.2542193	0.0646274	0	No	Sulfonylurea	GLP1ra	UC_1
-0.1076768	0.0115943	0	No	Sulfonylurea	GLP1ra	UC_2
0.3330061	0.1108930	0	No	Sulfonylurea	GLP1ra	UC_3
-0.2602804	0.0677459	0	No	Sulfonylurea	GLP1ra	UC_4
-0.2993517	0.0896114	0	No	Sulfonylurea	GLP1ra	UC_5

© 2023 Vashisht R et al. *JAMA Network Open.*

The forest plot illustrate the effect size of the comparison between Sulfonylurea and SGLT2i at each UC along with the effect size obtained from the random effect meta-analysis across all the UC for outcome Abdominal Pain

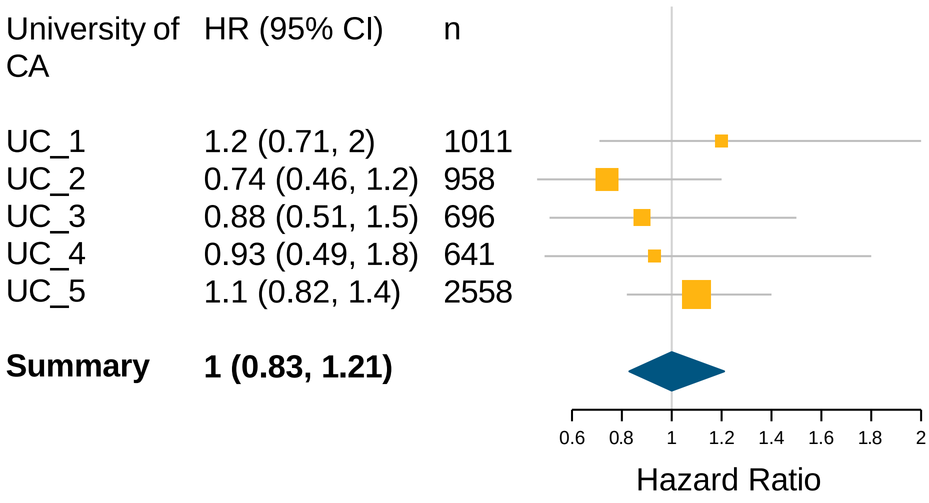

The table below shows the Leave-One-UC-Out diagnostics. The DFFITS value, Cook's distance, Covariance ratio, leave-one-out amount of heteroginity, indicator for influential estimates, comparator and treated groups are provided for each Leave-One-UC-Out analysis. The influential estimate from one UC with respect to pooled estimate are marked as Yes or No, with Yes indicating an influential UC and No otherwise.

eTable 15: Leave-One-UC-Out Sensitivity Analysis

DFFITs	Cook's Dist	Residual Heterogeneity	Influential	Comparator	Treated	UC
0.2862686	0.0819497	0	No	Sulfonylurea	SGLT2i	UC_1
-0.5827707	0.3396216	0	No	Sulfonylurea	SGLT2i	UC_2
-0.1908878	0.0364382	0	No	Sulfonylurea	SGLT2i	UC_3
-0.0727191	0.0052881	0	No	Sulfonylurea	SGLT2i	UC_4
0.9588228	0.9193412	0	Yes	Sulfonylurea	SGLT2i	UC_5

© 2023 Vashisht R et al. *JAMA Network Open.*

4.3 Cardiac Arrhythmia

4.3.1 eTable: Drug comparision table

Effect size of each drug comparison at each UC health site is tabulated.

eTable 16: Hazard ratios of drug class comparison at each UC

Comparator	Treated	UC	N	Hazard Ratio (95% CI)	P-value	Adjusted P-Value
DPP4i	GLP1ra	UC_1	583	1 (0.54-1.9)	9.97210e-01	9.99597e-01
DPP4i	GLP1ra	UC_2	883	1.2 (0.78-1.8)	4.19298e-01	9.99597e-01
DPP4i	GLP1ra	UC_3	671	1.2 (0.71-2)	5.03548e-01	9.99597e-01
DPP4i	GLP1ra	UC_4	454	0.84 (0.44-1.6)	6.03518e-01	9.99597e-01
DPP4i	GLP1ra	UC_5	2275	0.78 (0.58-1)	1.00468e-01	9.99597e-01
DPP4i	SGLT2i	UC_1	861	1.1 (0.68-1.8)	6.65103e-01	9.99597e-01
DPP4i	SGLT2i	UC_2	825	1.1 (0.69-1.7)	7.62594e-01	9.99597e-01
DPP4i	SGLT2i	UC_3	530	0.94 (0.54-1.6)	8.31391e-01	9.99597e-01
DPP4i	SGLT2i	UC_4	432	1.6 (0.74-3.4)	2.43220e-01	9.99597e-01
DPP4i	SGLT2i	UC_5	2552	0.96 (0.73-1.3)	7.51106e-01	9.99597e-01
GLP1ra	SGLT2i	UC_1	589	1.4 (0.74-2.6)	3.15848e-01	9.99597e-01
GLP1ra	SGLT2i	UC_2	981	0.8 (0.53-1.2)	2.92798e-01	9.99597e-01
GLP1ra	SGLT2i	UC_3	617	1.2 (0.65-2.1)	6.02969e-01	9.99597e-01
GLP1ra	SGLT2i	UC_4	518	1 (0.51-2)	9.94442e-01	9.99597e-01
GLP1ra	SGLT2i	UC_5	2512	1.3 (0.93-1.7)	1.39217e-01	9.99597e-01
Sulfonylurea	DPP4i	UC_1	1495	1 (0.72-1.4)	9.99597e-01	9.99597e-01
Sulfonylurea	DPP4i	UC_2	1687	0.91 (0.7-1.2)	4.59348e-01	9.99597e-01
Sulfonylurea	DPP4i	UC_3	1688	0.99 (0.76-1.3)	9.24044e-01	9.99597e-01
Sulfonylurea	DPP4i	UC_4	1036	0.79 (0.56-1.1)	1.73027e-01	9.99597e-01
Sulfonylurea	DPP4i	UC_5	5081	1.1 (0.89-1.3)	4.91823e-01	9.99597e-01
Sulfonylurea	GLP1ra	UC_1	632	0.88 (0.48-1.6)	6.75656e-01	9.99597e-01
Sulfonylurea	GLP1ra	UC_2	962	0.91 (0.63-1.3)	6.23793e-01	9.99597e-01
Sulfonylurea	GLP1ra	UC_3	852	0.86 (0.55-1.3)	4.98800e-01	9.99597e-01
Sulfonylurea	GLP1ra	UC_4	550	0.8 (0.44-1.4)	4.56569e-01	9.99597e-01
Sulfonylurea	GLP1ra	UC_5	2245	0.98 (0.72-1.3)	8.79967e-01	9.99597e-01
Sulfonylurea	SGLT2i	UC_1	1014	1 (0.66-1.6)	8.53725e-01	9.99597e-01
Sulfonylurea	SGLT2i	UC_2	886	0.96 (0.63-1.4)	8.41388e-01	9.99597e-01
Sulfonylurea	SGLT2i	UC_3	715	1.4 (0.84-2.4)	1.94239e-01	9.99597e-01
Sulfonylurea	SGLT2i	UC_4	587	0.6 (0.34-1)	7.02161e-02	9.99597e-01
Sulfonylurea	SGLT2i	UC_5	2562	1.1 (0.82-1.4)	5.68734e-01	9.99597e-01

4.3.2 eFigure: Individual effect size, meta analysis and sensitivity analysis

The forest plot illustrate the effect size of the comparison between DPP4i and GLP1ra at each UC along with the effect size obtained from the random effect meta-analysis across all the UC for outcome Cardiac Arrhythmia

© 2023 Vashisht R et al. *JAMA Network Open.*

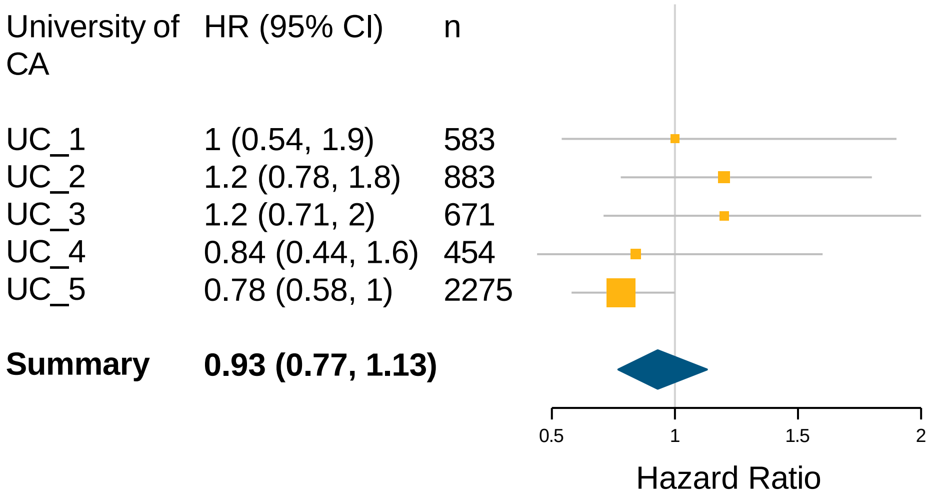

The table below shows the Leave-One-UC-Out diagnostics. The DFFITS value, Cook's distance, Covariance ratio, leave-one-out amount of heteroginity, indicator for influential estimates, comparator and treated groups are provided for each Leave-One-UC-Out analysis. The influential estimate from one UC with respect to pooled estimate are marked as Yes or No, with Yes indicating an influential UC and No otherwise.

eTable 17: Leave-One-UC-Out Sensitivity Analysis

DFFITs	Cook's Dist	Residual Heterogeneity	Influential	Comparator	Treated	UC
-0.1753115	0.0353047	0.0169821	No	DPP4i	GLP1ra	UC_1
0.7173715	0.4987409	0.0000000	Yes	DPP4i	GLP1ra	UC_2
0.4305897	0.1827978	0.0004463	No	DPP4i	GLP1ra	UC_3
-0.3762595	0.1604427	0.0161006	No	DPP4i	GLP1ra	UC_4
-1.6805008	2.6269470	0.0000000	Yes	DPP4i	GLP1ra	UC_5

© 2023 Vashisht R et al. *JAMA Network Open.*

The forest plot illustrate the effect size of the comparison between DPP4i and SGLT2i at each UC along with the effect size obtained from the random effect meta-analysis across all the UC for outcome Cardiac Arrhythmia

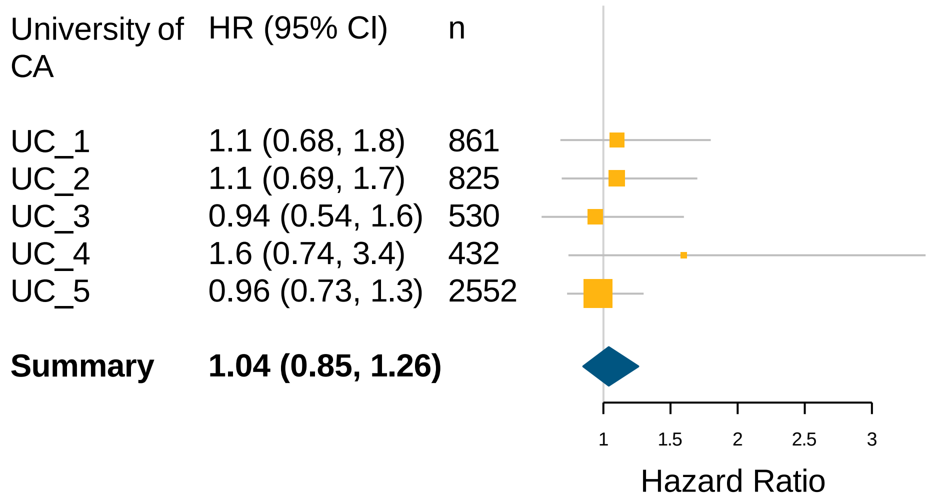

The table below shows the Leave-One-UC-Out diagnostics. The DFFITS value, Cook's distance, Covariance ratio, leave-one-out amount of heteroginity, indicator for influential estimates, comparator and treated groups are provided for each Leave-One-UC-Out analysis. The influential estimate from one UC with respect to pooled estimate are marked as Yes or No, with Yes indicating an influential UC and No otherwise.

eTable 18: Leave-One-UC-Out Sensitivity Analysis

DFFITs	Cook's Dist	Residual Heterogeneity	Influential	Comparator	Treated	UC
0.1115848	0.0124512	0	No	DPP4i	SGLT2i	UC_1
0.1343081	0.0180387	0	No	DPP4i	SGLT2i	UC_2
-0.1476591	0.0218032	0	No	DPP4i	SGLT2i	UC_3
0.3046316	0.0928004	0	No	DPP4i	SGLT2i	UC_4
-0.6613494	0.4373830	0	No	DPP4i	SGLT2i	UC_5

© 2023 Vashisht R et al. *JAMA Network Open.*

The forest plot illustrate the effect size of the comparison between GLP1ra and SGLT2i at each UC along with the effect size obtained from the random effect meta-analysis across all the UC for outcome Cardiac Arrhythmia

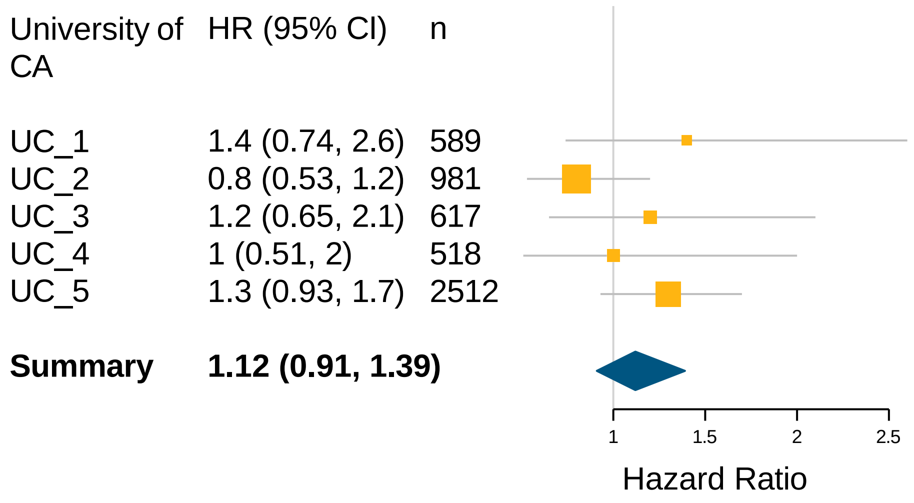

The table below shows the Leave-One-UC-Out diagnostics. The DFFITS value, Cook's distance, Covariance ratio, leave-one-out amount of heteroginity, indicator for influential estimates, comparator and treated groups are provided for each Leave-One-UC-Out analysis. The influential estimate from one UC with respect to pooled estimate are marked as Yes or No, with Yes indicating an influential UC and No otherwise.

eTable 19: Leave-One-UC-Out Sensitivity Analysis

DFFITs	Cook's Dist	Residual Heterogeneity	Influential	Comparator	Treated	UC
0.3245698	0.1147872	0.0121949	No	GLP1ra	SGLT2i	UC_1
-1.0904841	1.1186557	0.0000000	Yes	GLP1ra	SGLT2i	UC_2
0.1721519	0.0355649	0.0211914	No	GLP1ra	SGLT2i	UC_3
-0.0583852	0.0038436	0.0185872	No	GLP1ra	SGLT2i	UC_4
1.1233371	1.1310551	0.0000000	Yes	GLP1ra	SGLT2i	UC_5

© 2023 Vashisht R et al. *JAMA Network Open.*

The forest plot illustrate the effect size of the comparison between Sulfonylurea and DPP4i at each UC along with the effect size obtained from the random effect meta-analysis across all the UC for outcome Cardiac Arrhythmia

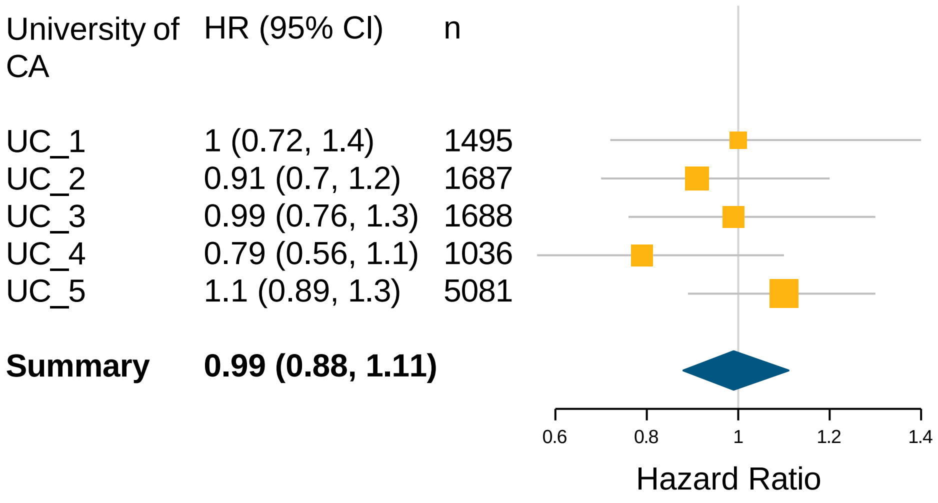

The table below shows the Leave-One-UC-Out diagnostics. The DFFITS value, Cook's distance, Covariance ratio, leave-one-out amount of heteroginity, indicator for influential estimates, comparator and treated groups are provided for each Leave-One-UC-Out analysis. The influential estimate from one UC with respect to pooled estimate are marked as Yes or No, with Yes indicating an influential UC and No otherwise.

eTable 20: Leave-One-UC-Out Sensitivity Analysis

DFFITs	Cook's Dist	Residual Heterogeneity	Influential	Comparator	Treated	UC
0.1002730	0.0106157	0.0016059	No	Sulfonylurea	DPP4i	UC_1
-0.3208661	0.1029550	0.0000000	No	Sulfonylurea	DPP4i	UC_2
0.0911992	0.0091239	0.0018187	No	Sulfonylurea	DPP4i	UC_3
-0.5113404	0.2614690	0.0000000	No	Sulfonylurea	DPP4i	UC_4
1.0995631	1.2090391	0.0000000	Yes	Sulfonylurea	DPP4i	UC_5

© 2023 Vashisht R et al. *JAMA Network Open.*

The forest plot illustrate the effect size of the comparison between Sulfonylurea and GLP1ra at each UC along with the effect size obtained from the random effect meta-analysis across all the UC for outcome Cardiac Arrhythmia

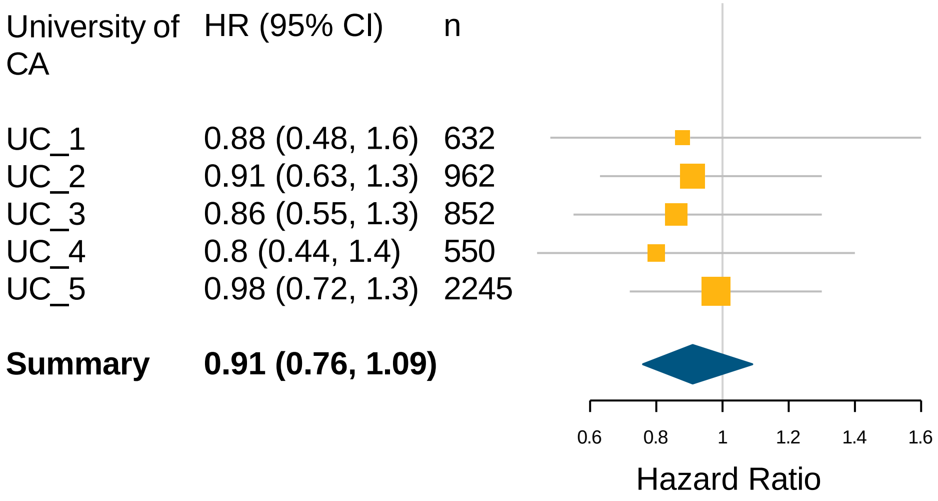

The table below shows the Leave-One-UC-Out diagnostics. The DFFITS value, Cook's distance, Covariance ratio, leave-one-out amount of heteroginity, indicator for influential estimates, comparator and treated groups are provided for each Leave-One-UC-Out analysis. The influential estimate from one UC with respect to pooled estimate are marked as Yes or No, with Yes indicating an influential UC and No otherwise.

eTable 21: Leave-One-UC-Out Sensitivity Analysis

DFFITs	Cook's Dist	Residual Heterogeneity	Influential	Comparator	Treated	UC
-0.0386655	0.0014950	0	No	Sulfonylurea	GLP1ra	UC_1
-0.0079815	0.0000637	0	No	Sulfonylurea	GLP1ra	UC_2
-0.1377677	0.0189799	0	No	Sulfonylurea	GLP1ra	UC_3
-0.1547419	0.0239450	0	No	Sulfonylurea	GLP1ra	UC_4
0.4729338	0.2236663	0	No	Sulfonylurea	GLP1ra	UC_5

© 2023 Vashisht R et al. *JAMA Network Open.*

The forest plot illustrate the effect size of the comparison between Sulfonylurea and SGLT2i at each UC along with the effect size obtained from the random effect meta-analysis across all the UC for outcome Cardiac Arrhythmia

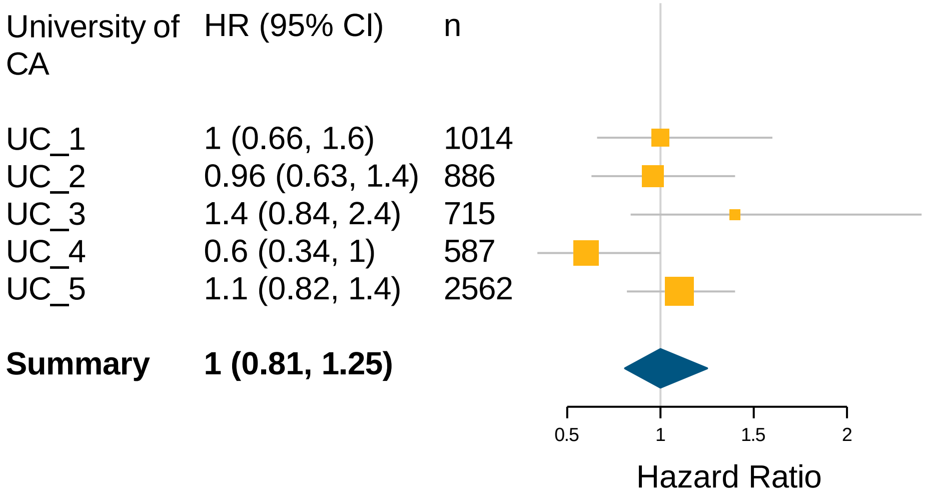

The table below shows the Leave-One-UC-Out diagnostics. The DFFITS value, Cook's distance, Covariance ratio, leave-one-out amount of heteroginity, indicator for influential estimates, comparator and treated groups are provided for each Leave-One-UC-Out analysis. The influential estimate from one UC with respect to pooled estimate are marked as Yes or No, with Yes indicating an influential UC and No otherwise.

eTable 22: Leave-One-UC-Out Sensitivity Analysis

DFFITs	Cook's Dist	Residual Heterogeneity	Influential	Comparator	Treated	UC
0.0655038	0.0055549	0.0366907	No	Sulfonylurea	SGLT2i	UC_1
-0.0179388	0.0004374	0.0376406	No	Sulfonylurea	SGLT2i	UC_2
0.4457096	0.1886712	0.0122811	No	Sulfonylurea	SGLT2i	UC_3
-0.7613173	0.4747552	0.0000000	Yes	Sulfonylurea	SGLT2i	UC_4
0.3592859	0.2037462	0.0371724	No	Sulfonylurea	SGLT2i	UC_5

© 2023 Vashisht R et al. *JAMA Network Open.*

4.4 Cardiovascular Disease

4.4.1 eTable: Drug comparision table

Effect size of each drug comparison at each UC health site is tabulated.

eTable 23: Hazard ratios of drug class comparison at each UC

Comparator	Treated	UC	N	Hazard Ratio (95% CI)	P-value	Adjusted P-Value
DPP4i	GLP1ra	UC_1	630	0.57 (0.25-1.3)	1.75194e-01	5.839800e-01
DPP4i	GLP1ra	UC_2	930	1.1 (0.66-1.9)	6.62000e-01	8.050164e-01
DPP4i	GLP1ra	UC_3	763	0.71 (0.37-1.4)	3.07489e-01	6.097017e-01
DPP4i	GLP1ra	UC_4	493	0.73 (0.29-1.8)	4.95576e-01	7.824884e-01
DPP4i	GLP1ra	UC_5	2501	0.98 (0.64-1.5)	9.42988e-01	9.969390e-01
DPP4i	SGLT2i	UC_1	899	0.87 (0.46-1.6)	6.70847e-01	8.050164e-01
DPP4i	SGLT2i	UC_2	877	1.4 (0.87-2.4)	1.60191e-01	5.839800e-01
DPP4i	SGLT2i	UC_3	567	0.65 (0.3-1.4)	2.72802e-01	6.097017e-01
DPP4i	SGLT2i	UC_4	462	1.2 (0.5-3)	6.65159e-01	8.050164e-01
DPP4i	SGLT2i	UC_5	2752	1 (0.66-1.5)	9.96939e-01	9.969390e-01
GLP1ra	SGLT2i	UC_1	626	1.9 (0.69-5.1)	2.22078e-01	6.097017e-01
GLP1ra	SGLT2i	UC_2	1061	1.3 (0.79-2.2)	2.80576e-01	6.097017e-01
GLP1ra	SGLT2i	UC_3	680	0.5 (0.2-1.2)	1.26568e-01	5.424343e-01
GLP1ra	SGLT2i	UC_4	567	1.6 (0.59-4.1)	3.65821e-01	6.097017e-01
GLP1ra	SGLT2i	UC_5	2847	1 (0.66-1.5)	9.83574e-01	9.969390e-01
Sulfonylurea	DPP4i	UC_1	1510	0.96 (0.67-1.4)	8.03537e-01	9.271581e-01
Sulfonylurea	DPP4i	UC_2	1732	0.78 (0.58-1.1)	1.01382e-01	5.424343e-01
Sulfonylurea	DPP4i	UC_3	1837	0.99 (0.73-1.3)	9.25687e-01	9.969390e-01
Sulfonylurea	DPP4i	UC_4	1109	0.73 (0.49-1.1)	1.15715e-01	5.424343e-01
Sulfonylurea	DPP4i	UC_5	5310	0.79 (0.64-0.98)	3.34694e-02	3.346940e-01
Sulfonylurea	GLP1ra	UC_1	675	0.68 (0.32-1.5)	3.30570e-01	6.097017e-01
Sulfonylurea	GLP1ra	UC_2	992	0.76 (0.47-1.2)	2.69038e-01	6.097017e-01
Sulfonylurea	GLP1ra	UC_3	947	0.42 (0.24-0.75)	3.54805e-03	1.064415e-01
Sulfonylurea	GLP1ra	UC_4	592	0.54 (0.25-1.2)	1.11669e-01	5.424343e-01
Sulfonylurea	GLP1ra	UC_5	2431	0.88 (0.58-1.3)	5.44507e-01	8.050164e-01
Sulfonylurea	SGLT2i	UC_1	1030	0.74 (0.41-1.3)	3.13921e-01	6.097017e-01
Sulfonylurea	SGLT2i	UC_2	907	0.89 (0.57-1.4)	6.03023e-01	8.050164e-01
Sulfonylurea	SGLT2i	UC_3	760	0.72 (0.36-1.5)	3.58907e-01	6.097017e-01
Sulfonylurea	SGLT2i	UC_4	636	0.49 (0.26-0.92)	2.73359e-02	3.346940e-01
Sulfonylurea	SGLT2i	UC_5	2715	0.89 (0.61-1.3)	5.76577e-01	8.050164e-01

4.4.2 eFigure: Individual effect size, meta analysis and sensitivity analysis

The forest plot illustrate the effect size of the comparison between DPP4i and GLP1ra at each UC along with the effect size obtained from the random effect meta-analysis across all the UC for outcome Cardiovascular Disease

© 2023 Vashisht R et al. *JAMA Network Open.*

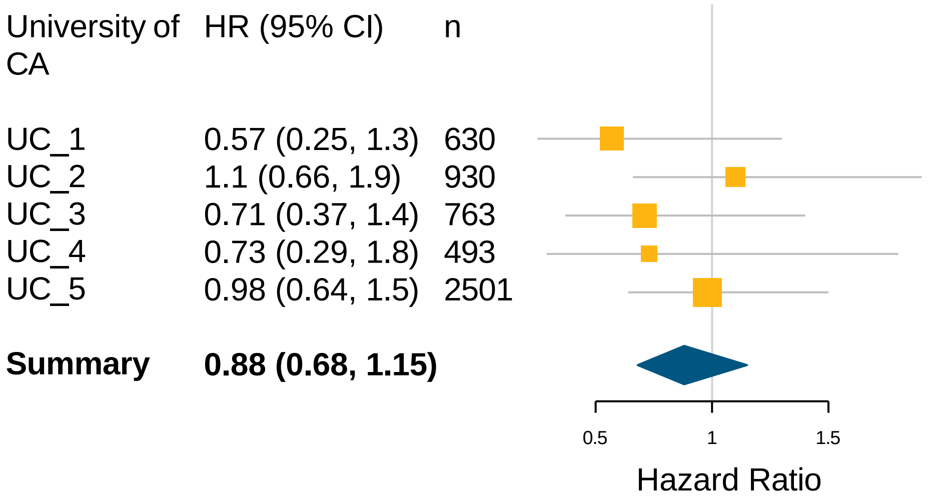

The table below shows the Leave-One-UC-Out diagnostics. The DFFITS value, Cook's distance, Covariance ratio, leave-one-out amount of heteroginity, indicator for influential estimates, comparator and treated groups are provided for each Leave-One-UC-Out analysis. The influential estimate from one UC with respect to pooled estimate are marked as Yes or No, with Yes indicating an influential UC and No otherwise.

eTable 24: Leave-One-UC-Out Sensitivity Analysis

DFFITs	Cook's Dist	Residual Heterogeneity	Influential	Comparator	Treated	UC
-0.3764141	0.1416876	0	No	DPP4i	GLP1ra	UC_1
0.5532046	0.3060353	0	No	DPP4i	GLP1ra	UC_2
-0.3068986	0.0941868	0	No	DPP4i	GLP1ra	UC_3
-0.1305070	0.0170321	0	No	DPP4i	GLP1ra	UC_4
0.4971300	0.2471382	0	No	DPP4i	GLP1ra	UC_5

© 2023 Vashisht R et al. *JAMA Network Open.*

The forest plot illustrate the effect size of the comparison between DPP4i and SGLT2i at each UC along with the effect size obtained from the random effect meta-analysis across all the UC for outcome Cardiovascular Disease

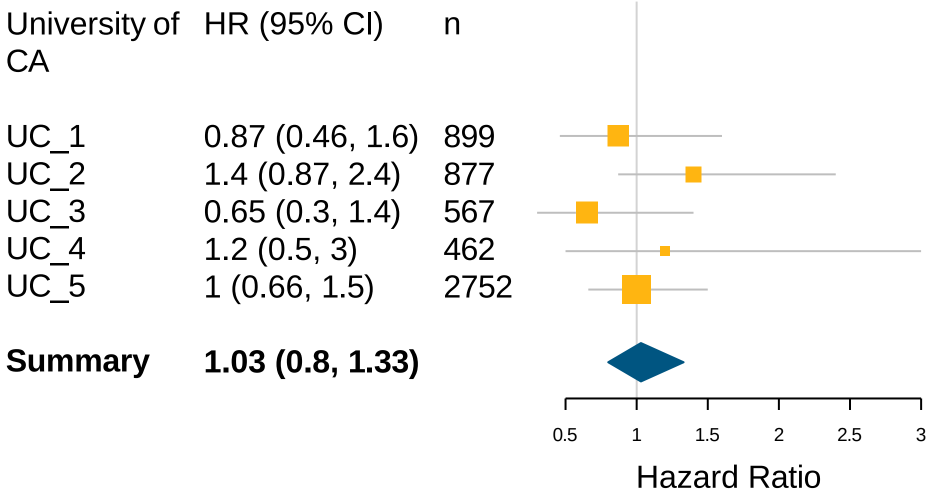

The table below shows the Leave-One-UC-Out diagnostics. The DFFITS value, Cook's distance, Covariance ratio, leave-one-out amount of heteroginity, indicator for influential estimates, comparator and treated groups are provided for each Leave-One-UC-Out analysis. The influential estimate from one UC with respect to pooled estimate are marked as Yes or No, with Yes indicating an influential UC and No otherwise.

eTable 25: Leave-One-UC-Out Sensitivity Analysis

DFFITs	Cook's Dist	Residual Heterogeneity	Influential	Comparator	Treated	UC
-0.2611679	0.0682087	0.0000000	No	DPP4i	SGLT2i	UC_1
0.8001890	0.6403024	0.0000000	Yes	DPP4i	SGLT2i	UC_2
-0.4363055	0.1903625	0.0000000	No	DPP4i	SGLT2i	UC_3
0.1109680	0.0124222	0.0018378	No	DPP4i	SGLT2i	UC_4
-0.0994642	0.0113083	0.0062747	No	DPP4i	SGLT2i	UC_5

© 2023 Vashisht R et al. *JAMA Network Open.*

The forest plot illustrate the effect size of the comparison between GLP1ra and SGLT2i at each UC along with the effect size obtained from the random effect meta-analysis across all the UC for outcome Cardiovascular Disease

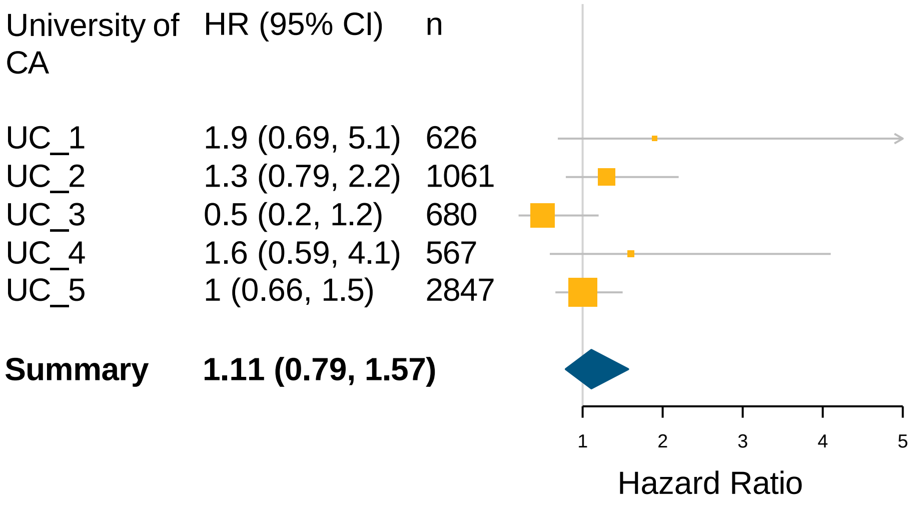

The table below shows the Leave-One-UC-Out diagnostics. The DFFITS value, Cook's distance, Covariance ratio, leave-one-out amount of heteroginity, indicator for influential estimates, comparator and treated groups are provided for each Leave-One-UC-Out analysis. The influential estimate from one UC with respect to pooled estimate are marked as Yes or No, with Yes indicating an influential UC and No otherwise.

eTable 26: Leave-One-UC-Out Sensitivity Analysis

DFFITs	Cook's Dist	Residual Heterogeneity	Influential	Comparator	Treated	UC
0.3495178	0.1215200	0.0365311	No	GLP1ra	SGLT2i	UC_1
0.2406497	0.0864925	0.0905940	No	GLP1ra	SGLT2i	UC_2
-0.4628002	0.1811490	0.0000000	No	GLP1ra	SGLT2i	UC_3
0.2534010	0.0686971	0.0578463	No	GLP1ra	SGLT2i	UC_4
-0.2443169	0.1113249	0.1090039	No	GLP1ra	SGLT2i	UC_5

The forest plot illustrate the effect size of the comparison between Sulfonylurea and DPP4i at each UC along with the effect size obtained from the random effect meta-analysis across all the UC for outcome Cardiovascular Disease

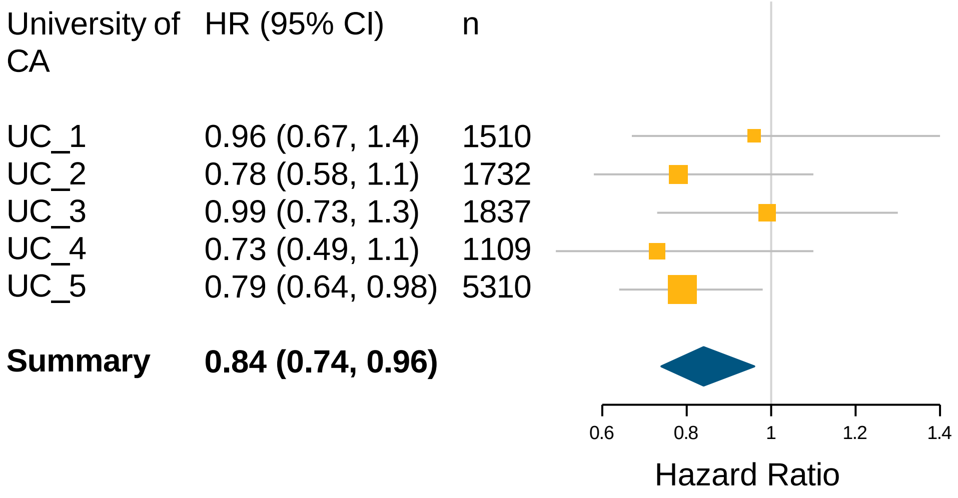

The table below shows the Leave-One-UC-Out diagnostics. The DFFITS value, Cook's distance, Covariance ratio, leave-one-out amount of heteroginity, indicator for influential estimates, comparator and treated groups are provided for each Leave-One-UC-Out analysis. The influential estimate from one UC with respect to pooled estimate are marked as Yes or No, with Yes indicating an influential UC and No otherwise.

eTable 27: Leave-One-UC-Out Sensitivity Analysis

DFFITs	Cook's Dist	Residual Heterogeneity	Influential	Comparator	Treated	UC
0.2913696	0.0848962	0	No	Sulfonylurea	DPP4i	UC_1
-0.2270844	0.0515673	0	No	Sulfonylurea	DPP4i	UC_2
0.6450346	0.4160697	0	No	Sulfonylurea	DPP4i	UC_3
-0.2495914	0.0622959	0	No	Sulfonylurea	DPP4i	UC_4
-0.5725926	0.3278623	0	No	Sulfonylurea	DPP4i	UC_5

© 2023 Vashisht R et al. *JAMA Network Open.*

The forest plot illustrate the effect size of the comparison between Sulfonylurea and GLP1ra at each UC along with the effect size obtained from the random effect meta-analysis across all the UC for outcome Cardiovascular Disease

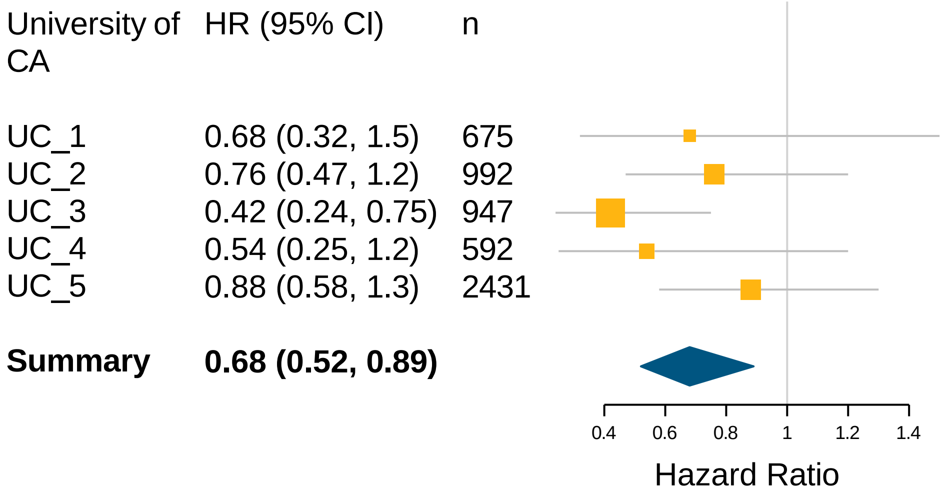

The table below shows the Leave-One-UC-Out diagnostics. The DFFITS value, Cook's distance, Covariance ratio, leave-one-out amount of heteroginity, indicator for influential estimates, comparator and treated groups are provided for each Leave-One-UC-Out analysis. The influential estimate from one UC with respect to pooled estimate are marked as Yes or No, with Yes indicating an influential UC and No otherwise.

eTable 28: Leave-One-UC-Out Sensitivity Analysis

DFFITs	Cook's Dist	Residual Heterogeneity	Influential	Comparator	Treated	UC
0.1283421	0.0190643	0.0445401	No	Sulfonylurea	GLP1ra	UC_1
0.3799653	0.2092026	0.0508302	No	Sulfonylurea	GLP1ra	UC_2
-0.9985335	0.8271325	0.0000000	Yes	Sulfonylurea	GLP1ra	UC_3
-0.1237666	0.0167982	0.0345080	No	Sulfonylurea	GLP1ra	UC_4
1.0161774	0.7326161	0.0000000	Yes	Sulfonylurea	GLP1ra	UC_5

© 2023 Vashisht R et al. *JAMA Network Open.*

The forest plot illustrate the effect size of the comparison between Sulfonylurea and SGLT2i at each UC along with the effect size obtained from the random effect meta-analysis across all the UC for outcome Cardiovascular Disease

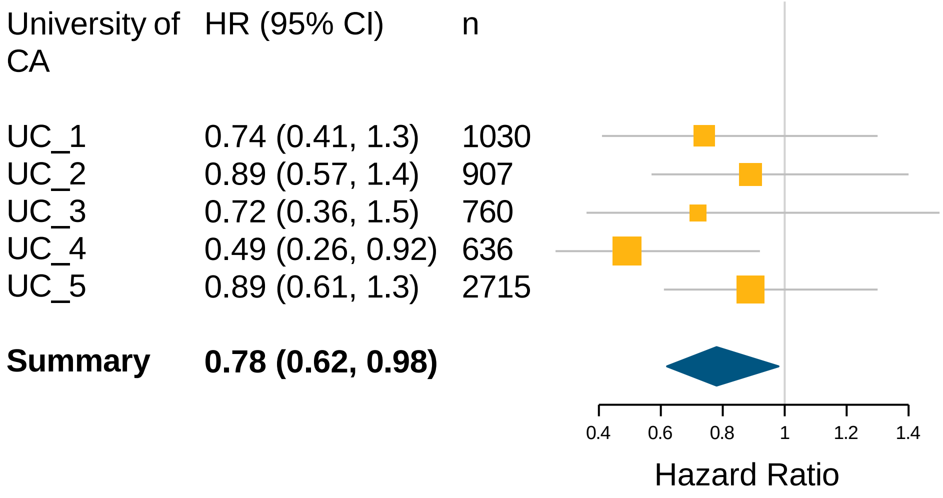

The table below shows the Leave-One-UC-Out diagnostics. The DFFITS value, Cook's distance, Covariance ratio, leave-one-out amount of heteroginity, indicator for influential estimates, comparator and treated groups are provided for each Leave-One-UC-Out analysis. The influential estimate from one UC with respect to pooled estimate are marked as Yes or No, with Yes indicating an influential UC and No otherwise.

eTable 29: Leave-One-UC-Out Sensitivity Analysis

DFFITs	Cook's Dist	Residual Heterogeneity	Influential	Comparator	Treated	UC
-0.0909932	0.0082798	0	No	Sulfonylurea	SGLT2i	UC_1
0.3757752	0.1412070	0	No	Sulfonylurea	SGLT2i	UC_2
-0.0825804	0.0068195	0	No	Sulfonylurea	SGLT2i	UC_3
-0.6010275	0.3612340	0	No	Sulfonylurea	SGLT2i	UC_4
0.6166602	0.3802698	0	No	Sulfonylurea	SGLT2i	UC_5

© 2023 Vashisht R et al. *JAMA Network Open.*

4.5 Cataract

4.5.1 eTable: Drug comparision table

Effect size of each drug comparison at each UC health site is tabulated.

eTable 30: Hazard ratios of drug class comparison at each UC

Comparator	Treated	UC	N	Hazard Ratio (95% CI)	P-value	Adjusted P-Value
DPP4i	GLP1ra	UC_1	548	1.1 (0.64-2)	6.95430e-01	8.692875e-01
DPP4i	GLP1ra	UC_2	987	1 (0.67-1.5)	9.60363e-01	9.731910e-01
DPP4i	GLP1ra	UC_3	735	0.77 (0.47-1.3)	3.08990e-01	7.865700e-01
DPP4i	GLP1ra	UC_4	511	0.78 (0.31-2)	6.01405e-01	8.692875e-01
DPP4i	GLP1ra	UC_5	2370	0.79 (0.6-1)	8.65314e-02	3.244927e-01
DPP4i	SGLT2i	UC_1	858	0.98 (0.65-1.5)	9.26697e-01	9.731910e-01
DPP4i	SGLT2i	UC_2	982	0.82 (0.55-1.2)	3.45887e-01	7.982008e-01
DPP4i	SGLT2i	UC_3	579	1.1 (0.58-2)	8.00197e-01	9.602364e-01
DPP4i	SGLT2i	UC_4	494	2.6 (1-6.6)	5.00603e-02	2.804687e-01
DPP4i	SGLT2i	UC_5	2579	0.88 (0.68-1.1)	3.14628e-01	7.865700e-01
GLP1ra	SGLT2i	UC_1	592	1.3 (0.78-2.2)	3.09981e-01	7.865700e-01
GLP1ra	SGLT2i	UC_2	1155	0.91 (0.61-1.4)	6.52153e-01	8.692875e-01
GLP1ra	SGLT2i	UC_3	700	0.99 (0.58-1.7)	9.73191e-01	9.731910e-01
GLP1ra	SGLT2i	UC_4	586	1.6 (0.68-3.7)	2.81835e-01	7.865700e-01
GLP1ra	SGLT2i	UC_5	2712	1 (0.76-1.3)	9.22539e-01	9.731910e-01
Sulfonylurea	DPP4i	UC_1	1393	0.87 (0.64-1.2)	3.79210e-01	8.018044e-01
Sulfonylurea	DPP4i	UC_2	1938	1.1 (0.81-1.4)	6.71755e-01	8.692875e-01
Sulfonylurea	DPP4i	UC_3	1777	1.1 (0.86-1.4)	4.10517e-01	8.018044e-01
Sulfonylurea	DPP4i	UC_4	1168	0.54 (0.36-0.83)	4.51175e-03	5.150680e-02
Sulfonylurea	DPP4i	UC_5	4972	0.79 (0.68-0.91)	1.56539e-03	4.696170e-02
Sulfonylurea	GLP1ra	UC_1	626	1.1 (0.66-1.9)	6.89783e-01	8.692875e-01
Sulfonylurea	GLP1ra	UC_2	1075	1.1 (0.74-1.6)	6.54339e-01	8.692875e-01
Sulfonylurea	GLP1ra	UC_3	949	0.83 (0.52-1.3)	4.27629e-01	8.018044e-01
Sulfonylurea	GLP1ra	UC_4	589	0.45 (0.21-0.97)	4.03739e-02	2.804687e-01
Sulfonylurea	GLP1ra	UC_5	2323	0.68 (0.52-0.89)	5.15068e-03	5.150680e-02
Sulfonylurea	SGLT2i	UC_1	1037	1 (0.69-1.5)	9.31701e-01	9.731910e-01
Sulfonylurea	SGLT2i	UC_2	1038	0.67 (0.44-1)	6.33968e-02	2.804687e-01
Sulfonylurea	SGLT2i	UC_3	805	1.2 (0.72-2.1)	4.73515e-01	8.356147e-01
Sulfonylurea	SGLT2i	UC_4	654	0.83 (0.43-1.6)	5.94680e-01	8.692875e-01
Sulfonylurea	SGLT2i	UC_5	2614	0.8 (0.62-1)	6.54427e-02	2.804687e-01

4.5.2 eFigure: Individual effect size, meta analysis and sensitivity analysis

The forest plot illustrate the effect size of the comparison between DPP4i and GLP1ra at each UC along with the effect size obtained from the random effect meta-analysis across all the UC for outcome Cataract

© 2023 Vashisht R et al. *JAMA Network Open.*

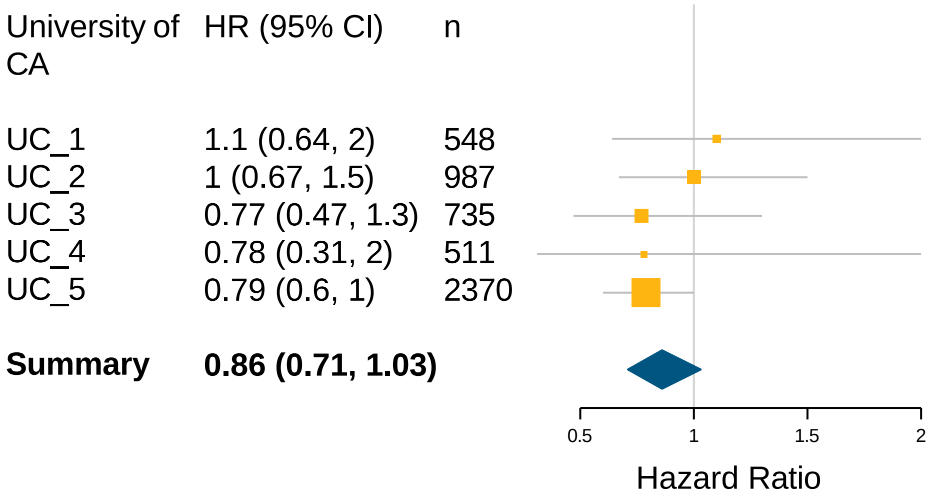

The table below shows the Leave-One-UC-Out diagnostics. The DFFITS value, Cook's distance, Covariance ratio, leave-one-out amount of heteroginity, indicator for influential estimates, comparator and treated groups are provided for each Leave-One-UC-Out analysis. The influential estimate from one UC with respect to pooled estimate are marked as Yes or No, with Yes indicating an influential UC and No otherwise.

eTable 31: Leave-One-UC-Out Sensitivity Analysis

DFFITs	Cook's Dist	Residual Heterogeneity	Influential	Comparator	Treated	UC
0.3114716	0.0970146	0	No	DPP4i	GLP1ra	UC_1
0.4372512	0.1911886	0	No	DPP4i	GLP1ra	UC_2
-0.1687343	0.0284712	0	No	DPP4i	GLP1ra	UC_3
-0.0398858	0.0015909	0	No	DPP4i	GLP1ra	UC_4
-0.9136671	0.8347875	0	Yes	DPP4i	GLP1ra	UC_5

© 2023 Vashisht R et al. *JAMA Network Open.*

The forest plot illustrate the effect size of the comparison between DPP4i and SGLT2i at each UC along with the effect size obtained from the random effect meta-analysis across all the UC for outcome Cataract

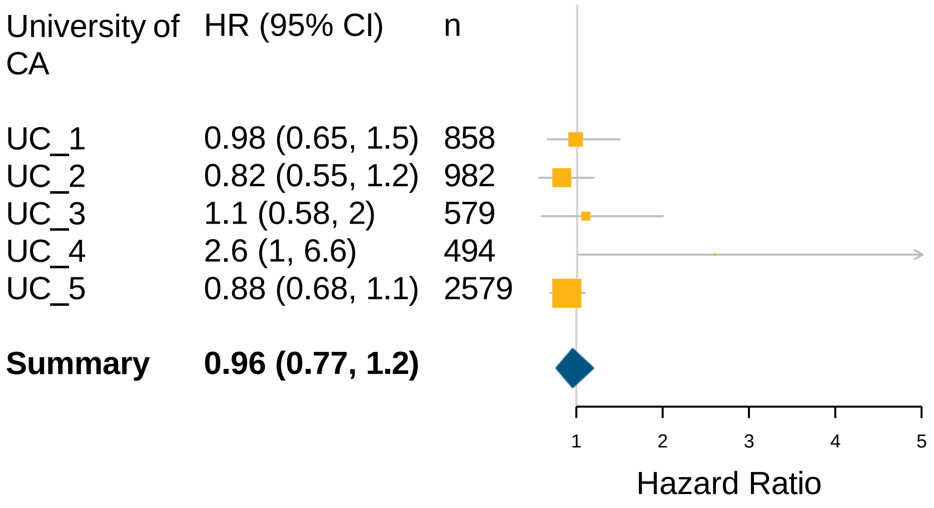

The table below shows the Leave-One-UC-Out diagnostics. The DFFITS value, Cook's distance, Covariance ratio, leave-one-out amount of heteroginity, indicator for influential estimates, comparator and treated groups are provided for each Leave-One-UC-Out analysis. The influential estimate from one UC with respect to pooled estimate are marked as Yes or No, with Yes indicating an influential UC and No otherwise.

eTable 32: Leave-One-UC-Out Sensitivity Analysis

DFFITs	Cook's Dist	Residual Heterogeneity	Influential	Comparator	Treated	UC
-0.2045377	0.0581054	0.0426367	No	DPP4i	SGLT2i	UC_1
-0.5736848	0.4363831	0.0367175	No	DPP4i	SGLT2i	UC_2
0.0185652	0.0003834	0.0311769	No	DPP4i	SGLT2i	UC_3
0.5923897	0.3256922	0.0000000	No	DPP4i	SGLT2i	UC_4
-0.5568468	0.6020519	0.0490378	Yes	DPP4i	SGLT2i	UC_5

The forest plot illustrate the effect size of the comparison between GLP1ra and SGLT2i at each UC along with the effect size obtained from the random effect meta-analysis across all the UC for outcome Cataract

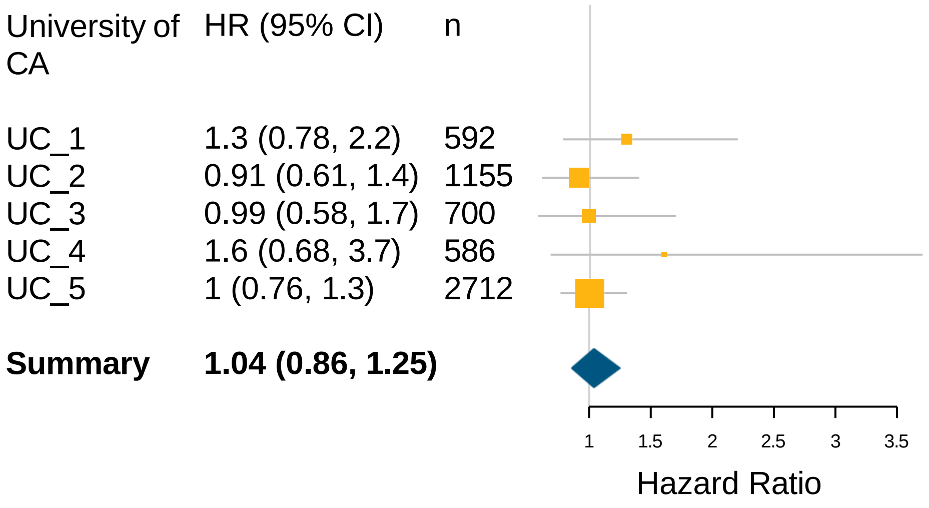

The table below shows the Leave-One-UC-Out diagnostics. The DFFITS value, Cook's distance, Covariance ratio, leave-one-out amount of heteroginity, indicator for influential estimates, comparator and treated groups are provided for each Leave-One-UC-Out analysis. The influential estimate from one UC with respect to pooled estimate are marked as Yes or No, with Yes indicating an influential UC and No otherwise.

eTable 33: Leave-One-UC-Out Sensitivity Analysis

DFFITs	Cook's Dist	Residual Heterogeneity	Influential	Comparator	Treated	UC
0.3557986	0.1265927	0	No	GLP1ra	SGLT2i	UC_1
-0.3535011	0.1249630	0	No	GLP1ra	SGLT2i	UC_2
-0.0686243	0.0047093	0	No	GLP1ra	SGLT2i	UC_3
0.2340329	0.0547714	0	No	GLP1ra	SGLT2i	UC_4
-0.3740037	0.1398788	0	No	GLP1ra	SGLT2i	UC_5

© 2023 Vashisht R et al. *JAMA Network Open.*

The forest plot illustrate the effect size of the comparison between Sulfonylurea and DPP4i at each UC along with the effect size obtained from the random effect meta-analysis across all the UC for outcome Cataract

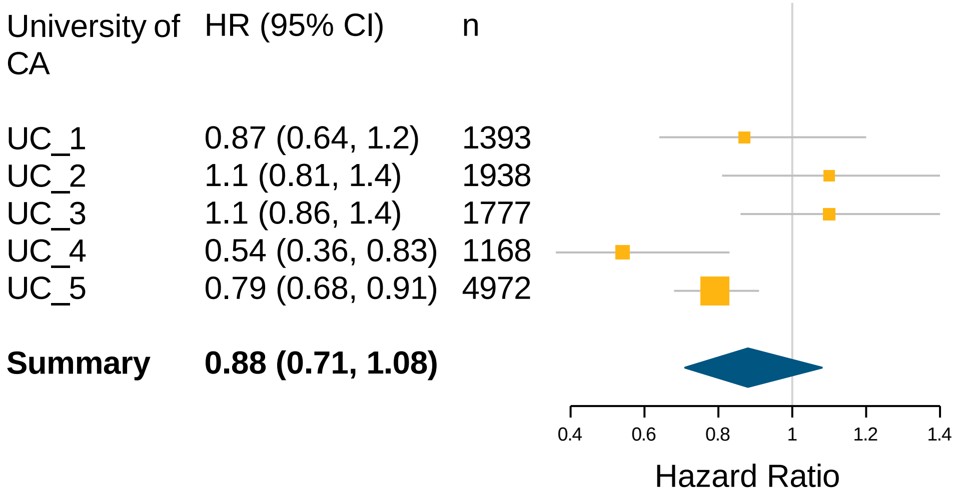

The table below shows the Leave-One-UC-Out diagnostics. The DFFITS value, Cook's distance, Covariance ratio, leave-one-out amount of heteroginity, indicator for influential estimates, comparator and treated groups are provided for each Leave-One-UC-Out analysis. The influential estimate from one UC with respect to pooled estimate are marked as Yes or No, with Yes indicating an influential UC and No otherwise.

eTable 34: Leave-One-UC-Out Sensitivity Analysis

DFFITs	Cook's Dist	Residual Heterogeneity	Influential	Comparator	Treated	UC
0.0254604	0.0007972	0.0520790	No	Sulfonylurea	DPP4i	UC_1
0.5353689	0.2860232	0.0374200	No	Sulfonylurea	DPP4i	UC_2
0.5967439	0.3330493	0.0341080	No	Sulfonylurea	DPP4i	UC_3
-0.7423980	0.4505972	0.0224048	No	Sulfonylurea	DPP4i	UC_4
-0.2305193	0.0729767	0.0536148	No	Sulfonylurea	DPP4i	UC_5

© 2023 Vashisht R et al. *JAMA Network Open.*

The forest plot illustrate the effect size of the comparison between Sulfonylurea and GLP1ra at each UC along with the effect size obtained from the random effect meta-analysis across all the UC for outcome Cataract

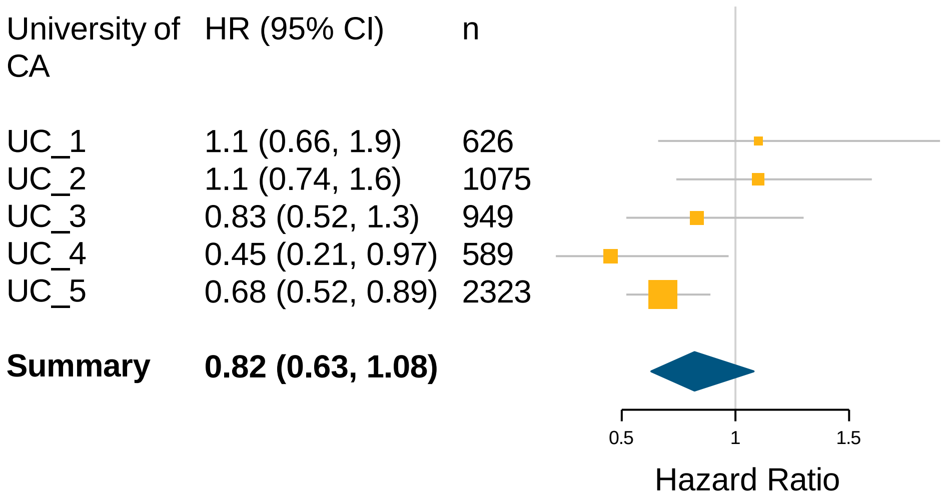

The table below shows the Leave-One-UC-Out diagnostics. The DFFITS value, Cook's distance, Covariance ratio, leave-one-out amount of heteroginity, indicator for influential estimates, comparator and treated groups are provided for each Leave-One-UC-Out analysis. The influential estimate from one UC with respect to pooled estimate are marked as Yes or No, with Yes indicating an influential UC and No otherwise.

eTable 35: Leave-One-UC-Out Sensitivity Analysis

DFFITs	Cook's Dist	Residual Heterogeneity	Influential	Comparator	Treated	UC
0.4147158	0.1766558	0.0466043	No	Sulfonylurea	GLP1ra	UC_1
0.7396815	0.4283972	0.0256243	No	Sulfonylurea	GLP1ra	UC_2
0.0312347	0.0012824	0.0742966	No	Sulfonylurea	GLP1ra	UC_3
-0.4329112	0.1746018	0.0300652	No	Sulfonylurea	GLP1ra	UC_4
-0.6441411	0.3916973	0.0399676	No	Sulfonylurea	GLP1ra	UC_5

© 2023 Vashisht R et al. *JAMA Network Open.*

The forest plot illustrate the effect size of the comparison between Sulfonylurea and SGLT2i at each UC along with the effect size obtained from the random effect meta-analysis across all the UC for outcome Cataract

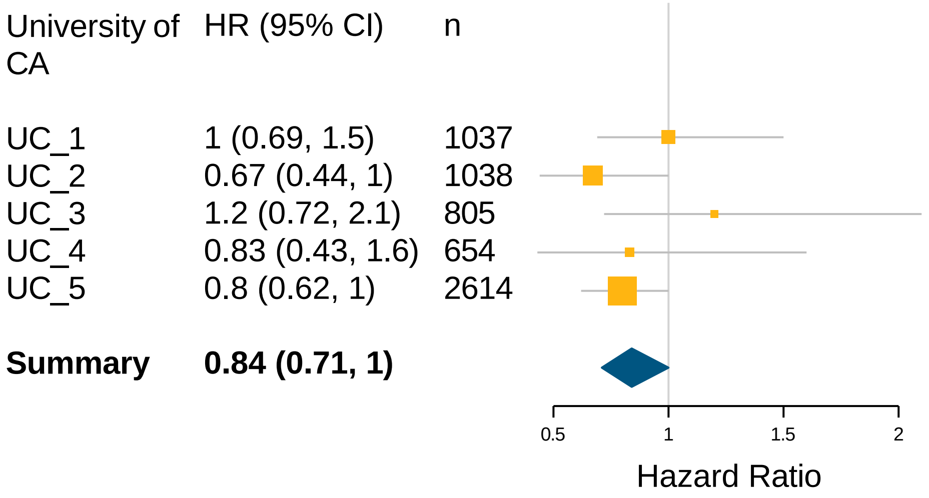

The table below shows the Leave-One-UC-Out diagnostics. The DFFITS value, Cook's distance, Covariance ratio, leave-one-out amount of heteroginity, indicator for influential estimates, comparator and treated groups are provided for each Leave-One-UC-Out analysis. The influential estimate from one UC with respect to pooled estimate are marked as Yes or No, with Yes indicating an influential UC and No otherwise.

eTable 36: Leave-One-UC-Out Sensitivity Analysis

DFFITs	Cook's Dist	Residual Heterogeneity	Influential	Comparator	Treated	UC
0.4510702	0.2034644	0.0000000	No	Sulfonylurea	SGLT2i	UC_1
-0.5376617	0.2890801	0.0000000	No	Sulfonylurea	SGLT2i	UC_2
0.4450423	0.1980627	0.0000000	No	Sulfonylurea	SGLT2i	UC_3
-0.1383975	0.0207922	0.0096108	No	Sulfonylurea	SGLT2i	UC_4
-0.5015277	0.3942573	0.0084388	No	Sulfonylurea	SGLT2i	UC_5

© 2023 Vashisht R et al. *JAMA Network Open.*

4.6 Cerebrovascular Diseases

4.6.1 eTable: Drug comparision table

Effect size of each drug comparison at each UC health site is tabulated.

eTable 37: Hazard ratios of drug class comparison at each UC

Comparator	Treated	UC	N	Hazard Ratio (95% CI)	P-value	Adjusted P-Value
DPP4i	GLP1ra	UC_1	678	3.8 (0.78-18)	9.91911e-02	7.439332e-01
DPP4i	GLP1ra	UC_2	1052	0.89 (0.39-2.1)	7.95583e-01	9.887930e-01
DPP4i	GLP1ra	UC_3	825	1.1 (0.43-2.5)	9.13107e-01	9.887930e-01
DPP4i	GLP1ra	UC_4	537	0.69 (0.19-2.4)	5.63397e-01	9.540240e-01
DPP4i	GLP1ra	UC_5	2615	0.6 (0.36-1)	4.81081e-02	7.439332e-01
DPP4i	SGLT2i	UC_1	1006	1.5 (0.65-3.3)	3.56915e-01	9.522725e-01
DPP4i	SGLT2i	UC_2	1022	1 (0.44-2.3)	9.88793e-01	9.887930e-01
DPP4i	SGLT2i	UC_3	668	0.95 (0.13-6.8)	9.60348e-01	9.887930e-01
DPP4i	SGLT2i	UC_4	528	2 (0.5-8.1)	3.20703e-01	9.522725e-01
DPP4i	SGLT2i	UC_5	2912	0.75 (0.47-1.2)	2.29240e-01	9.522725e-01
GLP1ra	SGLT2i	UC_1	684	1.4 (0.4-5)	5.93836e-01	9.540240e-01
GLP1ra	SGLT2i	UC_2	1248	0.92 (0.38-2.2)	8.58788e-01	9.887930e-01
GLP1ra	SGLT2i	UC_3	783	0.26 (0.06-1.2)	8.88474e-02	7.439332e-01
GLP1ra	SGLT2i	UC_4	630	2.2 (0.55-8.8)	2.64695e-01	9.522725e-01
GLP1ra	SGLT2i	UC_5	3005	0.89 (0.52-1.5)	6.84521e-01	9.778871e-01
Sulfonylurea	DPP4i	UC_1	1661	1.1 (0.7-1.9)	5.96354e-01	9.540240e-01
Sulfonylurea	DPP4i	UC_2	1977	0.82 (0.53-1.3)	3.80909e-01	9.522725e-01
Sulfonylurea	DPP4i	UC_3	2011	1 (0.65-1.5)	9.83448e-01	9.887930e-01
Sulfonylurea	DPP4i	UC_4	1223	0.84 (0.45-1.6)	5.76467e-01	9.540240e-01
Sulfonylurea	DPP4i	UC_5	5604	0.93 (0.71-1.2)	6.18735e-01	9.540240e-01
Sulfonylurea	GLP1ra	UC_1	724	0.72 (0.28-1.9)	5.11122e-01	9.540240e-01
Sulfonylurea	GLP1ra	UC_2	1132	0.52 (0.21-1.3)	1.50860e-01	7.543000e-01
Sulfonylurea	GLP1ra	UC_3	1028	0.96 (0.44-2.1)	9.23510e-01	9.887930e-01
Sulfonylurea	GLP1ra	UC_4	641	0.58 (0.17-2)	3.80113e-01	9.522725e-01
Sulfonylurea	GLP1ra	UC_5	2549	0.87 (0.5-1.5)	6.36016e-01	9.540240e-01
Sulfonylurea	SGLT2i	UC_1	1154	0.93 (0.46-1.9)	8.29756e-01	9.887930e-01
Sulfonylurea	SGLT2i	UC_2	1082	0.82 (0.39-1.7)	5.89705e-01	9.540240e-01
Sulfonylurea	SGLT2i	UC_3	907	0.31 (0.08-1.1)	7.31855e-02	7.439332e-01
Sulfonylurea	SGLT2i	UC_4	723	1.1 (0.44-2.8)	8.10459e-01	9.887930e-01
Sulfonylurea	SGLT2i	UC_5	2912	0.7 (0.43-1.1)	1.41123e-01	7.543000e-01

4.6.2 eFigure: Individual effect size, meta analysis and sensitivity analysis

The forest plot illustrate the effect size of the comparison between DPP4i and GLP1ra at each UC along with the effect size obtained from the random effect meta-analysis across all the UC for outcome Cerebrovascular Diseases

© 2023 Vashisht R et al. *JAMA Network Open.*

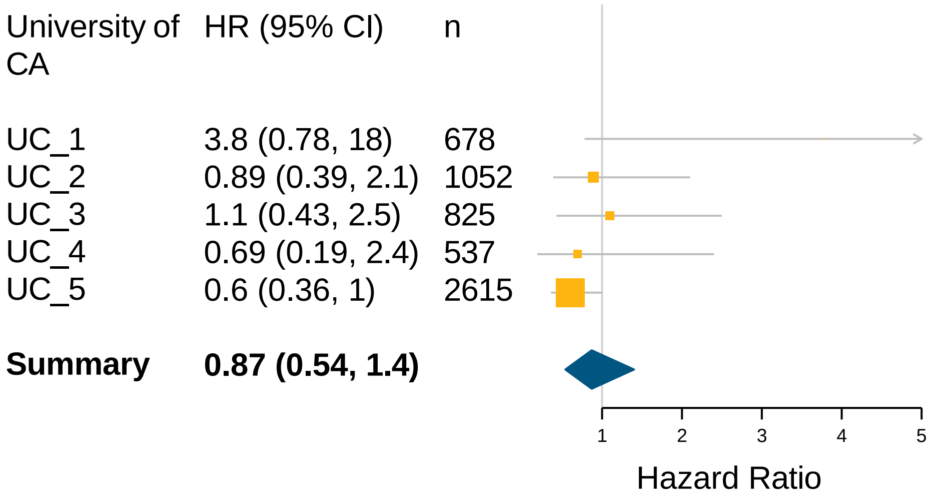

The table below shows the Leave-One-UC-Out diagnostics. The DFFITS value, Cook's distance, Covariance ratio, leave-one-out amount of heteroginity, indicator for influential estimates, comparator and treated groups are provided for each Leave-One-UC-Out analysis. The influential estimate from one UC with respect to pooled estimate are marked as Yes or No, with Yes indicating an influential UC and No otherwise.

eTable 38: Leave-One-UC-Out Sensitivity Analysis

DFFITs	Cook's Dist	Residual Heterogeneity	Influential	Comparator	Treated	UC
0.7666299	0.5200974	0.0000000	Yes	DPP4i	GLP1ra	UC_1
-0.2093884	0.0620779	0.1947549	No	DPP4i	GLP1ra	UC_2
0.0716287	0.0063094	0.1488486	No	DPP4i	GLP1ra	UC_3
-0.3038899	0.1057526	0.1562244	No	DPP4i	GLP1ra	UC_4
-1.2020744	0.7942262	0.0152306	Yes	DPP4i	GLP1ra	UC_5

© 2023 Vashisht R et al. *JAMA Network Open.*

The forest plot illustrate the effect size of the comparison between DPP4i and SGLT2i at each UC along with the effect size obtained from the random effect meta-analysis across all the UC for outcome Cerebrovascular Diseases

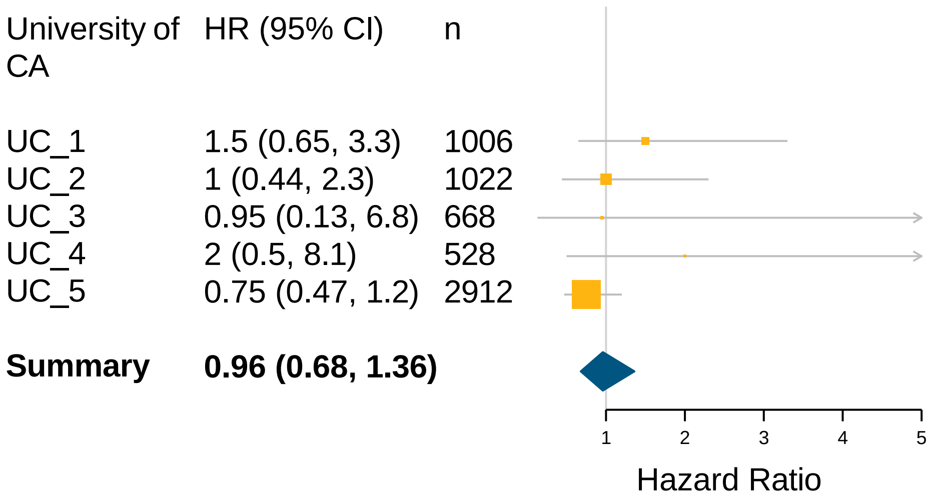

The table below shows the Leave-One-UC-Out diagnostics. The DFFITS value, Cook's distance, Covariance ratio, leave-one-out amount of heteroginity, indicator for influential estimates, comparator and treated groups are provided for each Leave-One-UC-Out analysis. The influential estimate from one UC with respect to pooled estimate are marked as Yes or No, with Yes indicating an influential UC and No otherwise.

eTable 39: Leave-One-UC-Out Sensitivity Analysis

DFFITs	Cook's Dist	Residual Heterogeneity	Influential	Comparator	Treated	UC
0.5645977	0.3187705	0.0000000	No	DPP4i	SGLT2i	UC_1
-0.1545305	0.0268330	0.0220161	No	DPP4i	SGLT2i	UC_2
-0.1426314	0.0206646	0.0160755	No	DPP4i	SGLT2i	UC_3
0.2750849	0.0756717	0.0000000	No	DPP4i	SGLT2i	UC_4
-1.6829891	2.8324523	0.0000000	Yes	DPP4i	SGLT2i	UC_5

© 2023 Vashisht R et al. *JAMA Network Open.*

The forest plot illustrate the effect size of the comparison between GLP1ra and SGLT2i at each UC along with the effect size obtained from the random effect meta-analysis across all the UC for outcome Cerebrovascular Diseases

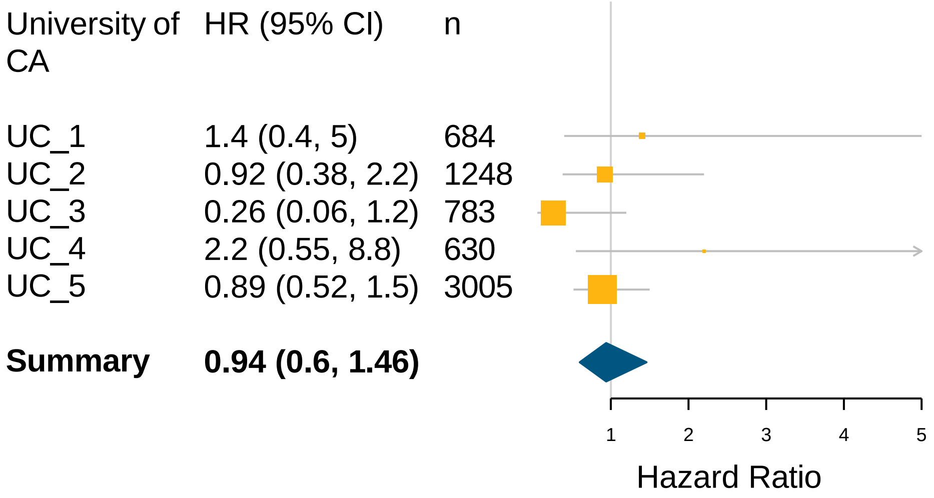

The table below shows the Leave-One-UC-Out diagnostics. The DFFITS value, Cook's distance, Covariance ratio, leave-one-out amount of heteroginity, indicator for influential estimates, comparator and treated groups are provided for each Leave-One-UC-Out analysis. The influential estimate from one UC with respect to pooled estimate are marked as Yes or No, with Yes indicating an influential UC and No otherwise.

eTable 40: Leave-One-UC-Out Sensitivity Analysis

DFFITs	Cook's Dist	Residual Heterogeneity	Influential	Comparator	Treated	UC
0.2350575	0.0617613	0.0874835	No	GLP1ra	SGLT2i	UC_1
-0.0549988	0.0046457	0.1605358	No	GLP1ra	SGLT2i	UC_2
-0.3731886	0.1319097	0.0000000	No	GLP1ra	SGLT2i	UC_3
0.3720031	0.1294567	0.0000000	No	GLP1ra	SGLT2i	UC_4
-0.1116606	0.0309738	0.1941344	No	GLP1ra	SGLT2i	UC_5

© 2023 Vashisht R et al. *JAMA Network Open.*

The forest plot illustrate the effect size of the comparison between Sulfonylurea and DPP4i at each UC along with the effect size obtained from the random effect meta-analysis across all the UC for outcome Cerebrovascular Diseases

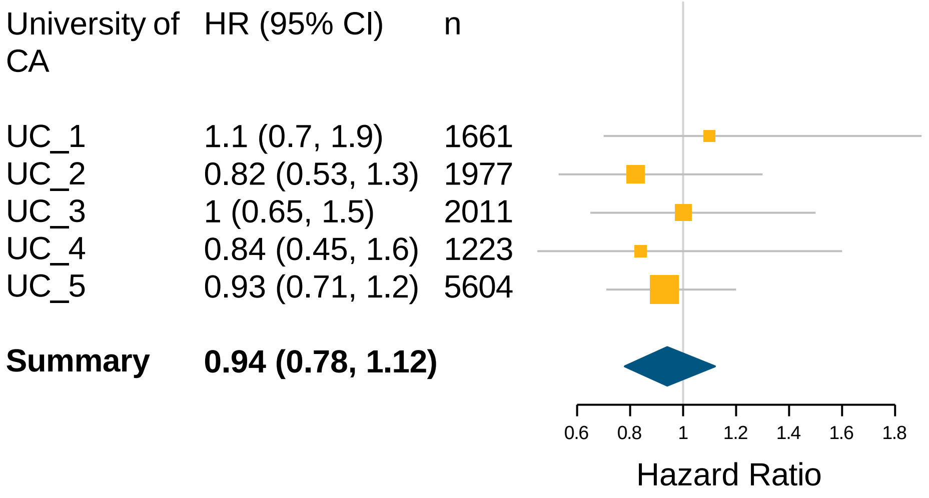

The table below shows the Leave-One-UC-Out diagnostics. The DFFITS value, Cook's distance, Covariance ratio, leave-one-out amount of heteroginity, indicator for influential estimates, comparator and treated groups are provided for each Leave-One-UC-Out analysis. The influential estimate from one UC with respect to pooled estimate are marked as Yes or No, with Yes indicating an influential UC and No otherwise.

eTable 41: Leave-One-UC-Out Sensitivity Analysis

DFFITs	Cook's Dist	Residual Heterogeneity	Influential	Comparator	Treated	UC
0.2577817	0.0664514	0	No	Sulfonylurea	DPP4i	UC_1
-0.2717384	0.0738418	0	No	Sulfonylurea	DPP4i	UC_2
0.1601813	0.0256580	0	No	Sulfonylurea	DPP4i	UC_3
-0.1017771	0.0103586	0	No	Sulfonylurea	DPP4i	UC_4
-0.0618041	0.0038197	0	No	Sulfonylurea	DPP4i	UC_5

© 2023 Vashisht R et al. *JAMA Network Open.*

The forest plot illustrate the effect size of the comparison between Sulfonylurea and GLP1ra at each UC along with the effect size obtained from the random effect meta-analysis across all the UC for outcome Cerebrovascular Diseases

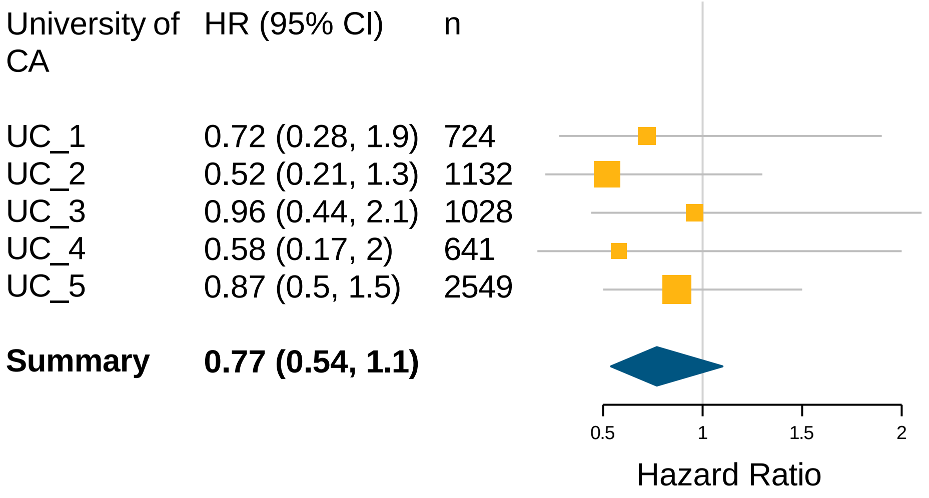

The table below shows the Leave-One-UC-Out diagnostics. The DFFITS value, Cook's distance, Covariance ratio, leave-one-out amount of heteroginity, indicator for influential estimates, comparator and treated groups are provided for each Leave-One-UC-Out analysis. The influential estimate from one UC with respect to pooled estimate are marked as Yes or No, with Yes indicating an influential UC and No otherwise.

eTable 42: Leave-One-UC-Out Sensitivity Analysis

DFFITs	Cook's Dist	Residual Heterogeneity	Influential	Comparator	Treated	UC
-0.0629827	0.0039668	0	No	Sulfonylurea	GLP1ra	UC_1
-0.3927476	0.1542507	0	No	Sulfonylurea	GLP1ra	UC_2
0.3114357	0.0969922	0	No	Sulfonylurea	GLP1ra	UC_3
-0.1439670	0.0207265	0	No	Sulfonylurea	GLP1ra	UC_4
0.4688889	0.2198568	0	No	Sulfonylurea	GLP1ra	UC_5

© 2023 Vashisht R et al. *JAMA Network Open.*

The forest plot illustrate the effect size of the comparison between Sulfonylurea and SGLT2i at each UC along with the effect size obtained from the random effect meta-analysis across all the UC for outcome Cerebrovascular Diseases

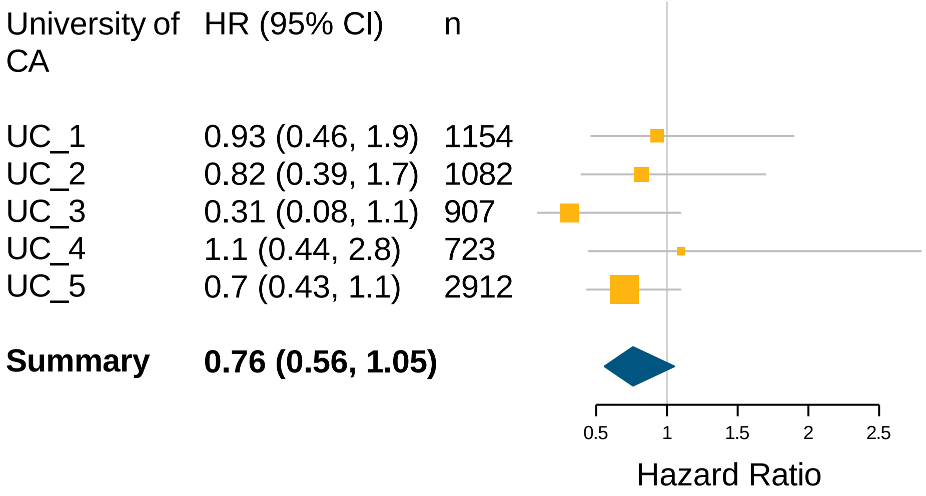

The table below shows the Leave-One-UC-Out diagnostics. The DFFITS value, Cook's distance, Covariance ratio, leave-one-out amount of heteroginity, indicator for influential estimates, comparator and treated groups are provided for each Leave-One-UC-Out analysis. The influential estimate from one UC with respect to pooled estimate are marked as Yes or No, with Yes indicating an influential UC and No otherwise.

eTable 43: Leave-One-UC-Out Sensitivity Analysis

DFFITs	Cook's Dist	Residual Heterogeneity	Influential	Comparator	Treated	UC
0.2985145	0.0891109	0	No	Sulfonylurea	SGLT2i	UC_1
0.0975080	0.0095078	0	No	Sulfonylurea	SGLT2i	UC_2
-0.3571163	0.1275321	0	No	Sulfonylurea	SGLT2i	UC_3
0.2957249	0.0874532	0	No	Sulfonylurea	SGLT2i	UC_4
-0.4441872	0.1973023	0	No	Sulfonylurea	SGLT2i	UC_5

© 2023 Vashisht R et al. *JAMA Network Open.*

4.7 Chronic Kidney Disease

4.7.1 eTable: Drug comparision table

Effect size of each drug comparison at each UC health site is tabulated.

eTable 44: Hazard ratios of drug class comparison at each UC

Comparator	Treated	UC	N	Hazard Ratio (95% CI)	P-value	Adjusted P-Value
DPP4i	GLP1ra	UC_1	656	1 (0.49-2.2)	9.08617e-01	9.399486e-01
DPP4i	GLP1ra	UC_2	972	0.61 (0.36-1)	6.53367e-02	3.387596e-01
DPP4i	GLP1ra	UC_3	792	0.42 (0.23-0.76)	4.00642e-03	1.201926e-01
DPP4i	GLP1ra	UC_4	496	0.81 (0.39-1.7)	5.66788e-01	8.096971e-01
DPP4i	GLP1ra	UC_5	2523	0.93 (0.65-1.3)	6.90191e-01	8.282292e-01
DPP4i	SGLT2i	UC_1	960	0.58 (0.33-1)	5.73904e-02	3.387596e-01
DPP4i	SGLT2i	UC_2	925	0.65 (0.4-1.1)	9.03359e-02	3.387596e-01
DPP4i	SGLT2i	UC_3	639	0.56 (0.29-1.1)	8.48675e-02	3.387596e-01
DPP4i	SGLT2i	UC_4	479	0.86 (0.41-1.8)	6.81560e-01	8.282292e-01
DPP4i	SGLT2i	UC_5	2784	0.85 (0.6-1.2)	3.76754e-01	6.379517e-01
GLP1ra	SGLT2i	UC_1	655	0.71 (0.31-1.6)	4.05214e-01	6.398116e-01
GLP1ra	SGLT2i	UC_2	1133	0.96 (0.55-1.7)	8.95120e-01	9.399486e-01
GLP1ra	SGLT2i	UC_3	747	3.7 (1.2-11)	2.00646e-02	3.009690e-01
GLP1ra	SGLT2i	UC_4	589	0.67 (0.3-1.5)	3.28426e-01	6.379517e-01
GLP1ra	SGLT2i	UC_5	2905	0.74 (0.5-1.1)	1.22041e-01	4.068033e-01
Sulfonylurea	DPP4i	UC_1	1602	0.94 (0.66-1.3)	7.47221e-01	8.517933e-01
Sulfonylurea	DPP4i	UC_2	1822	1.1 (0.85-1.5)	3.70779e-01	6.379517e-01
Sulfonylurea	DPP4i	UC_3	1958	1.1 (0.79-1.4)	6.86304e-01	8.282292e-01
Sulfonylurea	DPP4i	UC_4	1119	0.82 (0.53-1.3)	3.78600e-01	6.379517e-01
Sulfonylurea	DPP4i	UC_5	5215	1 (0.83-1.2)	9.57661e-01	9.576610e-01
Sulfonylurea	GLP1ra	UC_1	704	0.72 (0.37-1.4)	3.37632e-01	6.379517e-01
Sulfonylurea	GLP1ra	UC_2	1054	0.75 (0.44-1.3)	2.91814e-01	6.379517e-01
Sulfonylurea	GLP1ra	UC_3	1007	0.66 (0.37-1.2)	1.50454e-01	4.103291e-01
Sulfonylurea	GLP1ra	UC_4	592	0.54 (0.28-1)	5.98023e-02	3.387596e-01
Sulfonylurea	GLP1ra	UC_5	2427	0.87 (0.61-1.2)	4.50158e-01	6.752370e-01
Sulfonylurea	SGLT2i	UC_1	1126	0.6 (0.33-1.1)	7.94132e-02	3.387596e-01
Sulfonylurea	SGLT2i	UC_2	995	0.8 (0.48-1.3)	3.82771e-01	6.379517e-01
Sulfonylurea	SGLT2i	UC_3	881	0.91 (0.49-1.7)	7.66614e-01	8.517933e-01
Sulfonylurea	SGLT2i	UC_4	662	0.87 (0.47-1.6)	6.68635e-01	8.282292e-01
Sulfonylurea	SGLT2i	UC_5	2768	0.75 (0.52-1.1)	1.36542e-01	4.096260e-01

4.7.2 eFigure: Individual effect size, meta analysis and sensitivity analysis

The forest plot illustrate the effect size of the comparison between DPP4i and GLP1ra at each UC along with the effect size obtained from the random effect meta-analysis across all the UC for outcome Chronic Kidney Disease

© 2023 Vashisht R et al. *JAMA Network Open.*

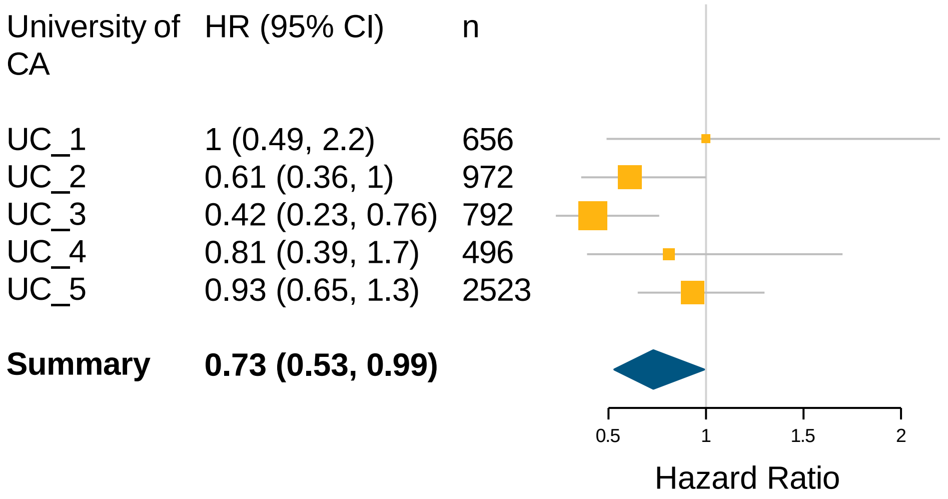

The table below shows the Leave-One-UC-Out diagnostics. The DFFITS value, Cook's distance, Covariance ratio, leave-one-out amount of heteroginity, indicator for influential estimates, comparator and treated groups are provided for each Leave-One-UC-Out analysis. The influential estimate from one UC with respect to pooled estimate are marked as Yes or No, with Yes indicating an influential UC and No otherwise.

eTable 45: Leave-One-UC-Out Sensitivity Analysis

DFFITs	Cook's Dist	Residual Heterogeneity	Influential	Comparator	Treated	UC
0.3403426	0.1265206	0.0634303	No	DPP4i	GLP1ra	UC_1
-0.2403049	0.0714034	0.0725398	No	DPP4i	GLP1ra	UC_2
-1.0559872	0.7477645	0.0000000	Yes	DPP4i	GLP1ra	UC_3
0.1531113	0.0274218	0.0773651	No	DPP4i	GLP1ra	UC_4
0.9524437	0.6524124	0.0240675	Yes	DPP4i	GLP1ra	UC_5

© 2023 Vashisht R et al. *JAMA Network Open.*

The forest plot illustrate the effect size of the comparison between DPP4i and SGLT2i at each UC along with the effect size obtained from the random effect meta-analysis across all the UC for outcome Chronic Kidney Disease

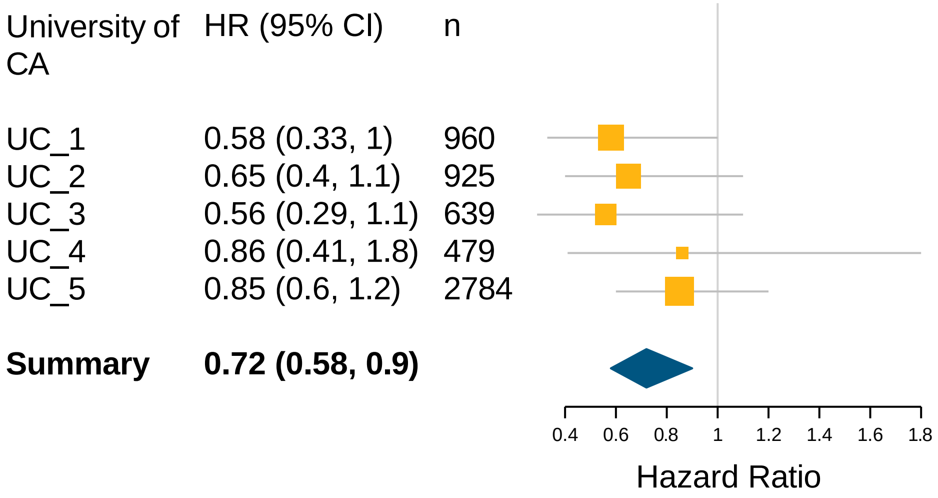

The table below shows the Leave-One-UC-Out diagnostics. The DFFITS value, Cook's distance, Covariance ratio, leave-one-out amount of heteroginity, indicator for influential estimates, comparator and treated groups are provided for each Leave-One-UC-Out analysis. The influential estimate from one UC with respect to pooled estimate are marked as Yes or No, with Yes indicating an influential UC and No otherwise.

eTable 46: Leave-One-UC-Out Sensitivity Analysis

DFFITs	Cook's Dist	Residual Heterogeneity	Influential	Comparator	Treated	UC
-0.3773085	0.1423617	0	No	DPP4i	SGLT2i	UC_1
-0.2255210	0.0508597	0	No	DPP4i	SGLT2i	UC_2
-0.2853383	0.0814179	0	No	DPP4i	SGLT2i	UC_3
0.1569967	0.0246480	0	No	DPP4i	SGLT2i	UC_4
1.0534414	1.1097388	0	Yes	DPP4i	SGLT2i	UC_5

© 2023 Vashisht R et al. *JAMA Network Open.*

The forest plot illustrate the effect size of the comparison between GLP1ra and SGLT2i at each UC along with the effect size obtained from the random effect meta-analysis across all the UC for outcome Chronic Kidney Disease

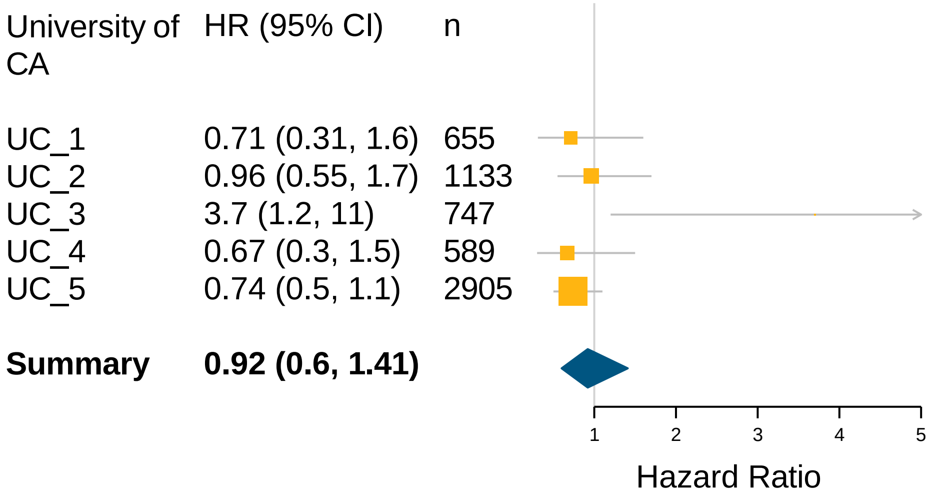

The table below shows the Leave-One-UC-Out diagnostics. The DFFITS value, Cook's distance, Covariance ratio, leave-one-out amount of heteroginity, indicator for influential estimates, comparator and treated groups are provided for each Leave-One-UC-Out analysis. The influential estimate from one UC with respect to pooled estimate are marked as Yes or No, with Yes indicating an influential UC and No otherwise.

eTable 47: Leave-One-UC-Out Sensitivity Analysis

DFFITs	Cook's Dist	Residual Heterogeneity	Influential	Comparator	Treated	UC
-0.3182081	0.1207284	0.1666996	No	GLP1ra	SGLT2i	UC_1
-0.0914097	0.0127770	0.2144112	No	GLP1ra	SGLT2i	UC_2
0.9015203	0.6024062	0.0000000	Yes	GLP1ra	SGLT2i	UC_3
-0.3709628	0.1623931	0.1619804	No	GLP1ra	SGLT2i	UC_4
-0.4314584	0.3127203	0.2148813	No	GLP1ra	SGLT2i	UC_5

© 2023 Vashisht R et al. *JAMA Network Open.*

The forest plot illustrate the effect size of the comparison between Sulfonylurea and DPP4i at each UC along with the effect size obtained from the random effect meta-analysis across all the UC for outcome Chronic Kidney Disease

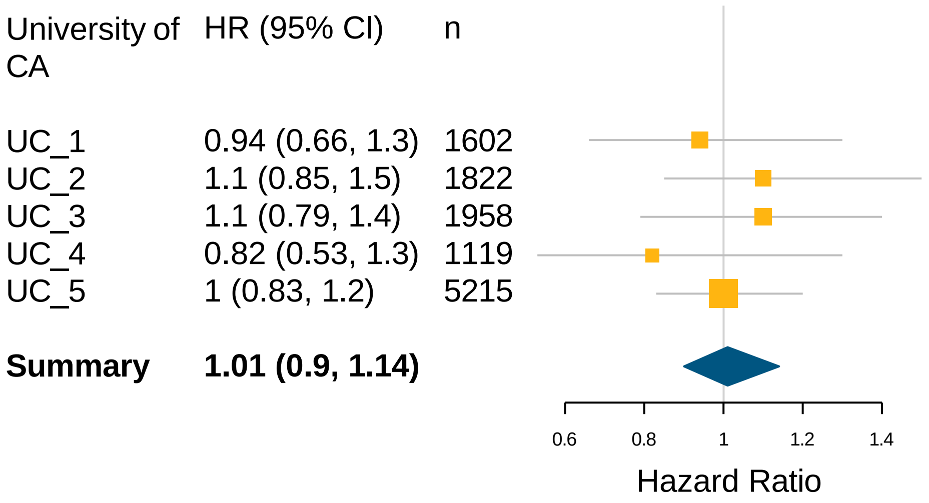

The table below shows the Leave-One-UC-Out diagnostics. The DFFITS value, Cook's distance, Covariance ratio, leave-one-out amount of heteroginity, indicator for influential estimates, comparator and treated groups are provided for each Leave-One-UC-Out analysis. The influential estimate from one UC with respect to pooled estimate are marked as Yes or No, with Yes indicating an influential UC and No otherwise.

eTable 48: Leave-One-UC-Out Sensitivity Analysis

DFFITs	Cook's Dist	Residual Heterogeneity	Influential	Comparator	Treated	UC
-0.1760582	0.0309965	0	No	Sulfonylurea	DPP4i	UC_1
0.3006831	0.0904103	0	No	Sulfonylurea	DPP4i	UC_2
0.2953037	0.0872043	0	No	Sulfonylurea	DPP4i	UC_3
-0.2689283	0.0723224	0	No	Sulfonylurea	DPP4i	UC_4
-0.1504027	0.0226210	0	No	Sulfonylurea	DPP4i	UC_5

© 2023 Vashisht R et al. *JAMA Network Open.*

The forest plot illustrate the effect size of the comparison between Sulfonylurea and GLP1ra at each UC along with the effect size obtained from the random effect meta-analysis across all the UC for outcome Chronic Kidney Disease

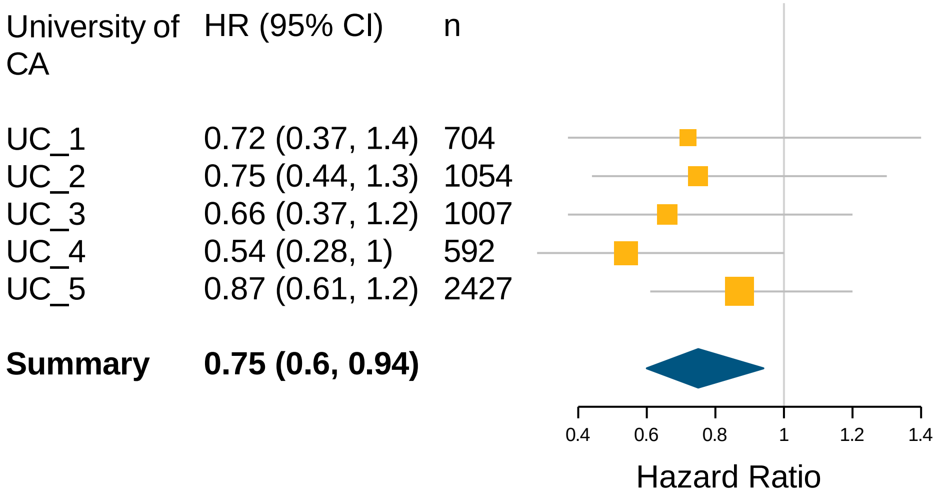

The table below shows the Leave-One-UC-Out diagnostics. The DFFITS value, Cook's distance, Covariance ratio, leave-one-out amount of heteroginity, indicator for influential estimates, comparator and treated groups are provided for each Leave-One-UC-Out analysis. The influential estimate from one UC with respect to pooled estimate are marked as Yes or No, with Yes indicating an influential UC and No otherwise.

eTable 49: Leave-One-UC-Out Sensitivity Analysis

DFFITs	Cook's Dist	Residual Heterogeneity	Influential	Comparator	Treated	UC
-0.0472832	0.0022357	0	No	Sulfonylurea	GLP1ra	UC_1
-0.0022322	0.0000050	0	No	Sulfonylurea	GLP1ra	UC_2
-0.1925764	0.0370857	0	No	Sulfonylurea	GLP1ra	UC_3
-0.4100777	0.1681638	0	No	Sulfonylurea	GLP1ra	UC_4
1.0163441	1.0329553	0	Yes	Sulfonylurea	GLP1ra	UC_5

© 2023 Vashisht R et al. *JAMA Network Open.*

The forest plot illustrate the effect size of the comparison between Sulfonylurea and SGLT2i at each UC along with the effect size obtained from the random effect meta-analysis across all the UC for outcome Chronic Kidney Disease

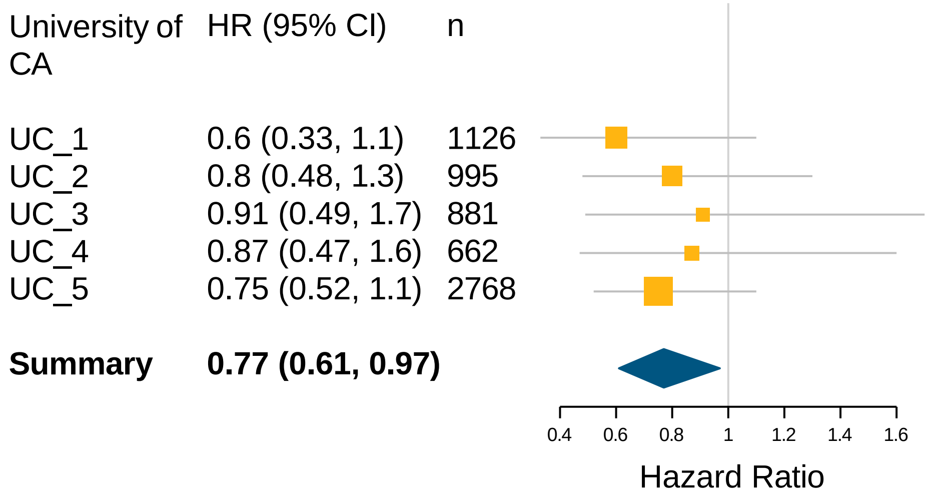

The table below shows the Leave-One-UC-Out diagnostics. The DFFITS value, Cook's distance, Covariance ratio, leave-one-out amount of heteroginity, indicator for influential estimates, comparator and treated groups are provided for each Leave-One-UC-Out analysis. The influential estimate from one UC with respect to pooled estimate are marked as Yes or No, with Yes indicating an influential UC and No otherwise.

eTable 50: Leave-One-UC-Out Sensitivity Analysis

DFFITs	Cook's Dist	Residual Heterogeneity	Influential	Comparator	Treated	UC
-0.3626976	0.1315496	0	No	Sulfonylurea	SGLT2i	UC_1
0.0830938	0.0069046	0	No	Sulfonylurea	SGLT2i	UC_2
0.2209952	0.0488389	0	No	Sulfonylurea	SGLT2i	UC_3
0.1666910	0.0277859	0	No	Sulfonylurea	SGLT2i	UC_4
-0.1430213	0.0204551	0	No	Sulfonylurea	SGLT2i	UC_5

© 2023 Vashisht R et al. *JAMA Network Open.*

4.8 Chronic Liver Diseases

4.8.1 eTable: Drug comparision table

Effect size of each drug comparison at each UC health site is tabulated.

eTable 51: Hazard ratios of drug class comparison at each UC

Comparator	Treated	UC	N	Hazard Ratio (95% CI)	P-value	Adjusted P-Value
DPP4i	GLP1ra	UC_1	645	0.72 (0.3-1.7)	4.53039e-01	7.787333e-01
DPP4i	GLP1ra	UC_2	923	1.1 (0.65-1.9)	6.71268e-01	8.460950e-01
DPP4i	GLP1ra	UC_3	779	1.1 (0.5-2.2)	8.81629e-01	9.446025e-01
DPP4i	GLP1ra	UC_4	475	0.4 (0.15-1)	5.70407e-02	2.444601e-01
DPP4i	GLP1ra	UC_5	2452	1.5 (0.94-2.5)	8.46212e-02	2.968960e-01
DPP4i	SGLT2i	UC_1	981	0.51 (0.19-1.4)	1.81342e-01	4.945691e-01
DPP4i	SGLT2i	UC_2	959	0.77 (0.42-1.4)	3.86246e-01	7.664213e-01
DPP4i	SGLT2i	UC_3	629	1.1 (0.39-3.4)	8.04590e-01	9.100333e-01
DPP4i	SGLT2i	UC_4	483	0.36 (0.14-0.92)	3.21094e-02	2.219658e-01
DPP4i	SGLT2i	UC_5	2781	0.74 (0.42-1.3)	3.15004e-01	6.750086e-01
GLP1ra	SGLT2i	UC_1	636	0.62 (0.2-1.9)	4.08758e-01	7.664213e-01
GLP1ra	SGLT2i	UC_2	1105	0.61 (0.34-1.1)	8.90688e-02	2.968960e-01
GLP1ra	SGLT2i	UC_3	722	0.89 (0.34-2.3)	8.19030e-01	9.100333e-01
GLP1ra	SGLT2i	UC_4	553	1.4 (0.49-4)	5.33064e-01	8.416800e-01
GLP1ra	SGLT2i	UC_5	2814	0.56 (0.34-0.91)	2.06180e-02	2.219658e-01
Sulfonylurea	DPP4i	UC_1	1627	0.84 (0.52-1.3)	4.67240e-01	7.787333e-01
Sulfonylurea	DPP4i	UC_2	1840	1.1 (0.74-1.7)	6.13949e-01	8.460950e-01
Sulfonylurea	DPP4i	UC_3	1953	0.89 (0.56-1.4)	6.36197e-01	8.460950e-01
Sulfonylurea	DPP4i	UC_4	1115	1.3 (0.77-2.3)	3.01874e-01	6.750086e-01
Sulfonylurea	DPP4i	UC_5	5406	0.95 (0.71-1.3)	7.62551e-01	9.100333e-01
Sulfonylurea	GLP1ra	UC_1	689	0.82 (0.35-1.9)	6.39837e-01	8.460950e-01
Sulfonylurea	GLP1ra	UC_2	995	1.6 (0.89-2.8)	1.20571e-01	3.617130e-01
Sulfonylurea	GLP1ra	UC_3	973	1 (0.5-2)	9.95308e-01	9.953080e-01
Sulfonylurea	GLP1ra	UC_4	562	0.36 (0.15-0.86)	2.07196e-02	2.219658e-01
Sulfonylurea	GLP1ra	UC_5	2362	0.91 (0.59-1.4)	6.76876e-01	8.460950e-01
Sulfonylurea	SGLT2i	UC_1	1126	0.45 (0.2-1)	5.66200e-02	2.444601e-01
Sulfonylurea	SGLT2i	UC_2	1014	0.98 (0.52-1.9)	9.61742e-01	9.949055e-01
Sulfonylurea	SGLT2i	UC_3	878	0.61 (0.28-1.3)	2.18882e-01	5.472050e-01
Sulfonylurea	SGLT2i	UC_4	651	0.38 (0.16-0.9)	2.72838e-02	2.219658e-01
Sulfonylurea	SGLT2i	UC_5	2757	0.57 (0.34-0.97)	3.69943e-02	2.219658e-01

4.8.2 eFigure: Individual effect size, meta analysis and sensitivity analysis

The forest plot illustrate the effect size of the comparison between DPP4i and GLP1ra at each UC along with the effect size obtained from the random effect meta-analysis across all the UC for outcome Chronic Liver Diseases

© 2023 Vashisht R et al. *JAMA Network Open.*

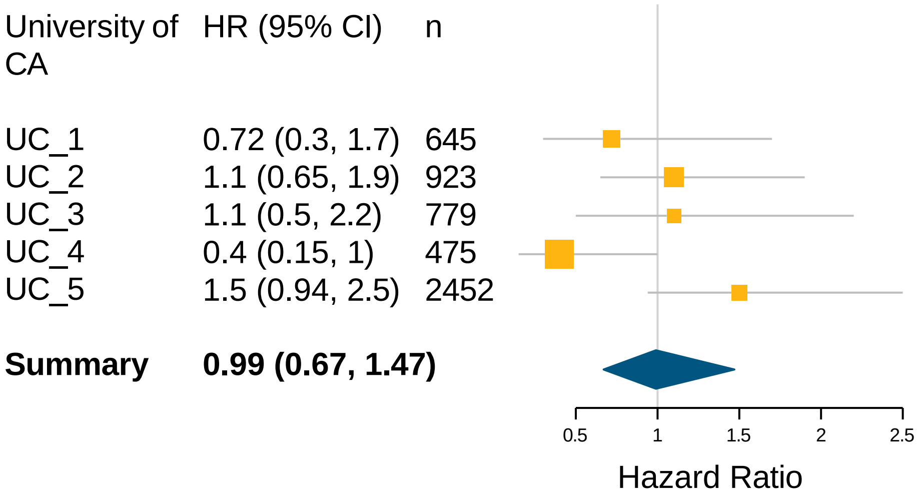

The table below shows the Leave-One-UC-Out diagnostics. The DFFITS value, Cook's distance, Covariance ratio, leave-one-out amount of heteroginity, indicator for influential estimates, comparator and treated groups are provided for each Leave-One-UC-Out analysis. The influential estimate from one UC with respect to pooled estimate are marked as Yes or No, with Yes indicating an influential UC and No otherwise.

eTable 52: Leave-One-UC-Out Sensitivity Analysis

DFFITs	Cook's Dist	Residual Heterogeneity	Influential	Comparator	Treated	UC
-0.2141286	0.0493712	0.1025538	No	DPP4i	GLP1ra	UC_1
0.3130326	0.1562253	0.1740860	No	DPP4i	GLP1ra	UC_2
0.2463747	0.0773894	0.1429050	No	DPP4i	GLP1ra	UC_3
-0.9930293	0.7320665	0.0000000	Yes	DPP4i	GLP1ra	UC_4
0.8449622	0.5204326	0.0423696	Yes	DPP4i	GLP1ra	UC_5

© 2023 Vashisht R et al. *JAMA Network Open.*

The forest plot illustrate the effect size of the comparison between DPP4i and SGLT2i at each UC along with the effect size obtained from the random effect meta-analysis across all the UC for outcome Chronic Liver Diseases

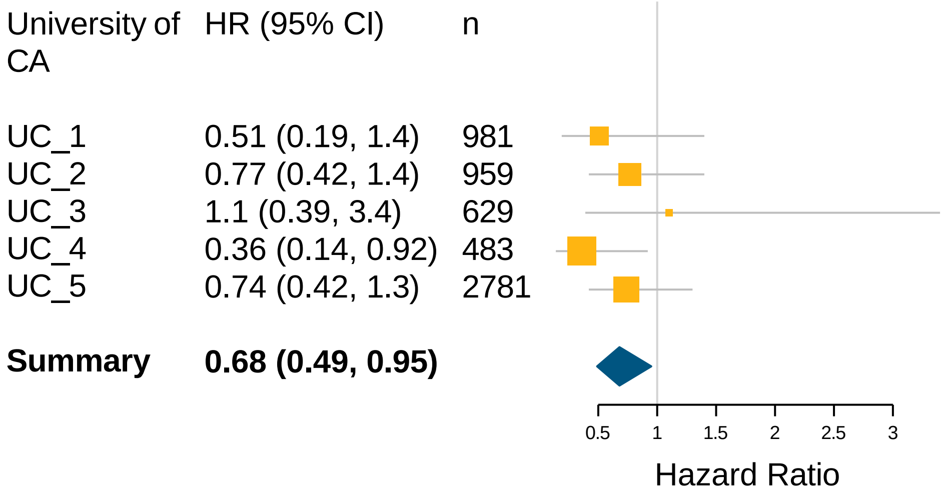

The table below shows the Leave-One-UC-Out diagnostics. The DFFITS value, Cook's distance, Covariance ratio, leave-one-out amount of heteroginity, indicator for influential estimates, comparator and treated groups are provided for each Leave-One-UC-Out analysis. The influential estimate from one UC with respect to pooled estimate are marked as Yes or No, with Yes indicating an influential UC and No otherwise.

eTable 53: Leave-One-UC-Out Sensitivity Analysis

DFFITs	Cook's Dist	Residual Heterogeneity	Influential	Comparator	Treated	UC
-0.2149552	0.0462058	0	No	DPP4i	SGLT2i	UC_1
0.3238228	0.1048612	0	No	DPP4i	SGLT2i	UC_2
0.2977627	0.0886626	0	No	DPP4i	SGLT2i	UC_3
-0.5420225	0.2937883	0	No	DPP4i	SGLT2i	UC_4
0.2650312	0.0702415	0	No	DPP4i	SGLT2i	UC_5

© 2023 Vashisht R et al. *JAMA Network Open.*

The forest plot illustrate the effect size of the comparison between GLP1ra and SGLT2i at each UC along with the effect size obtained from the random effect meta-analysis across all the UC for outcome Chronic Liver Diseases

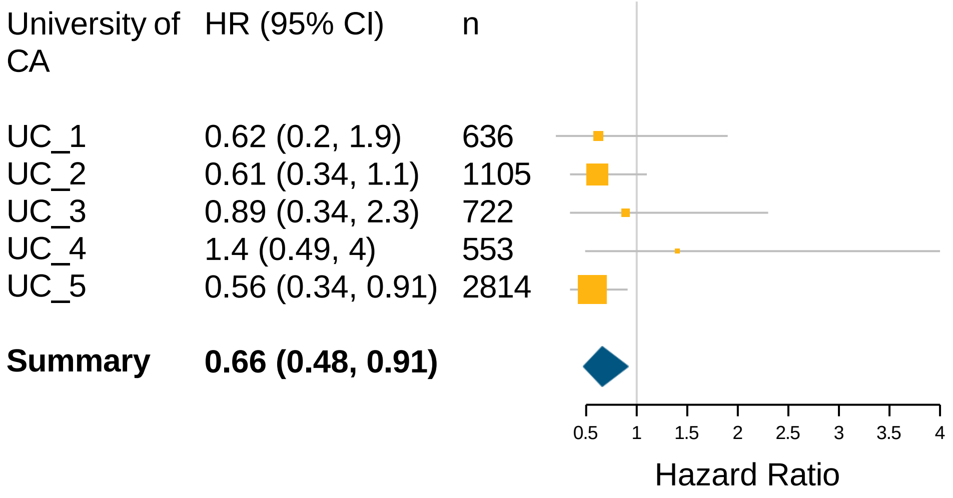

The table below shows the Leave-One-UC-Out diagnostics. The DFFITS value, Cook's distance, Covariance ratio, leave-one-out amount of heteroginity, indicator for influential estimates, comparator and treated groups are provided for each Leave-One-UC-Out analysis. The influential estimate from one UC with respect to pooled estimate are marked as Yes or No, with Yes indicating an influential UC and No otherwise.

eTable 54: Leave-One-UC-Out Sensitivity Analysis

DFFITs	Cook's Dist	Residual Heterogeneity	Influential	Comparator	Treated	UC
-0.0364853	0.0013312	0	No	GLP1ra	SGLT2i	UC_1
-0.2169856	0.0470828	0	No	GLP1ra	SGLT2i	UC_2
0.2261458	0.0511419	0	No	GLP1ra	SGLT2i	UC_3
0.4667955	0.2178980	0	No	GLP1ra	SGLT2i	UC_4
-0.7558470	0.5713047	0	Yes	GLP1ra	SGLT2i	UC_5

© 2023 Vashisht R et al. *JAMA Network Open.*

The forest plot illustrate the effect size of the comparison between Sulfonylurea and DPP4i at each UC along with the effect size obtained from the random effect meta-analysis across all the UC for outcome Chronic Liver Diseases

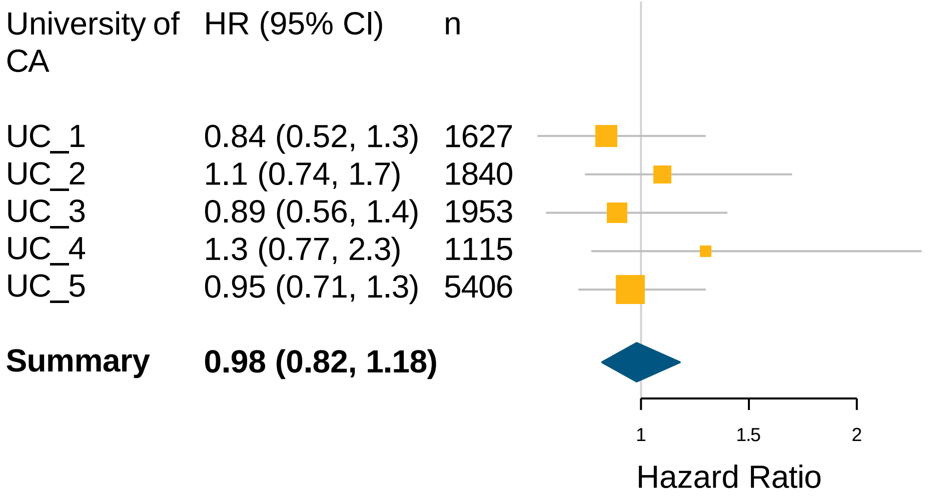

The table below shows the Leave-One-UC-Out diagnostics. The DFFITS value, Cook's distance, Covariance ratio, leave-one-out amount of heteroginity, indicator for influential estimates, comparator and treated groups are provided for each Leave-One-UC-Out analysis. The influential estimate from one UC with respect to pooled estimate are marked as Yes or No, with Yes indicating an influential UC and No otherwise.

eTable 55: Leave-One-UC-Out Sensitivity Analysis

DFFITs	Cook's Dist	Residual Heterogeneity	Influential	Comparator	Treated	UC
-0.3208688	0.1029568	0	No	Sulfonylurea	DPP4i	UC_1
0.2922701	0.0854218	0	No	Sulfonylurea	DPP4i	UC_2
-0.2025534	0.0410279	0	No	Sulfonylurea	DPP4i	UC_3
0.3798206	0.1442637	0	No	Sulfonylurea	DPP4i	UC_4
-0.2108862	0.0444730	0	No	Sulfonylurea	DPP4i	UC_5

© 2023 Vashisht R et al. *JAMA Network Open.*

The forest plot illustrate the effect size of the comparison between Sulfonylurea and GLP1ra at each UC along with the effect size obtained from the random effect meta-analysis across all the UC for outcome Chronic Liver Diseases

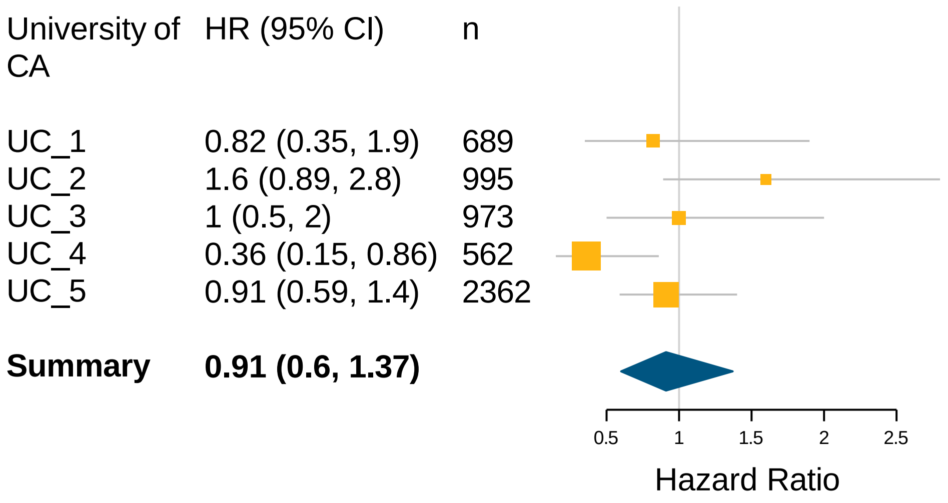

The table below shows the Leave-One-UC-Out diagnostics. The DFFITS value, Cook's distance, Covariance ratio, leave-one-out amount of heteroginity, indicator for influential estimates, comparator and treated groups are provided for each Leave-One-UC-Out analysis. The influential estimate from one UC with respect to pooled estimate are marked as Yes or No, with Yes indicating an influential UC and No otherwise.

eTable 56: Leave-One-UC-Out Sensitivity Analysis

DFFITs	Cook's Dist	Residual Heterogeneity	Influential	Comparator	Treated	UC
-0.0254129	0.0007561	0.1604744	No	Sulfonylurea	GLP1ra	UC_1
0.8393072	0.4444692	0.0377914	No	Sulfonylurea	GLP1ra	UC_2
0.1734786	0.0389859	0.1793178	No	Sulfonylurea	GLP1ra	UC_3
-0.8948316	0.5153481	0.0000000	Yes	Sulfonylurea	GLP1ra	UC_4
0.1096785	0.0213648	0.2328981	No	Sulfonylurea	GLP1ra	UC_5

© 2023 Vashisht R et al. *JAMA Network Open.*

The forest plot illustrate the effect size of the comparison between Sulfonylurea and SGLT2i at each UC along with the effect size obtained from the random effect meta-analysis across all the UC for outcome Chronic Liver Diseases

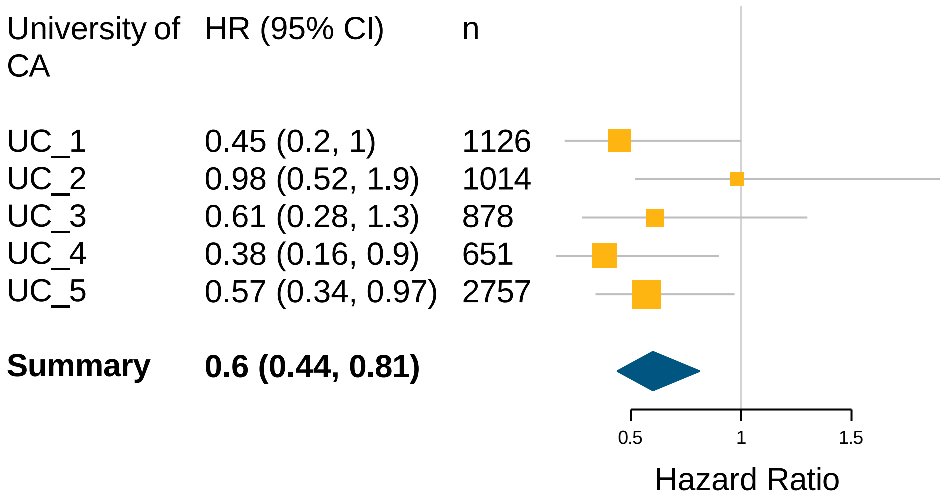

The table below shows the Leave-One-UC-Out diagnostics. The DFFITS value, Cook's distance, Covariance ratio, leave-one-out amount of heteroginity, indicator for influential estimates, comparator and treated groups are provided for each Leave-One-UC-Out analysis. The influential estimate from one UC with respect to pooled estimate are marked as Yes or No, with Yes indicating an influential UC and No otherwise.

eTable 57: Leave-One-UC-Out Sensitivity Analysis

DFFITs	Cook's Dist	Residual Heterogeneity	Influential	Comparator	Treated	UC
-0.2911005	0.0897859	0.0100389	No	Sulfonylurea	SGLT2i	UC_1
0.9154561	0.8380600	0.0000000	Yes	Sulfonylurea	SGLT2i	UC_2
0.0818599	0.0081470	0.0331028	No	Sulfonylurea	SGLT2i	UC_3
-0.4179166	0.1746543	0.0000000	No	Sulfonylurea	SGLT2i	UC_4
-0.0356798	0.0019582	0.0384916	No	Sulfonylurea	SGLT2i	UC_5

© 2023 Vashisht R et al. *JAMA Network Open.*

4.9 Chronic Obstructive Pulmonary Disease

4.9.1 eTable: Drug comparision table

Effect size of each drug comparison at each UC health site is tabulated.

eTable 58: Hazard ratios of drug class comparison at each UC

Comparator	Treated	UC	N	Hazard Ratio (95% CI)	P-value	Adjusted P-Value
DPP4i	GLP1ra	UC_1	686	1.4 (0.31-6.2)	6.76294e-01	9.86142e-01
DPP4i	GLP1ra	UC_2	1032	1 (0.43-2.5)	9.24298e-01	9.86142e-01
DPP4i	GLP1ra	UC_3	820	1 (0.34-3)	9.71702e-01	9.86142e-01
DPP4i	GLP1ra	UC_4	524	0.68 (0.24-1.9)	4.60118e-01	9.86142e-01
DPP4i	GLP1ra	UC_5	2617	1.7 (0.93-3.1)	8.64649e-02	9.86142e-01
DPP4i	SGLT2i	UC_1	1027	1.3 (0.35-4.8)	7.07215e-01	9.86142e-01
DPP4i	SGLT2i	UC_2	1012	0.76 (0.32-1.8)	5.27827e-01	9.86142e-01
DPP4i	SGLT2i	UC_3	667	1.3 (0.3-5.9)	7.11966e-01	9.86142e-01
DPP4i	SGLT2i	UC_4	536	1.2 (0.39-3.4)	7.97457e-01	9.86142e-01
DPP4i	SGLT2i	UC_5	2920	1 (0.56-1.9)	9.30466e-01	9.86142e-01
GLP1ra	SGLT2i	UC_1	693	4 (0.45-36)	2.11707e-01	9.86142e-01
GLP1ra	SGLT2i	UC_2	1199	0.87 (0.32-2.3)	7.78392e-01	9.86142e-01
GLP1ra	SGLT2i	UC_3	781	0.51 (0.13-2)	3.41698e-01	9.86142e-01
GLP1ra	SGLT2i	UC_4	628	1 (0.29-3.5)	9.73430e-01	9.86142e-01
GLP1ra	SGLT2i	UC_5	3017	0.8 (0.45-1.4)	4.32431e-01	9.86142e-01
Sulfonylurea	DPP4i	UC_1	1691	0.82 (0.42-1.6)	5.62771e-01	9.86142e-01
Sulfonylurea	DPP4i	UC_2	1963	1 (0.62-1.7)	9.49702e-01	9.86142e-01
Sulfonylurea	DPP4i	UC_3	2012	0.9 (0.54-1.5)	6.99170e-01	9.86142e-01
Sulfonylurea	DPP4i	UC_4	1218	1 (0.49-2.1)	9.80089e-01	9.86142e-01
Sulfonylurea	DPP4i	UC_5	5614	0.97 (0.69-1.4)	8.45343e-01	9.86142e-01
Sulfonylurea	GLP1ra	UC_1	720	1.2 (0.32-4.5)	7.90539e-01	9.86142e-01
Sulfonylurea	GLP1ra	UC_2	1100	0.61 (0.27-1.4)	2.31881e-01	9.86142e-01
Sulfonylurea	GLP1ra	UC_3	1024	0.56 (0.22-1.4)	2.26137e-01	9.86142e-01
Sulfonylurea	GLP1ra	UC_4	630	1.5 (0.46-4.6)	5.23008e-01	9.86142e-01
Sulfonylurea	GLP1ra	UC_5	2529	1.1 (0.6-1.9)	8.29579e-01	9.86142e-01
Sulfonylurea	SGLT2i	UC_1	1168	0.7 (0.25-2)	5.00307e-01	9.86142e-01
Sulfonylurea	SGLT2i	UC_2	1054	0.83 (0.35-2)	6.78919e-01	9.86142e-01
Sulfonylurea	SGLT2i	UC_3	906	1.5 (0.51-4.2)	4.76615e-01	9.86142e-01
Sulfonylurea	SGLT2i	UC_4	729	1 (0.38-2.7)	9.86142e-01	9.86142e-01
Sulfonylurea	SGLT2i	UC_5	2905	0.98 (0.55-1.7)	9.45827e-01	9.86142e-01

4.9.2 eFigure: Individual effect size, meta analysis and sensitivity analysis

The forest plot illustrate the effect size of the comparison between DPP4i and GLP1ra at each UC along with the effect size obtained from the random effect meta-analysis across all the UC for outcome Chronic Obstructive Pulmonary Disease

© 2023 Vashisht R et al. *JAMA Network Open.*

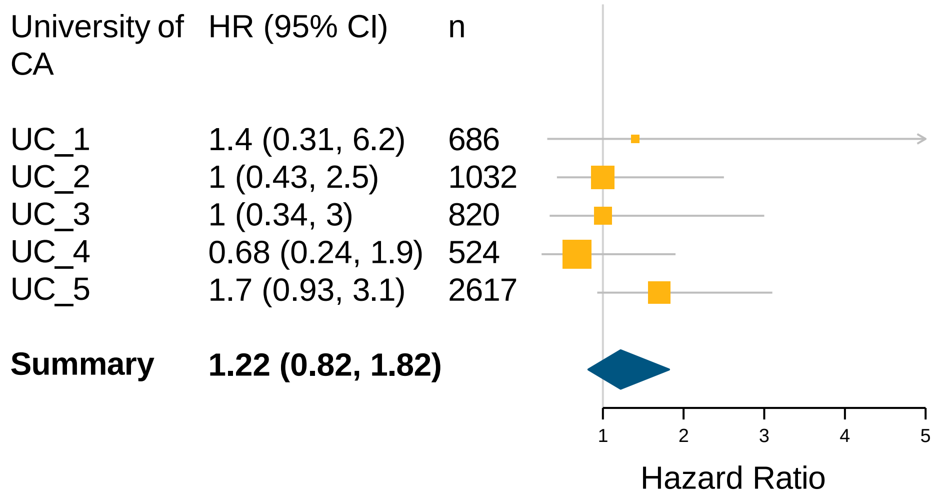

The table below shows the Leave-One-UC-Out diagnostics. The DFFITS value, Cook's distance, Covariance ratio, leave-one-out amount of heteroginity, indicator for influential estimates, comparator and treated groups are provided for each Leave-One-UC-Out analysis. The influential estimate from one UC with respect to pooled estimate are marked as Yes or No, with Yes indicating an influential UC and No otherwise.

eTable 59: Leave-One-UC-Out Sensitivity Analysis

DFFITs	Cook's Dist	Residual Heterogeneity	Influential	Comparator	Treated	UC
0.0512951	0.0026312	0	No	DPP4i	GLP1ra	UC_1
-0.2541562	0.0645954	0	No	DPP4i	GLP1ra	UC_2
-0.1524199	0.0232318	0	No	DPP4i	GLP1ra	UC_3
-0.5030187	0.2530278	0	No	DPP4i	GLP1ra	UC_4
1.2749951	1.6256124	0	Yes	DPP4i	GLP1ra	UC_5

© 2023 Vashisht R et al. *JAMA Network Open.*

The forest plot illustrate the effect size of the comparison between DPP4i and SGLT2i at each UC along with the effect size obtained from the random effect meta-analysis across all the UC for outcome Chronic Obstructive Pulmonary Disease

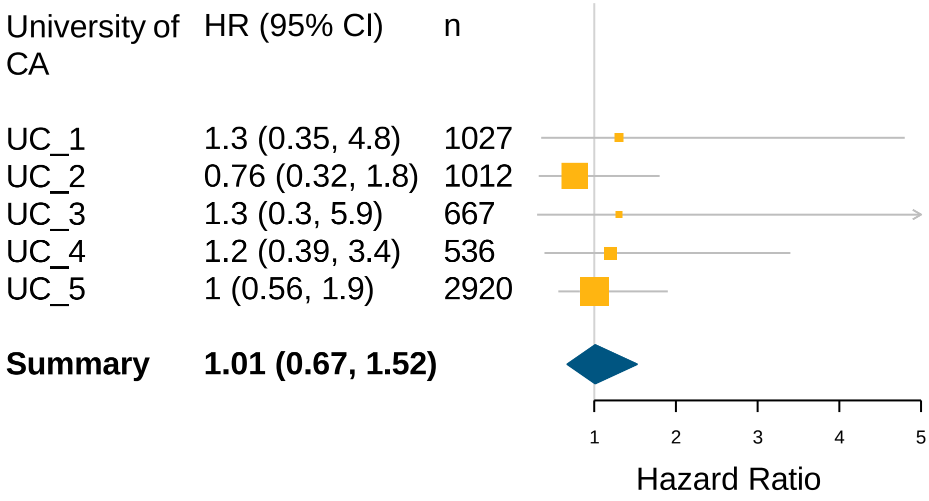

The table below shows the Leave-One-UC-Out diagnostics. The DFFITS value, Cook's distance, Covariance ratio, leave-one-out amount of heteroginity, indicator for influential estimates, comparator and treated groups are provided for each Leave-One-UC-Out analysis. The influential estimate from one UC with respect to pooled estimate are marked as Yes or No, with Yes indicating an influential UC and No otherwise.

eTable 60: Leave-One-UC-Out Sensitivity Analysis

DFFITs	Cook's Dist	Residual Heterogeneity	Influential	Comparator	Treated	UC
0.1317445	0.0173566	0	No	DPP4i	SGLT2i	UC_1
-0.3977522	0.1582068	0	No	DPP4i	SGLT2i	UC_2
0.0993140	0.0098633	0	No	DPP4i	SGLT2i	UC_3
0.1385654	0.0192004	0	No	DPP4i	SGLT2i	UC_4
-0.0394309	0.0015548	0	No	DPP4i	SGLT2i	UC_5

© 2023 Vashisht R et al. *JAMA Network Open.*

The forest plot illustrate the effect size of the comparison between GLP1ra and SGLT2i at each UC along with the effect size obtained from the random effect meta-analysis across all the UC for outcome Chronic Obstructive Pulmonary Disease

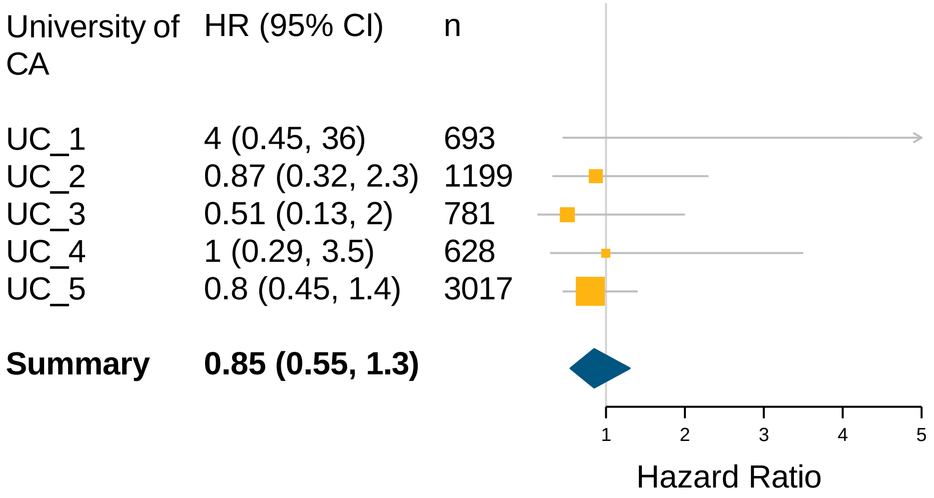

The table below shows the Leave-One-UC-Out diagnostics. The DFFITS value, Cook's distance, Covariance ratio, leave-one-out amount of heteroginity, indicator for influential estimates, comparator and treated groups are provided for each Leave-One-UC-Out analysis. The influential estimate from one UC with respect to pooled estimate are marked as Yes or No, with Yes indicating an influential UC and No otherwise.

eTable 61: Leave-One-UC-Out Sensitivity Analysis

DFFITs	Cook's Dist	Residual Heterogeneity	Influential	Comparator	Treated	UC
0.2799886	0.0783936	0	No	GLP1ra	SGLT2i	UC_1
0.0264933	0.0007019	0	No	GLP1ra	SGLT2i	UC_2
-0.2516678	0.0633367	0	No	GLP1ra	SGLT2i	UC_3
0.1001096	0.0100219	0	No	GLP1ra	SGLT2i	UC_4
-0.3476128	0.1208347	0	No	GLP1ra	SGLT2i	UC_5

© 2023 Vashisht R et al. *JAMA Network Open.*

The forest plot illustrate the effect size of the comparison between Sulfonylurea and DPP4i at each UC along with the effect size obtained from the random effect meta-analysis across all the UC for outcome Chronic Obstructive Pulmonary Disease

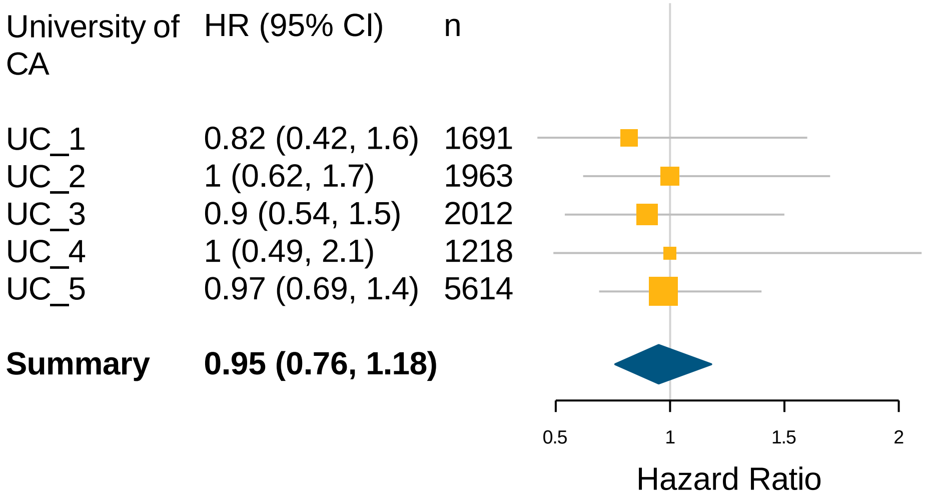

The table below shows the Leave-One-UC-Out diagnostics. The DFFITS value, Cook's distance, Covariance ratio, leave-one-out amount of heteroginity, indicator for influential estimates, comparator and treated groups are provided for each Leave-One-UC-Out analysis. The influential estimate from one UC with respect to pooled estimate are marked as Yes or No, with Yes indicating an influential UC and No otherwise.

eTable 62: Leave-One-UC-Out Sensitivity Analysis

DFFITs	Cook's Dist	Residual Heterogeneity	Influential	Comparator	Treated	UC
-0.1590090	0.0252839	0	No	Sulfonylurea	DPP4i	UC_1
0.1182256	0.0139773	0	No	Sulfonylurea	DPP4i	UC_2
-0.1053585	0.0111004	0	No	Sulfonylurea	DPP4i	UC_3
0.0503453	0.0025346	0	No	Sulfonylurea	DPP4i	UC_4
0.1434091	0.0205662	0	No	Sulfonylurea	DPP4i	UC_5

© 2023 Vashisht R et al. *JAMA Network Open.*

The forest plot illustrate the effect size of the comparison between Sulfonylurea and GLP1ra at each UC along with the effect size obtained from the random effect meta-analysis across all the UC for outcome Chronic Obstructive Pulmonary Disease

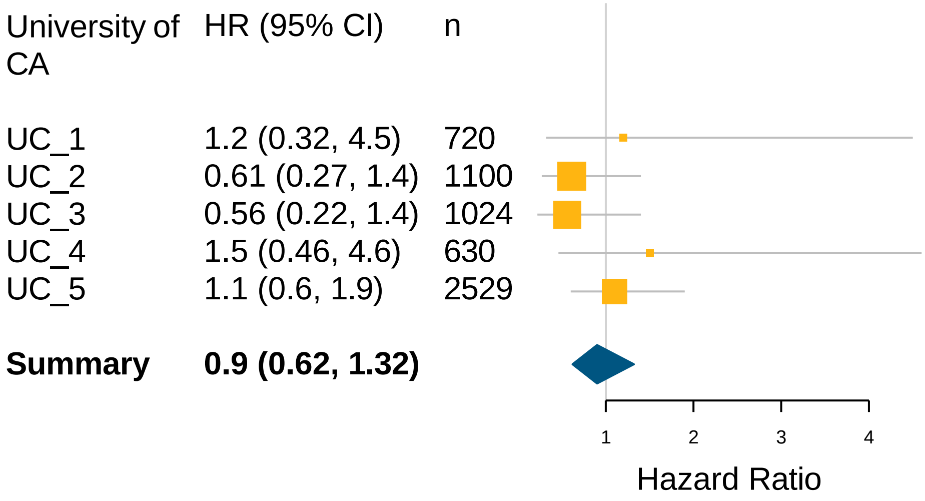

The table below shows the Leave-One-UC-Out diagnostics. The DFFITS value, Cook's distance, Covariance ratio, leave-one-out amount of heteroginity, indicator for influential estimates, comparator and treated groups are provided for each Leave-One-UC-Out analysis. The influential estimate from one UC with respect to pooled estimate are marked as Yes or No, with Yes indicating an influential UC and No otherwise.

eTable 63: Leave-One-UC-Out Sensitivity Analysis

DFFITs	Cook's Dist	Residual Heterogeneity	Influential	Comparator	Treated	UC
0.1430853	0.0206848	0.0046951	No	Sulfonylurea	GLP1ra	UC_1
-0.5454097	0.2974717	0.0000000	No	Sulfonylurea	GLP1ra	UC_2
-0.4974424	0.2474489	0.0000000	No	Sulfonylurea	GLP1ra	UC_3
0.3181861	0.1012424	0.0000000	No	Sulfonylurea	GLP1ra	UC_4
0.7733487	0.5980682	0.0000000	Yes	Sulfonylurea	GLP1ra	UC_5

© 2023 Vashisht R et al. *JAMA Network Open.*

The forest plot illustrate the effect size of the comparison between Sulfonylurea and SGLT2i at each UC along with the effect size obtained from the random effect meta-analysis across all the UC for outcome Chronic Obstructive Pulmonary Disease

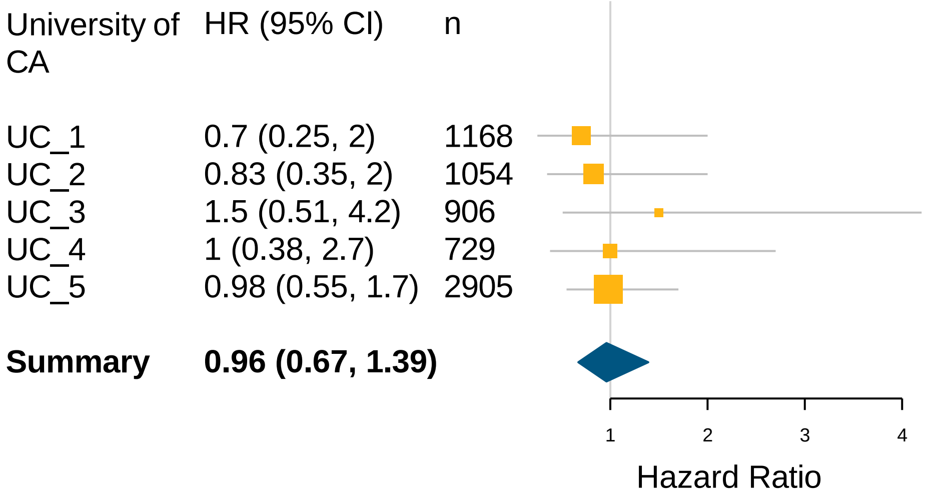

The table below shows the Leave-One-UC-Out diagnostics. The DFFITS value, Cook's distance, Covariance ratio, leave-one-out amount of heteroginity, indicator for influential estimates, comparator and treated groups are provided for each Leave-One-UC-Out analysis. The influential estimate from one UC with respect to pooled estimate are marked as Yes or No, with Yes indicating an influential UC and No otherwise.

eTable 64: Leave-One-UC-Out Sensitivity Analysis

DFFITs	Cook's Dist	Residual Heterogeneity	Influential	Comparator	Treated	UC
-0.2448383	0.0599458	0	No	Sulfonylurea	SGLT2i	UC_1
-0.1731555	0.0299828	0	No	Sulfonylurea	SGLT2i	UC_2
0.3290471	0.1082720	0	No	Sulfonylurea	SGLT2i	UC_3
0.0328503	0.0010791	0	No	Sulfonylurea	SGLT2i	UC_4
0.0685254	0.0046957	0	No	Sulfonylurea	SGLT2i	UC_5

© 2023 Vashisht R et al. *JAMA Network Open.*

4.10 Coronary Heart Disease

4.10.1 eTable: Drug comparision table

Effect size of each drug comparison at each UC health site is tabulated.

eTable 65: Hazard ratios of drug class comparison at each UC

Comparator	Treated	UC	N	Hazard Ratio (95% CI)	P-value	Adjusted P-Value
DPP4i	GLP1ra	UC_1	630	0.9 (0.42-1.9)	7.85480e-01	9.271662e-01
DPP4i	GLP1ra	UC_2	954	0.5 (0.28-0.91)	2.43109e-02	2.987280e-01
DPP4i	GLP1ra	UC_3	784	0.81 (0.4-1.7)	5.65995e-01	8.439845e-01
DPP4i	GLP1ra	UC_4	487	0.64 (0.26-1.5)	3.15609e-01	8.439845e-01
DPP4i	GLP1ra	UC_5	2408	1.1 (0.78-1.6)	5.74038e-01	8.439845e-01
DPP4i	SGLT2i	UC_1	917	0.95 (0.55-1.7)	8.66729e-01	9.630322e-01
DPP4i	SGLT2i	UC_2	855	0.98 (0.59-1.6)	9.52211e-01	9.683020e-01
DPP4i	SGLT2i	UC_3	579	0.77 (0.33-1.8)	5.44522e-01	8.439845e-01
DPP4i	SGLT2i	UC_4	467	0.85 (0.39-1.9)	6.84406e-01	8.927035e-01
DPP4i	SGLT2i	UC_5	2632	1.2 (0.84-1.6)	3.61287e-01	8.439845e-01
GLP1ra	SGLT2i	UC_1	629	1.4 (0.6-3.3)	4.29764e-01	8.439845e-01
GLP1ra	SGLT2i	UC_2	1076	1.9 (1.1-3.4)	2.98728e-02	2.987280e-01
GLP1ra	SGLT2i	UC_3	694	0.74 (0.28-2)	5.48516e-01	8.439845e-01
GLP1ra	SGLT2i	UC_4	568	1.5 (0.58-4)	3.85893e-01	8.439845e-01
GLP1ra	SGLT2i	UC_5	2712	1.1 (0.75-1.5)	7.47192e-01	9.271662e-01
Sulfonylurea	DPP4i	UC_1	1558	0.91 (0.62-1.3)	6.18922e-01	8.439845e-01
Sulfonylurea	DPP4i	UC_2	1713	0.84 (0.61-1.1)	2.66723e-01	8.439845e-01
Sulfonylurea	DPP4i	UC_3	1866	1.2 (0.87-1.8)	2.30830e-01	8.439845e-01
Sulfonylurea	DPP4i	UC_4	1105	0.78 (0.48-1.3)	3.02406e-01	8.439845e-01
Sulfonylurea	DPP4i	UC_5	5169	0.94 (0.76-1.2)	5.35690e-01	8.439845e-01
Sulfonylurea	GLP1ra	UC_1	690	0.99 (0.49-2)	9.68302e-01	9.683020e-01
Sulfonylurea	GLP1ra	UC_2	1024	0.41 (0.24-0.72)	1.92042e-03	5.761260e-02
Sulfonylurea	GLP1ra	UC_3	971	0.96 (0.47-2)	9.14697e-01	9.683020e-01
Sulfonylurea	GLP1ra	UC_4	591	0.69 (0.31-1.5)	3.59571e-01	8.439845e-01
Sulfonylurea	GLP1ra	UC_5	2344	1 (0.73-1.5)	8.03544e-01	9.271662e-01
Sulfonylurea	SGLT2i	UC_1	1069	1.2 (0.71-2.1)	4.76391e-01	8.439845e-01
Sulfonylurea	SGLT2i	UC_2	888	0.69 (0.42-1.1)	1.35181e-01	8.110860e-01
Sulfonylurea	SGLT2i	UC_3	820	1.5 (0.65-3.4)	3.46180e-01	8.439845e-01
Sulfonylurea	SGLT2i	UC_4	637	0.55 (0.27-1.1)	8.46396e-02	6.347970e-01
Sulfonylurea	SGLT2i	UC_5	2583	1.1 (0.78-1.5)	6.16733e-01	8.439845e-01

4.10.2 eFigure: Individual effect size, meta analysis and sensitivity analysis

The forest plot illustrate the effect size of the comparison between DPP4i and GLP1ra at each UC along with the effect size obtained from the random effect meta-analysis across all the UC for outcome Coronary Heart Disease

© 2023 Vashisht R et al. *JAMA Network Open.*

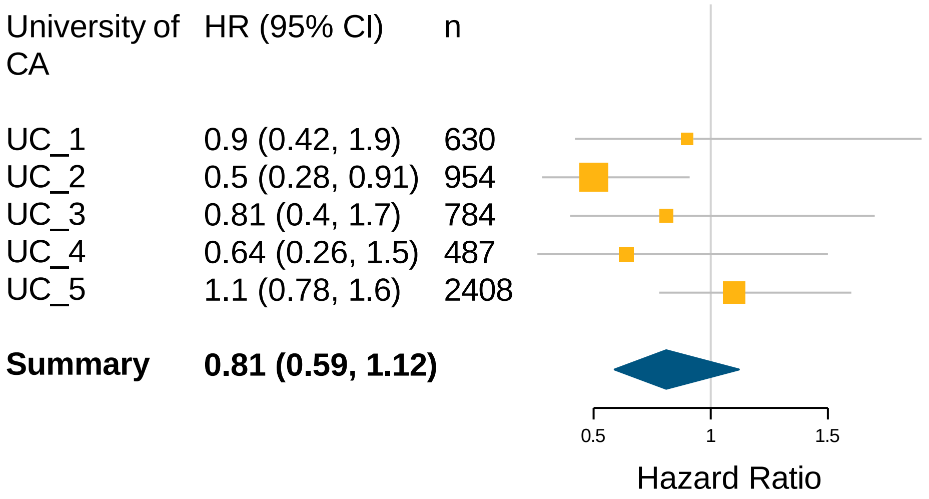

The table below shows the Leave-One-UC-Out diagnostics. The DFFITS value, Cook's distance, Covariance ratio, leave-one-out amount of heteroginity, indicator for influential estimates, comparator and treated groups are provided for each Leave-One-UC-Out analysis. The influential estimate from one UC with respect to pooled estimate are marked as Yes or No, with Yes indicating an influential UC and No otherwise.

eTable 66: Leave-One-UC-Out Sensitivity Analysis

DFFITs	Cook's Dist	Residual Heterogeneity	Influential	Comparator	Treated	UC
0.2457086	0.0732004	0.0768030	No	DPP4i	GLP1ra	UC_1
-1.2621902	1.1272708	0.0000000	Yes	DPP4i	GLP1ra	UC_2
0.1378658	0.0234782	0.0782030	No	DPP4i	GLP1ra	UC_3
-0.1052915	0.0121259	0.0596093	No	DPP4i	GLP1ra	UC_4
1.7349942	1.4251512	0.0000000	Yes	DPP4i	GLP1ra	UC_5

© 2023 Vashisht R et al. *JAMA Network Open.*

The forest plot illustrate the effect size of the comparison between DPP4i and SGLT2i at each UC along with the effect size obtained from the random effect meta-analysis across all the UC for outcome Coronary Heart Disease

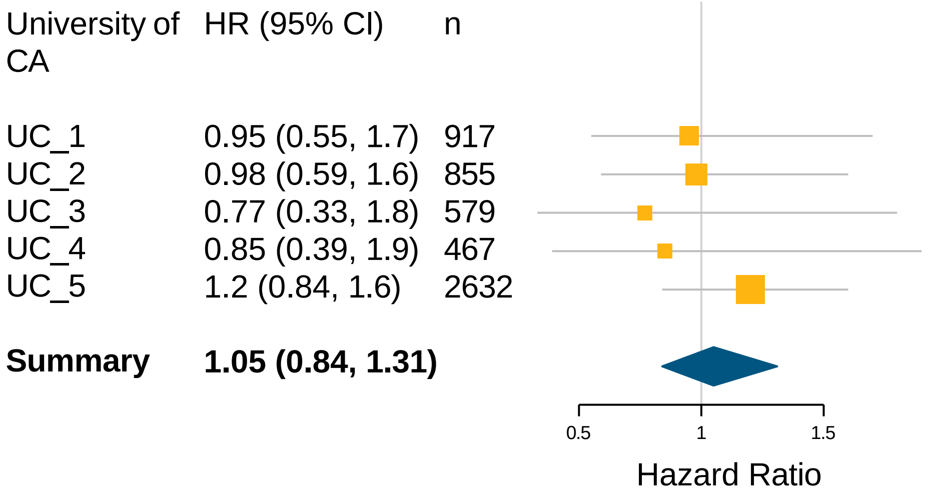

The table below shows the Leave-One-UC-Out diagnostics. The DFFITS value, Cook's distance, Covariance ratio, leave-one-out amount of heteroginity, indicator for influential estimates, comparator and treated groups are provided for each Leave-One-UC-Out analysis. The influential estimate from one UC with respect to pooled estimate are marked as Yes or No, with Yes indicating an influential UC and No otherwise.

eTable 67: Leave-One-UC-Out Sensitivity Analysis

DFFITs	Cook's Dist	Residual Heterogeneity	Influential	Comparator	Treated	UC
-0.1585937	0.0251520	0	No	DPP4i	SGLT2i	UC_1
-0.1451105	0.0210571	0	No	DPP4i	SGLT2i	UC_2
-0.2018903	0.0407597	0	No	DPP4i	SGLT2i	UC_3
-0.1587571	0.0252038	0	No	DPP4i	SGLT2i	UC_4
1.1360133	1.2905262	0	Yes	DPP4i	SGLT2i	UC_5

© 2023 Vashisht R et al. *JAMA Network Open.*

The forest plot illustrate the effect size of the comparison between GLP1ra and SGLT2i at each UC along with the effect size obtained from the random effect meta-analysis across all the UC for outcome Coronary Heart Disease

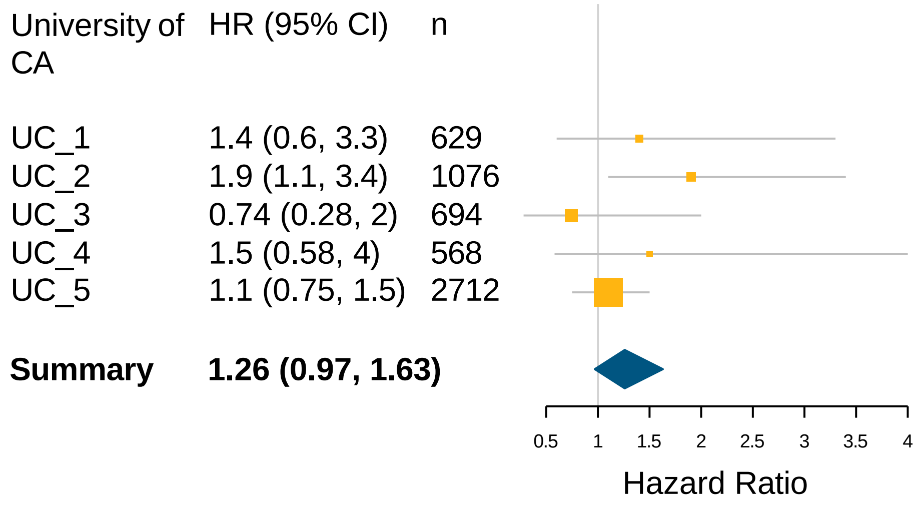

The table below shows the Leave-One-UC-Out diagnostics. The DFFITS value, Cook's distance, Covariance ratio, leave-one-out amount of heteroginity, indicator for influential estimates, comparator and treated groups are provided for each Leave-One-UC-Out analysis. The influential estimate from one UC with respect to pooled estimate are marked as Yes or No, with Yes indicating an influential UC and No otherwise.

eTable 68: Leave-One-UC-Out Sensitivity Analysis

DFFITs	Cook's Dist	Residual Heterogeneity	Influential	Comparator	Treated	UC
-0.0645354	0.0048192	0.0297158	No	GLP1ra	SGLT2i	UC_1
0.8359147	0.6987533	0.0000000	Yes	GLP1ra	SGLT2i	UC_2
-0.2976903	0.0886195	0.0000000	No	GLP1ra	SGLT2i	UC_3
-0.0229164	0.0005813	0.0259516	No	GLP1ra	SGLT2i	UC_4
-1.2556696	1.5767060	0.0000000	Yes	GLP1ra	SGLT2i	UC_5

© 2023 Vashisht R et al. *JAMA Network Open.*

The forest plot illustrate the effect size of the comparison between Sulfonylurea and DPP4i at each UC along with the effect size obtained from the random effect meta-analysis across all the UC for outcome Coronary Heart Disease

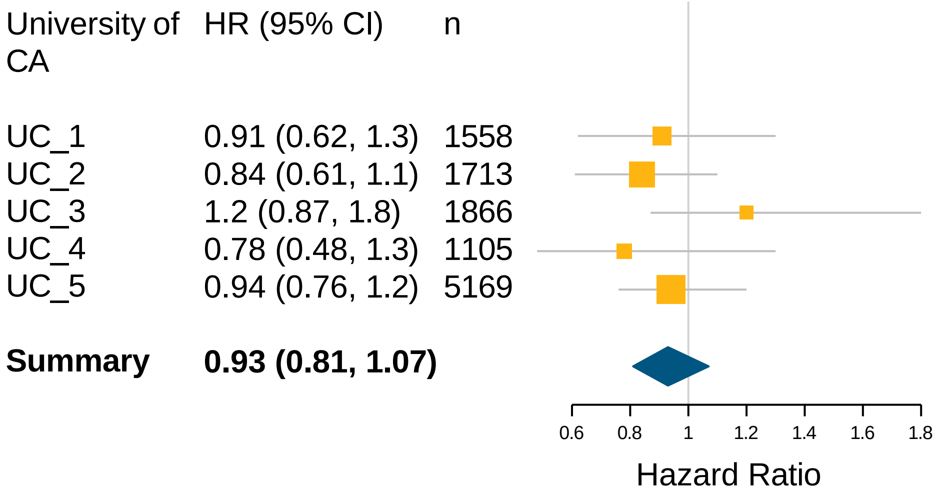

The table below shows the Leave-One-UC-Out diagnostics. The DFFITS value, Cook's distance, Covariance ratio, leave-one-out amount of heteroginity, indicator for influential estimates, comparator and treated groups are provided for each Leave-One-UC-Out analysis. The influential estimate from one UC with respect to pooled estimate are marked as Yes or No, with Yes indicating an influential UC and No otherwise.

eTable 69: Leave-One-UC-Out Sensitivity Analysis

DFFITs	Cook's Dist	Residual Heterogeneity	Influential	Comparator	Treated	UC
-0.0563612	0.0031766	0	No	Sulfonylurea	DPP4i	UC_1
-0.4325820	0.1871272	0	No	Sulfonylurea	DPP4i	UC_2
0.6288744	0.3954830	0	No	Sulfonylurea	DPP4i	UC_3
-0.2171018	0.0471332	0	No	Sulfonylurea	DPP4i	UC_4
0.0763449	0.0058286	0	No	Sulfonylurea	DPP4i	UC_5

© 2023 Vashisht R et al. *JAMA Network Open.*

The forest plot illustrate the effect size of the comparison between Sulfonylurea and GLP1ra at each UC along with the effect size obtained from the random effect meta-analysis across all the UC for outcome Coronary Heart Disease

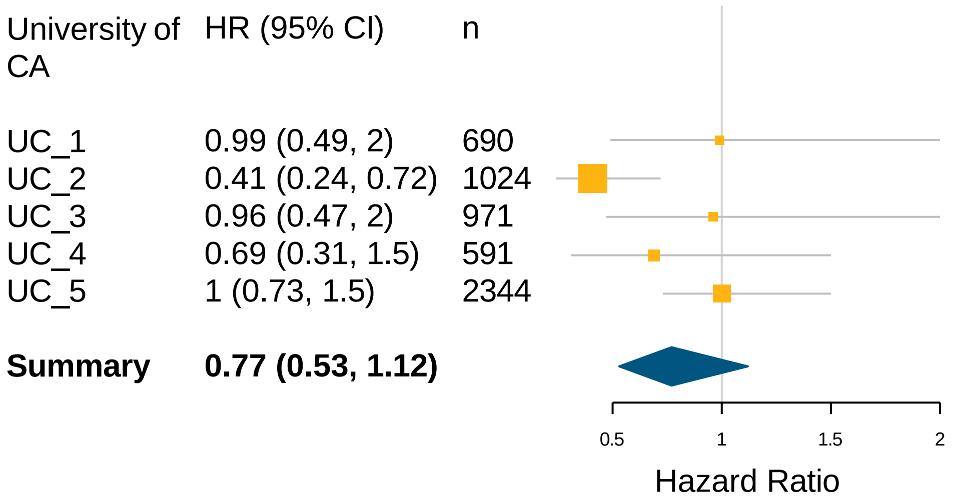

The table below shows the Leave-One-UC-Out diagnostics. The DFFITS value, Cook's distance, Covariance ratio, leave-one-out amount of heteroginity, indicator for influential estimates, comparator and treated groups are provided for each Leave-One-UC-Out analysis. The influential estimate from one UC with respect to pooled estimate are marked as Yes or No, with Yes indicating an influential UC and No otherwise.

eTable 70: Leave-One-UC-Out Sensitivity Analysis

DFFITs	Cook's Dist	Residual Heterogeneity	Influential	Comparator	Treated	UC
0.2700508	0.0863734	0.1264753	No	Sulfonylurea	GLP1ra	UC_1
-1.5698098	1.1710451	0.0000000	Yes	Sulfonylurea	GLP1ra	UC_2
0.2311025	0.0633355	0.1282371	No	Sulfonylurea	GLP1ra	UC_3
-0.0860577	0.0086004	0.1268280	No	Sulfonylurea	GLP1ra	UC_4
0.5787411	0.3411030	0.0889617	No	Sulfonylurea	GLP1ra	UC_5

The forest plot illustrate the effect size of the comparison between Sulfonylurea and SGLT2i at each UC along with the effect size obtained from the random effect meta-analysis across all the UC for outcome Coronary Heart Disease

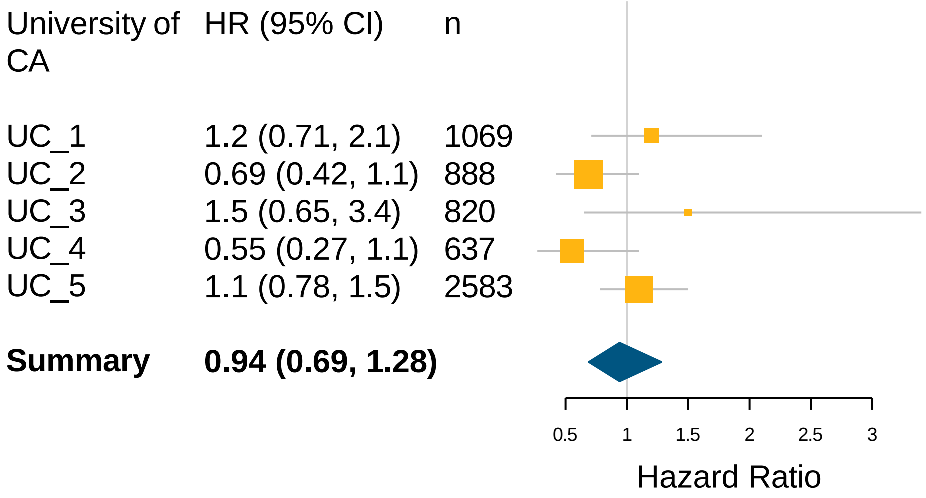

The table below shows the Leave-One-UC-Out diagnostics. The DFFITS value, Cook's distance, Covariance ratio, leave-one-out amount of heteroginity, indicator for influential estimates, comparator and treated groups are provided for each Leave-One-UC-Out analysis. The influential estimate from one UC with respect to pooled estimate are marked as Yes or No, with Yes indicating an influential UC and No otherwise.

eTable 71: Leave-One-UC-Out Sensitivity Analysis

DFFITs	Cook's Dist	Residual Heterogeneity	Influential	Comparator	Treated	UC
0.3747492	0.1676711	0.0716377	No	Sulfonylurea	SGLT2i	UC_1
-0.6547878	0.3752425	0.0341059	No	Sulfonylurea	SGLT2i	UC_2
0.3675909	0.1370992	0.0508723	No	Sulfonylurea	SGLT2i	UC_3
-0.5804915	0.2836124	0.0197062	No	Sulfonylurea	SGLT2i	UC_4
0.3578628	0.1887300	0.0832878	No	Sulfonylurea	SGLT2i	UC_5

© 2023 Vashisht R et al. *JAMA Network Open.*

4.11 Depression Bipolar Other Depressive Mood Disorders

4.11.1 eTable: Drug comparision table

Effect size of each drug comparison at each UC health site is tabulated.

eTable 72: Hazard ratios of drug class comparison at each UC

Comparator	Treated	UC	N	Hazard Ratio (95% CI)	P-value	Adjusted P-Value
DPP4i	GLP1ra	UC_1	563	2.1 (0.99-4.5)	5.26511e-02	3.081535e-01
DPP4i	GLP1ra	UC_2	819	0.58 (0.35-0.96)	3.35658e-02	2.517435e-01
DPP4i	GLP1ra	UC_3	624	0.91 (0.56-1.5)	7.16628e-01	9.578844e-01
DPP4i	GLP1ra	UC_4	444	1.2 (0.63-2.2)	6.16342e-01	9.469770e-01
DPP4i	GLP1ra	UC_5	2243	1 (0.74-1.4)	8.60210e-01	9.740162e-01
DPP4i	SGLT2i	UC_1	918	0.96 (0.5-1.8)	8.98329e-01	9.740162e-01
DPP4i	SGLT2i	UC_2	860	0.98 (0.61-1.6)	9.41549e-01	9.740162e-01
DPP4i	SGLT2i	UC_3	523	0.35 (0.14-0.88)	2.64295e-02	2.517435e-01
DPP4i	SGLT2i	UC_4	473	0.96 (0.42-2.2)	9.25328e-01	9.740162e-01
DPP4i	SGLT2i	UC_5	2599	0.92 (0.66-1.3)	6.31318e-01	9.469770e-01
GLP1ra	SGLT2i	UC_1	584	1 (0.52-1.9)	9.95198e-01	9.951980e-01
GLP1ra	SGLT2i	UC_2	977	1.2 (0.71-2)	5.02604e-01	9.068300e-01
GLP1ra	SGLT2i	UC_3	564	0.3 (0.13-0.7)	5.33202e-03	7.998030e-02
GLP1ra	SGLT2i	UC_4	519	0.55 (0.26-1.2)	1.18140e-01	4.116600e-01
GLP1ra	SGLT2i	UC_5	2511	0.79 (0.57-1.1)	1.72681e-01	4.709482e-01
Sulfonylurea	DPP4i	UC_1	1543	0.95 (0.64-1.4)	7.98237e-01	9.578844e-01
Sulfonylurea	DPP4i	UC_2	1673	1.1 (0.83-1.5)	4.45138e-01	8.902760e-01
Sulfonylurea	DPP4i	UC_3	1651	1.1 (0.78-1.4)	6.92541e-01	9.578844e-01
Sulfonylurea	DPP4i	UC_4	1098	1.2 (0.71-1.9)	5.44098e-01	9.068300e-01
Sulfonylurea	DPP4i	UC_5	5131	0.92 (0.75-1.1)	4.24598e-01	8.902760e-01
Sulfonylurea	GLP1ra	UC_1	605	1.4 (0.77-2.5)	2.73388e-01	6.834700e-01
Sulfonylurea	GLP1ra	UC_2	877	0.93 (0.54-1.6)	7.87385e-01	9.578844e-01
Sulfonylurea	GLP1ra	UC_3	770	1.5 (0.9-2.5)	1.23498e-01	4.116600e-01
Sulfonylurea	GLP1ra	UC_4	519	1.8 (0.88-3.5)	1.11959e-01	4.116600e-01
Sulfonylurea	GLP1ra	UC_5	2183	0.96 (0.7-1.3)	7.86260e-01	9.578844e-01
Sulfonylurea	SGLT2i	UC_1	1073	0.76 (0.43-1.4)	3.53598e-01	8.159954e-01
Sulfonylurea	SGLT2i	UC_2	931	1.5 (0.87-2.5)	1.44313e-01	4.329390e-01
Sulfonylurea	SGLT2i	UC_3	736	0.26 (0.11-0.58)	1.12629e-03	3.378870e-02
Sulfonylurea	SGLT2i	UC_4	643	0.8 (0.4-1.6)	5.21582e-01	9.068300e-01
Sulfonylurea	SGLT2i	UC_5	2607	0.72 (0.51-1)	6.16307e-02	3.081535e-01

4.11.2 eFigure: Individual effect size, meta analysis and sensitivity analysis

The forest plot illustrate the effect size of the comparison between DPP4i and GLP1ra at each UC along with the effect size obtained from the random effect meta-analysis across all the UC for outcome Depression Bipolar Other Depressive Mood Disorders

© 2023 Vashisht R et al. *JAMA Network Open.*

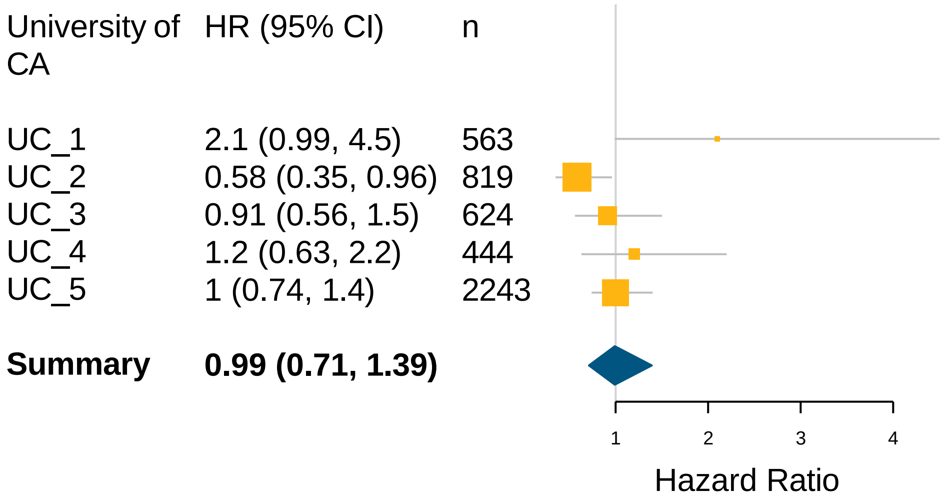

The table below shows the Leave-One-UC-Out diagnostics. The DFFITS value, Cook's distance, Covariance ratio, leave-one-out amount of heteroginity, indicator for influential estimates, comparator and treated groups are provided for each Leave-One-UC-Out analysis. The influential estimate from one UC with respect to pooled estimate are marked as Yes or No, with Yes indicating an influential UC and No otherwise.

eTable 73: Leave-One-UC-Out Sensitivity Analysis

DFFITs	Cook's Dist	Residual Heterogeneity	Influential	Comparator	Treated	UC
0.7117607	0.3876309	0.0216220	No	DPP4i	GLP1ra	UC_1
-0.7839028	0.3655640	0.0172116	No	DPP4i	GLP1ra	UC_2
-0.1977667	0.0537897	0.1255511	No	DPP4i	GLP1ra	UC_3
0.1647372	0.0313197	0.1011404	No	DPP4i	GLP1ra	UC_4
-0.0971701	0.0173798	0.1585455	No	DPP4i	GLP1ra	UC_5

© 2023 Vashisht R et al. *JAMA Network Open.*

The forest plot illustrate the effect size of the comparison between DPP4i and SGLT2i at each UC along with the effect size obtained from the random effect meta-analysis across all the UC for outcome Depression Bipolar Other Depressive Mood Disorders

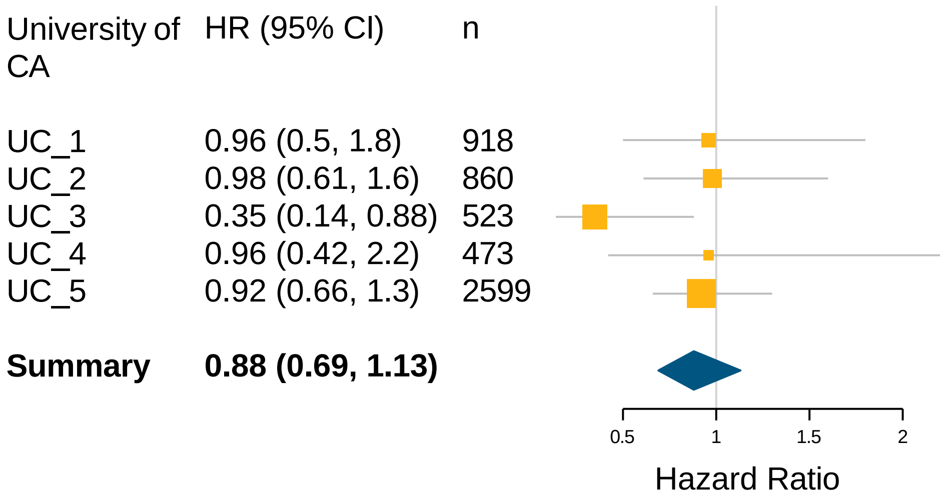

The table below shows the Leave-One-UC-Out diagnostics. The DFFITS value, Cook's distance, Covariance ratio, leave-one-out amount of heteroginity, indicator for influential estimates, comparator and treated groups are provided for each Leave-One-UC-Out analysis. The influential estimate from one UC with respect to pooled estimate are marked as Yes or No, with Yes indicating an influential UC and No otherwise.

eTable 74: Leave-One-UC-Out Sensitivity Analysis

DFFITs	Cook's Dist	Residual Heterogeneity	Influential	Comparator	Treated	UC
0.2811636	0.0983357	0.0321846	No	DPP4i	SGLT2i	UC_1
0.4098713	0.2438936	0.0345046	No	DPP4i	SGLT2i	UC_2
-0.5544455	0.3006636	0.0000000	No	DPP4i	SGLT2i	UC_3
0.2199761	0.0548826	0.0295435	No	DPP4i	SGLT2i	UC_4
0.3398179	0.2522444	0.0461984	No	DPP4i	SGLT2i	UC_5

© 2023 Vashisht R et al. *JAMA Network Open.*

The forest plot illustrate the effect size of the comparison between GLP1ra and SGLT2i at each UC along with the effect size obtained from the random effect meta-analysis across all the UC for outcome Depression Bipolar Other Depressive Mood Disorders

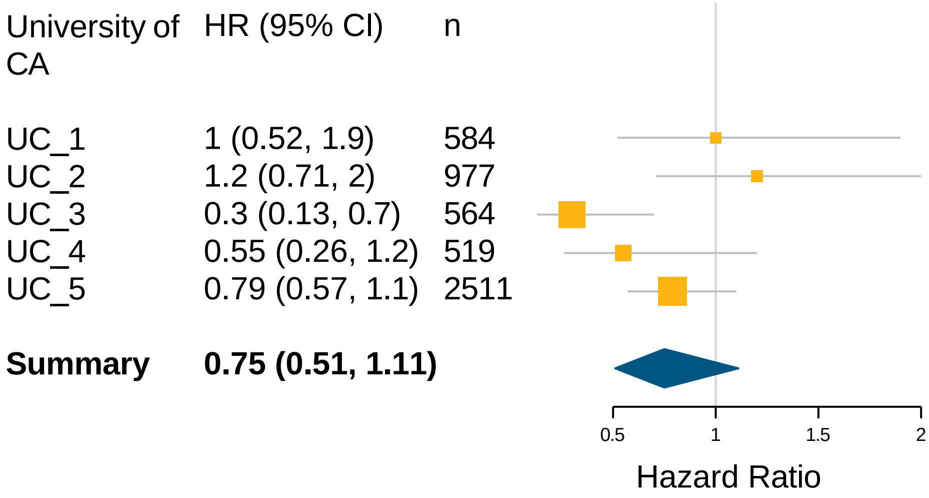

The table below shows the Leave-One-UC-Out diagnostics. The DFFITS value, Cook's distance, Covariance ratio, leave-one-out amount of heteroginity, indicator for influential estimates, comparator and treated groups are provided for each Leave-One-UC-Out analysis. The influential estimate from one UC with respect to pooled estimate are marked as Yes or No, with Yes indicating an influential UC and No otherwise.

eTable 75: Leave-One-UC-Out Sensitivity Analysis

DFFITs	Cook's Dist	Residual Heterogeneity	Influential	Comparator	Treated	UC
0.3802393	0.1741374	0.1462601	No	GLP1ra	SGLT2i	UC_1
0.6910126	0.4485560	0.0924227	No	GLP1ra	SGLT2i	UC_2
-0.8763843	0.5167750	0.0088819	Yes	GLP1ra	SGLT2i	UC_3
-0.2455622	0.0653906	0.1244233	No	GLP1ra	SGLT2i	UC_4
0.2041527	0.0838163	0.2353528	No	GLP1ra	SGLT2i	UC_5

© 2023 Vashisht R et al. *JAMA Network Open.*

The forest plot illustrate the effect size of the comparison between Sulfonylurea and DPP4i at each UC along with the effect size obtained from the random effect meta-analysis across all the UC for outcome Depression Bipolar Other Depressive Mood Disorders

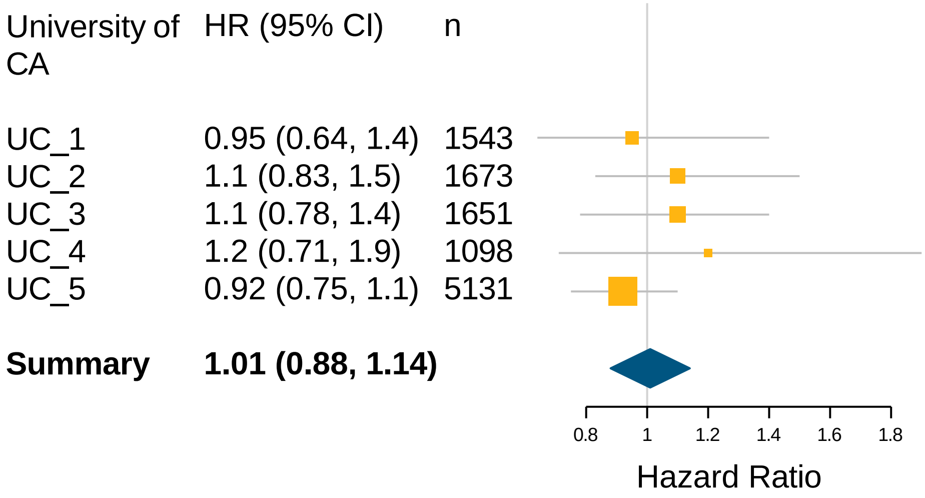

The table below shows the Leave-One-UC-Out diagnostics. The DFFITS value, Cook's distance, Covariance ratio, leave-one-out amount of heteroginity, indicator for influential estimates, comparator and treated groups are provided for each Leave-One-UC-Out analysis. The influential estimate from one UC with respect to pooled estimate are marked as Yes or No, with Yes indicating an influential UC and No otherwise.

eTable 76: Leave-One-UC-Out Sensitivity Analysis

DFFITs	Cook's Dist	Residual Heterogeneity	Influential	Comparator	Treated	UC
-0.1044880	0.0109177	0	No	Sulfonylurea	DPP4i	UC_1
0.3159209	0.0998060	0	No	Sulfonylurea	DPP4i	UC_2
0.3251418	0.1057172	0	No	Sulfonylurea	DPP4i	UC_3
0.1962060	0.0384968	0	No	Sulfonylurea	DPP4i	UC_4
-1.1013278	1.2129230	0	Yes	Sulfonylurea	DPP4i	UC_5

© 2023 Vashisht R et al. *JAMA Network Open.*

The forest plot illustrate the effect size of the comparison between Sulfonylurea and GLP1ra at each UC along with the effect size obtained from the random effect meta-analysis across all the UC for outcome Depression Bipolar Other Depressive Mood Disorders

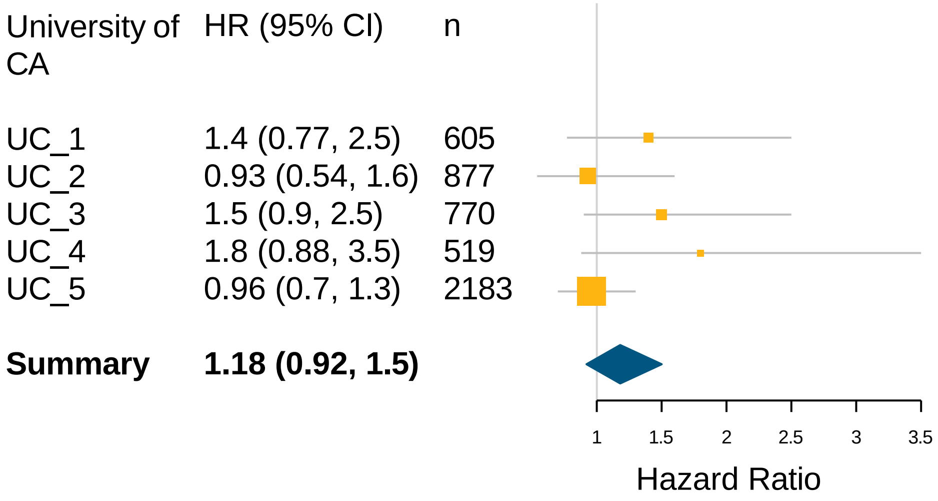

The table below shows the Leave-One-UC-Out diagnostics. The DFFITS value, Cook's distance, Covariance ratio, leave-one-out amount of heteroginity, indicator for influential estimates, comparator and treated groups are provided for each Leave-One-UC-Out analysis. The influential estimate from one UC with respect to pooled estimate are marked as Yes or No, with Yes indicating an influential UC and No otherwise.

eTable 77: Leave-One-UC-Out Sensitivity Analysis

DFFITs	Cook's Dist	Residual Heterogeneity	Influential	Comparator	Treated	UC
0.1174649	0.0156615	0.0301591	No	Sulfonylurea	GLP1ra	UC_1
-0.4952714	0.2776834	0.0280600	No	Sulfonylurea	GLP1ra	UC_2
0.4499796	0.2015350	0.0154395	No	Sulfonylurea	GLP1ra	UC_3
0.5691855	0.2947994	0.0032357	No	Sulfonylurea	GLP1ra	UC_4
-1.2703243	0.9871141	0.0000000	Yes	Sulfonylurea	GLP1ra	UC_5

© 2023 Vashisht R et al. *JAMA Network Open.*

The forest plot illustrate the effect size of the comparison between Sulfonylurea and SGLT2i at each UC along with the effect size obtained from the random effect meta-analysis across all the UC for outcome Depression Bipolar Other Depressive Mood Disorders

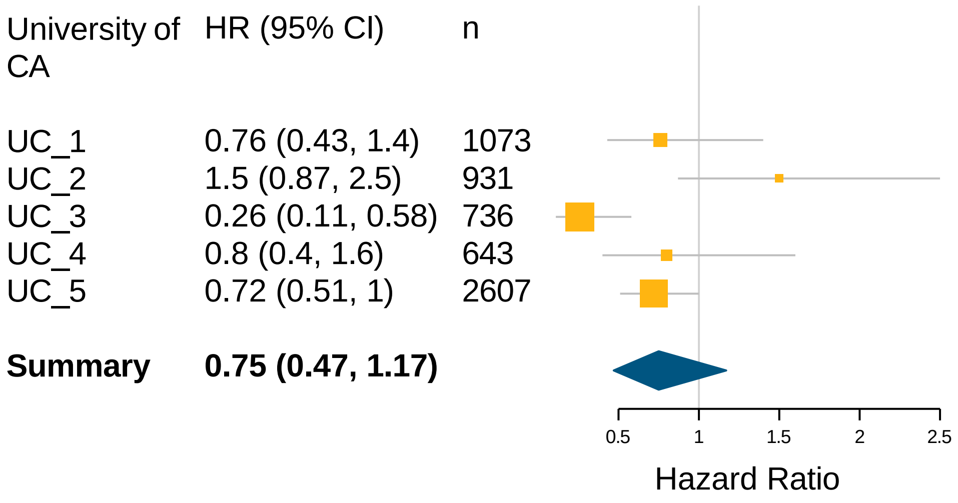

The table below shows the Leave-One-UC-Out diagnostics. The DFFITS value, Cook's distance, Covariance ratio, leave-one-out amount of heteroginity, indicator for influential estimates, comparator and treated groups are provided for each Leave-One-UC-Out analysis. The influential estimate from one UC with respect to pooled estimate are marked as Yes or No, with Yes indicating an influential UC and No otherwise.

eTable 78: Leave-One-UC-Out Sensitivity Analysis

DFFITs	Cook's Dist	Residual Heterogeneity	Influential	Comparator	Treated	UC
0.0756279	0.0075693	0.2597872	No	Sulfonylurea	SGLT2i	UC_1
0.9163717	0.4972413	0.0735426	Yes	Sulfonylurea	SGLT2i	UC_2
-0.9292560	0.5804095	0.0580740	Yes	Sulfonylurea	SGLT2i	UC_3
0.1084911	0.0143379	0.2394149	No	Sulfonylurea	SGLT2i	UC_4
0.0484801	0.0042509	0.3388226	No	Sulfonylurea	SGLT2i	UC_5

4.12 Diarrhea

4.12.1 eTable: Drug comparision table

Effect size of each drug comparison at each UC health site is tabulated.

eTable 79: Hazard ratios of drug class comparison at each UC

Comparator	Treated	UC	N	Hazard Ratio (95% CI)	P-value	Adjusted P-Value
DPP4i	GLP1ra	UC_1	661	1.8 (0.78-4)	1.70437e-01	6.677513e-01
DPP4i	GLP1ra	UC_2	947	1 (0.61-1.7)	9.10139e-01	9.904080e-01
DPP4i	GLP1ra	UC_3	763	1.1 (0.65-1.8)	7.35965e-01	9.904080e-01
DPP4i	GLP1ra	UC_4	512	1.2 (0.43-3.3)	7.35034e-01	9.904080e-01
DPP4i	GLP1ra	UC_5	2512	1 (0.7-1.4)	9.90408e-01	9.904080e-01
DPP4i	SGLT2i	UC_1	991	1.4 (0.63-3.3)	3.93234e-01	7.802179e-01
DPP4i	SGLT2i	UC_2	978	0.62 (0.36-1.1)	8.42407e-02	4.938945e-01
DPP4i	SGLT2i	UC_3	623	0.54 (0.26-1.1)	9.87789e-02	4.938945e-01
DPP4i	SGLT2i	UC_4	512	4.8 (1.4-17)	1.40227e-02	4.206810e-01
DPP4i	SGLT2i	UC_5	2829	1 (0.7-1.5)	9.22082e-01	9.904080e-01
GLP1ra	SGLT2i	UC_1	669	0.74 (0.33-1.7)	4.65722e-01	7.802179e-01
GLP1ra	SGLT2i	UC_2	1136	0.83 (0.49-1.4)	4.94138e-01	7.802179e-01
GLP1ra	SGLT2i	UC_3	721	0.67 (0.34-1.3)	2.38437e-01	6.906300e-01
GLP1ra	SGLT2i	UC_4	603	1.8 (0.64-4.9)	2.69413e-01	6.906300e-01
GLP1ra	SGLT2i	UC_5	2874	0.78 (0.54-1.1)	1.78067e-01	6.677513e-01
Sulfonylurea	DPP4i	UC_1	1658	1.1 (0.69-1.8)	6.76914e-01	9.904080e-01
Sulfonylurea	DPP4i	UC_2	1877	1.4 (0.97-1.9)	7.29371e-02	4.938945e-01
Sulfonylurea	DPP4i	UC_3	1912	1.2 (0.88-1.7)	2.46120e-01	6.906300e-01
Sulfonylurea	DPP4i	UC_4	1206	0.73 (0.42-1.3)	2.76252e-01	6.906300e-01
Sulfonylurea	DPP4i	UC_5	5519	1 (0.8-1.3)	9.88778e-01	9.904080e-01
Sulfonylurea	GLP1ra	UC_1	705	2.1 (0.92-4.9)	7.70652e-02	4.938945e-01
Sulfonylurea	GLP1ra	UC_2	1038	1.2 (0.74-2)	4.37528e-01	7.802179e-01
Sulfonylurea	GLP1ra	UC_3	957	1.2 (0.74-2)	4.68058e-01	7.802179e-01
Sulfonylurea	GLP1ra	UC_4	612	0.75 (0.33-1.7)	4.87498e-01	7.802179e-01
Sulfonylurea	GLP1ra	UC_5	2450	1.4 (0.98-2.1)	6.21658e-02	4.938945e-01
Sulfonylurea	SGLT2i	UC_1	1150	1 (0.5-2.2)	9.05759e-01	9.904080e-01
Sulfonylurea	SGLT2i	UC_2	1043	1 (0.57-1.8)	9.59785e-01	9.904080e-01
Sulfonylurea	SGLT2i	UC_3	869	0.74 (0.39-1.4)	3.48584e-01	7.802179e-01
Sulfonylurea	SGLT2i	UC_4	703	1.1 (0.5-2.3)	8.60551e-01	9.904080e-01
Sulfonylurea	SGLT2i	UC_5	2825	0.98 (0.66-1.4)	9.00160e-01	9.904080e-01

4.12.2 eFigure: Individual effect size, meta analysis and sensitivity analysis

The forest plot illustrate the effect size of the comparison between DPP4i and GLP1ra at each UC along with the effect size obtained from the random effect meta-analysis across all the UC for outcome Diarrhea

© 2023 Vashisht R et al. *JAMA Network Open.*

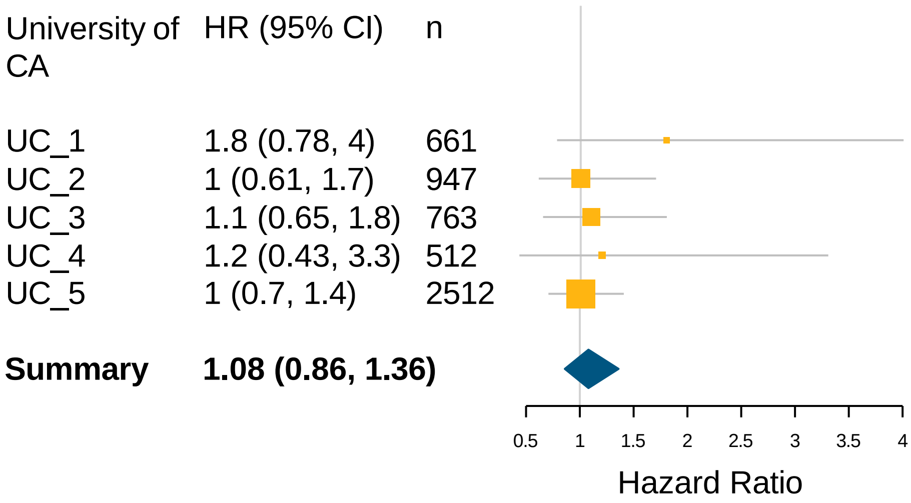

The table below shows the Leave-One-UC-Out diagnostics. The DFFITS value, Cook's distance, Covariance ratio, leave-one-out amount of heteroginity, indicator for influential estimates, comparator and treated groups are provided for each Leave-One-UC-Out analysis. The influential estimate from one UC with respect to pooled estimate are marked as Yes or No, with Yes indicating an influential UC and No otherwise.

eTable 80: Leave-One-UC-Out Sensitivity Analysis

DFFITs	Cook's Dist	Residual Heterogeneity	Influential	Comparator	Treated	UC
0.3795767	0.1440784	0	No	DPP4i	GLP1ra	UC_1
-0.1688626	0.0285146	0	No	DPP4i	GLP1ra	UC_2
0.0404467	0.0016359	0	No	DPP4i	GLP1ra	UC_3
0.0487811	0.0023796	0	No	DPP4i	GLP1ra	UC_4
-0.5339675	0.2851213	0	No	DPP4i	GLP1ra	UC_5

© 2023 Vashisht R et al. *JAMA Network Open.*

The forest plot illustrate the effect size of the comparison between DPP4i and SGLT2i at each UC along with the effect size obtained from the random effect meta-analysis across all the UC for outcome Diarrhea

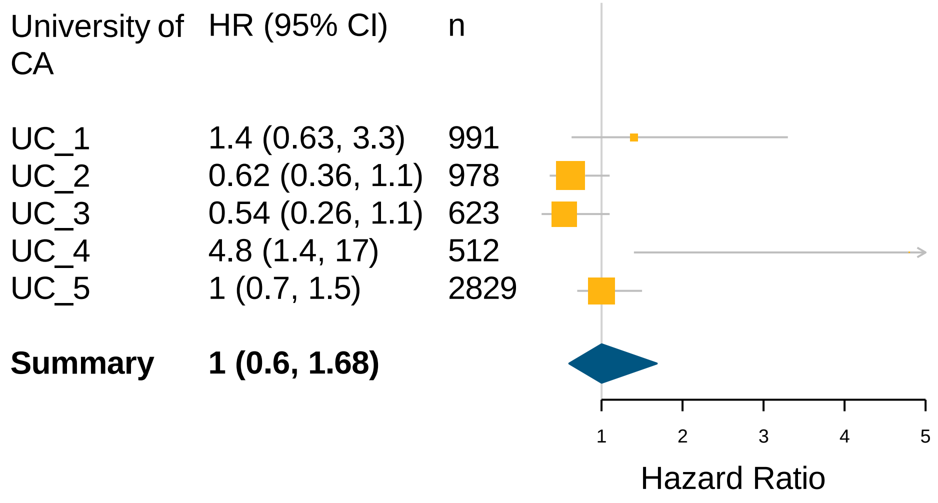

The table below shows the Leave-One-UC-Out diagnostics. The DFFITS value, Cook's distance, Covariance ratio, leave-one-out amount of heteroginity, indicator for influential estimates, comparator and treated groups are provided for each Leave-One-UC-Out analysis. The influential estimate from one UC with respect to pooled estimate are marked as Yes or No, with Yes indicating an influential UC and No otherwise.

eTable 81: Leave-One-UC-Out Sensitivity Analysis

DFFITs	Cook's Dist	Residual Heterogeneity	Influential	Comparator	Treated	UC
0.2106879	0.0495995	0.2610630	No	DPP4i	SGLT2i	UC_1
-0.5715659	0.3997500	0.2811127	No	DPP4i	SGLT2i	UC_2
-0.5932971	0.3710076	0.2338075	No	DPP4i	SGLT2i	UC_3
0.8461983	0.5311858	0.0546762	Yes	DPP4i	SGLT2i	UC_4
-0.1764271	0.0599560	0.4489412	No	DPP4i	SGLT2i	UC_5

© 2023 Vashisht R et al. *JAMA Network Open.*

The forest plot illustrate the effect size of the comparison between GLP1ra and SGLT2i at each UC along with the effect size obtained from the random effect meta-analysis across all the UC for outcome Diarrhea

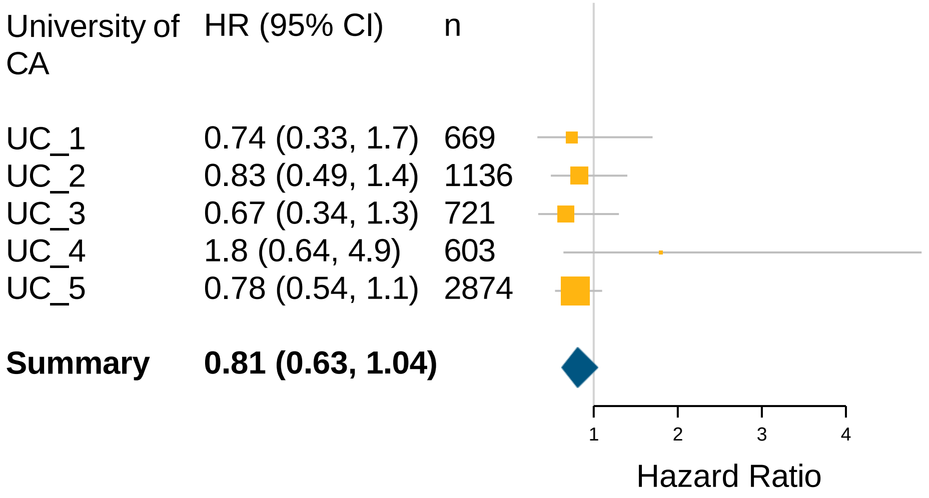

The table below shows the Leave-One-UC-Out diagnostics. The DFFITS value, Cook's distance, Covariance ratio, leave-one-out amount of heteroginity, indicator for influential estimates, comparator and treated groups are provided for each Leave-One-UC-Out analysis. The influential estimate from one UC with respect to pooled estimate are marked as Yes or No, with Yes indicating an influential UC and No otherwise.

eTable 82: Leave-One-UC-Out Sensitivity Analysis

DFFITs	Cook's Dist	Residual Heterogeneity	Influential	Comparator	Treated	UC
-0.0723497	0.0052345	0	No	GLP1ra	SGLT2i	UC_1
0.0548723	0.0030110	0	No	GLP1ra	SGLT2i	UC_2
-0.2384576	0.0568620	0	No	GLP1ra	SGLT2i	UC_3
0.3989098	0.1591290	0	No	GLP1ra	SGLT2i	UC_4
-0.2854372	0.0814744	0	No	GLP1ra	SGLT2i	UC_5

The forest plot illustrate the effect size of the comparison between Sulfonylurea and DPP4i at each UC along with the effect size obtained from the random effect meta-analysis across all the UC for outcome Diarrhea

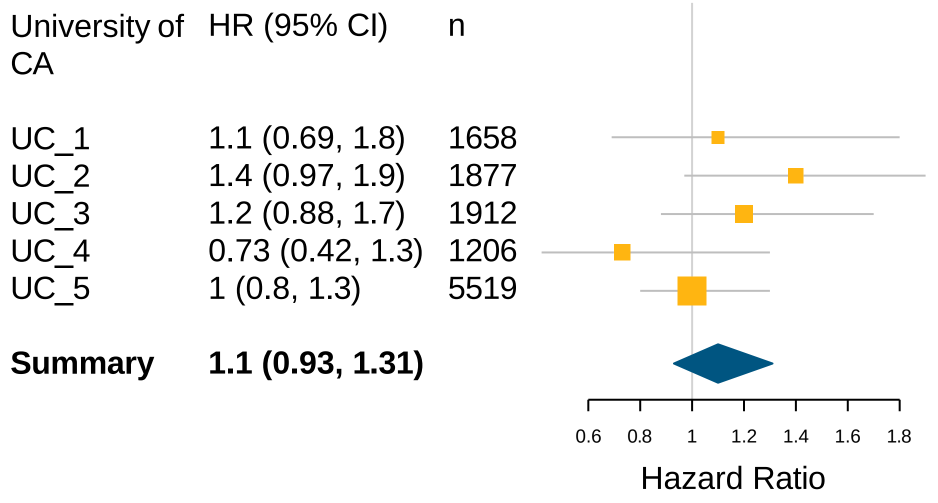

The table below shows the Leave-One-UC-Out diagnostics. The DFFITS value, Cook's distance, Covariance ratio, leave-one-out amount of heteroginity, indicator for influential estimates, comparator and treated groups are provided for each Leave-One-UC-Out analysis. The influential estimate from one UC with respect to pooled estimate are marked as Yes or No, with Yes indicating an influential UC and No otherwise.

eTable 83: Leave-One-UC-Out Sensitivity Analysis

DFFITs	Cook's Dist	Residual Heterogeneity	Influential	Comparator	Treated	UC
0.0172536	0.0003483	0.0185634	No	Sulfonylurea	DPP4i	UC_1
0.8075276	0.5242316	0.0000000	Yes	Sulfonylurea	DPP4i	UC_2
0.2646546	0.0924791	0.0185124	No	Sulfonylurea	DPP4i	UC_3
-0.3665094	0.1236502	0.0000000	No	Sulfonylurea	DPP4i	UC_4
-0.4800659	0.2838533	0.0123912	No	Sulfonylurea	DPP4i	UC_5

© 2023 Vashisht R et al. *JAMA Network Open.*

The forest plot illustrate the effect size of the comparison between Sulfonylurea and GLP1ra at each UC along with the effect size obtained from the random effect meta-analysis across all the UC for outcome Diarrhea

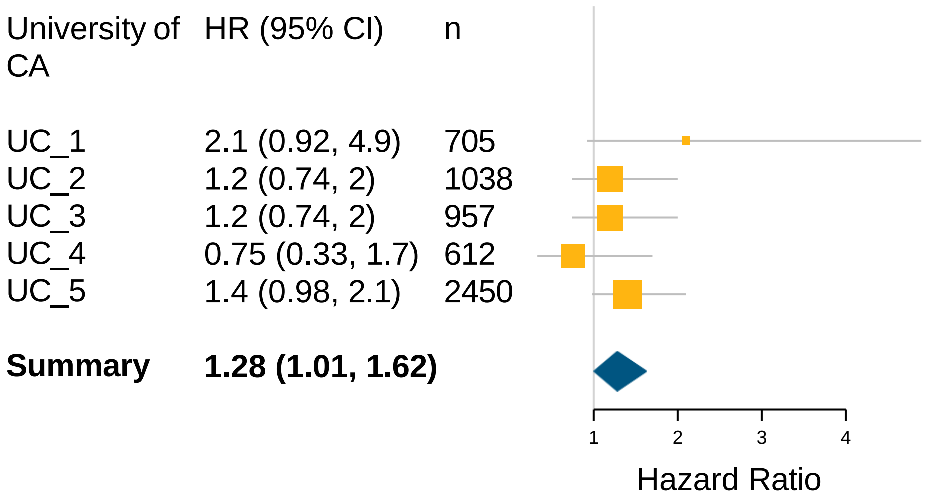

The table below shows the Leave-One-UC-Out diagnostics. The DFFITS value, Cook's distance, Covariance ratio, leave-one-out amount of heteroginity, indicator for influential estimates, comparator and treated groups are provided for each Leave-One-UC-Out analysis. The influential estimate from one UC with respect to pooled estimate are marked as Yes or No, with Yes indicating an influential UC and No otherwise.

eTable 84: Leave-One-UC-Out Sensitivity Analysis

DFFITs	Cook's Dist	Residual Heterogeneity	Influential	Comparator	Treated	UC
0.3561306	0.1268290	0.0000000	No	Sulfonylurea	GLP1ra	UC_1
-0.1267669	0.0177961	0.0069107	No	Sulfonylurea	GLP1ra	UC_2
-0.1267669	0.0177961	0.0069107	No	Sulfonylurea	GLP1ra	UC_3
-0.4023561	0.1618905	0.0000000	No	Sulfonylurea	GLP1ra	UC_4
0.4626740	0.2140672	0.0000000	No	Sulfonylurea	GLP1ra	UC_5

© 2023 Vashisht R et al. *JAMA Network Open.*

The forest plot illustrate the effect size of the comparison between Sulfonylurea and SGLT2i at each UC along with the effect size obtained from the random effect meta-analysis across all the UC for outcome Diarrhea

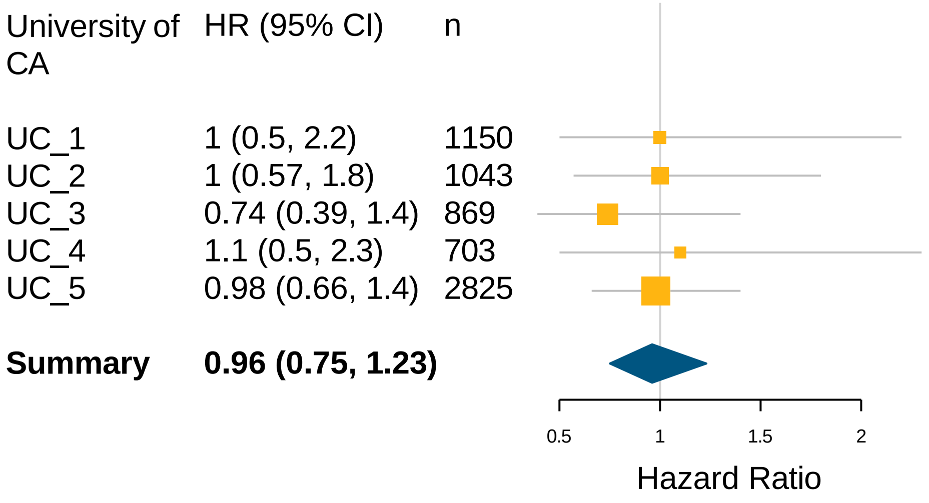

The table below shows the Leave-One-UC-Out diagnostics. The DFFITS value, Cook's distance, Covariance ratio, leave-one-out amount of heteroginity, indicator for influential estimates, comparator and treated groups are provided for each Leave-One-UC-Out analysis. The influential estimate from one UC with respect to pooled estimate are marked as Yes or No, with Yes indicating an influential UC and No otherwise.

eTable 85: Leave-One-UC-Out Sensitivity Analysis

DFFITs	Cook's Dist	Residual Heterogeneity	Influential	Comparator	Treated	UC
0.0447268	0.0020005	0	No	Sulfonylurea	SGLT2i	UC_1
0.0810898	0.0065755	0	No	Sulfonylurea	SGLT2i	UC_2
-0.3621294	0.1311377	0	No	Sulfonylurea	SGLT2i	UC_3
0.1314097	0.0172685	0	No	Sulfonylurea	SGLT2i	UC_4
0.1501446	0.0225434	0	No	Sulfonylurea	SGLT2i	UC_5

© 2023 Vashisht R et al. *JAMA Network Open.*

4.13 Fracture Of Bone

4.13.1 eTable: Drug comparision table

Effect size of each drug comparison at each UC health site is tabulated.

eTable 86: Hazard ratios of drug class comparison at each UC

Comparator	Treated	UC	N	Hazard Ratio (95% CI)	P-value	Adjusted P-Value
DPP4i	GLP1ra	UC_1	655	1.2 (0.53-2.9)	6.28345e-01	9.425175e-01
DPP4i	GLP1ra	UC_2	1003	1.3 (0.67-2.7)	4.11752e-01	7.936818e-01
DPP4i	GLP1ra	UC_3	786	0.91 (0.45-1.9)	8.04378e-01	9.945010e-01
DPP4i	GLP1ra	UC_4	524	0.52 (0.13-2.1)	3.58212e-01	7.936818e-01
DPP4i	GLP1ra	UC_5	2550	0.98 (0.65-1.5)	9.13059e-01	9.945010e-01
DPP4i	SGLT2i	UC_1	987	0.48 (0.22-1)	5.43819e-02	5.438190e-01
DPP4i	SGLT2i	UC_2	1005	0.97 (0.47-2)	9.44997e-01	9.945010e-01
DPP4i	SGLT2i	UC_3	637	0.65 (0.27-1.6)	3.45334e-01	7.936818e-01
DPP4i	SGLT2i	UC_4	540	0.86 (0.26-2.8)	8.07790e-01	9.945010e-01
DPP4i	SGLT2i	UC_5	2882	0.56 (0.34-0.91)	2.07017e-02	5.438190e-01
GLP1ra	SGLT2i	UC_1	668	1.3 (0.5-3.2)	6.14236e-01	9.425175e-01
GLP1ra	SGLT2i	UC_2	1179	0.98 (0.46-2.1)	9.57922e-01	9.945010e-01
GLP1ra	SGLT2i	UC_3	745	1.3 (0.53-3.1)	5.69824e-01	9.425175e-01
GLP1ra	SGLT2i	UC_4	614	2 (0.37-11)	4.10683e-01	7.936818e-01
GLP1ra	SGLT2i	UC_5	2955	0.69 (0.44-1.1)	1.17060e-01	7.936818e-01
Sulfonylurea	DPP4i	UC_1	1630	1.2 (0.77-1.8)	4.49753e-01	7.936818e-01
Sulfonylurea	DPP4i	UC_2	1945	1 (0.69-1.5)	9.17320e-01	9.945010e-01
Sulfonylurea	DPP4i	UC_3	1937	1.2 (0.8-1.7)	4.22151e-01	7.936818e-01
Sulfonylurea	DPP4i	UC_4	1229	0.98 (0.54-1.8)	9.58799e-01	9.945010e-01
Sulfonylurea	DPP4i	UC_5	5579	0.99 (0.77-1.3)	9.15664e-01	9.945010e-01
Sulfonylurea	GLP1ra	UC_1	707	1.4 (0.59-3.3)	4.45410e-01	7.936818e-01
Sulfonylurea	GLP1ra	UC_2	1086	1 (0.52-1.9)	9.76863e-01	9.945010e-01
Sulfonylurea	GLP1ra	UC_3	982	0.66 (0.36-1.2)	1.91794e-01	7.936818e-01
Sulfonylurea	GLP1ra	UC_4	627	0.42 (0.08-2.2)	3.05777e-01	7.936818e-01
Sulfonylurea	GLP1ra	UC_5	2497	1.6 (1-2.6)	3.68865e-02	5.438190e-01
Sulfonylurea	SGLT2i	UC_1	1145	1.5 (0.68-3.4)	3.06974e-01	7.936818e-01
Sulfonylurea	SGLT2i	UC_2	1068	1.4 (0.69-2.9)	3.33583e-01	7.936818e-01
Sulfonylurea	SGLT2i	UC_3	870	1 (0.44-2.3)	9.94501e-01	9.945010e-01
Sulfonylurea	SGLT2i	UC_4	737	0.55 (0.2-1.5)	2.28941e-01	7.936818e-01
Sulfonylurea	SGLT2i	UC_5	2872	0.79 (0.47-1.3)	3.79488e-01	7.936818e-01

4.13.2 eFigure: Individual effect size, meta analysis and sensitivity analysis

The forest plot illustrate the effect size of the comparison between DPP4i and GLP1ra at each UC along with the effect size obtained from the random effect meta-analysis across all the UC for outcome Fracture Of Bone

© 2023 Vashisht R et al. *JAMA Network Open.*

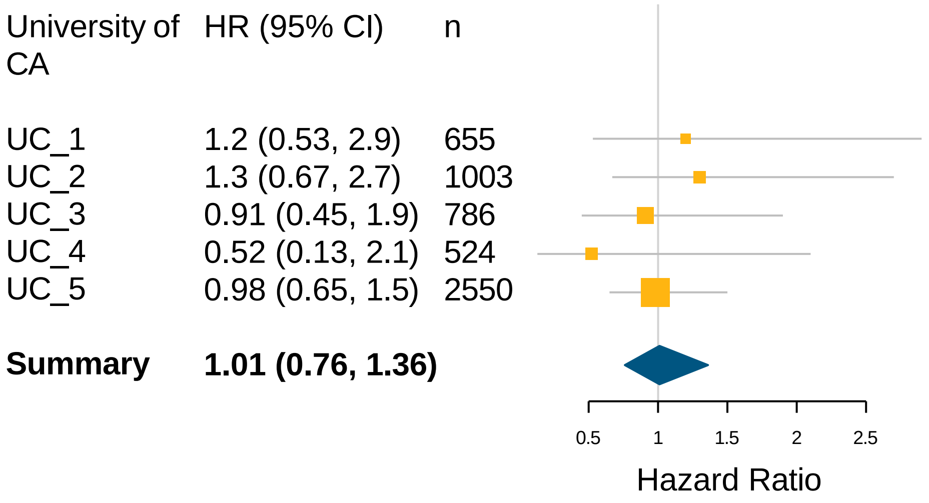

The table below shows the Leave-One-UC-Out diagnostics. The DFFITS value, Cook's distance, Covariance ratio, leave-one-out amount of heteroginity, indicator for influential estimates, comparator and treated groups are provided for each Leave-One-UC-Out analysis. The influential estimate from one UC with respect to pooled estimate are marked as Yes or No, with Yes indicating an influential UC and No otherwise.

eTable 87: Leave-One-UC-Out Sensitivity Analysis

DFFITs	Cook's Dist	Residual Heterogeneity	Influential	Comparator	Treated	UC
0.1526394	0.0232988	0	No	DPP4i	GLP1ra	UC_1
0.3582787	0.1283636	0	No	DPP4i	GLP1ra	UC_2
-0.1434682	0.0205831	0	No	DPP4i	GLP1ra	UC_3
-0.2076689	0.0431264	0	No	DPP4i	GLP1ra	UC_4
-0.2189988	0.0479605	0	No	DPP4i	GLP1ra	UC_5

© 2023 Vashisht R et al. *JAMA Network Open.*

The forest plot illustrate the effect size of the comparison between DPP4i and SGLT2i at each UC along with the effect size obtained from the random effect meta-analysis across all the UC for outcome Fracture Of Bone

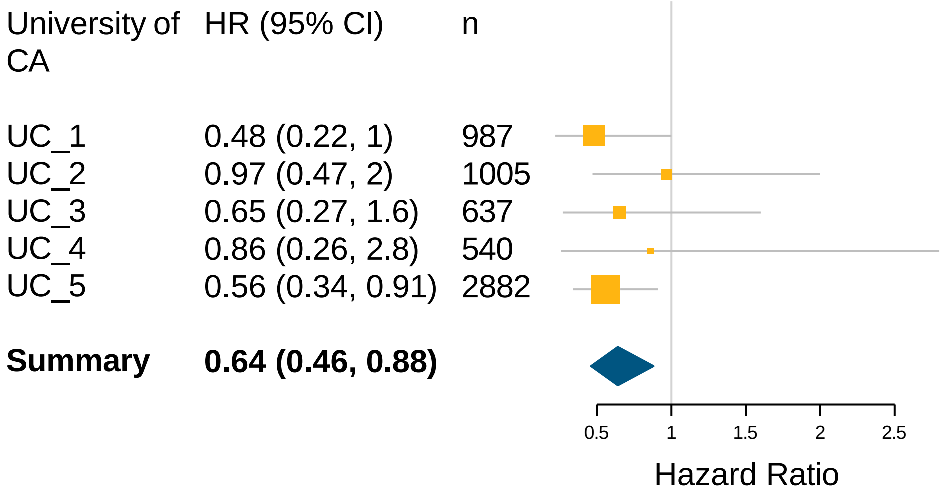

The table below shows the Leave-One-UC-Out diagnostics. The DFFITS value, Cook's distance, Covariance ratio, leave-one-out amount of heteroginity, indicator for influential estimates, comparator and treated groups are provided for each Leave-One-UC-Out analysis. The influential estimate from one UC with respect to pooled estimate are marked as Yes or No, with Yes indicating an influential UC and No otherwise.

eTable 88: Leave-One-UC-Out Sensitivity Analysis

DFFITs	Cook's Dist	Residual Heterogeneity	Influential	Comparator	Treated	UC
-0.3794602	0.1439900	0	No	DPP4i	SGLT2i	UC_1
0.6236177	0.3888990	0	No	DPP4i	SGLT2i	UC_2
0.0170296	0.0002900	0	No	DPP4i	SGLT2i	UC_3
0.1431388	0.0204887	0	No	DPP4i	SGLT2i	UC_4
-0.5854183	0.3427146	0	No	DPP4i	SGLT2i	UC_5

© 2023 Vashisht R et al. *JAMA Network Open.*

The forest plot illustrate the effect size of the comparison between GLP1ra and SGLT2i at each UC along with the effect size obtained from the random effect meta-analysis across all the UC for outcome Fracture Of Bone

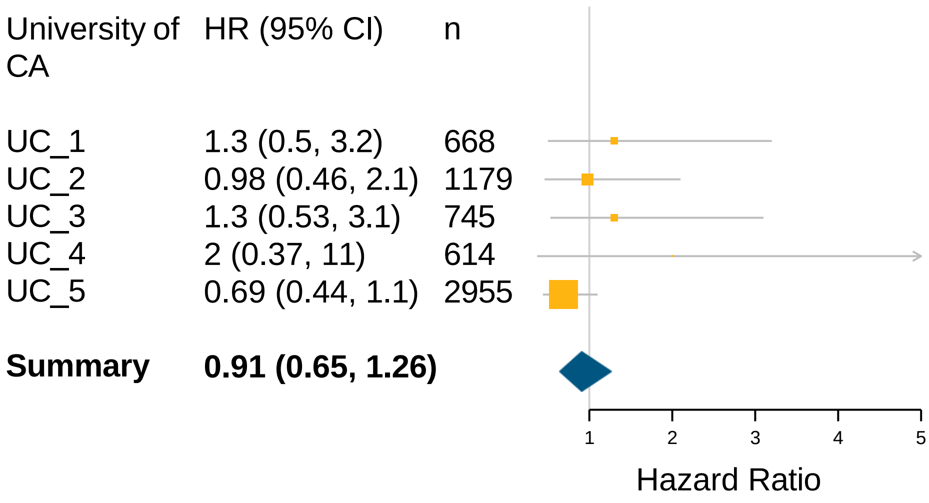

The table below shows the Leave-One-UC-Out diagnostics. The DFFITS value, Cook's distance, Covariance ratio, leave-one-out amount of heteroginity, indicator for influential estimates, comparator and treated groups are provided for each Leave-One-UC-Out analysis. The influential estimate from one UC with respect to pooled estimate are marked as Yes or No, with Yes indicating an influential UC and No otherwise.

eTable 89: Leave-One-UC-Out Sensitivity Analysis

DFFITs	Cook's Dist	Residual Heterogeneity	Influential	Comparator	Treated	UC
0.3083888	0.0951037	0.0000000	No	GLP1ra	SGLT2i	UC_1
-0.1659307	0.0322467	0.0256897	No	GLP1ra	SGLT2i	UC_2
0.3457850	0.1195673	0.0000000	No	GLP1ra	SGLT2i	UC_3
0.1839948	0.0338541	0.0000000	No	GLP1ra	SGLT2i	UC_4
-1.7101341	2.9245586	0.0000000	Yes	GLP1ra	SGLT2i	UC_5

© 2023 Vashisht R et al. *JAMA Network Open.*

The forest plot illustrate the effect size of the comparison between Sulfonylurea and DPP4i at each UC along with the effect size obtained from the random effect meta-analysis across all the UC for outcome Fracture Of Bone

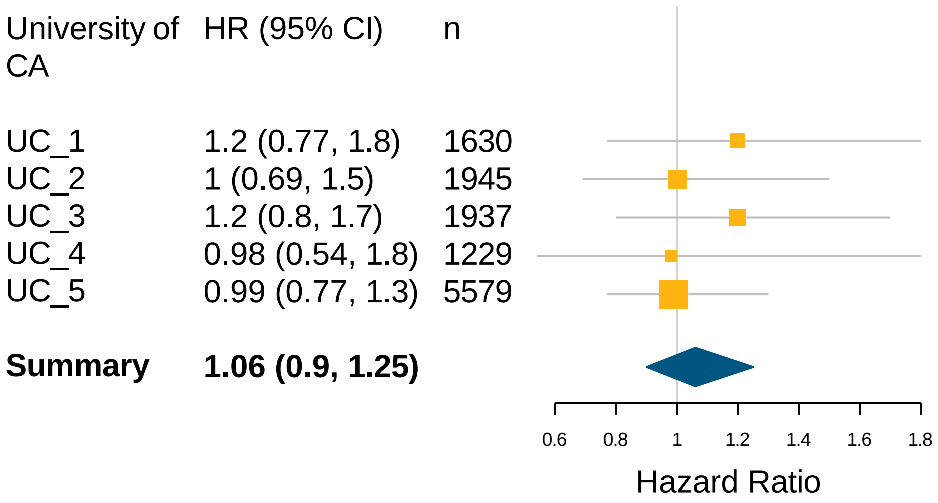

The table below shows the Leave-One-UC-Out diagnostics. The DFFITS value, Cook's distance, Covariance ratio, leave-one-out amount of heteroginity, indicator for influential estimates, comparator and treated groups are provided for each Leave-One-UC-Out analysis. The influential estimate from one UC with respect to pooled estimate are marked as Yes or No, with Yes indicating an influential UC and No otherwise.

eTable 90: Leave-One-UC-Out Sensitivity Analysis

DFFITs	Cook's Dist	Residual Heterogeneity	Influential	Comparator	Treated	UC
0.2651488	0.0703039	0	No	Sulfonylurea	DPP4i	UC_1
-0.1503356	0.0226008	0	No	Sulfonylurea	DPP4i	UC_2
0.3535096	0.1249690	0	No	Sulfonylurea	DPP4i	UC_3
-0.0749161	0.0056124	0	No	Sulfonylurea	DPP4i	UC_4
-0.5290447	0.2798883	0	No	Sulfonylurea	DPP4i	UC_5

© 2023 Vashisht R et al. *JAMA Network Open.*

The forest plot illustrate the effect size of the comparison between Sulfonylurea and GLP1ra at each UC along with the effect size obtained from the random effect meta-analysis across all the UC for outcome Fracture Of Bone

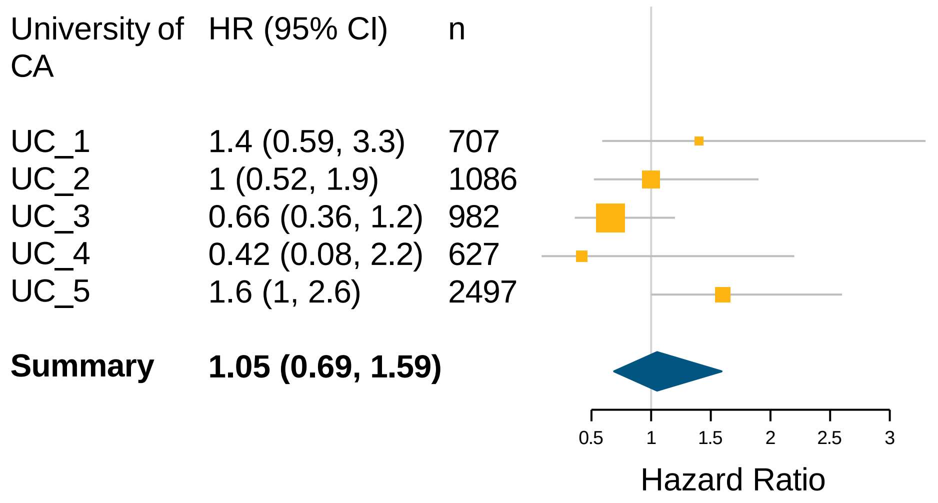

The table below shows the Leave-One-UC-Out diagnostics. The DFFITS value, Cook's distance, Covariance ratio, leave-one-out amount of heteroginity, indicator for influential estimates, comparator and treated groups are provided for each Leave-One-UC-Out analysis. The influential estimate from one UC with respect to pooled estimate are marked as Yes or No, with Yes indicating an influential UC and No otherwise.

eTable 91: Leave-One-UC-Out Sensitivity Analysis

DFFITs	Cook's Dist	Residual Heterogeneity	Influential	Comparator	Treated	UC
0.3242801	0.1227426	0.1373861	No	Sulfonylurea	GLP1ra	UC_1
0.0456245	0.0029471	0.1729532	No	Sulfonylurea	GLP1ra	UC_2
-1.2665263	0.9041694	0.0096072	Yes	Sulfonylurea	GLP1ra	UC_3
-0.2642290	0.0692384	0.0834878	No	Sulfonylurea	GLP1ra	UC_4
1.4313054	0.8142827	0.0000000	Yes	Sulfonylurea	GLP1ra	UC_5

© 2023 Vashisht R et al. *JAMA Network Open.*

The forest plot illustrate the effect size of the comparison between Sulfonylurea and SGLT2i at each UC along with the effect size obtained from the random effect meta-analysis across all the UC for outcome Fracture Of Bone

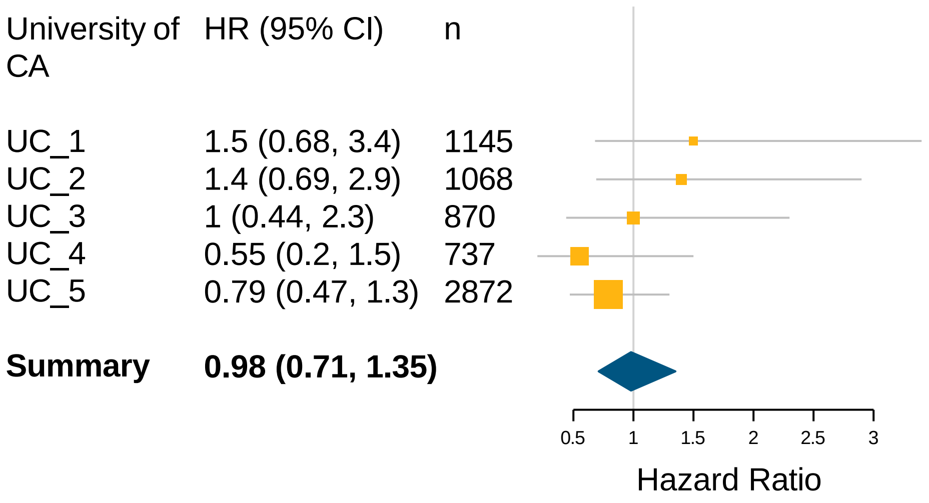

The table below shows the Leave-One-UC-Out diagnostics. The DFFITS value, Cook's distance, Covariance ratio, leave-one-out amount of heteroginity, indicator for influential estimates, comparator and treated groups are provided for each Leave-One-UC-Out analysis. The influential estimate from one UC with respect to pooled estimate are marked as Yes or No, with Yes indicating an influential UC and No otherwise.

eTable 92: Leave-One-UC-Out Sensitivity Analysis

DFFITs	Cook's Dist	Residual Heterogeneity	Influential	Comparator	Treated	UC
0.4913367	0.2414118	0.0000000	No	Sulfonylurea	SGLT2i	UC_1
0.5439805	0.2959148	0.0000000	No	Sulfonylurea	SGLT2i	UC_2
-0.0609280	0.0046420	0.0445862	No	Sulfonylurea	SGLT2i	UC_3
-0.3946172	0.1557228	0.0000000	No	Sulfonylurea	SGLT2i	UC_4
-0.8518879	0.7257130	0.0000000	Yes	Sulfonylurea	SGLT2i	UC_5

© 2023 Vashisht R et al. *JAMA Network Open.*

4.14 Glaucoma

4.14.1 eTable: Drug comparision table

Effect size of each drug comparison at each UC health site is tabulated.

eTable 93: Hazard ratios of drug class comparison at each UC

Comparator	Treated	UC	N	Hazard Ratio (95% CI)	P-value	Adjusted P-Value
DPP4i	GLP1ra	UC_1	642	1.6 (0.65-4.2)	2.98131e-01	6.388521e-01
DPP4i	GLP1ra	UC_2	1029	0.87 (0.38-2)	7.44645e-01	8.218428e-01
DPP4i	GLP1ra	UC_3	818	0.6 (0.25-1.5)	2.63524e-01	6.081323e-01
DPP4i	GLP1ra	UC_4	519	0.49 (0.09-2.7)	4.12678e-01	7.501236e-01
DPP4i	GLP1ra	UC_5	2567	0.91 (0.52-1.6)	7.46938e-01	8.218428e-01
DPP4i	SGLT2i	UC_1	968	0.92 (0.41-2.1)	8.48793e-01	8.487930e-01
DPP4i	SGLT2i	UC_2	1023	0.54 (0.24-1.2)	1.25194e-01	3.714525e-01
DPP4i	SGLT2i	UC_3	668	0.62 (0.2-1.9)	4.08777e-01	7.501236e-01
DPP4i	SGLT2i	UC_4	519	0.71 (0.2-2.5)	6.03310e-01	7.501236e-01
DPP4i	SGLT2i	UC_5	2835	1.5 (0.87-2.6)	1.42223e-01	3.714525e-01
GLP1ra	SGLT2i	UC_1	666	0.78 (0.33-1.9)	5.76881e-01	7.501236e-01
GLP1ra	SGLT2i	UC_2	1230	0.81 (0.34-1.9)	6.25103e-01	7.501236e-01
GLP1ra	SGLT2i	UC_3	771	0.86 (0.29-2.6)	7.94448e-01	8.218428e-01
GLP1ra	SGLT2i	UC_4	607	5.5 (0.64-48)	1.18706e-01	3.714525e-01
GLP1ra	SGLT2i	UC_5	2934	1.1 (0.67-1.9)	6.23802e-01	7.501236e-01
Sulfonylurea	DPP4i	UC_1	1577	1.5 (0.86-2.6)	1.48581e-01	3.714525e-01
Sulfonylurea	DPP4i	UC_2	1990	1.2 (0.72-1.8)	5.50812e-01	7.501236e-01
Sulfonylurea	DPP4i	UC_3	1981	1.2 (0.75-2)	4.30872e-01	7.501236e-01
Sulfonylurea	DPP4i	UC_4	1192	0.62 (0.33-1.2)	1.34660e-01	3.714525e-01
Sulfonylurea	DPP4i	UC_5	5439	0.67 (0.51-0.87)	2.97714e-03	8.931420e-02
Sulfonylurea	GLP1ra	UC_1	700	2.9 (0.91-9)	7.28902e-02	3.714525e-01
Sulfonylurea	GLP1ra	UC_2	1122	0.74 (0.33-1.7)	4.63909e-01	7.501236e-01
Sulfonylurea	GLP1ra	UC_3	1024	0.76 (0.32-1.8)	5.49655e-01	7.501236e-01
Sulfonylurea	GLP1ra	UC_4	608	0.26 (0.06-1.2)	8.61251e-02	3.714525e-01
Sulfonylurea	GLP1ra	UC_5	2480	0.67 (0.4-1.1)	1.29498e-01	3.714525e-01
Sulfonylurea	SGLT2i	UC_1	1126	1.2 (0.54-2.9)	6.03037e-01	7.501236e-01
Sulfonylurea	SGLT2i	UC_2	1084	0.53 (0.24-1.2)	1.18838e-01	3.714525e-01
Sulfonylurea	SGLT2i	UC_3	910	0.86 (0.29-2.6)	7.94378e-01	8.218428e-01
Sulfonylurea	SGLT2i	UC_4	697	0.32 (0.12-0.86)	2.45105e-02	3.676575e-01
Sulfonylurea	SGLT2i	UC_5	2812	0.64 (0.4-1)	6.35295e-02	3.714525e-01

4.14.2 eFigure: Individual effect size, meta analysis and sensitivity analysis

The forest plot illustrate the effect size of the comparison between DPP4i and GLP1ra at each UC along with the effect size obtained from the random effect meta-analysis across all the UC for outcome Glaucoma

© 2023 Vashisht R et al. *JAMA Network Open.*

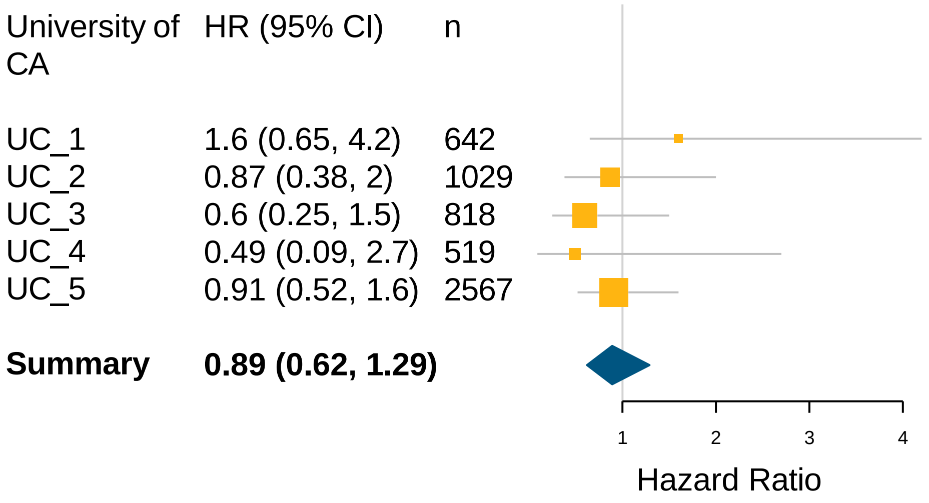

The table below shows the Leave-One-UC-Out diagnostics. The DFFITS value, Cook's distance, Covariance ratio, leave-one-out amount of heteroginity, indicator for influential estimates, comparator and treated groups are provided for each Leave-One-UC-Out analysis. The influential estimate from one UC with respect to pooled estimate are marked as Yes or No, with Yes indicating an influential UC and No otherwise.

eTable 94: Leave-One-UC-Out Sensitivity Analysis

DFFITs	Cook's Dist	Residual Heterogeneity	Influential	Comparator	Treated	UC
0.5752264	0.3308855	0	No	DPP4i	GLP1ra	UC_1
-0.0320915	0.0010299	0	No	DPP4i	GLP1ra	UC_2
-0.4293607	0.1843506	0	No	DPP4i	GLP1ra	UC_3
-0.1569551	0.0246349	0	No	DPP4i	GLP1ra	UC_4
0.0818016	0.0066915	0	No	DPP4i	GLP1ra	UC_5

© 2023 Vashisht R et al. *JAMA Network Open.*

The forest plot illustrate the effect size of the comparison between DPP4i and SGLT2i at each UC along with the effect size obtained from the random effect meta-analysis across all the UC for outcome Glaucoma

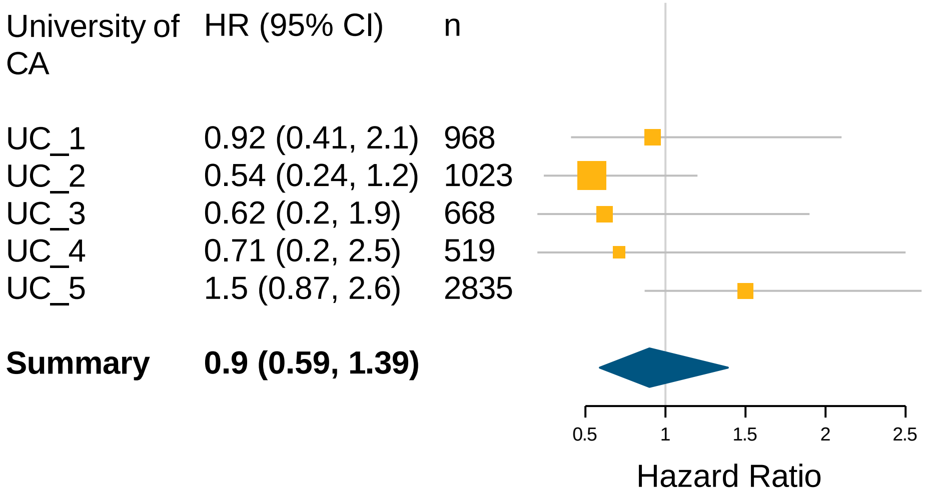

The table below shows the Leave-One-UC-Out diagnostics. The DFFITS value, Cook's distance, Covariance ratio, leave-one-out amount of heteroginity, indicator for influential estimates, comparator and treated groups are provided for each Leave-One-UC-Out analysis. The influential estimate from one UC with respect to pooled estimate are marked as Yes or No, with Yes indicating an influential UC and No otherwise.

eTable 95: Leave-One-UC-Out Sensitivity Analysis

DFFITs	Cook's Dist	Residual Heterogeneity	Influential	Comparator	Treated	UC
0.2072679	0.0593820	0.1509509	No	DPP4i	SGLT2i	UC_1
-1.0703329	0.8405549	0.0000000	Yes	DPP4i	SGLT2i	UC_2
-0.1645749	0.0292928	0.0930555	No	DPP4i	SGLT2i	UC_3
-0.0122554	0.0001660	0.1112740	No	DPP4i	SGLT2i	UC_4
1.6502872	1.5262880	0.0000000	Yes	DPP4i	SGLT2i	UC_5

© 2023 Vashisht R et al. *JAMA Network Open.*

The forest plot illustrate the effect size of the comparison between GLP1ra and SGLT2i at each UC along with the effect size obtained from the random effect meta-analysis across all the UC for outcome Glaucoma

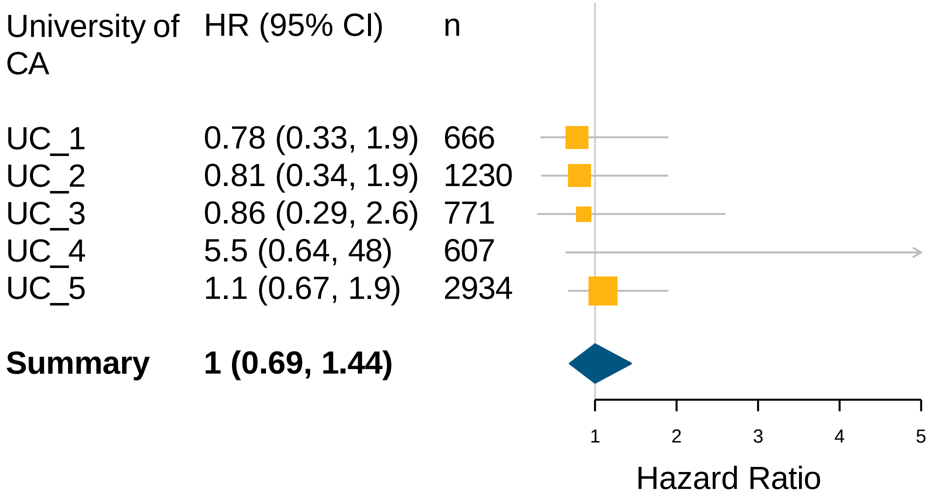

The table below shows the Leave-One-UC-Out diagnostics. The DFFITS value, Cook's distance, Covariance ratio, leave-one-out amount of heteroginity, indicator for influential estimates, comparator and treated groups are provided for each Leave-One-UC-Out analysis. The influential estimate from one UC with respect to pooled estimate are marked as Yes or No, with Yes indicating an influential UC and No otherwise.

eTable 96: Leave-One-UC-Out Sensitivity Analysis

DFFITs	Cook's Dist	Residual Heterogeneity	Influential	Comparator	Treated	UC
-0.2814212	0.0791979	0.0000000	No	GLP1ra	SGLT2i	UC_1
-0.2484644	0.0617346	0.0000000	No	GLP1ra	SGLT2i	UC_2
-0.0967426	0.0094723	0.0037864	No	GLP1ra	SGLT2i	UC_3
0.2721291	0.0740543	0.0000000	No	GLP1ra	SGLT2i	UC_4
0.5174771	0.2677826	0.0000000	No	GLP1ra	SGLT2i	UC_5

© 2023 Vashisht R et al. *JAMA Network Open.*

The forest plot illustrate the effect size of the comparison between Sulfonylurea and DPP4i at each UC along with the effect size obtained from the random effect meta-analysis across all the UC for outcome Glaucoma

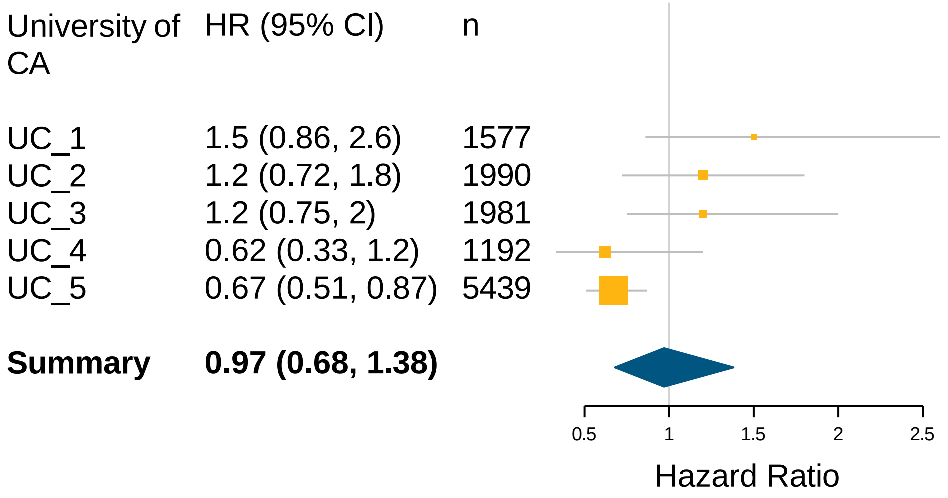

The table below shows the Leave-One-UC-Out diagnostics. The DFFITS value, Cook's distance, Covariance ratio, leave-one-out amount of heteroginity, indicator for influential estimates, comparator and treated groups are provided for each Leave-One-UC-Out analysis. The influential estimate from one UC with respect to pooled estimate are marked as Yes or No, with Yes indicating an influential UC and No otherwise.

eTable 97: Leave-One-UC-Out Sensitivity Analysis

DFFITs	Cook's Dist	Residual Heterogeneity	Influential	Comparator	Treated	UC
0.5801562	0.2915912	0.0806097	No	Sulfonylurea	DPP4i	UC_1
0.2763763	0.0857709	0.1249973	No	Sulfonylurea	DPP4i	UC_2
0.2611696	0.0763770	0.1254470	No	Sulfonylurea	DPP4i	UC_3
-0.4541515	0.2214194	0.1210581	No	Sulfonylurea	DPP4i	UC_4
-1.2141140	0.6404565	0.0352690	Yes	Sulfonylurea	DPP4i	UC_5

© 2023 Vashisht R et al. *JAMA Network Open.*

The forest plot illustrate the effect size of the comparison between Sulfonylurea and GLP1ra at each UC along with the effect size obtained from the random effect meta-analysis across all the UC for outcome Glaucoma

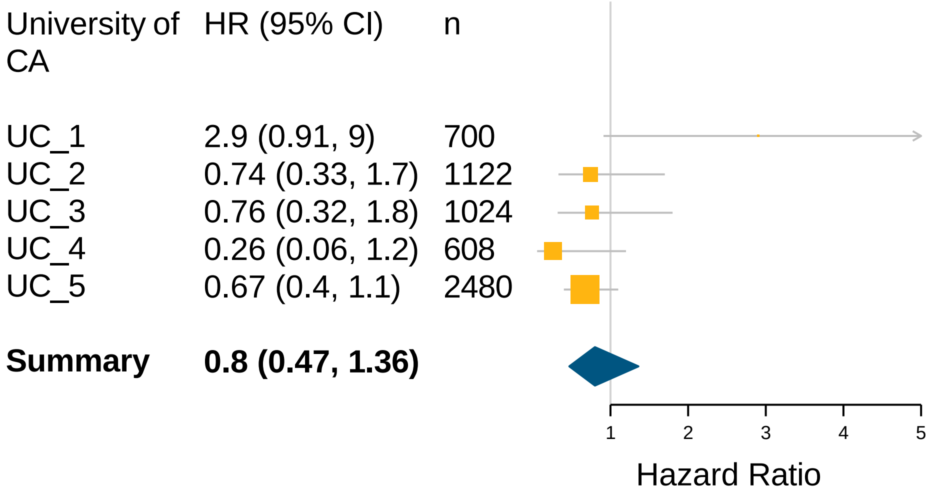

The table below shows the Leave-One-UC-Out diagnostics. The DFFITS value, Cook's distance, Covariance ratio, leave-one-out amount of heteroginity, indicator for influential estimates, comparator and treated groups are provided for each Leave-One-UC-Out analysis. The influential estimate from one UC with respect to pooled estimate are marked as Yes or No, with Yes indicating an influential UC and No otherwise.

eTable 98: Leave-One-UC-Out Sensitivity Analysis

DFFITs	Cook's Dist	Residual Heterogeneity	Influential	Comparator	Treated	UC
0.8277961	0.4650573	0.0000000	Yes	Sulfonylurea	GLP1ra	UC_1
-0.0892141	0.0114370	0.3089242	No	Sulfonylurea	GLP1ra	UC_2
-0.0643039	0.0057113	0.2975009	No	Sulfonylurea	GLP1ra	UC_3
-0.3959582	0.1498130	0.1271152	No	Sulfonylurea	GLP1ra	UC_4
-0.2053118	0.0777172	0.3545034	No	Sulfonylurea	GLP1ra	UC_5

© 2023 Vashisht R et al. *JAMA Network Open.*

The forest plot illustrate the effect size of the comparison between Sulfonylurea and SGLT2i at each UC along with the effect size obtained from the random effect meta-analysis across all the UC for outcome Glaucoma

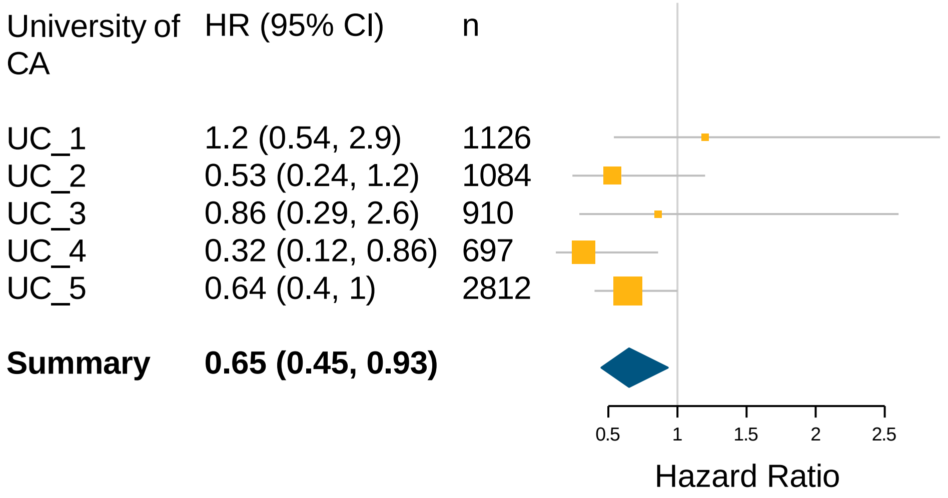

The table below shows the Leave-One-UC-Out diagnostics. The DFFITS value, Cook's distance, Covariance ratio, leave-one-out amount of heteroginity, indicator for influential estimates, comparator and treated groups are provided for each Leave-One-UC-Out analysis. The influential estimate from one UC with respect to pooled estimate are marked as Yes or No, with Yes indicating an influential UC and No otherwise.

eTable 99: Leave-One-UC-Out Sensitivity Analysis

DFFITs	Cook's Dist	Residual Heterogeneity	Influential	Comparator	Treated	UC
0.6195072	0.3441345	0.0000000	No	Sulfonylurea	SGLT2i	UC_1
-0.2348981	0.0688685	0.0682749	No	Sulfonylurea	SGLT2i	UC_2
0.1910195	0.0406247	0.0590817	No	Sulfonylurea	SGLT2i	UC_3
-0.4823314	0.2146280	0.0000000	No	Sulfonylurea	SGLT2i	UC_4
-0.0232492	0.0011889	0.1121452	No	Sulfonylurea	SGLT2i	UC_5

© 2023 Vashisht R et al. *JAMA Network Open.*

4.15 Headache

4.15.1 eTable: Drug comparision table

Effect size of each drug comparison at each UC health site is tabulated.

eTable 100: Hazard ratios of drug class comparison at each UC

Comparator	Treated	UC	N	Hazard Ratio (95% CI)	P-value	Adjusted P-Value
DPP4i	GLP1ra	UC_1	608	2 (0.93-4.3)	7.71437e-02	9.517159e-01
DPP4i	GLP1ra	UC_2	927	1 (0.61-1.7)	8.95390e-01	9.517159e-01
DPP4i	GLP1ra	UC_3	701	0.9 (0.51-1.6)	7.12801e-01	9.517159e-01
DPP4i	GLP1ra	UC_4	485	0.93 (0.34-2.6)	8.93897e-01	9.517159e-01
DPP4i	GLP1ra	UC_5	2414	1 (0.72-1.4)	9.19992e-01	9.517159e-01
DPP4i	SGLT2i	UC_1	934	0.73 (0.39-1.4)	3.26057e-01	9.517159e-01
DPP4i	SGLT2i	UC_2	956	1.3 (0.68-2.3)	4.55803e-01	9.517159e-01
DPP4i	SGLT2i	UC_3	574	0.9 (0.42-2)	7.97857e-01	9.517159e-01
DPP4i	SGLT2i	UC_4	503	1.3 (0.39-4.2)	6.96521e-01	9.517159e-01
DPP4i	SGLT2i	UC_5	2717	0.72 (0.49-1.1)	1.01497e-01	9.517159e-01
GLP1ra	SGLT2i	UC_1	605	0.76 (0.35-1.7)	4.89036e-01	9.517159e-01
GLP1ra	SGLT2i	UC_2	1091	0.87 (0.52-1.4)	5.86834e-01	9.517159e-01
GLP1ra	SGLT2i	UC_3	654	0.82 (0.4-1.7)	5.94866e-01	9.517159e-01
GLP1ra	SGLT2i	UC_4	565	0.76 (0.26-2.2)	6.08304e-01	9.517159e-01
GLP1ra	SGLT2i	UC_5	2754	1 (0.71-1.5)	8.81663e-01	9.517159e-01
Sulfonylurea	DPP4i	UC_1	1558	0.95 (0.63-1.4)	7.86320e-01	9.517159e-01
Sulfonylurea	DPP4i	UC_2	1872	1.1 (0.76-1.6)	6.20962e-01	9.517159e-01
Sulfonylurea	DPP4i	UC_3	1812	0.99 (0.71-1.4)	9.63863e-01	9.638630e-01
Sulfonylurea	DPP4i	UC_4	1188	1.5 (0.84-2.7)	1.64554e-01	9.517159e-01
Sulfonylurea	DPP4i	UC_5	5385	0.97 (0.77-1.2)	7.95417e-01	9.517159e-01
Sulfonylurea	GLP1ra	UC_1	657	1.6 (0.82-3.2)	1.69231e-01	9.517159e-01
Sulfonylurea	GLP1ra	UC_2	1007	1.2 (0.71-2)	4.98195e-01	9.517159e-01
Sulfonylurea	GLP1ra	UC_3	903	1.1 (0.61-1.8)	8.55017e-01	9.517159e-01
Sulfonylurea	GLP1ra	UC_4	584	1.1 (0.41-2.9)	8.46680e-01	9.517159e-01
Sulfonylurea	GLP1ra	UC_5	2365	1 (0.71-1.5)	8.49256e-01	9.517159e-01
Sulfonylurea	SGLT2i	UC_1	1082	0.66 (0.36-1.2)	1.68791e-01	9.517159e-01
Sulfonylurea	SGLT2i	UC_2	1020	1.5 (0.82-2.7)	1.93377e-01	9.517159e-01
Sulfonylurea	SGLT2i	UC_3	804	0.87 (0.47-1.6)	6.69381e-01	9.517159e-01
Sulfonylurea	SGLT2i	UC_4	693	1.6 (0.46-5.8)	4.50605e-01	9.517159e-01
Sulfonylurea	SGLT2i	UC_5	2724	0.95 (0.63-1.4)	7.95521e-01	9.517159e-01

4.15.2 eFigure: Individual effect size, meta analysis and sensitivity analysis

The forest plot illustrate the effect size of the comparison between DPP4i and GLP1ra at each UC along with the effect size obtained from the random effect meta-analysis across all the UC for outcome Headache

© 2023 Vashisht R et al. *JAMA Network Open.*

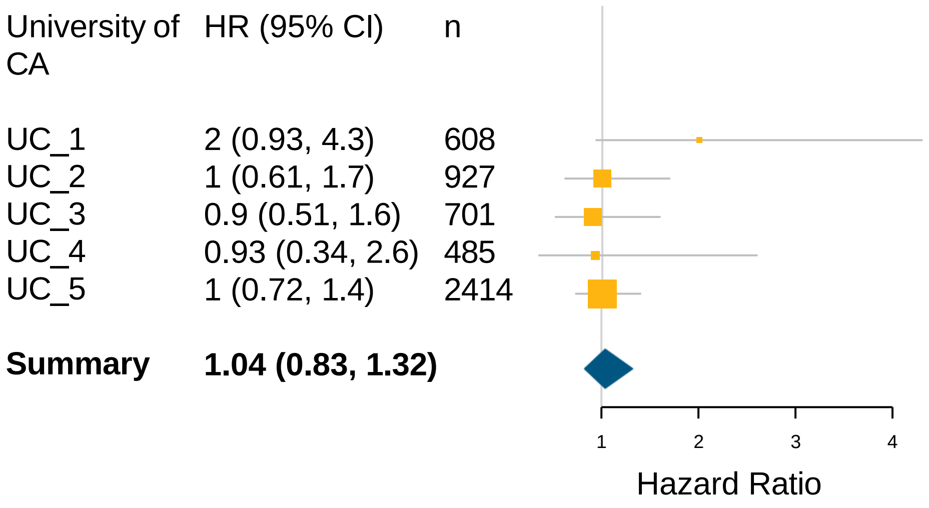

The table below shows the Leave-One-UC-Out diagnostics. The DFFITS value, Cook's distance, Covariance ratio, leave-one-out amount of heteroginity, indicator for influential estimates, comparator and treated groups are provided for each Leave-One-UC-Out analysis. The influential estimate from one UC with respect to pooled estimate are marked as Yes or No, with Yes indicating an influential UC and No otherwise.

eTable 101: Leave-One-UC-Out Sensitivity Analysis

DFFITs	Cook's Dist	Residual Heterogeneity	Influential	Comparator	Treated	UC
0.5556664	0.3087651	0.0000000	No	DPP4i	GLP1ra	UC_1
-0.1251367	0.0166352	0.0042609	No	DPP4i	GLP1ra	UC_2
-0.2462362	0.0606323	0.0000000	No	DPP4i	GLP1ra	UC_3
-0.0734049	0.0054419	0.0026816	No	DPP4i	GLP1ra	UC_4
-0.3380764	0.1217050	0.0018655	No	DPP4i	GLP1ra	UC_5

© 2023 Vashisht R et al. *JAMA Network Open.*

The forest plot illustrate the effect size of the comparison between DPP4i and SGLT2i at each UC along with the effect size obtained from the random effect meta-analysis across all the UC for outcome Headache

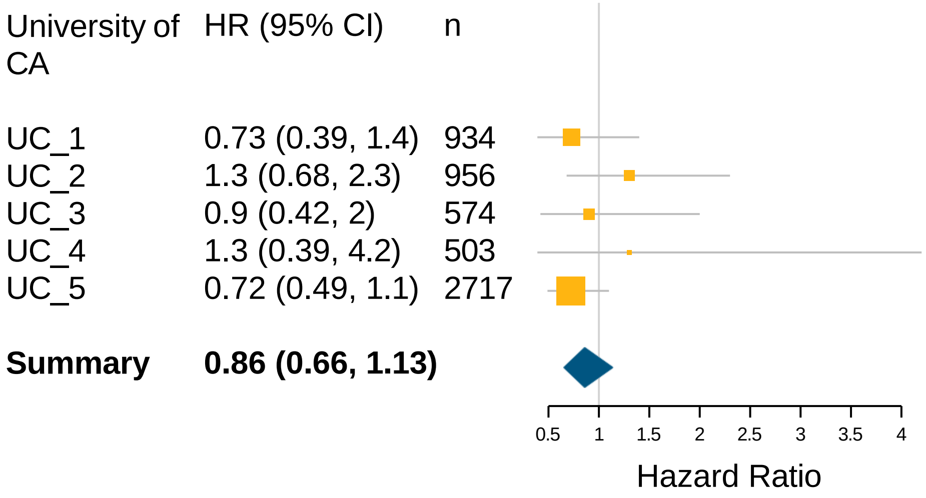

The table below shows the Leave-One-UC-Out diagnostics. The DFFITS value, Cook's distance, Covariance ratio, leave-one-out amount of heteroginity, indicator for influential estimates, comparator and treated groups are provided for each Leave-One-UC-Out analysis. The influential estimate from one UC with respect to pooled estimate are marked as Yes or No, with Yes indicating an influential UC and No otherwise.

eTable 102: Leave-One-UC-Out Sensitivity Analysis

DFFITs	Cook's Dist	Residual Heterogeneity	Influential	Comparator	Treated	UC
-0.2590719	0.0671182	0.0000000	No	DPP4i	SGLT2i	UC_1
0.7382831	0.5450620	0.0000000	Yes	DPP4i	SGLT2i	UC_2
-0.0182460	0.0003488	0.0075592	No	DPP4i	SGLT2i	UC_3
0.1642095	0.0269647	0.0000000	No	DPP4i	SGLT2i	UC_4
-1.0453754	1.0928098	0.0000000	Yes	DPP4i	SGLT2i	UC_5

© 2023 Vashisht R et al. *JAMA Network Open.*

The forest plot illustrate the effect size of the comparison between GLP1ra and SGLT2i at each UC along with the effect size obtained from the random effect meta-analysis across all the UC for outcome Headache

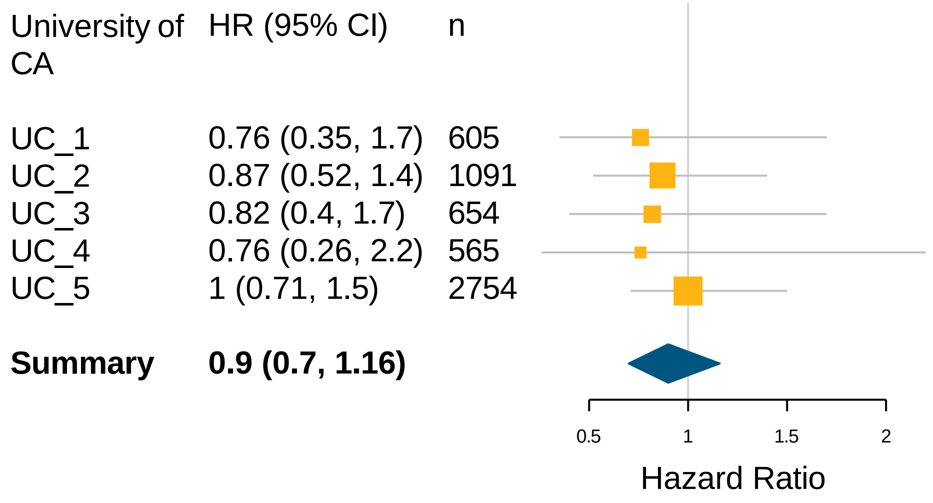

The table below shows the Leave-One-UC-Out diagnostics. The DFFITS value, Cook's distance, Covariance ratio, leave-one-out amount of heteroginity, indicator for influential estimates, comparator and treated groups are provided for each Leave-One-UC-Out analysis. The influential estimate from one UC with respect to pooled estimate are marked as Yes or No, with Yes indicating an influential UC and No otherwise.

eTable 103: Leave-One-UC-Out Sensitivity Analysis

DFFITs	Cook's Dist	Residual Heterogeneity	Influential	Comparator	Treated	UC
-0.1506583	0.0226979	0	No	GLP1ra	SGLT2i	UC_1
-0.0959817	0.0092125	0	No	GLP1ra	SGLT2i	UC_2
-0.1017638	0.0103559	0	No	GLP1ra	SGLT2i	UC_3
-0.0784622	0.0061563	0	No	GLP1ra	SGLT2i	UC_4
0.6815232	0.4644739	0	Yes	GLP1ra	SGLT2i	UC_5

© 2023 Vashisht R et al. *JAMA Network Open.*

The forest plot illustrate the effect size of the comparison between Sulfonylurea and DPP4i at each UC along with the effect size obtained from the random effect meta-analysis across all the UC for outcome Headache

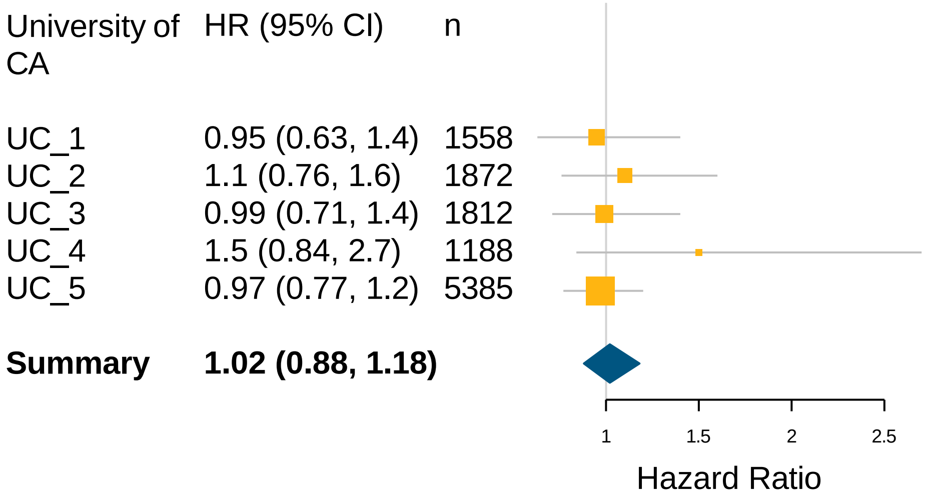

The table below shows the Leave-One-UC-Out diagnostics. The DFFITS value, Cook's distance, Covariance ratio, leave-one-out amount of heteroginity, indicator for influential estimates, comparator and treated groups are provided for each Leave-One-UC-Out analysis. The influential estimate from one UC with respect to pooled estimate are marked as Yes or No, with Yes indicating an influential UC and No otherwise.

eTable 104: Leave-One-UC-Out Sensitivity Analysis

DFFITs	Cook's Dist	Residual Heterogeneity	Influential	Comparator	Treated	UC
-0.1481726	0.0219551	0	No	Sulfonylurea	DPP4i	UC_1
0.1911711	0.0365464	0	No	Sulfonylurea	DPP4i	UC_2
-0.0897012	0.0080463	0	No	Sulfonylurea	DPP4i	UC_3
0.3527984	0.1244667	0	No	Sulfonylurea	DPP4i	UC_4
-0.5257012	0.2763618	0	No	Sulfonylurea	DPP4i	UC_5

The forest plot illustrate the effect size of the comparison between Sulfonylurea and GLP1ra at each UC along with the effect size obtained from the random effect meta-analysis across all the UC for outcome Headache

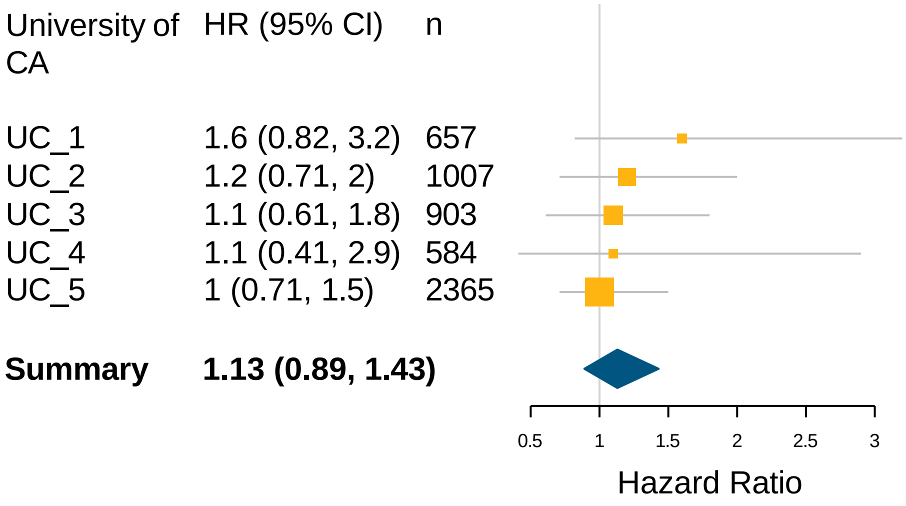

The table below shows the Leave-One-UC-Out diagnostics. The DFFITS value, Cook's distance, Covariance ratio, leave-one-out amount of heteroginity, indicator for influential estimates, comparator and treated groups are provided for each Leave-One-UC-Out analysis. The influential estimate from one UC with respect to pooled estimate are marked as Yes or No, with Yes indicating an influential UC and No otherwise.

eTable 105: Leave-One-UC-Out Sensitivity Analysis

DFFITs	Cook's Dist	Residual Heterogeneity	Influential	Comparator	Treated	UC
0.4023013	0.1618463	0	No	Sulfonylurea	GLP1ra	UC_1
0.1359335	0.0184779	0	No	Sulfonylurea	GLP1ra	UC_2
-0.0513130	0.0026330	0	No	Sulfonylurea	GLP1ra	UC_3
-0.0134368	0.0001805	0	No	Sulfonylurea	GLP1ra	UC_4
-0.6860639	0.4706837	0	Yes	Sulfonylurea	GLP1ra	UC_5

© 2023 Vashisht R et al. *JAMA Network Open.*

The forest plot illustrate the effect size of the comparison between Sulfonylurea and SGLT2i at each UC along with the effect size obtained from the random effect meta-analysis across all the UC for outcome Headache

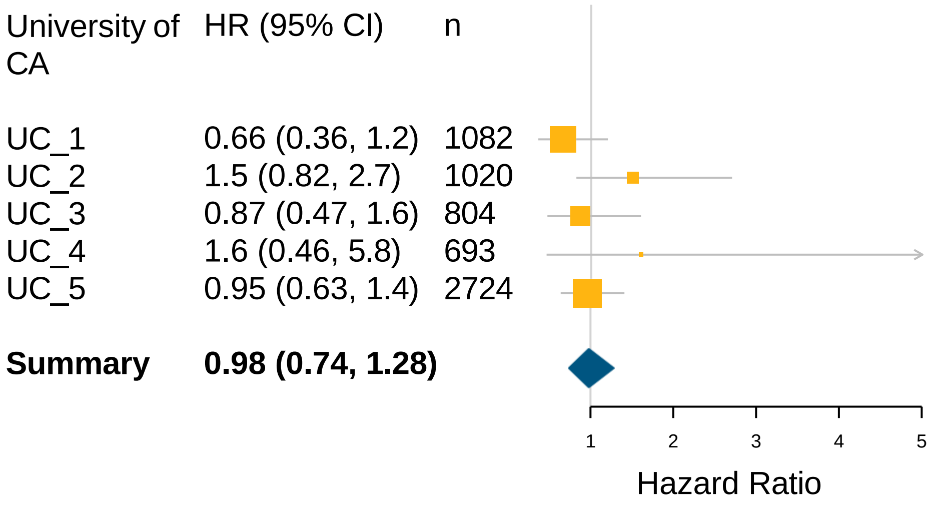

The table below shows the Leave-One-UC-Out diagnostics. The DFFITS value, Cook's distance, Covariance ratio, leave-one-out amount of heteroginity, indicator for influential estimates, comparator and treated groups are provided for each Leave-One-UC-Out analysis. The influential estimate from one UC with respect to pooled estimate are marked as Yes or No, with Yes indicating an influential UC and No otherwise.

eTable 106: Leave-One-UC-Out Sensitivity Analysis

DFFITs	Cook's Dist	Residual Heterogeneity	Influential	Comparator	Treated	UC
-0.6319953	0.3665594	0.0000000	No	Sulfonylurea	SGLT2i	UC_1
0.7570740	0.5251151	0.0000000	Yes	Sulfonylurea	SGLT2i	UC_2
-0.2312670	0.0688151	0.0388745	No	Sulfonylurea	SGLT2i	UC_3
0.1680850	0.0289420	0.0188627	No	Sulfonylurea	SGLT2i	UC_4
-0.1380169	0.0369460	0.0553892	No	Sulfonylurea	SGLT2i	UC_5

© 2023 Vashisht R et al. *JAMA Network Open.*

4.16 Heart Failure

4.16.1 eTable: Drug comparision table

Effect size of each drug comparison at each UC health site is tabulated.

eTable 107: Hazard ratios of drug class comparison at each UC

Comparator	Treated	UC	N	Hazard Ratio (95% CI)	P-value	Adjusted P-Value
DPP4i	GLP1ra	UC_1	673	1.9 (0.63-5.6)	2.57899e-01	4.551159e-01
DPP4i	GLP1ra	UC_2	993	1.2 (0.64-2.3)	5.39633e-01	8.993883e-01
DPP4i	GLP1ra	UC_3	810	0.45 (0.22-0.94)	3.28175e-02	4.550446e-01
DPP4i	GLP1ra	UC_4	522	0.41 (0.13-1.3)	1.32263e-01	4.550446e-01
DPP4i	GLP1ra	UC_5	2612	0.98 (0.59-1.6)	9.30291e-01	9.302910e-01
DPP4i	SGLT2i	UC_1	984	1.8 (0.73-4.7)	1.97186e-01	4.550446e-01
DPP4i	SGLT2i	UC_2	942	1.1 (0.59-2.1)	7.58042e-01	9.020796e-01
DPP4i	SGLT2i	UC_3	632	0.4 (0.15-1)	5.24550e-02	4.550446e-01
DPP4i	SGLT2i	UC_4	496	1.1 (0.39-3.2)	8.41941e-01	9.020796e-01
DPP4i	SGLT2i	UC_5	2871	1.1 (0.63-1.8)	8.24915e-01	9.020796e-01
GLP1ra	SGLT2i	UC_1	675	0.8 (0.24-2.6)	7.19102e-01	9.020796e-01
GLP1ra	SGLT2i	UC_2	1145	1.2 (0.63-2.2)	6.02545e-01	9.020796e-01
GLP1ra	SGLT2i	UC_3	739	0.46 (0.14-1.5)	1.93435e-01	4.550446e-01
GLP1ra	SGLT2i	UC_4	607	2.5 (0.65-9.8)	1.79920e-01	4.550446e-01
GLP1ra	SGLT2i	UC_5	2991	0.9 (0.53-1.5)	7.06050e-01	9.020796e-01
Sulfonylurea	DPP4i	UC_1	1646	0.7 (0.42-1.2)	1.73523e-01	4.550446e-01
Sulfonylurea	DPP4i	UC_2	1868	0.66 (0.46-0.94)	2.27414e-02	4.550446e-01
Sulfonylurea	DPP4i	UC_3	1948	1.4 (0.98-2.1)	6.62407e-02	4.550446e-01
Sulfonylurea	DPP4i	UC_4	1188	1 (0.63-1.7)	9.01543e-01	9.302910e-01
Sulfonylurea	DPP4i	UC_5	5562	0.84 (0.64-1.1)	1.96591e-01	4.550446e-01
Sulfonylurea	GLP1ra	UC_1	715	2 (0.76-5.4)	1.56010e-01	4.550446e-01
Sulfonylurea	GLP1ra	UC_2	1072	0.94 (0.52-1.7)	8.40180e-01	9.020796e-01
Sulfonylurea	GLP1ra	UC_3	1008	0.66 (0.32-1.3)	2.48311e-01	4.551159e-01
Sulfonylurea	GLP1ra	UC_4	623	0.48 (0.16-1.4)	1.69945e-01	4.550446e-01
Sulfonylurea	GLP1ra	UC_5	2530	0.74 (0.44-1.2)	2.38795e-01	4.551159e-01
Sulfonylurea	SGLT2i	UC_1	1122	1.1 (0.52-2.5)	7.33441e-01	9.020796e-01
Sulfonylurea	SGLT2i	UC_2	986	0.85 (0.49-1.5)	5.72140e-01	9.020796e-01
Sulfonylurea	SGLT2i	UC_3	839	0.58 (0.24-1.4)	2.37084e-01	4.551159e-01
Sulfonylurea	SGLT2i	UC_4	684	0.54 (0.24-1.2)	1.22378e-01	4.550446e-01
Sulfonylurea	SGLT2i	UC_5	2843	0.93 (0.57-1.5)	7.78323e-01	9.020796e-01

4.16.2 eFigure: Individual effect size, meta analysis and sensitivity analysis

The forest plot illustrate the effect size of the comparison between DPP4i and GLP1ra at each UC along with the effect size obtained from the random effect meta-analysis across all the UC for outcome Heart Failure

© 2023 Vashisht R et al. *JAMA Network Open.*

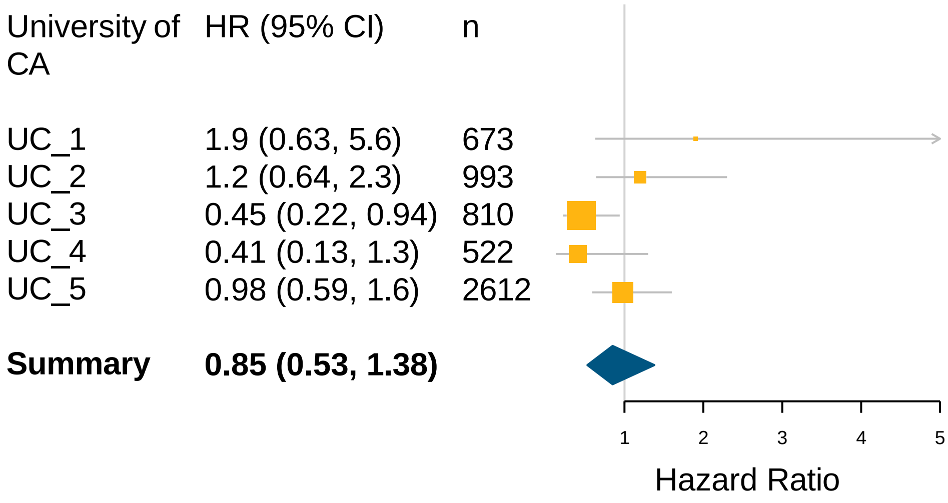

The table below shows the Leave-One-UC-Out diagnostics. The DFFITS value, Cook's distance, Covariance ratio, leave-one-out amount of heteroginity, indicator for influential estimates, comparator and treated groups are provided for each Leave-One-UC-Out analysis. The influential estimate from one UC with respect to pooled estimate are marked as Yes or No, with Yes indicating an influential UC and No otherwise.

eTable 108: Leave-One-UC-Out Sensitivity Analysis

DFFITs	Cook's Dist	Residual Heterogeneity	Influential	Comparator	Treated	UC
0.4808647	0.2197747	0.1198331	No	DPP4i	GLP1ra	UC_1
0.4011326	0.2029839	0.2073236	No	DPP4i	GLP1ra	UC_2
-0.9226716	0.5601337	0.0466622	Yes	DPP4i	GLP1ra	UC_3
-0.4205172	0.1720084	0.1289723	No	DPP4i	GLP1ra	UC_4
0.1782263	0.0551658	0.2948040	No	DPP4i	GLP1ra	UC_5

© 2023 Vashisht R et al. *JAMA Network Open.*

The forest plot illustrate the effect size of the comparison between DPP4i and SGLT2i at each UC along with the effect size obtained from the random effect meta-analysis across all the UC for outcome Heart Failure

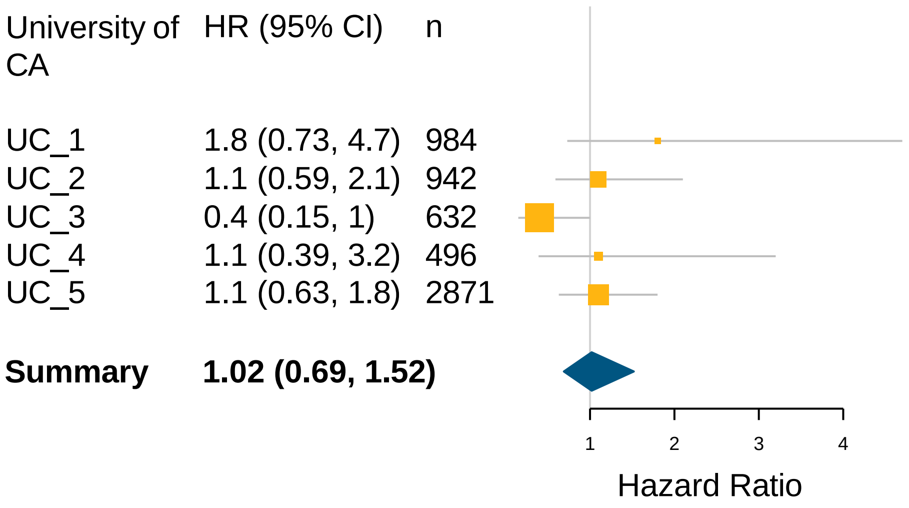

The table below shows the Leave-One-UC-Out diagnostics. The DFFITS value, Cook's distance, Covariance ratio, leave-one-out amount of heteroginity, indicator for influential estimates, comparator and treated groups are provided for each Leave-One-UC-Out analysis. The influential estimate from one UC with respect to pooled estimate are marked as Yes or No, with Yes indicating an influential UC and No otherwise.

eTable 109: Leave-One-UC-Out Sensitivity Analysis

DFFITs	Cook's Dist	Residual Heterogeneity	Influential	Comparator	Treated	UC
0.4607734	0.2018603	0.0365524	No	DPP4i	SGLT2i	UC_1
0.1472174	0.0335939	0.1354019	No	DPP4i	SGLT2i	UC_2
-0.7736969	0.4930742	0.0000000	Yes	DPP4i	SGLT2i	UC_3
0.0893241	0.0092486	0.1039943	No	DPP4i	SGLT2i	UC_4
0.1616479	0.0468532	0.1467734	No	DPP4i	SGLT2i	UC_5

The forest plot illustrate the effect size of the comparison between GLP1ra and SGLT2i at each UC along with the effect size obtained from the random effect meta-analysis across all the UC for outcome Heart Failure

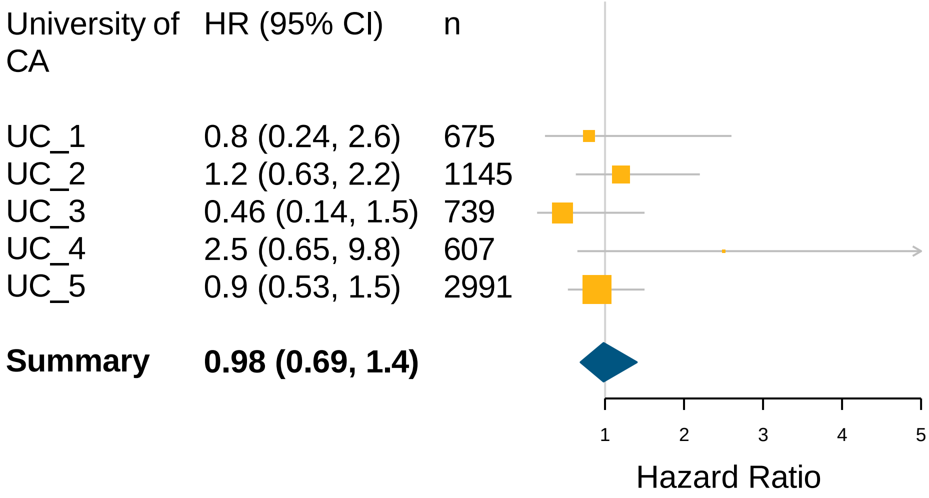

The table below shows the Leave-One-UC-Out diagnostics. The DFFITS value, Cook's distance, Covariance ratio, leave-one-out amount of heteroginity, indicator for influential estimates, comparator and treated groups are provided for each Leave-One-UC-Out analysis. The influential estimate from one UC with respect to pooled estimate are marked as Yes or No, with Yes indicating an influential UC and No otherwise.

eTable 110: Leave-One-UC-Out Sensitivity Analysis

DFFITs	Cook's Dist	Residual Heterogeneity	Influential	Comparator	Treated	UC
-0.1383440	0.0216407	0.0488428	No	GLP1ra	SGLT2i	UC_1
0.4177131	0.2396096	0.0386537	No	GLP1ra	SGLT2i	UC_2
-0.4038957	0.1629147	0.0000000	No	GLP1ra	SGLT2i	UC_3
0.3706072	0.1372100	0.0000000	No	GLP1ra	SGLT2i	UC_4
-0.2013703	0.0834088	0.0754499	No	GLP1ra	SGLT2i	UC_5

© 2023 Vashisht R et al. *JAMA Network Open.*

The forest plot illustrate the effect size of the comparison between Sulfonylurea and DPP4i at each UC along with the effect size obtained from the random effect meta-analysis across all the UC for outcome Heart Failure

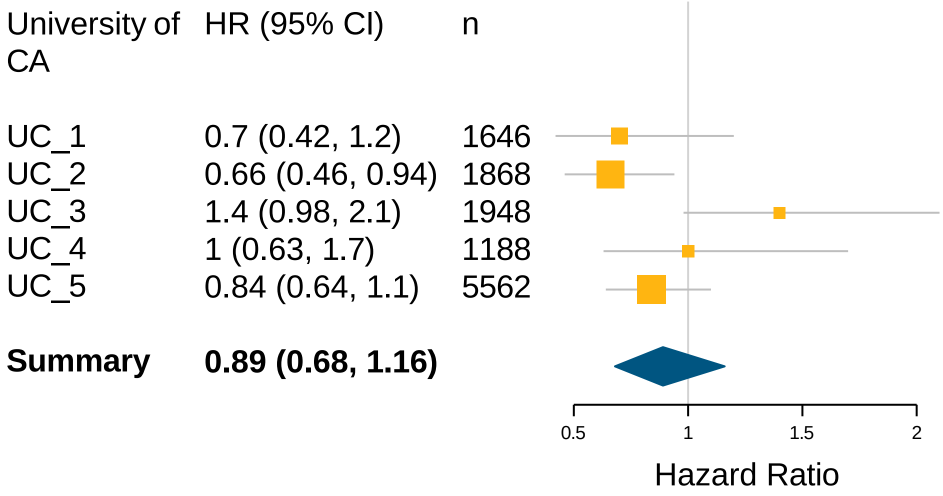

The table below shows the Leave-One-UC-Out diagnostics. The DFFITS value, Cook's distance, Covariance ratio, leave-one-out amount of heteroginity, indicator for influential estimates, comparator and treated groups are provided for each Leave-One-UC-Out analysis. The influential estimate from one UC with respect to pooled estimate are marked as Yes or No, with Yes indicating an influential UC and No otherwise.

eTable 111: Leave-One-UC-Out Sensitivity Analysis

DFFITs	Cook's Dist	Residual Heterogeneity	Influential	Comparator	Treated	UC
-0.3068822	0.1030796	0.0625258	No	Sulfonylurea	DPP4i	UC_1
-0.6420676	0.3728002	0.0428760	No	Sulfonylurea	DPP4i	UC_2
1.3207135	0.7431019	0.0000000	Yes	Sulfonylurea	DPP4i	UC_3
0.1627067	0.0308388	0.0699029	No	Sulfonylurea	DPP4i	UC_4
-0.0990139	0.0163924	0.0979877	No	Sulfonylurea	DPP4i	UC_5

© 2023 Vashisht R et al. *JAMA Network Open.*

The forest plot illustrate the effect size of the comparison between Sulfonylurea and GLP1ra at each UC along with the effect size obtained from the random effect meta-analysis across all the UC for outcome Heart Failure

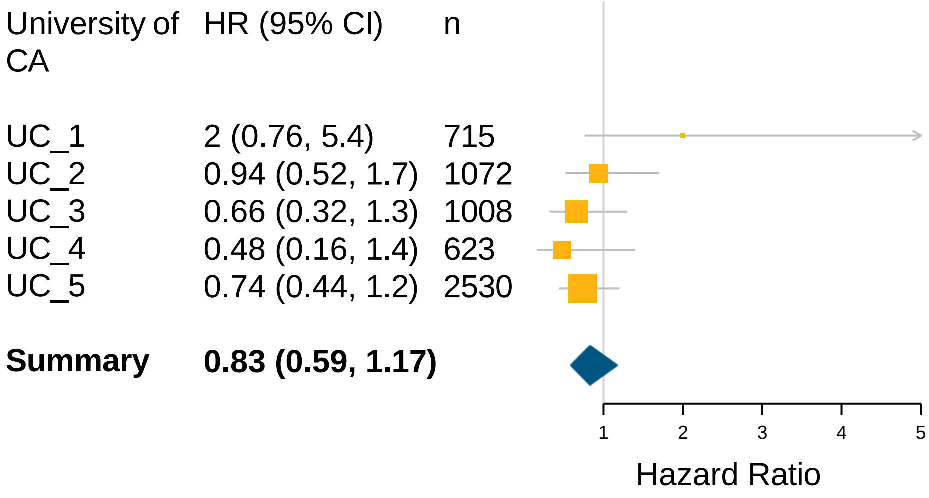

The table below shows the Leave-One-UC-Out diagnostics. The DFFITS value, Cook's distance, Covariance ratio, leave-one-out amount of heteroginity, indicator for influential estimates, comparator and treated groups are provided for each Leave-One-UC-Out analysis. The influential estimate from one UC with respect to pooled estimate are marked as Yes or No, with Yes indicating an influential UC and No otherwise.

eTable 112: Leave-One-UC-Out Sensitivity Analysis

DFFITs	Cook's Dist	Residual Heterogeneity	Influential	Comparator	Treated	UC
0.6109984	0.3361603	0.0000000	No	Sulfonylurea	GLP1ra	UC_1
0.1572362	0.0355104	0.0795712	No	Sulfonylurea	GLP1ra	UC_2
-0.3356768	0.1380563	0.0626906	No	Sulfonylurea	GLP1ra	UC_3
-0.3242036	0.1061293	0.0309031	No	Sulfonylurea	GLP1ra	UC_4
-0.2559170	0.1078178	0.0878687	No	Sulfonylurea	GLP1ra	UC_5

© 2023 Vashisht R et al. *JAMA Network Open.*

The forest plot illustrate the effect size of the comparison between Sulfonylurea and SGLT2i at each UC along with the effect size obtained from the random effect meta-analysis across all the UC for outcome Heart Failure

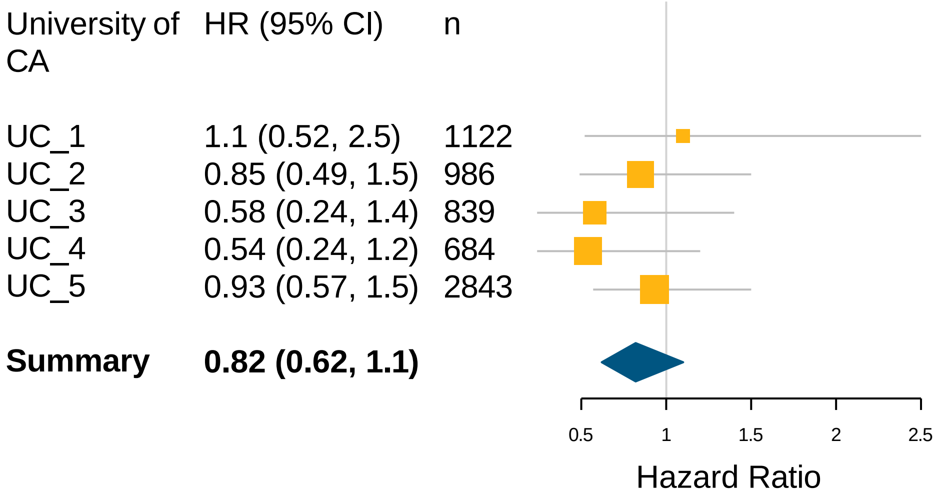

The table below shows the Leave-One-UC-Out diagnostics. The DFFITS value, Cook's distance, Covariance ratio, leave-one-out amount of heteroginity, indicator for influential estimates, comparator and treated groups are provided for each Leave-One-UC-Out analysis. The influential estimate from one UC with respect to pooled estimate are marked as Yes or No, with Yes indicating an influential UC and No otherwise.

eTable 113: Leave-One-UC-Out Sensitivity Analysis

DFFITs	Cook's Dist	Residual Heterogeneity	Influential	Comparator	Treated	UC
0.3096892	0.0959074	0	No	Sulfonylurea	SGLT2i	UC_1
0.0809846	0.0065585	0	No	Sulfonylurea	SGLT2i	UC_2
-0.2860418	0.0818199	0	No	Sulfonylurea	SGLT2i	UC_3
-0.4239690	0.1797497	0	No	Sulfonylurea	SGLT2i	UC_4
0.4635548	0.2148830	0	No	Sulfonylurea	SGLT2i	UC_5

© 2023 Vashisht R et al. *JAMA Network Open.*

4.17 Hypertension

4.17.1 eTable: Drug comparision table

Effect size of each drug comparison at each UC health site is tabulated.

eTable 114: Hazard ratios of drug class comparison at each UC

Comparator	Treated	UC	N	Hazard Ratio (95% CI)	P-value	Adjusted P-Value
DPP4i	GLP1ra	UC_1	277	1.6 (0.91-2.9)	1.00229e-01	3.678333e-01
DPP4i	GLP1ra	UC_2	355	0.84 (0.56-1.3)	4.18228e-01	6.603600e-01
DPP4i	GLP1ra	UC_3	287	0.68 (0.38-1.2)	1.87912e-01	5.124873e-01
DPP4i	GLP1ra	UC_4	234	0.91 (0.52-1.6)	7.36654e-01	8.499854e-01
DPP4i	GLP1ra	UC_5	1068	0.94 (0.71-1.2)	6.44070e-01	7.842612e-01
DPP4i	SGLT2i	UC_1	347	0.95 (0.54-1.7)	8.62105e-01	9.236839e-01
DPP4i	SGLT2i	UC_2	340	0.54 (0.33-0.88)	1.31591e-02	1.968980e-01
DPP4i	SGLT2i	UC_3	218	0.83 (0.47-1.5)	5.34148e-01	6.983778e-01
DPP4i	SGLT2i	UC_4	213	1.3 (0.73-2.3)	3.73950e-01	6.599118e-01
DPP4i	SGLT2i	UC_5	1189	0.87 (0.66-1.1)	2.99386e-01	5.987720e-01
GLP1ra	SGLT2i	UC_1	279	0.54 (0.29-1)	5.55197e-02	2.775985e-01
GLP1ra	SGLT2i	UC_2	378	0.65 (0.39-1.1)	9.83461e-02	3.678333e-01
GLP1ra	SGLT2i	UC_3	268	1.2 (0.65-2.3)	5.35423e-01	6.983778e-01
GLP1ra	SGLT2i	UC_4	258	1.4 (0.78-2.4)	2.76926e-01	5.987720e-01
GLP1ra	SGLT2i	UC_5	1159	1 (0.76-1.3)	9.88080e-01	9.880800e-01
Sulfonylurea	DPP4i	UC_1	584	0.76 (0.54-1.1)	1.10350e-01	3.678333e-01
Sulfonylurea	DPP4i	UC_2	666	1 (0.77-1.3)	9.02496e-01	9.336166e-01
Sulfonylurea	DPP4i	UC_3	610	1.3 (0.93-1.7)	1.28468e-01	3.854040e-01
Sulfonylurea	DPP4i	UC_4	526	0.87 (0.63-1.2)	4.04411e-01	6.603600e-01
Sulfonylurea	DPP4i	UC_5	2313	0.94 (0.8-1.1)	4.85476e-01	6.935371e-01
Sulfonylurea	GLP1ra	UC_1	315	1.1 (0.64-1.7)	8.45936e-01	9.236839e-01
Sulfonylurea	GLP1ra	UC_2	385	0.91 (0.61-1.4)	6.53551e-01	7.842612e-01
Sulfonylurea	GLP1ra	UC_3	383	0.74 (0.45-1.2)	2.53778e-01	5.987720e-01
Sulfonylurea	GLP1ra	UC_4	288	0.79 (0.47-1.3)	3.69746e-01	6.599118e-01
Sulfonylurea	GLP1ra	UC_5	1051	0.74 (0.57-0.97)	2.63603e-02	1.977022e-01
Sulfonylurea	SGLT2i	UC_1	454	0.56 (0.35-0.91)	1.96898e-02	1.968980e-01
Sulfonylurea	SGLT2i	UC_2	372	0.55 (0.35-0.85)	7.18782e-03	1.968980e-01
Sulfonylurea	SGLT2i	UC_3	312	1.2 (0.72-2)	4.60853e-01	6.912795e-01
Sulfonylurea	SGLT2i	UC_4	280	0.76 (0.47-1.2)	2.80635e-01	5.987720e-01
Sulfonylurea	SGLT2i	UC_5	1210	0.76 (0.59-0.98)	3.31406e-02	1.988436e-01

4.17.2 eFigure: Individual effect size, meta analysis and sensitivity analysis

The forest plot illustrate the effect size of the comparison between DPP4i and GLP1ra at each UC along with the effect size obtained from the random effect meta-analysis across all the UC for outcome Hypertension

© 2023 Vashisht R et al. *JAMA Network Open.*

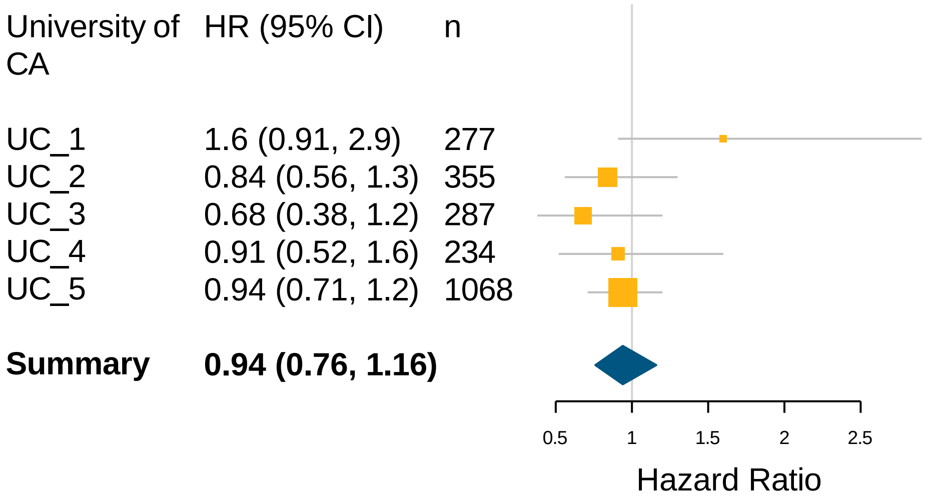

The table below shows the Leave-One-UC-Out diagnostics. The DFFITS value, Cook's distance, Covariance ratio, leave-one-out amount of heteroginity, indicator for influential estimates, comparator and treated groups are provided for each Leave-One-UC-Out analysis. The influential estimate from one UC with respect to pooled estimate are marked as Yes or No, with Yes indicating an influential UC and No otherwise.

eTable 115: Leave-One-UC-Out Sensitivity Analysis

DFFITs	Cook's Dist	Residual Heterogeneity	Influential	Comparator	Treated	UC
0.6050067	0.3299251	0.0000000	No	DPP4i	GLP1ra	UC_1
-0.2713722	0.0970588	0.0272870	No	DPP4i	GLP1ra	UC_2
-0.3950084	0.1512739	0.0066522	No	DPP4i	GLP1ra	UC_3
-0.0672475	0.0054191	0.0277696	No	DPP4i	GLP1ra	UC_4
-0.0279420	0.0017013	0.0419831	No	DPP4i	GLP1ra	UC_5

© 2023 Vashisht R et al. *JAMA Network Open.*

The forest plot illustrate the effect size of the comparison between DPP4i and SGLT2i at each UC along with the effect size obtained from the random effect meta-analysis across all the UC for outcome Hypertension

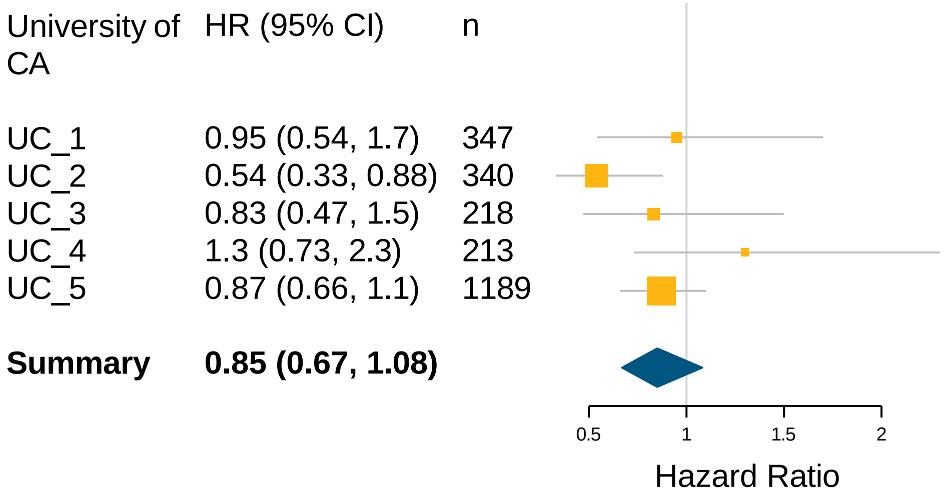

The table below shows the Leave-One-UC-Out diagnostics. The DFFITS value, Cook's distance, Covariance ratio, leave-one-out amount of heteroginity, indicator for influential estimates, comparator and treated groups are provided for each Leave-One-UC-Out analysis. The influential estimate from one UC with respect to pooled estimate are marked as Yes or No, with Yes indicating an influential UC and No otherwise.

eTable 116: Leave-One-UC-Out Sensitivity Analysis

DFFITs	Cook's Dist	Residual Heterogeneity	Influential	Comparator	Treated	UC
0.1402503	0.0233961	0.0422585	No	DPP4i	SGLT2i	UC_1
-0.7378317	0.4033278	0.0000000	No	DPP4i	SGLT2i	UC_2
-0.0474141	0.0027150	0.0446477	No	DPP4i	SGLT2i	UC_3
0.4925274	0.2022864	0.0040222	No	DPP4i	SGLT2i	UC_4
0.0288969	0.0017940	0.0665496	No	DPP4i	SGLT2i	UC_5

© 2023 Vashisht R et al. *JAMA Network Open.*

The forest plot illustrate the effect size of the comparison between GLP1ra and SGLT2i at each UC along with the effect size obtained from the random effect meta-analysis across all the UC for outcome Hypertension

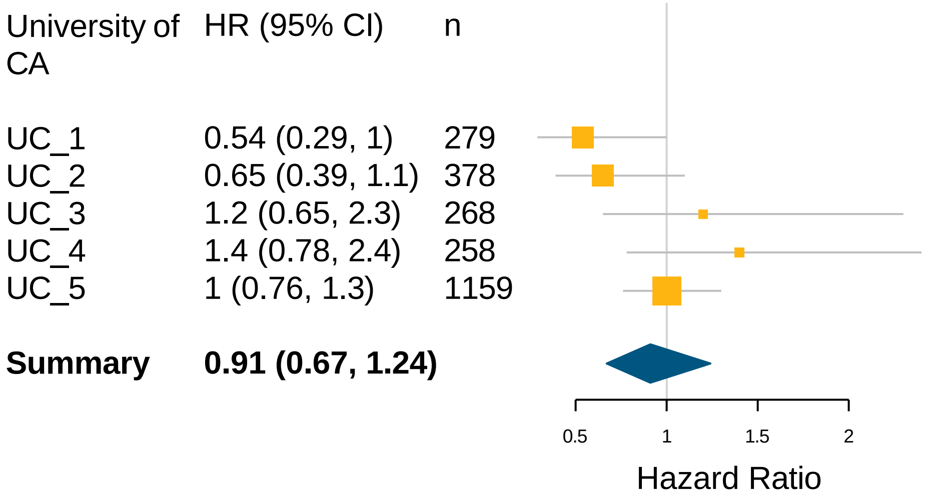

The table below shows the Leave-One-UC-Out diagnostics. The DFFITS value, Cook's distance, Covariance ratio, leave-one-out amount of heteroginity, indicator for influential estimates, comparator and treated groups are provided for each Leave-One-UC-Out analysis. The influential estimate from one UC with respect to pooled estimate are marked as Yes or No, with Yes indicating an influential UC and No otherwise.

eTable 117: Leave-One-UC-Out Sensitivity Analysis

DFFITs	Cook's Dist	Residual Heterogeneity	Influential	Comparator	Treated	UC
-0.6634033	0.3516902	0.0256168	No	GLP1ra	SGLT2i	UC_1
-0.5288053	0.2693204	0.0524387	No	GLP1ra	SGLT2i	UC_2
0.3318087	0.1235678	0.0768324	No	GLP1ra	SGLT2i	UC_3
0.5800258	0.3187393	0.0498001	No	GLP1ra	SGLT2i	UC_4
0.1978094	0.0735083	0.1237976	No	GLP1ra	SGLT2i	UC_5

© 2023 Vashisht R et al. *JAMA Network Open.*

The forest plot illustrate the effect size of the comparison between Sulfonylurea and DPP4i at each UC along with the effect size obtained from the random effect meta-analysis across all the UC for outcome Hypertension

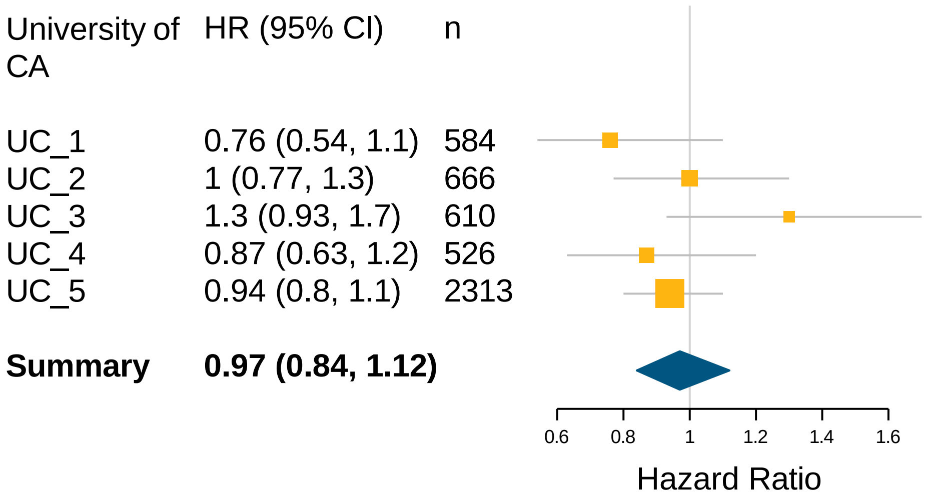

The table below shows the Leave-One-UC-Out diagnostics. The DFFITS value, Cook's distance, Covariance ratio, leave-one-out amount of heteroginity, indicator for influential estimates, comparator and treated groups are provided for each Leave-One-UC-Out analysis. The influential estimate from one UC with respect to pooled estimate are marked as Yes or No, with Yes indicating an influential UC and No otherwise.

eTable 118: Leave-One-UC-Out Sensitivity Analysis

DFFITs	Cook's Dist	Residual Heterogeneity	Influential	Comparator	Treated	UC
-0.4659180	0.2018816	0.0062957	No	Sulfonylurea	DPP4i	UC_1
0.1119019	0.0169259	0.0187807	No	Sulfonylurea	DPP4i	UC_2
0.7720733	0.4286524	0.0000000	No	Sulfonylurea	DPP4i	UC_3
-0.2501174	0.0720570	0.0147568	No	Sulfonylurea	DPP4i	UC_4
-0.0951302	0.0172213	0.0235613	No	Sulfonylurea	DPP4i	UC_5

© 2023 Vashisht R et al. *JAMA Network Open.*

The forest plot illustrate the effect size of the comparison between Sulfonylurea and GLP1ra at each UC along with the effect size obtained from the random effect meta-analysis across all the UC for outcome Hypertension

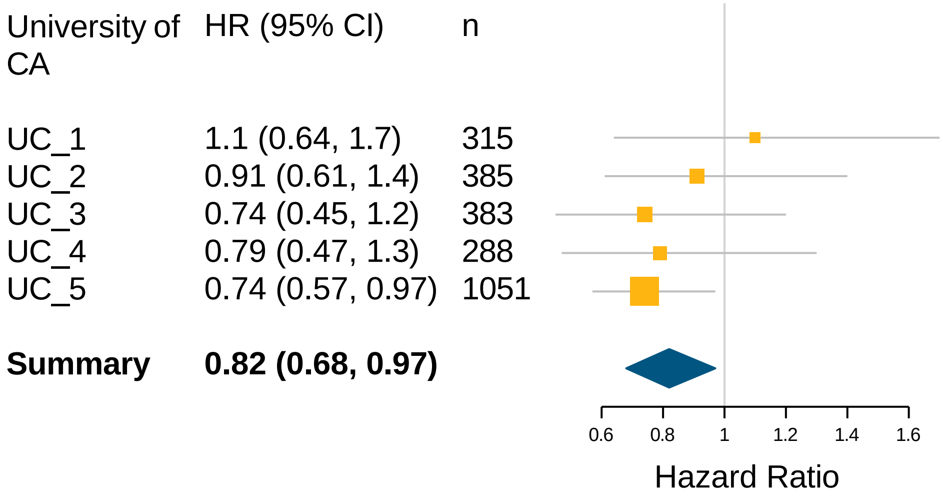

The table below shows the Leave-One-UC-Out diagnostics. The DFFITS value, Cook's distance, Covariance ratio, leave-one-out amount of heteroginity, indicator for influential estimates, comparator and treated groups are provided for each Leave-One-UC-Out analysis. The influential estimate from one UC with respect to pooled estimate are marked as Yes or No, with Yes indicating an influential UC and No otherwise.

eTable 119: Leave-One-UC-Out Sensitivity Analysis

DFFITs	Cook's Dist	Residual Heterogeneity	Influential	Comparator	Treated	UC
0.4990697	0.2490705	0	No	Sulfonylurea	GLP1ra	UC_1
0.2687900	0.0722480	0	No	Sulfonylurea	GLP1ra	UC_2
-0.1597329	0.0255146	0	No	Sulfonylurea	GLP1ra	UC_3
-0.0476691	0.0022723	0	No	Sulfonylurea	GLP1ra	UC_4
-0.8453091	0.7145475	0	Yes	Sulfonylurea	GLP1ra	UC_5

© 2023 Vashisht R et al. *JAMA Network Open.*

The forest plot illustrate the effect size of the comparison between Sulfonylurea and SGLT2i at each UC along with the effect size obtained from the random effect meta-analysis across all the UC for outcome Hypertension

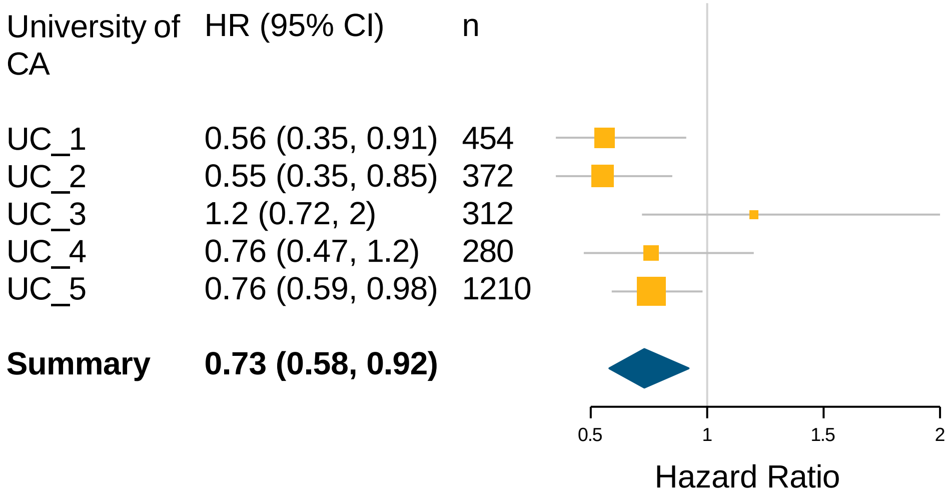

The table below shows the Leave-One-UC-Out diagnostics. The DFFITS value, Cook's distance, Covariance ratio, leave-one-out amount of heteroginity, indicator for influential estimates, comparator and treated groups are provided for each Leave-One-UC-Out analysis. The influential estimate from one UC with respect to pooled estimate are marked as Yes or No, with Yes indicating an influential UC and No otherwise.

eTable 120: Leave-One-UC-Out Sensitivity Analysis

DFFITs	Cook's Dist	Residual Heterogeneity	Influential	Comparator	Treated	UC
-0.4375955	0.1961187	0.0295967	No	Sulfonylurea	SGLT2i	UC_1
-0.5357796	0.2728580	0.0236006	No	Sulfonylurea	SGLT2i	UC_2
0.5897141	0.2475572	0.0000000	No	Sulfonylurea	SGLT2i	UC_3
0.0645760	0.0052425	0.0492727	No	Sulfonylurea	SGLT2i	UC_4
0.0889577	0.0147076	0.0654892	No	Sulfonylurea	SGLT2i	UC_5

© 2023 Vashisht R et al. *JAMA Network Open.*

4.18 Hypoglycemia

4.18.1 eTable: Drug comparision table

Effect size of each drug comparison at each UC health site is tabulated.

eTable 121: Hazard ratios of drug class comparison at each UC

Comparator	Treated	UC	N	Hazard Ratio (95% CI)	P-value	Adjusted P-Value
DPP4i	GLP1ra	UC_1	695	1.7 (0.41-7.2)	4.63141e-01	6.947115e-01
DPP4i	GLP1ra	UC_2	1083	2.6 (1.1-6.4)	3.04565e-02	1.541410e-01
DPP4i	GLP1ra	UC_3	857	2.1 (0.63-6.7)	2.28546e-01	4.570920e-01
DPP4i	GLP1ra	UC_4	553	1 (0.26-4.1)	9.77352e-01	9.967430e-01
DPP4i	GLP1ra	UC_5	2689	0.88 (0.4-1.9)	7.40188e-01	8.540631e-01
DPP4i	SGLT2i	UC_1	1057	0.41 (0.08-2.1)	2.85620e-01	5.040353e-01
DPP4i	SGLT2i	UC_2	1068	3.4 (0.93-13)	6.47204e-02	2.157347e-01
DPP4i	SGLT2i	UC_3	702	0.52 (0.13-2.1)	3.52674e-01	5.877900e-01
DPP4i	SGLT2i	UC_4	566	1 (0.14-7.1)	9.96743e-01	9.967430e-01
DPP4i	SGLT2i	UC_5	3000	1.3 (0.63-2.7)	4.60452e-01	6.947115e-01
GLP1ra	SGLT2i	UC_1	705	1.1 (0.21-5.3)	9.40317e-01	9.967430e-01
GLP1ra	SGLT2i	UC_2	1286	0.79 (0.35-1.8)	5.71021e-01	7.448100e-01
GLP1ra	SGLT2i	UC_3	806	0.41 (0.11-1.5)	1.82751e-01	4.217331e-01
GLP1ra	SGLT2i	UC_4	657	0.68 (0.11-4)	6.68233e-01	8.018796e-01
GLP1ra	SGLT2i	UC_5	3102	1.6 (0.72-3.3)	2.61920e-01	4.911000e-01
Sulfonylurea	DPP4i	UC_1	1727	0.66 (0.34-1.3)	2.22503e-01	4.570920e-01
Sulfonylurea	DPP4i	UC_2	2049	0.56 (0.34-0.95)	3.08282e-02	1.541410e-01
Sulfonylurea	DPP4i	UC_3	2105	0.64 (0.35-1.2)	1.36088e-01	3.711491e-01
Sulfonylurea	DPP4i	UC_4	1269	0.4 (0.15-1)	5.69398e-02	2.135242e-01
Sulfonylurea	DPP4i	UC_5	5778	0.34 (0.23-0.5)	8.19797e-08	2.459391e-06
Sulfonylurea	GLP1ra	UC_1	736	0.75 (0.24-2.4)	6.17247e-01	7.715587e-01
Sulfonylurea	GLP1ra	UC_2	1162	1 (0.55-1.9)	9.38098e-01	9.967430e-01
Sulfonylurea	GLP1ra	UC_3	1069	0.77 (0.34-1.8)	5.33306e-01	7.448100e-01
Sulfonylurea	GLP1ra	UC_4	656	0.69 (0.19-2.4)	5.65861e-01	7.448100e-01
Sulfonylurea	GLP1ra	UC_5	2603	0.3 (0.15-0.61)	9.37731e-04	1.406596e-02
Sulfonylurea	SGLT2i	UC_1	1198	0.28 (0.09-0.84)	2.27278e-02	1.541410e-01
Sulfonylurea	SGLT2i	UC_2	1123	0.56 (0.25-1.2)	1.54596e-01	3.864900e-01
Sulfonylurea	SGLT2i	UC_3	961	0.29 (0.08-1)	5.42037e-02	2.135242e-01
Sulfonylurea	SGLT2i	UC_4	767	0.1 (0.01-0.79)	2.89427e-02	1.541410e-01
Sulfonylurea	SGLT2i	UC_5	2975	0.59 (0.33-1.1)	7.39171e-02	2.217513e-01

4.18.2 eFigure: Individual effect size, meta analysis and sensitivity analysis

The forest plot illustrate the effect size of the comparison between DPP4i and GLP1ra at each UC along with the effect size obtained from the random effect meta-analysis across all the UC for outcome Hypoglycemia

© 2023 Vashisht R et al. *JAMA Network Open.*

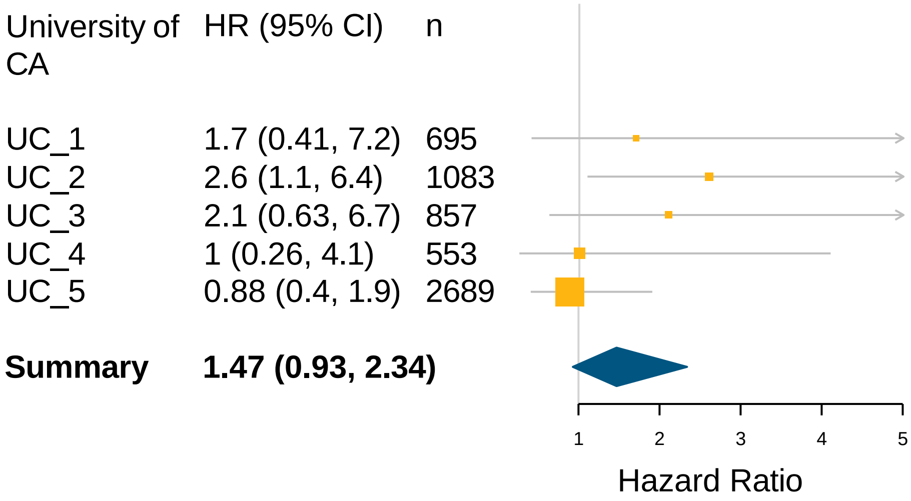

The table below shows the Leave-One-UC-Out diagnostics. The DFFITS value, Cook's distance, Covariance ratio, leave-one-out amount of heteroginity, indicator for influential estimates, comparator and treated groups are provided for each Leave-One-UC-Out analysis. The influential estimate from one UC with respect to pooled estimate are marked as Yes or No, with Yes indicating an influential UC and No otherwise.

eTable 122: Leave-One-UC-Out Sensitivity Analysis

DFFITs	Cook's Dist	Residual Heterogeneity	Influential	Comparator	Treated	UC
0.0103416	0.0001233	0.0817520	No	DPP4i	GLP1ra	UC_1
0.9176430	0.8420688	0.0000000	Yes	DPP4i	GLP1ra	UC_2
0.2217599	0.0564465	0.0537652	No	DPP4i	GLP1ra	UC_3
-0.2774154	0.0856538	0.0559287	No	DPP4i	GLP1ra	UC_4
-1.1924800	1.4220085	0.0000000	Yes	DPP4i	GLP1ra	UC_5

© 2023 Vashisht R et al. *JAMA Network Open.*

The forest plot illustrate the effect size of the comparison between DPP4i and SGLT2i at each UC along with the effect size obtained from the random effect meta-analysis across all the UC for outcome Hypoglycemia

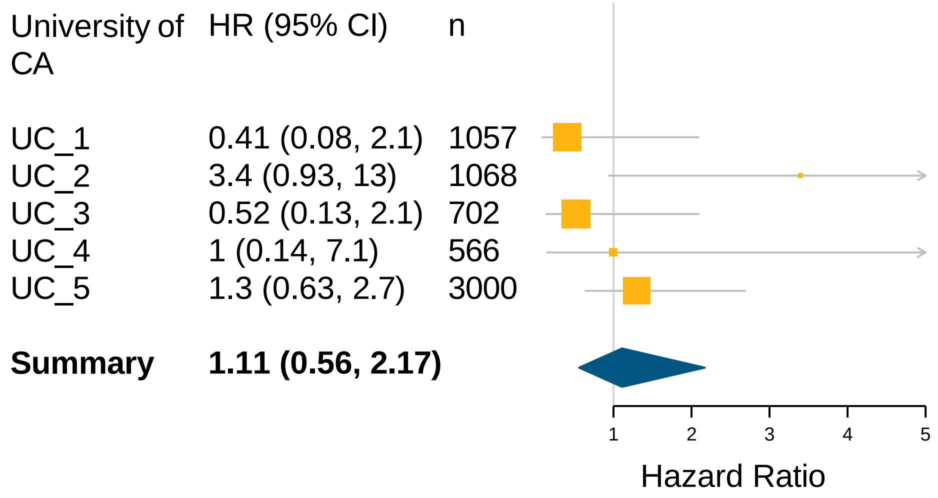

The table below shows the Leave-One-UC-Out diagnostics. The DFFITS value, Cook's distance, Covariance ratio, leave-one-out amount of heteroginity, indicator for influential estimates, comparator and treated groups are provided for each Leave-One-UC-Out analysis. The influential estimate from one UC with respect to pooled estimate are marked as Yes or No, with Yes indicating an influential UC and No otherwise.

eTable 123: Leave-One-UC-Out Sensitivity Analysis

DFFITs	Cook's Dist	Residual Heterogeneity	Influential	Comparator	Treated	UC
-0.4825074	0.2173526	0.1045779	No	DPP4i	SGLT2i	UC_1
0.5343273	0.2103247	0.0000000	No	DPP4i	SGLT2i	UC_2
-0.4820979	0.2277052	0.1483308	No	DPP4i	SGLT2i	UC_3
0.0351289	0.0013919	0.3108199	No	DPP4i	SGLT2i	UC_4
0.2646243	0.1430250	0.4742066	No	DPP4i	SGLT2i	UC_5

The forest plot illustrate the effect size of the comparison between GLP1ra and SGLT2i at each UC along with the effect size obtained from the random effect meta-analysis across all the UC for outcome Hypoglycemia

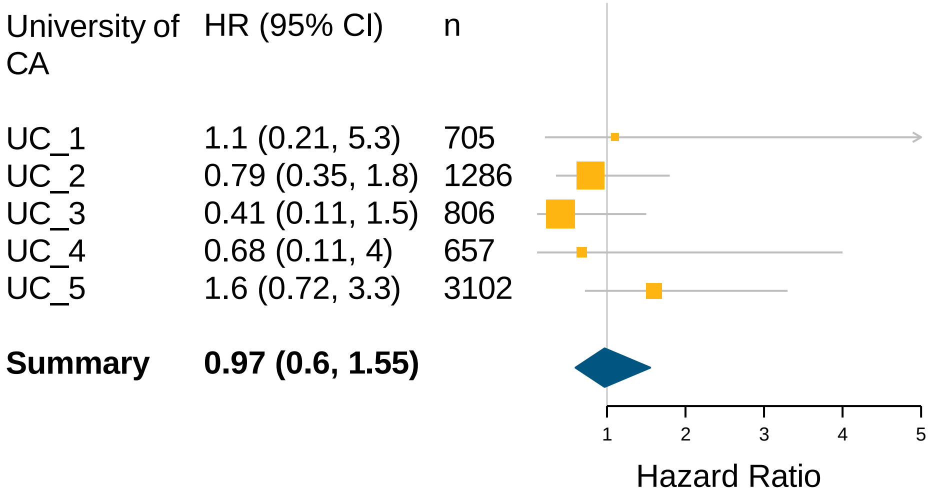

The table below shows the Leave-One-UC-Out diagnostics. The DFFITS value, Cook's distance, Covariance ratio, leave-one-out amount of heteroginity, indicator for influential estimates, comparator and treated groups are provided for each Leave-One-UC-Out analysis. The influential estimate from one UC with respect to pooled estimate are marked as Yes or No, with Yes indicating an influential UC and No otherwise.

eTable 124: Leave-One-UC-Out Sensitivity Analysis

DFFITs	Cook's Dist	Residual Heterogeneity	Influential	Comparator	Treated	UC
0.2056229	0.0465320	0.0681990	No	GLP1ra	SGLT2i	UC_1
-0.1914000	0.0483517	0.0558246	No	GLP1ra	SGLT2i	UC_2
-0.5350298	0.2862569	0.0000000	No	GLP1ra	SGLT2i	UC_3
-0.0044232	0.0000208	0.0538630	No	GLP1ra	SGLT2i	UC_4
1.3005096	1.6913252	0.0000000	Yes	GLP1ra	SGLT2i	UC_5

The forest plot illustrate the effect size of the comparison between Sulfonylurea and DPP4i at each UC along with the effect size obtained from the random effect meta-analysis across all the UC for outcome Hypoglycemia

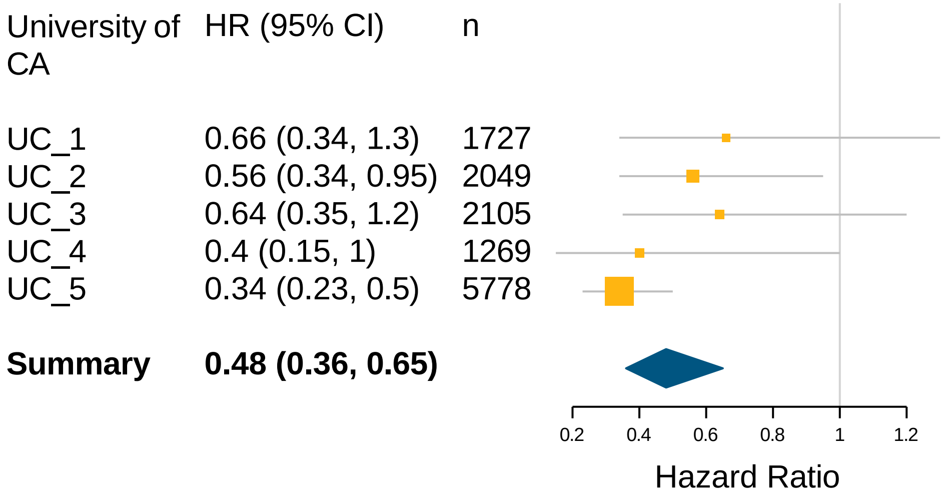

The table below shows the Leave-One-UC-Out diagnostics. The DFFITS value, Cook's distance, Covariance ratio, leave-one-out amount of heteroginity, indicator for influential estimates, comparator and treated groups are provided for each Leave-One-UC-Out analysis. The influential estimate from one UC with respect to pooled estimate are marked as Yes or No, with Yes indicating an influential UC and No otherwise.

eTable 125: Leave-One-UC-Out Sensitivity Analysis

DFFITs	Cook's Dist	Residual Heterogeneity	Influential	Comparator	Treated	UC
0.3791652	0.1457593	0.0274331	No	Sulfonylurea	DPP4i	UC_1
0.1647508	0.0342758	0.0502040	No	Sulfonylurea	DPP4i	UC_2
0.3973912	0.1614589	0.0282421	No	Sulfonylurea	DPP4i	UC_3
-0.2316799	0.0588181	0.0503369	No	Sulfonylurea	DPP4i	UC_4
-1.6009510	1.5545706	0.0000000	Yes	Sulfonylurea	DPP4i	UC_5

The forest plot illustrate the effect size of the comparison between Sulfonylurea and GLP1ra at each UC along with the effect size obtained from the random effect meta-analysis across all the UC for outcome Hypoglycemia

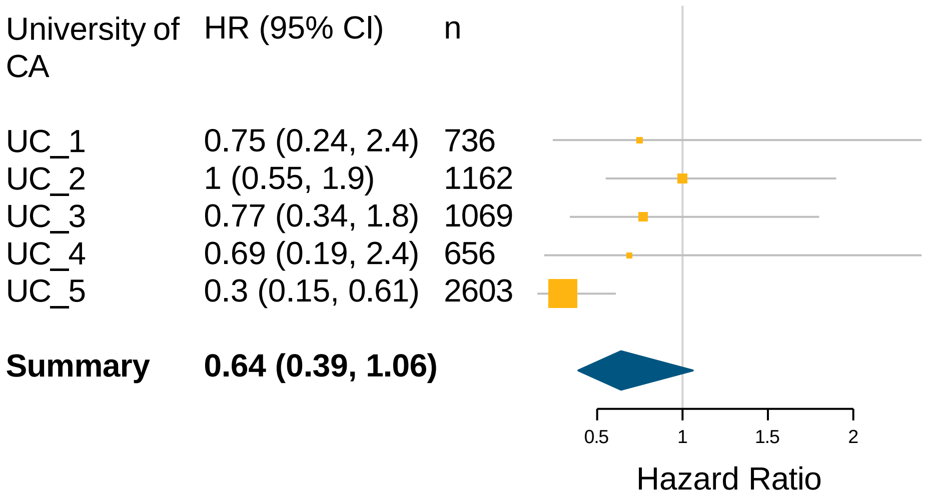

The table below shows the Leave-One-UC-Out diagnostics. The DFFITS value, Cook's distance, Covariance ratio, leave-one-out amount of heteroginity, indicator for influential estimates, comparator and treated groups are provided for each Leave-One-UC-Out analysis. The influential estimate from one UC with respect to pooled estimate are marked as Yes or No, with Yes indicating an influential UC and No otherwise.

eTable 126: Leave-One-UC-Out Sensitivity Analysis

DFFITs	Cook's Dist	Residual Heterogeneity	Influential	Comparator	Treated	UC
0.0903931	0.0094706	0.2034359	No	Sulfonylurea	GLP1ra	UC_1
0.9254525	0.6033785	0.0607369	Yes	Sulfonylurea	GLP1ra	UC_2
0.1554621	0.0319512	0.2276447	No	Sulfonylurea	GLP1ra	UC_3
0.0321839	0.0011732	0.2006855	No	Sulfonylurea	GLP1ra	UC_4
-1.6111985	1.2975084	0.0000000	Yes	Sulfonylurea	GLP1ra	UC_5

© 2023 Vashisht R et al. *JAMA Network Open.*

The forest plot illustrate the effect size of the comparison between Sulfonylurea and SGLT2i at each UC along with the effect size obtained from the random effect meta-analysis across all the UC for outcome Hypoglycemia

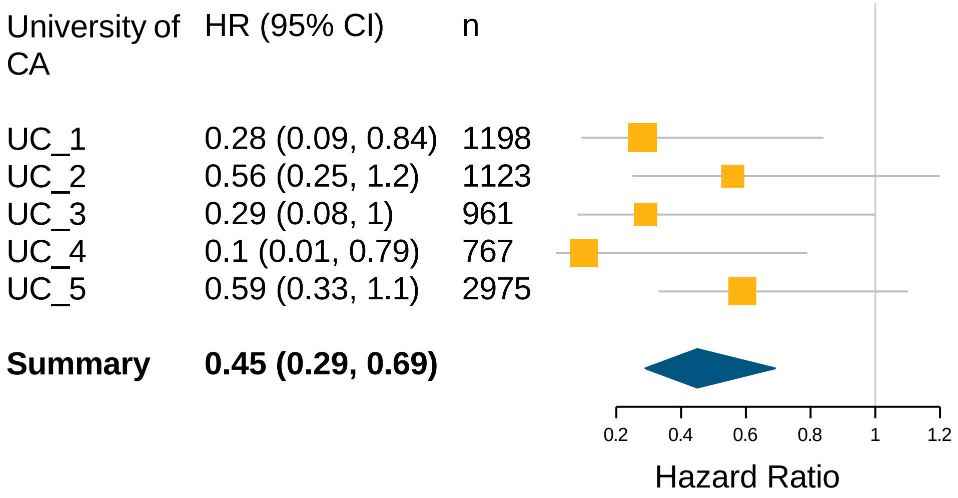

The table below shows the Leave-One-UC-Out diagnostics. The DFFITS value, Cook's distance, Covariance ratio, leave-one-out amount of heteroginity, indicator for influential estimates, comparator and treated groups are provided for each Leave-One-UC-Out analysis. The influential estimate from one UC with respect to pooled estimate are marked as Yes or No, with Yes indicating an influential UC and No otherwise.

eTable 127: Leave-One-UC-Out Sensitivity Analysis

DFFITs	Cook's Dist	Residual Heterogeneity	Influential	Comparator	Treated	UC
-0.2832515	0.0842143	0.0356847	No	Sulfonylurea	SGLT2i	UC_1
0.6459128	0.6049652	0.0995343	Yes	Sulfonylurea	SGLT2i	UC_2
-0.0992318	0.0107182	0.0573514	No	Sulfonylurea	SGLT2i	UC_3
-0.3789818	0.1411909	0.0000000	No	Sulfonylurea	SGLT2i	UC_4
0.9602280	0.7859405	0.0022219	Yes	Sulfonylurea	SGLT2i	UC_5

© 2023 Vashisht R et al. *JAMA Network Open.*

4.19 Liver Cirrhosis

4.19.1 eTable: Drug comparision table

Effect size of each drug comparison at each UC health site is tabulated.

eTable 128: Hazard ratios of drug class comparison at each UC

Comparator	Treated	UC	N	Hazard Ratio (95% CI)	P-value	Adjusted P-Value
DPP4i	GLP1ra	UC_1	704	0.67 (0.11-4)	6.63408e-01	9.426344e-01
DPP4i	GLP1ra	UC_2	1045	0.88 (0.3-2.6)	8.26025e-01	9.426344e-01
DPP4i	GLP1ra	UC_3	862	1.9 (0.34-10)	4.70193e-01	9.426344e-01
DPP4i	GLP1ra	UC_4	535	0.35 (0.07-1.7)	1.94451e-01	9.140900e-01
DPP4i	GLP1ra	UC_5	2682	1 (0.32-3.1)	9.99543e-01	9.995430e-01
DPP4i	SGLT2i	UC_1	1054	0.7 (0.12-4.2)	7.01233e-01	9.426344e-01
DPP4i	SGLT2i	UC_2	1037	1.1 (0.31-3.7)	9.22848e-01	9.546703e-01
DPP4i	SGLT2i	UC_3	708	0.68 (0.11-4.1)	6.69361e-01	9.426344e-01
DPP4i	SGLT2i	UC_4	547	0.2 (0.04-0.92)	3.93334e-02	9.140900e-01
DPP4i	SGLT2i	UC_5	2995	1.9 (0.71-5.2)	1.94504e-01	9.140900e-01
GLP1ra	SGLT2i	UC_1	707	0.47 (0.04-5.2)	5.34841e-01	9.426344e-01
GLP1ra	SGLT2i	UC_2	1260	1.1 (0.31-3.7)	9.17294e-01	9.546703e-01
GLP1ra	SGLT2i	UC_3	819	0.49 (0.04-5.5)	5.64968e-01	9.426344e-01
GLP1ra	SGLT2i	UC_4	637	2 (0.36-11)	4.29757e-01	9.426344e-01
GLP1ra	SGLT2i	UC_5	3106	1.2 (0.47-2.9)	7.35612e-01	9.426344e-01
Sulfonylurea	DPP4i	UC_1	1735	0.59 (0.23-1.5)	2.74227e-01	9.140900e-01
Sulfonylurea	DPP4i	UC_2	1981	0.73 (0.33-1.6)	4.25802e-01	9.426344e-01
Sulfonylurea	DPP4i	UC_3	2106	1.3 (0.49-3.3)	6.13396e-01	9.426344e-01
Sulfonylurea	DPP4i	UC_4	1213	1.1 (0.45-2.7)	8.23592e-01	9.426344e-01
Sulfonylurea	DPP4i	UC_5	5716	0.92 (0.48-1.7)	7.92743e-01	9.426344e-01
Sulfonylurea	GLP1ra	UC_1	742	0.39 (0.08-2)	2.66748e-01	9.140900e-01
Sulfonylurea	GLP1ra	UC_2	1126	0.5 (0.19-1.3)	1.62960e-01	9.140900e-01
Sulfonylurea	GLP1ra	UC_3	1074	4.8 (0.57-42)	1.49572e-01	9.140900e-01
Sulfonylurea	GLP1ra	UC_4	627	0.5 (0.09-2.7)	4.24281e-01	9.426344e-01
Sulfonylurea	GLP1ra	UC_5	2597	0.72 (0.27-1.9)	5.05648e-01	9.426344e-01
Sulfonylurea	SGLT2i	UC_1	1209	0.29 (0.06-1.4)	1.26254e-01	9.140900e-01
Sulfonylurea	SGLT2i	UC_2	1096	0.54 (0.18-1.6)	2.54819e-01	9.140900e-01
Sulfonylurea	SGLT2i	UC_3	976	2.2 (0.2-24)	5.28868e-01	9.426344e-01
Sulfonylurea	SGLT2i	UC_4	731	0.68 (0.11-4.1)	6.78110e-01	9.426344e-01
Sulfonylurea	SGLT2i	UC_5	2963	0.92 (0.41-2.1)	8.48371e-01	9.426344e-01

4.19.2 eFigure: Individual effect size, meta analysis and sensitivity analysis

The forest plot illustrate the effect size of the comparison between DPP4i and GLP1ra at each UC along with the effect size obtained from the random effect meta-analysis across all the UC for outcome Liver Cirrhosis

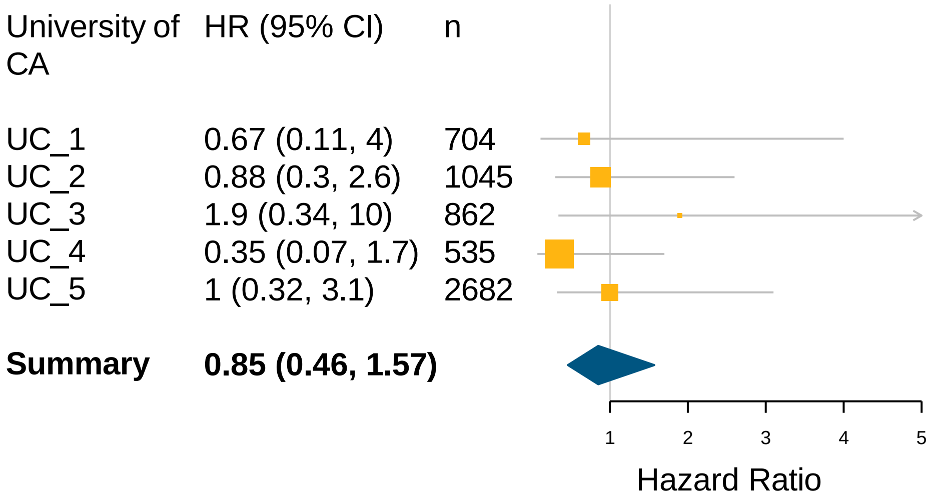

The table below shows the Leave-One-UC-Out diagnostics. The DFFITS value, Cook's distance, Covariance ratio, leave-one-out amount of heteroginity, indicator for influential estimates, comparator and treated groups are provided for each Leave-One-UC-Out analysis. The influential estimate from one UC with respect to pooled estimate are marked as Yes or No, with Yes indicating an influential UC and No otherwise.

eTable 129: Leave-One-UC-Out Sensitivity Analysis

DFFITs	Cook's Dist	Residual	Heterogeneity	Influential	Comparator	Treated	UC
-0.1019256	0.0103888		0	No	DPP4i	GLP1ra	UC_1
0.0440198	0.0019377		0	No	DPP4i	GLP1ra	UC_2
0.3842768	0.1476687		0	No	DPP4i	GLP1ra	UC_3
-0.4916492	0.2417189		0	No	DPP4i	GLP1ra	UC_4
0.2048275	0.0419543		0	No	DPP4i	GLP1ra	UC_5

© 2023 Vashisht R et al. *JAMA Network Open.*

The forest plot illustrate the effect size of the comparison between DPP4i and SGLT2i at each UC along with the effect size obtained from the random effect meta-analysis across all the UC for outcome Liver Cirrhosis

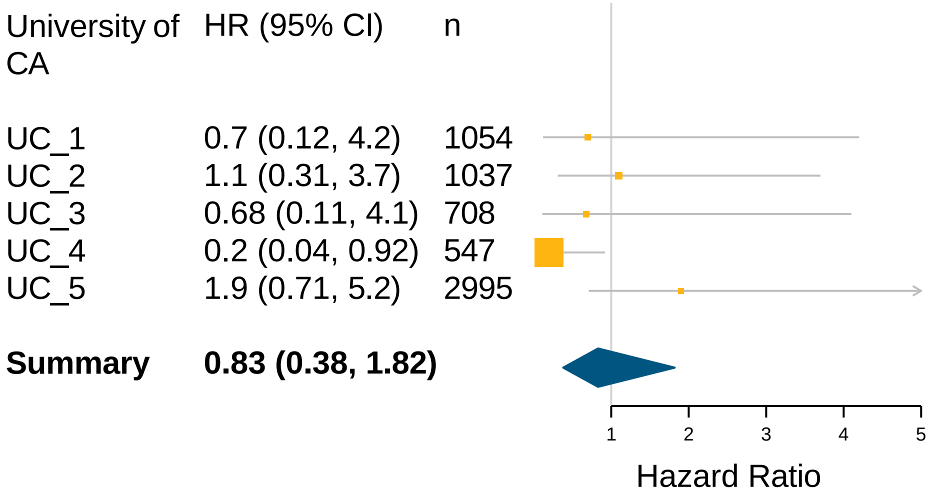

The table below shows the Leave-One-UC-Out diagnostics. The DFFITS value, Cook's distance, Covariance ratio, leave-one-out amount of heteroginity, indicator for influential estimates, comparator and treated groups are provided for each Leave-One-UC-Out analysis. The influential estimate from one UC with respect to pooled estimate are marked as Yes or No, with Yes indicating an influential UC and No otherwise.

eTable 130: Leave-One-UC-Out Sensitivity Analysis

DFFITs	Cook's Dist	Residual Heterogeneity	Influential	Comparator	Treated	UC
0.0158820	0.0002978	0.4777361	No	DPP4i	SGLT2i	UC_1
0.2911783	0.1223761	0.5797751	No	DPP4i	SGLT2i	UC_2
0.0035486	0.0000147	0.4717258	No	DPP4i	SGLT2i	UC_3
-1.1947982	0.9754581	0.0000000	Yes	DPP4i	SGLT2i	UC_4
1.1647011	0.6520371	0.0000000	Yes	DPP4i	SGLT2i	UC_5

© 2023 Vashisht R et al. *JAMA Network Open.*

The forest plot illustrate the effect size of the comparison between GLP1ra and SGLT2i at each UC along with the effect size obtained from the random effect meta-analysis across all the UC for outcome Liver Cirrhosis

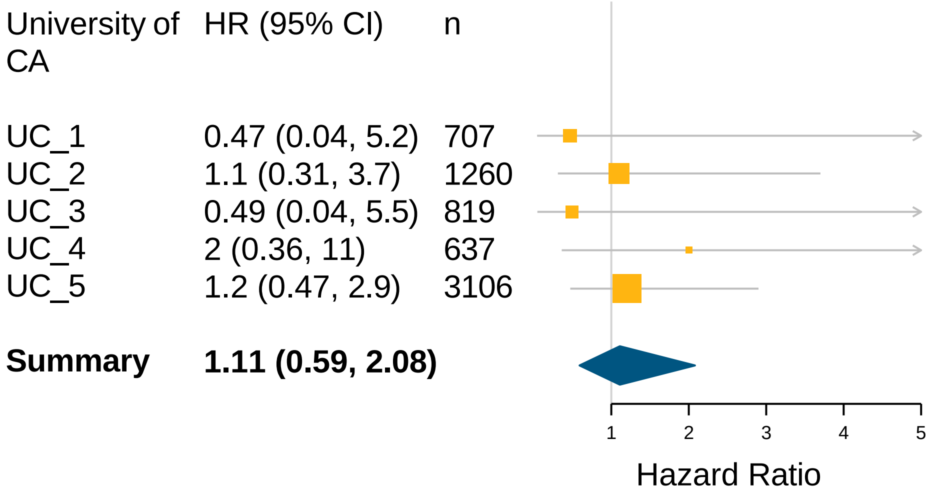

The table below shows the Leave-One-UC-Out diagnostics. The DFFITS value, Cook's distance, Covariance ratio, leave-one-out amount of heteroginity, indicator for influential estimates, comparator and treated groups are provided for each Leave-One-UC-Out analysis. The influential estimate from one UC with respect to pooled estimate are marked as Yes or No, with Yes indicating an influential UC and No otherwise.

eTable 131: Leave-One-UC-Out Sensitivity Analysis

DFFITs	Cook's Dist	Residual Heterogeneity	Influential	Comparator	Treated	UC
-0.1950394	0.0380404	0	No	GLP1ra	SGLT2i	UC_1
-0.0097301	0.0000947	0	No	GLP1ra	SGLT2i	UC_2
-0.1866526	0.0348392	0	No	GLP1ra	SGLT2i	UC_3
0.2857867	0.0816741	0	No	GLP1ra	SGLT2i	UC_4
0.2200525	0.0484231	0	No	GLP1ra	SGLT2i	UC_5

© 2023 Vashisht R et al. *JAMA Network Open.*

The forest plot illustrate the effect size of the comparison between Sulfonylurea and DPP4i at each UC along with the effect size obtained from the random effect meta-analysis across all the UC for outcome Liver Cirrhosis

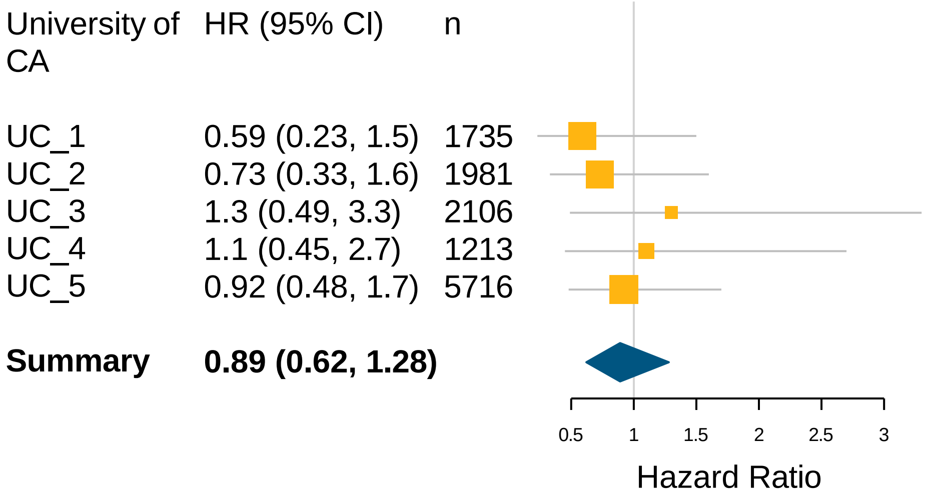

The table below shows the Leave-One-UC-Out diagnostics. The DFFITS value, Cook's distance, Covariance ratio, leave-one-out amount of heteroginity, indicator for influential estimates, comparator and treated groups are provided for each Leave-One-UC-Out analysis. The influential estimate from one UC with respect to pooled estimate are marked as Yes or No, with Yes indicating an influential UC and No otherwise.

eTable 132: Leave-One-UC-Out Sensitivity Analysis

DFFITs	Cook's Dist	Residual Heterogeneity	Influential	Comparator	Treated	UC
-0.3885996	0.1510096	0	No	Sulfonylurea	DPP4i	UC_1
-0.2827445	0.0799444	0	No	Sulfonylurea	DPP4i	UC_2
0.3493197	0.1220242	0	No	Sulfonylurea	DPP4i	UC_3
0.2277766	0.0518822	0	No	Sulfonylurea	DPP4i	UC_4
0.0957735	0.0091726	0	No	Sulfonylurea	DPP4i	UC_5

© 2023 Vashisht R et al. *JAMA Network Open.*

The forest plot illustrate the effect size of the comparison between Sulfonylurea and GLP1ra at each UC along with the effect size obtained from the random effect meta-analysis across all the UC for outcome Liver Cirrhosis

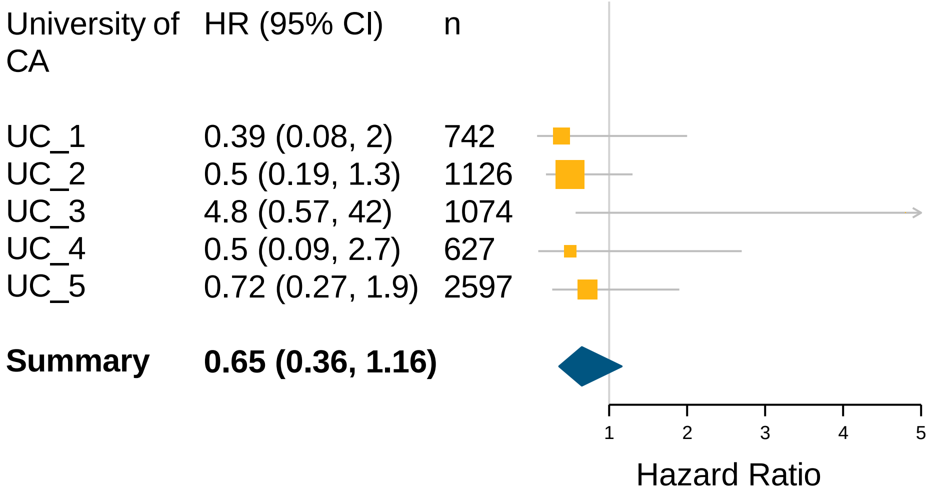

The table below shows the Leave-One-UC-Out diagnostics. The DFFITS value, Cook's distance, Covariance ratio, leave-one-out amount of heteroginity, indicator for influential estimates, comparator and treated groups are provided for each Leave-One-UC-Out analysis. The influential estimate from one UC with respect to pooled estimate are marked as Yes or No, with Yes indicating an influential UC and No otherwise.

eTable 133: Leave-One-UC-Out Sensitivity Analysis

DFFITs	Cook's Dist	Residual Heterogeneity	Influential	Comparator	Treated	UC
-0.3255159	0.1192016	0.1023074	No	Sulfonylurea	GLP1ra	UC_1
-0.4260179	0.2739069	0.1431346	No	Sulfonylurea	GLP1ra	UC_2
0.5248597	0.2723908	0.0000000	No	Sulfonylurea	GLP1ra	UC_3
-0.2176420	0.0555958	0.1459239	No	Sulfonylurea	GLP1ra	UC_4
-0.0115563	0.0002341	0.2104176	No	Sulfonylurea	GLP1ra	UC_5

© 2023 Vashisht R et al. *JAMA Network Open.*

The forest plot illustrate the effect size of the comparison between Sulfonylurea and SGLT2i at each UC along with the effect size obtained from the random effect meta-analysis across all the UC for outcome Liver Cirrhosis

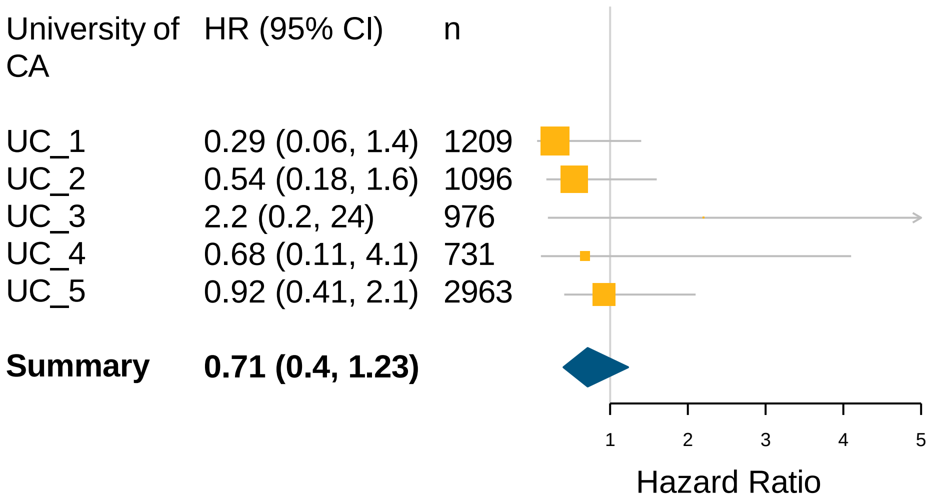

The table below shows the Leave-One-UC-Out diagnostics. The DFFITS value, Cook's distance, Covariance ratio, leave-one-out amount of heteroginity, indicator for influential estimates, comparator and treated groups are provided for each Leave-One-UC-Out analysis. The influential estimate from one UC with respect to pooled estimate are marked as Yes or No, with Yes indicating an influential UC and No otherwise.

eTable 134: Leave-One-UC-Out Sensitivity Analysis

DFFITs	Cook's Dist	Residual Heterogeneity	Influential	Comparator	Treated	UC
-0.4523537	0.2046239	0	No	Sulfonylurea	SGLT2i	UC_1
-0.3299747	0.1088833	0	No	Sulfonylurea	SGLT2i	UC_2
0.2291036	0.0524885	0	No	Sulfonylurea	SGLT2i	UC_3
-0.0134409	0.0001807	0	No	Sulfonylurea	SGLT2i	UC_4
0.8126874	0.6604608	0	Yes	Sulfonylurea	SGLT2i	UC_5

4.20 Myocardial Infarction

4.20.1 eTable: Drug comparision table

Effect size of each drug comparison at each UC health site is tabulated.

eTable 135: Hazard ratios of drug class comparison at each UC

Comparator	Treated	UC	N	Hazard Ratio (95% CI)	P-value	Adjusted P-Value
DPP4i	GLP1ra	UC_1	681	1.4 (0.32-6.4)	6.41592e-01	9.233588e-01
DPP4i	GLP1ra	UC_2	1055	1.4 (0.6-3.1)	4.52111e-01	9.233588e-01
DPP4i	GLP1ra	UC_3	843	1.6 (0.48-5.6)	4.28620e-01	9.233588e-01
DPP4i	GLP1ra	UC_4	534	1 (0.21-5.2)	9.57250e-01	9.754560e-01
DPP4i	GLP1ra	UC_5	2640	1 (0.47-2.2)	9.54301e-01	9.754560e-01
DPP4i	SGLT2i	UC_1	1010	1.1 (0.36-3.4)	8.55709e-01	9.754560e-01
DPP4i	SGLT2i	UC_2	1022	1.1 (0.46-2.4)	8.97767e-01	9.754560e-01
DPP4i	SGLT2i	UC_3	660	1.3 (0.46-3.5)	6.41608e-01	9.233588e-01
DPP4i	SGLT2i	UC_4	533	1 (0.26-4.1)	9.75456e-01	9.754560e-01
DPP4i	SGLT2i	UC_5	2915	0.89 (0.46-1.7)	7.38687e-01	9.233588e-01
GLP1ra	SGLT2i	UC_1	684	1.7 (0.41-7.2)	4.60927e-01	9.233588e-01
GLP1ra	SGLT2i	UC_2	1241	0.59 (0.26-1.3)	2.06761e-01	8.376900e-01
GLP1ra	SGLT2i	UC_3	773	1.5 (0.47-4.7)	5.00924e-01	9.233588e-01
GLP1ra	SGLT2i	UC_4	633	0.75 (0.17-3.3)	7.00338e-01	9.233588e-01
GLP1ra	SGLT2i	UC_5	3010	1 (0.49-2.1)	9.58779e-01	9.754560e-01
Sulfonylurea	DPP4i	UC_1	1680	0.64 (0.32-1.3)	2.11290e-01	8.376900e-01
Sulfonylurea	DPP4i	UC_2	1980	0.9 (0.58-1.4)	6.50170e-01	9.233588e-01
Sulfonylurea	DPP4i	UC_3	2048	0.9 (0.58-1.4)	6.30509e-01	9.233588e-01
Sulfonylurea	DPP4i	UC_4	1237	0.52 (0.26-1)	6.30471e-02	5.930655e-01
Sulfonylurea	DPP4i	UC_5	5651	0.68 (0.48-0.95)	2.41583e-02	5.930655e-01
Sulfonylurea	GLP1ra	UC_1	724	0.77 (0.21-2.9)	7.04224e-01	9.233588e-01
Sulfonylurea	GLP1ra	UC_2	1122	0.87 (0.43-1.7)	6.86223e-01	9.233588e-01
Sulfonylurea	GLP1ra	UC_3	1048	0.41 (0.17-1)	4.93502e-02	5.930655e-01
Sulfonylurea	GLP1ra	UC_4	641	0.5 (0.17-1.5)	2.07628e-01	8.376900e-01
Sulfonylurea	GLP1ra	UC_5	2566	0.75 (0.39-1.5)	4.01864e-01	9.233588e-01
Sulfonylurea	SGLT2i	UC_1	1150	0.63 (0.25-1.6)	3.25911e-01	9.233588e-01
Sulfonylurea	SGLT2i	UC_2	1071	0.52 (0.26-1.1)	7.90754e-02	5.930655e-01
Sulfonylurea	SGLT2i	UC_3	896	1.3 (0.59-2.9)	5.01424e-01	9.233588e-01
Sulfonylurea	SGLT2i	UC_4	732	0.56 (0.23-1.4)	2.23384e-01	8.376900e-01
Sulfonylurea	SGLT2i	UC_5	2881	0.91 (0.52-1.6)	7.32819e-01	9.233588e-01

4.20.2 eFigure: Individual effect size, meta analysis and sensitivity analysis

The forest plot illustrate the effect size of the comparison between DPP4i and GLP1ra at each UC along with the effect size obtained from the random effect meta-analysis across all the UC for outcome Myocardial Infarction

© 2023 Vashisht R et al. *JAMA Network Open.*

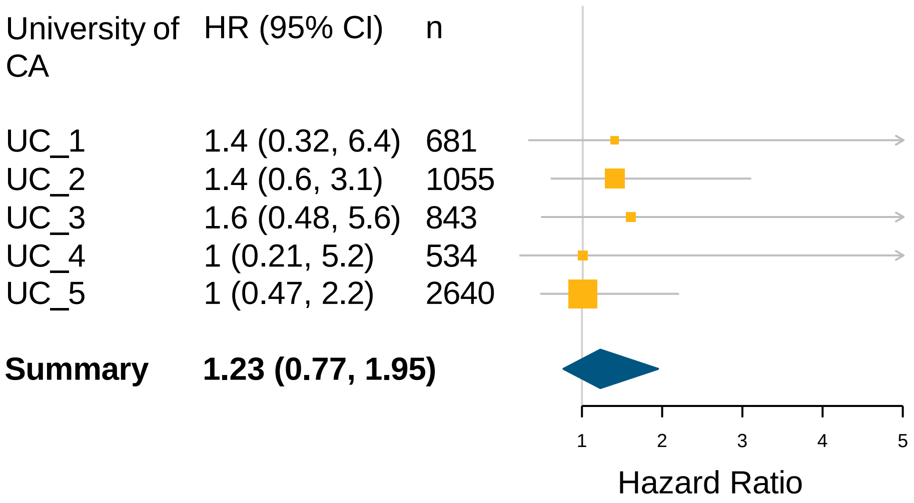

The table below shows the Leave-One-UC-Out diagnostics. The DFFITS value, Cook's distance, Covariance ratio, leave-one-out amount of heteroginity, indicator for influential estimates, comparator and treated groups are provided for each Leave-One-UC-Out analysis. The influential estimate from one UC with respect to pooled estimate are marked as Yes or No, with Yes indicating an influential UC and No otherwise.

eTable 136: Leave-One-UC-Out Sensitivity Analysis

DFFITs	Cook's Dist	Residual Heterogeneity	Influential	Comparator	Treated	UC
0.0583188	0.0034011	0	No	DPP4i	GLP1ra	UC_1
0.2574612	0.0662863	0	No	DPP4i	GLP1ra	UC_2
0.1851090	0.0342654	0	No	DPP4i	GLP1ra	UC_3
-0.0793130	0.0062906	0	No	DPP4i	GLP1ra	UC_4
-0.4914107	0.2414845	0	No	DPP4i	GLP1ra	UC_5

© 2023 Vashisht R et al. *JAMA Network Open.*

The forest plot illustrate the effect size of the comparison between DPP4i and SGLT2i at each UC along with the effect size obtained from the random effect meta-analysis across all the UC for outcome Myocardial Infarction

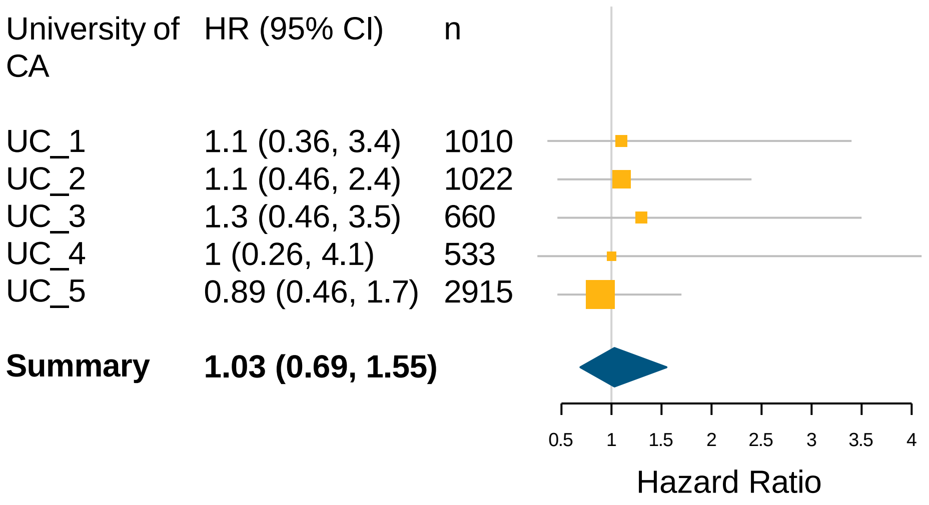

The table below shows the Leave-One-UC-Out diagnostics. The DFFITS value, Cook's distance, Covariance ratio, leave-one-out amount of heteroginity, indicator for influential estimates, comparator and treated groups are provided for each Leave-One-UC-Out analysis. The influential estimate from one UC with respect to pooled estimate are marked as Yes or No, with Yes indicating an influential UC and No otherwise.

eTable 137: Leave-One-UC-Out Sensitivity Analysis

DFFITs	Cook's Dist	Residual Heterogeneity	Influential	Comparator	Treated	UC
0.0455654	0.0020762	0	No	DPP4i	SGLT2i	UC_1
0.0964011	0.0092932	0	No	DPP4i	SGLT2i	UC_2
0.2109307	0.0444918	0	No	DPP4i	SGLT2i	UC_3
-0.0147842	0.0002186	0	No	DPP4i	SGLT2i	UC_4
-0.4491222	0.2017107	0	No	DPP4i	SGLT2i	UC_5

© 2023 Vashisht R et al. *JAMA Network Open.*

The forest plot illustrate the effect size of the comparison between GLP1ra and SGLT2i at each UC along with the effect size obtained from the random effect meta-analysis across all the UC for outcome Myocardial Infarction

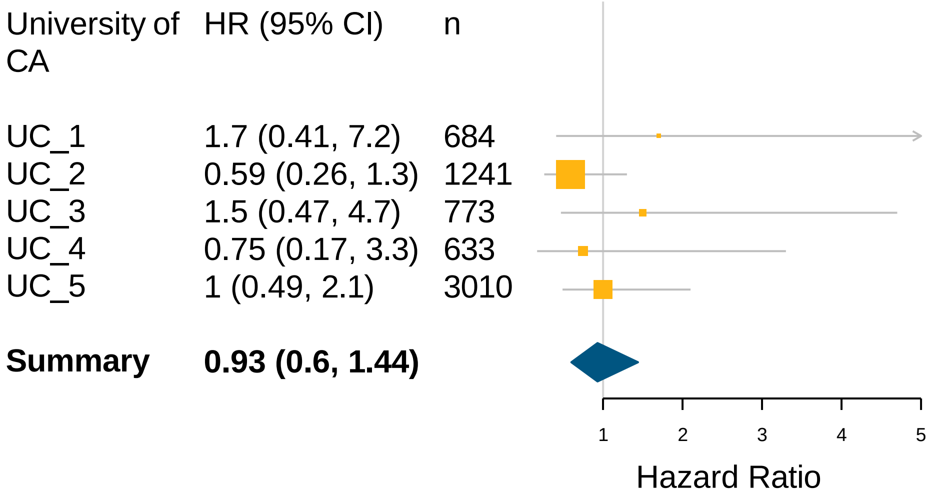

The table below shows the Leave-One-UC-Out diagnostics. The DFFITS value, Cook's distance, Covariance ratio, leave-one-out amount of heteroginity, indicator for influential estimates, comparator and treated groups are provided for each Leave-One-UC-Out analysis. The influential estimate from one UC with respect to pooled estimate are marked as Yes or No, with Yes indicating an influential UC and No otherwise.

eTable 138: Leave-One-UC-Out Sensitivity Analysis

DFFITs	Cook's Dist	Residual Heterogeneity	Influential	Comparator	Treated	UC
0.2817644	0.0793912	0	No	GLP1ra	SGLT2i	UC_1
-0.8667431	0.7512435	0	Yes	GLP1ra	SGLT2i	UC_2
0.3672842	0.1348977	0	No	GLP1ra	SGLT2i	UC_3
-0.0921289	0.0084877	0	No	GLP1ra	SGLT2i	UC_4
0.1922977	0.0369784	0	No	GLP1ra	SGLT2i	UC_5

© 2023 Vashisht R et al. *JAMA Network Open.*

The forest plot illustrate the effect size of the comparison between Sulfonylurea and DPP4i at each UC along with the effect size obtained from the random effect meta-analysis across all the UC for outcome Myocardial Infarction

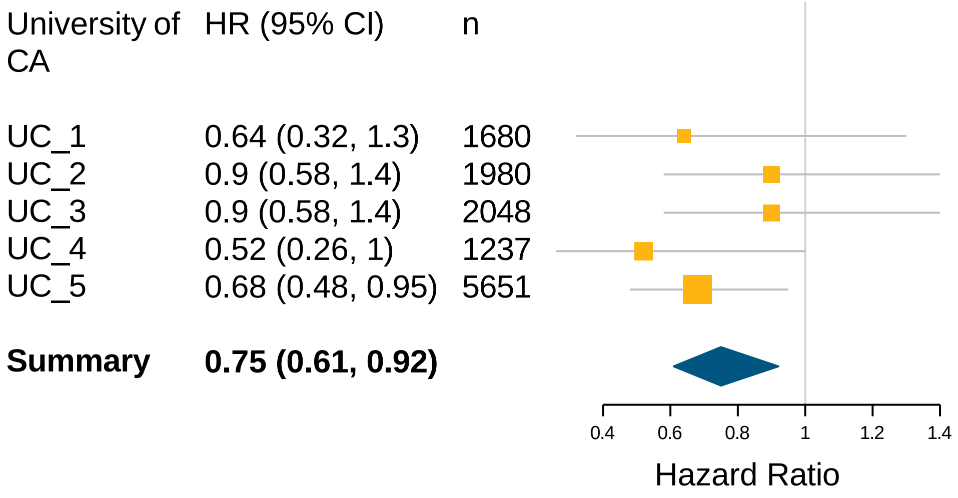

The table below shows the Leave-One-UC-Out diagnostics. The DFFITS value, Cook's distance, Covariance ratio, leave-one-out amount of heteroginity, indicator for influential estimates, comparator and treated groups are provided for each Leave-One-UC-Out analysis. The influential estimate from one UC with respect to pooled estimate are marked as Yes or No, with Yes indicating an influential UC and No otherwise.

eTable 139: Leave-One-UC-Out Sensitivity Analysis

DFFITs	Cook's Dist	Residual Heterogeneity	Influential	Comparator	Treated	UC
-0.1406378	0.0197790	0	No	Sulfonylurea	DPP4i	UC_1
0.5033243	0.2533354	0	No	Sulfonylurea	DPP4i	UC_2
0.5033243	0.2533354	0	No	Sulfonylurea	DPP4i	UC_3
-0.3597296	0.1294054	0	No	Sulfonylurea	DPP4i	UC_4
-0.5227219	0.2732382	0	No	Sulfonylurea	DPP4i	UC_5

© 2023 Vashisht R et al. *JAMA Network Open.*

The forest plot illustrate the effect size of the comparison between Sulfonylurea and GLP1ra at each UC along with the effect size obtained from the random effect meta-analysis across all the UC for outcome Myocardial Infarction

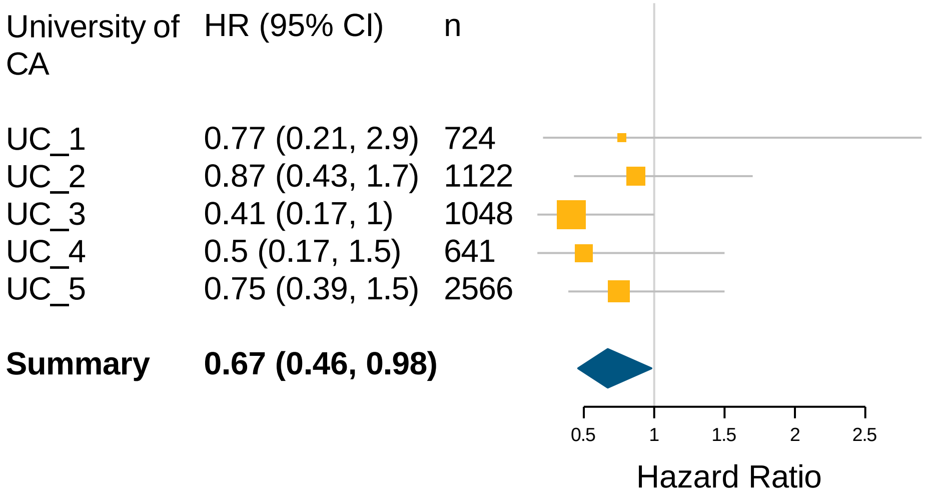

The table below shows the Leave-One-UC-Out diagnostics. The DFFITS value, Cook's distance, Covariance ratio, leave-one-out amount of heteroginity, indicator for influential estimates, comparator and treated groups are provided for each Leave-One-UC-Out analysis. The influential estimate from one UC with respect to pooled estimate are marked as Yes or No, with Yes indicating an influential UC and No otherwise.

eTable 140: Leave-One-UC-Out Sensitivity Analysis

DFFITs	Cook's Dist	Residual Heterogeneity	Influential	Comparator	Treated	UC
0.0644564	0.0041546	0	No	Sulfonylurea	GLP1ra	UC_1
0.5826141	0.3394392	0	No	Sulfonylurea	GLP1ra	UC_2
-0.5670898	0.3215909	0	No	Sulfonylurea	GLP1ra	UC_3
-0.2086068	0.0435168	0	No	Sulfonylurea	GLP1ra	UC_4
0.2648402	0.0701403	0	No	Sulfonylurea	GLP1ra	UC_5

© 2023 Vashisht R et al. *JAMA Network Open.*

The forest plot illustrate the effect size of the comparison between Sulfonylurea and SGLT2i at each UC along with the effect size obtained from the random effect meta-analysis across all the UC for outcome Myocardial Infarction

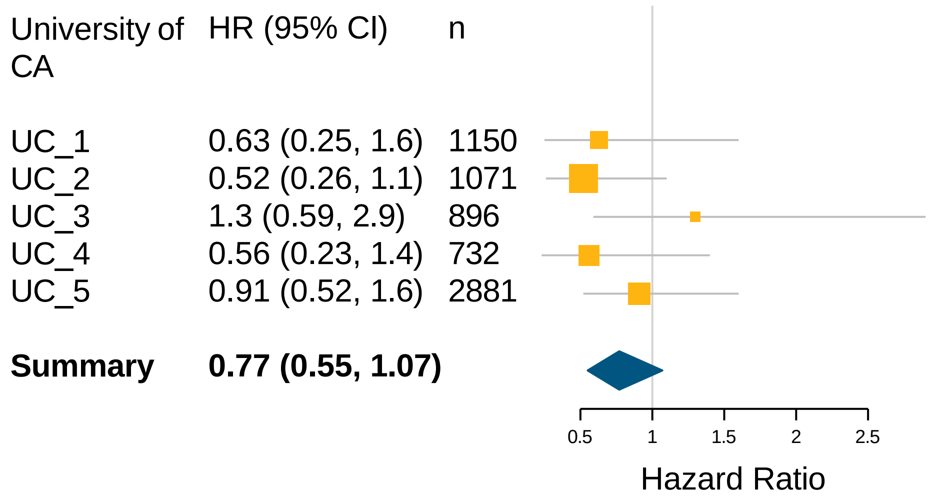

The table below shows the Leave-One-UC-Out diagnostics. The DFFITS value, Cook's distance, Covariance ratio, leave-one-out amount of heteroginity, indicator for influential estimates, comparator and treated groups are provided for each Leave-One-UC-Out analysis. The influential estimate from one UC with respect to pooled estimate are marked as Yes or No, with Yes indicating an influential UC and No otherwise.

eTable 141: Leave-One-UC-Out Sensitivity Analysis

DFFITs	Cook's Dist	Residual Heterogeneity	Influential	Comparator	Treated	UC
-0.1284054	0.0185079	0.0274734	No	Sulfonylurea	SGLT2i	UC_1
-0.6201478	0.3845833	0.0000000	No	Sulfonylurea	SGLT2i	UC_2
0.6549024	0.4288971	0.0000000	No	Sulfonylurea	SGLT2i	UC_3
-0.2712896	0.0776895	0.0118023	No	Sulfonylurea	SGLT2i	UC_4
0.4913374	0.2868828	0.0154843	No	Sulfonylurea	SGLT2i	UC_5

© 2023 Vashisht R et al. *JAMA Network Open.*

4.21 Nausea

4.21.1 eTable: Drug comparision table

Effect size of each drug comparison at each UC health site is tabulated.

eTable 142: Hazard ratios of drug class comparison at each UC

Comparator	Treated	UC	N	Hazard Ratio (95% CI)	P-value	Adjusted P-Value
DPP4i	GLP1ra	UC_1	657	1.1 (0.54-2.3)	7.72798e-01	8.279979e-01
DPP4i	GLP1ra	UC_2	973	1.3 (0.82-2.1)	2.60572e-01	5.583686e-01
DPP4i	GLP1ra	UC_3	787	2 (1.1-3.8)	2.31767e-02	2.317670e-01
DPP4i	GLP1ra	UC_4	527	0.98 (0.42-2.3)	9.59448e-01	9.632840e-01
DPP4i	GLP1ra	UC_5	2559	1.2 (0.81-1.7)	3.72852e-01	6.214200e-01
DPP4i	SGLT2i	UC_1	1001	0.85 (0.41-1.8)	6.68851e-01	7.714289e-01
DPP4i	SGLT2i	UC_2	984	0.7 (0.4-1.2)	2.20605e-01	5.090885e-01
DPP4i	SGLT2i	UC_3	635	1.4 (0.56-3.5)	4.72228e-01	7.078114e-01
DPP4i	SGLT2i	UC_4	527	1.2 (0.42-3.7)	6.94286e-01	7.714289e-01
DPP4i	SGLT2i	UC_5	2849	0.88 (0.6-1.3)	5.31510e-01	7.146065e-01
GLP1ra	SGLT2i	UC_1	675	0.69 (0.33-1.5)	3.29909e-01	6.214200e-01
GLP1ra	SGLT2i	UC_2	1165	0.69 (0.42-1.1)	1.30186e-01	4.450100e-01
GLP1ra	SGLT2i	UC_3	729	0.48 (0.23-0.97)	4.19789e-02	2.518734e-01
GLP1ra	SGLT2i	UC_4	612	0.54 (0.23-1.3)	1.65631e-01	4.450100e-01
GLP1ra	SGLT2i	UC_5	2909	0.65 (0.44-0.98)	3.73755e-02	2.518734e-01
Sulfonylurea	DPP4i	UC_1	1657	1.1 (0.7-1.8)	6.03576e-01	7.242912e-01
Sulfonylurea	DPP4i	UC_2	1896	1.3 (0.91-1.8)	1.59971e-01	4.450100e-01
Sulfonylurea	DPP4i	UC_3	1944	0.76 (0.52-1.1)	1.50525e-01	4.450100e-01
Sulfonylurea	DPP4i	UC_4	1227	1.5 (0.83-2.7)	1.73938e-01	4.450100e-01
Sulfonylurea	DPP4i	UC_5	5549	0.75 (0.59-0.96)	2.01289e-02	2.317670e-01
Sulfonylurea	GLP1ra	UC_1	700	2 (0.93-4.3)	7.70454e-02	3.852270e-01
Sulfonylurea	GLP1ra	UC_2	1056	1.2 (0.79-1.9)	3.56339e-01	6.214200e-01
Sulfonylurea	GLP1ra	UC_3	975	2.1 (1.2-3.7)	1.21718e-02	2.317670e-01
Sulfonylurea	GLP1ra	UC_4	619	1.3 (0.6-2.9)	4.95468e-01	7.078114e-01
Sulfonylurea	GLP1ra	UC_5	2472	1.3 (0.89-1.9)	1.78004e-01	4.450100e-01
Sulfonylurea	SGLT2i	UC_1	1163	0.83 (0.42-1.6)	5.85443e-01	7.242912e-01
Sulfonylurea	SGLT2i	UC_2	1051	0.8 (0.46-1.4)	4.46064e-01	7.043116e-01
Sulfonylurea	SGLT2i	UC_3	883	0.72 (0.37-1.4)	3.46667e-01	6.214200e-01
Sulfonylurea	SGLT2i	UC_4	718	0.98 (0.38-2.5)	9.63284e-01	9.632840e-01
Sulfonylurea	SGLT2i	UC_5	2841	0.88 (0.58-1.3)	5.47865e-01	7.146065e-01

4.21.2 eFigure: Individual effect size, meta analysis and sensitivity analysis

The forest plot illustrate the effect size of the comparison between DPP4i and GLP1ra at each UC along with the effect size obtained from the random effect meta-analysis across all the UC for outcome Nausea

© 2023 Vashisht R et al. *JAMA Network Open.*

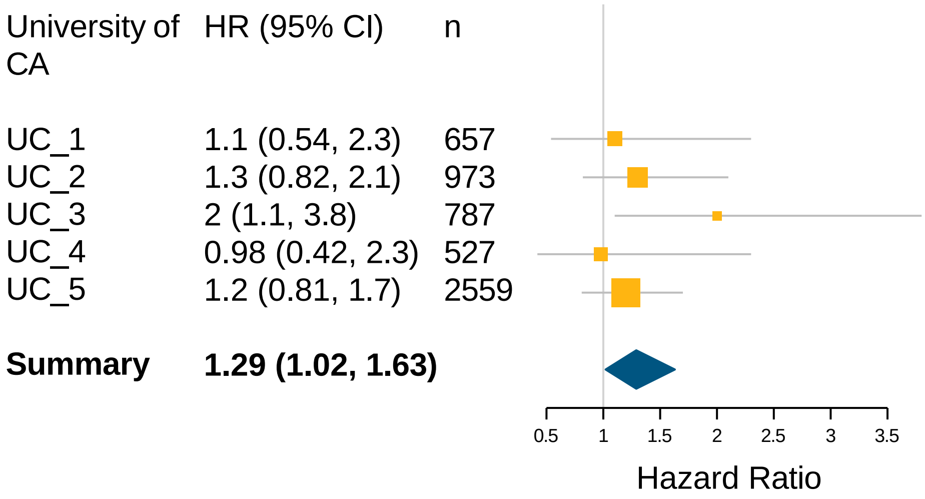

The table below shows the Leave-One-UC-Out diagnostics. The DFFITS value, Cook's distance, Covariance ratio, leave-one-out amount of heteroginity, indicator for influential estimates, comparator and treated groups are provided for each Leave-One-UC-Out analysis. The influential estimate from one UC with respect to pooled estimate are marked as Yes or No, with Yes indicating an influential UC and No otherwise.

eTable 143: Leave-One-UC-Out Sensitivity Analysis

DFFITs	Cook's Dist	Residual Heterogeneity	Influential	Comparator	Treated	UC
-0.1565864	0.0245193	0	No	DPP4i	GLP1ra	UC_1
0.0272809	0.0007442	0	No	DPP4i	GLP1ra	UC_2
0.6262707	0.3922150	0	No	DPP4i	GLP1ra	UC_3
-0.1908642	0.0364291	0	No	DPP4i	GLP1ra	UC_4
-0.4056485	0.1645507	0	No	DPP4i	GLP1ra	UC_5

© 2023 Vashisht R et al. *JAMA Network Open.*

The forest plot illustrate the effect size of the comparison between DPP4i and SGLT2i at each UC along with the effect size obtained from the random effect meta-analysis across all the UC for outcome Nausea

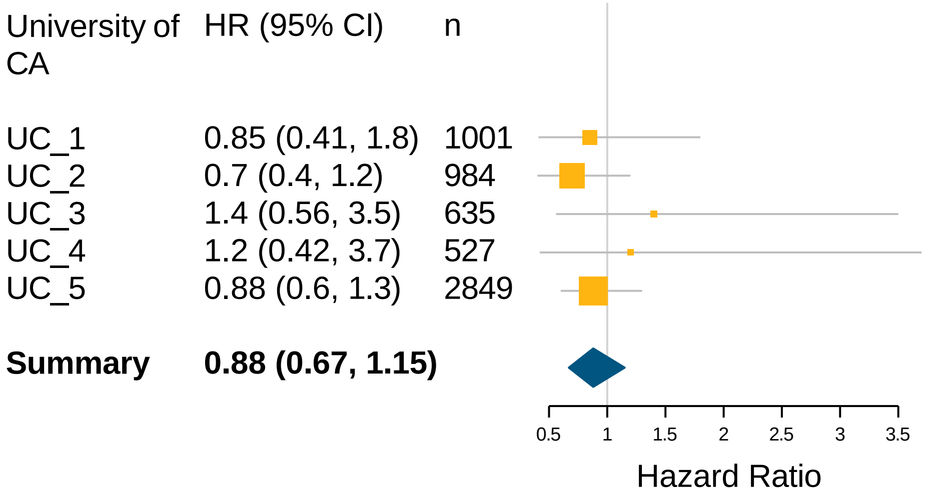

The table below shows the Leave-One-UC-Out diagnostics. The DFFITS value, Cook's distance, Covariance ratio, leave-one-out amount of heteroginity, indicator for influential estimates, comparator and treated groups are provided for each Leave-One-UC-Out analysis. The influential estimate from one UC with respect to pooled estimate are marked as Yes or No, with Yes indicating an influential UC and No otherwise.

eTable 144: Leave-One-UC-Out Sensitivity Analysis

DFFITs	Cook's Dist	Residual Heterogeneity	Influential	Comparator	Treated	UC
-0.0378896	0.0014356	0	No	DPP4i	SGLT2i	UC_1
-0.5233690	0.2739152	0	No	DPP4i	SGLT2i	UC_2
0.3187269	0.1015868	0	No	DPP4i	SGLT2i	UC_3
0.1470941	0.0216367	0	No	DPP4i	SGLT2i	UC_4
0.0033065	0.0000109	0	No	DPP4i	SGLT2i	UC_5

© 2023 Vashisht R et al. *JAMA Network Open.*

The forest plot illustrate the effect size of the comparison between GLP1ra and SGLT2i at each UC along with the effect size obtained from the random effect meta-analysis across all the UC for outcome Nausea

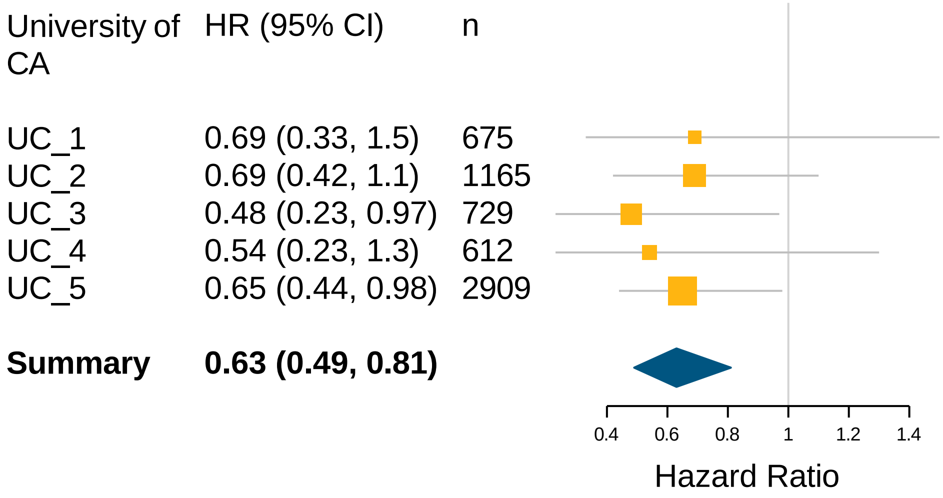

The table below shows the Leave-One-UC-Out diagnostics. The DFFITS value, Cook's distance, Covariance ratio, leave-one-out amount of heteroginity, indicator for influential estimates, comparator and treated groups are provided for each Leave-One-UC-Out analysis. The influential estimate from one UC with respect to pooled estimate are marked as Yes or No, with Yes indicating an influential UC and No otherwise.

eTable 145: Leave-One-UC-Out Sensitivity Analysis

DFFITs	Cook's Dist	Residual Heterogeneity	Influential	Comparator	Treated	UC
0.0878427	0.0077163	0	No	GLP1ra	SGLT2i	UC_1
0.2668900	0.0712303	0	No	GLP1ra	SGLT2i	UC_2
-0.2989636	0.0893793	0	No	GLP1ra	SGLT2i	UC_3
-0.1124114	0.0126363	0	No	GLP1ra	SGLT2i	UC_4
0.1564899	0.0244891	0	No	GLP1ra	SGLT2i	UC_5

© 2023 Vashisht R et al. *JAMA Network Open.*

The forest plot illustrate the effect size of the comparison between Sulfonylurea and DPP4i at each UC along with the effect size obtained from the random effect meta-analysis across all the UC for outcome Nausea

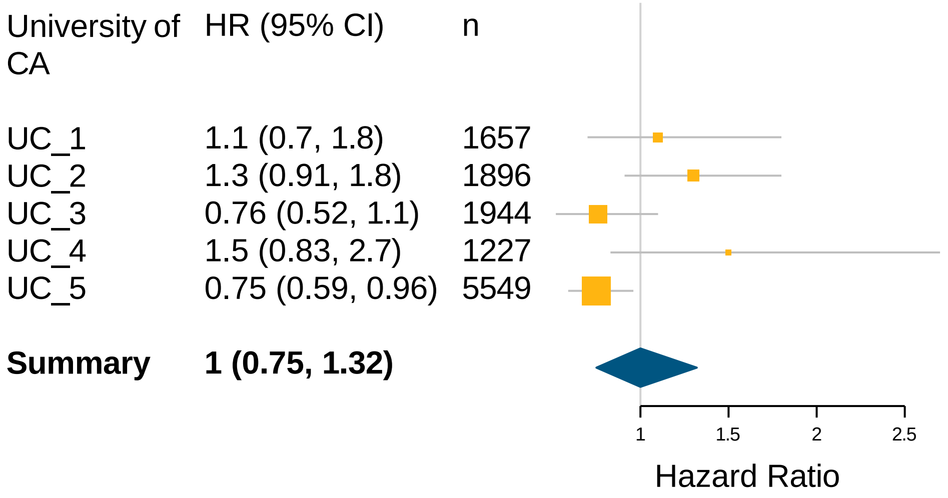

The table below shows the Leave-One-UC-Out diagnostics. The DFFITS value, Cook's distance, Covariance ratio, leave-one-out amount of heteroginity, indicator for influential estimates, comparator and treated groups are provided for each Leave-One-UC-Out analysis. The influential estimate from one UC with respect to pooled estimate are marked as Yes or No, with Yes indicating an influential UC and No otherwise.

eTable 146: Leave-One-UC-Out Sensitivity Analysis

DFFITs	Cook's Dist	Residual Heterogeneity	Influential	Comparator	Treated	UC
0.0885567	0.0090632	0.0798833	No	Sulfonylurea	DPP4i	UC_1
0.7271598	0.4079679	0.0403804	No	Sulfonylurea	DPP4i	UC_2
-0.5073205	0.3074059	0.0803200	No	Sulfonylurea	DPP4i	UC_3
0.4733455	0.2103111	0.0519860	No	Sulfonylurea	DPP4i	UC_4
-0.7936958	0.4787957	0.0428916	Yes	Sulfonylurea	DPP4i	UC_5

© 2023 Vashisht R et al. *JAMA Network Open.*

The forest plot illustrate the effect size of the comparison between Sulfonylurea and GLP1ra at each UC along with the effect size obtained from the random effect meta-analysis across all the UC for outcome Nausea

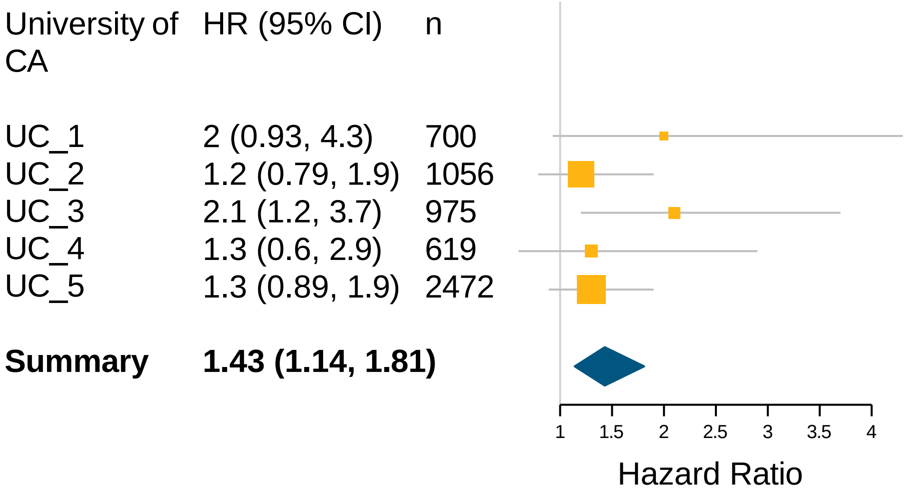

The table below shows the Leave-One-UC-Out diagnostics. The DFFITS value, Cook's distance, Covariance ratio, leave-one-out amount of heteroginity, indicator for influential estimates, comparator and treated groups are provided for each Leave-One-UC-Out analysis. The influential estimate from one UC with respect to pooled estimate are marked as Yes or No, with Yes indicating an influential UC and No otherwise.

eTable 147: Leave-One-UC-Out Sensitivity Analysis

DFFITs	Cook's Dist	Residual Heterogeneity	Influential	Comparator	Treated	UC
0.2835515	0.0804014	0.0000000	No	Sulfonylurea	GLP1ra	UC_1
-0.5837745	0.3407927	0.0000000	No	Sulfonylurea	GLP1ra	UC_2
0.6576148	0.4324572	0.0000000	No	Sulfonylurea	GLP1ra	UC_3
-0.1544910	0.0250814	0.0082167	No	Sulfonylurea	GLP1ra	UC_4
-0.4970072	0.2525719	0.0008419	No	Sulfonylurea	GLP1ra	UC_5

© 2023 Vashisht R et al. *JAMA Network Open.*

The forest plot illustrate the effect size of the comparison between Sulfonylurea and SGLT2i at each UC along with the effect size obtained from the random effect meta-analysis across all the UC for outcome Nausea

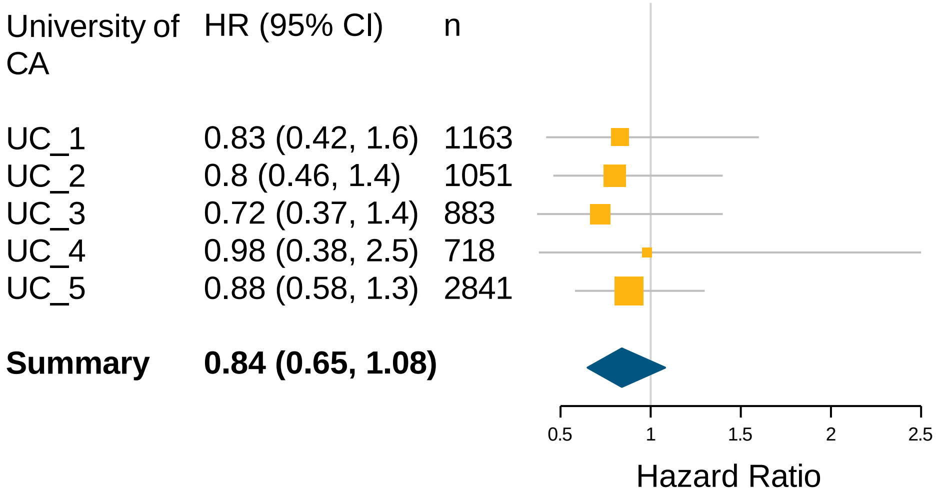

The table below shows the Leave-One-UC-Out diagnostics. The DFFITS value, Cook's distance, Covariance ratio, leave-one-out amount of heteroginity, indicator for influential estimates, comparator and treated groups are provided for each Leave-One-UC-Out analysis. The influential estimate from one UC with respect to pooled estimate are marked as Yes or No, with Yes indicating an influential UC and No otherwise.

eTable 148: Leave-One-UC-Out Sensitivity Analysis

DFFITs	Cook's Dist	Residual Heterogeneity	Influential	Comparator	Treated	UC
-0.0094434	0.0000892	0	No	Sulfonylurea	SGLT2i	UC_1
-0.0914808	0.0083687	0	No	Sulfonylurea	SGLT2i	UC_2
-0.2009629	0.0403861	0	No	Sulfonylurea	SGLT2i	UC_3
0.0981064	0.0096249	0	No	Sulfonylurea	SGLT2i	UC_4
0.2706069	0.0732281	0	No	Sulfonylurea	SGLT2i	UC_5

© 2023 Vashisht R et al. *JAMA Network Open.*

4.22 Neuropathy

4.22.1 eTable: Drug comparision table

Effect size of each drug comparison at each UC health site is tabulated.

eTable 149: Hazard ratios of drug class comparison at each UC

Comparator	Treated	UC	N	Hazard Ratio (95% CI)	P-value	Adjusted P-Value
DPP4i	GLP1ra	UC_1	487	0.98 (0.58-1.7)	9.41038e-01	9.734876e-01
DPP4i	GLP1ra	UC_2	750	1.2 (0.84-1.8)	2.83425e-01	9.567780e-01
DPP4i	GLP1ra	UC_3	566	0.75 (0.45-1.2)	2.53706e-01	9.567780e-01
DPP4i	GLP1ra	UC_4	428	1.1 (0.59-2.2)	6.92525e-01	9.567780e-01
DPP4i	GLP1ra	UC_5	2059	1.1 (0.85-1.3)	5.74093e-01	9.567780e-01
DPP4i	SGLT2i	UC_1	812	0.7 (0.45-1.1)	1.07066e-01	9.387660e-01
DPP4i	SGLT2i	UC_2	789	1.1 (0.74-1.7)	6.16681e-01	9.567780e-01
DPP4i	SGLT2i	UC_3	486	1.1 (0.66-2)	6.34131e-01	9.567780e-01
DPP4i	SGLT2i	UC_4	443	1.1 (0.57-2.1)	7.97315e-01	9.567780e-01
DPP4i	SGLT2i	UC_5	2363	0.97 (0.76-1.2)	7.91686e-01	9.567780e-01
GLP1ra	SGLT2i	UC_1	519	1.1 (0.64-1.8)	7.64108e-01	9.567780e-01
GLP1ra	SGLT2i	UC_2	867	0.73 (0.5-1.1)	1.26586e-01	9.387660e-01
GLP1ra	SGLT2i	UC_3	523	1.5 (0.86-2.5)	1.56461e-01	9.387660e-01
GLP1ra	SGLT2i	UC_4	478	0.91 (0.47-1.7)	7.66750e-01	9.567780e-01
GLP1ra	SGLT2i	UC_5	2321	0.83 (0.65-1.1)	1.23606e-01	9.387660e-01
Sulfonylurea	DPP4i	UC_1	1399	1.1 (0.81-1.4)	6.36400e-01	9.567780e-01
Sulfonylurea	DPP4i	UC_2	1533	0.97 (0.75-1.2)	7.83028e-01	9.567780e-01
Sulfonylurea	DPP4i	UC_3	1558	0.91 (0.71-1.2)	4.31279e-01	9.567780e-01
Sulfonylurea	DPP4i	UC_4	1065	1.1 (0.72-1.6)	7.50534e-01	9.567780e-01
Sulfonylurea	DPP4i	UC_5	4756	0.96 (0.83-1.1)	5.55977e-01	9.567780e-01
Sulfonylurea	GLP1ra	UC_1	558	1.2 (0.74-2)	4.50812e-01	9.567780e-01
Sulfonylurea	GLP1ra	UC_2	837	1 (0.72-1.5)	8.42323e-01	9.719112e-01
Sulfonylurea	GLP1ra	UC_3	723	0.7 (0.46-1.1)	1.15700e-01	9.387660e-01
Sulfonylurea	GLP1ra	UC_4	521	1 (0.57-1.9)	9.14149e-01	9.734876e-01
Sulfonylurea	GLP1ra	UC_5	2020	0.97 (0.77-1.2)	7.92344e-01	9.567780e-01
Sulfonylurea	SGLT2i	UC_1	971	1.1 (0.72-1.7)	6.47767e-01	9.567780e-01
Sulfonylurea	SGLT2i	UC_2	841	0.81 (0.56-1.2)	2.90336e-01	9.567780e-01
Sulfonylurea	SGLT2i	UC_3	672	1 (0.65-1.5)	9.84762e-01	9.847620e-01
Sulfonylurea	SGLT2i	UC_4	611	1 (0.57-1.9)	8.81271e-01	9.734876e-01
Sulfonylurea	SGLT2i	UC_5	2413	0.87 (0.69-1.1)	2.56277e-01	9.567780e-01

4.22.2 eFigure: Individual effect size, meta analysis and sensitivity analysis

The forest plot illustrate the effect size of the comparison between DPP4i and GLP1ra at each UC along with the effect size obtained from the random effect meta-analysis across all the UC for outcome Neuropathy

© 2023 Vashisht R et al. *JAMA Network Open.*

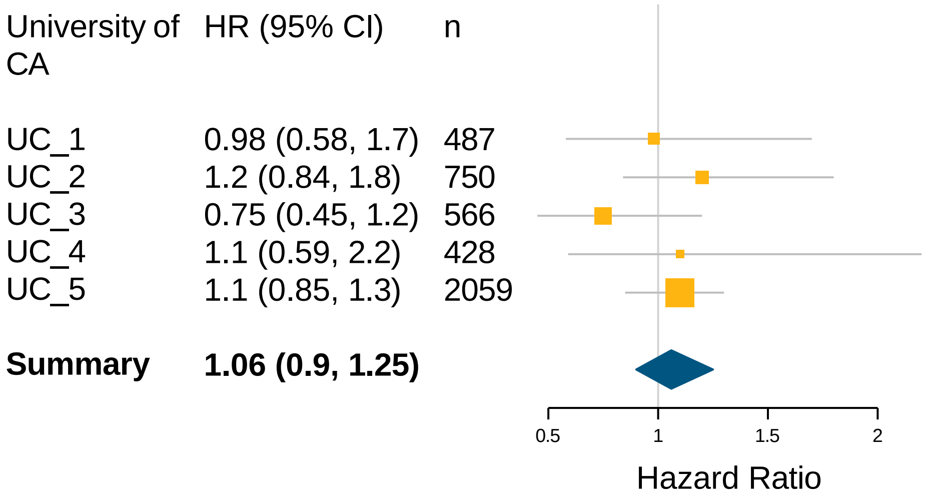

The table below shows the Leave-One-UC-Out diagnostics. The DFFITS value, Cook's distance, Covariance ratio, leave-one-out amount of heteroginity, indicator for influential estimates, comparator and treated groups are provided for each Leave-One-UC-Out analysis. The influential estimate from one UC with respect to pooled estimate are marked as Yes or No, with Yes indicating an influential UC and No otherwise.

eTable 150: Leave-One-UC-Out Sensitivity Analysis

DFFITs	Cook's Dist	Residual Heterogeneity	Influential	Comparator	Treated	UC
-0.0950733	0.0090389	0	No	DPP4i	GLP1ra	UC_1
0.3223644	0.1039188	0	No	DPP4i	GLP1ra	UC_2
-0.5074573	0.2575130	0	No	DPP4i	GLP1ra	UC_3
0.0275462	0.0007588	0	No	DPP4i	GLP1ra	UC_4
0.5763139	0.3321378	0	No	DPP4i	GLP1ra	UC_5

© 2023 Vashisht R et al. *JAMA Network Open.*

The forest plot illustrate the effect size of the comparison between DPP4i and SGLT2i at each UC along with the effect size obtained from the random effect meta-analysis across all the UC for outcome Neuropathy

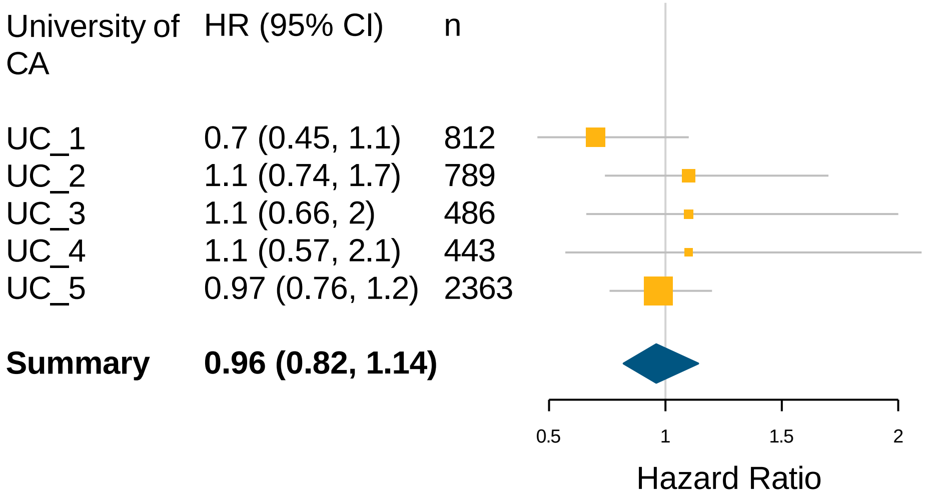

The table below shows the Leave-One-UC-Out diagnostics. The DFFITS value, Cook's distance, Covariance ratio, leave-one-out amount of heteroginity, indicator for influential estimates, comparator and treated groups are provided for each Leave-One-UC-Out analysis. The influential estimate from one UC with respect to pooled estimate are marked as Yes or No, with Yes indicating an influential UC and No otherwise.

eTable 151: Leave-One-UC-Out Sensitivity Analysis

DFFITs	Cook's Dist	Residual Heterogeneity	Influential	Comparator	Treated	UC
-0.6138808	0.3768496	0	No	DPP4i	SGLT2i	UC_1
0.2982058	0.0889267	0	No	DPP4i	SGLT2i	UC_2
0.1547067	0.0239342	0	No	DPP4i	SGLT2i	UC_3
0.1087819	0.0118335	0	No	DPP4i	SGLT2i	UC_4
0.0772293	0.0059644	0	No	DPP4i	SGLT2i	UC_5

© 2023 Vashisht R et al. *JAMA Network Open.*

The forest plot illustrate the effect size of the comparison between GLP1ra and SGLT2i at each UC along with the effect size obtained from the random effect meta-analysis across all the UC for outcome Neuropathy

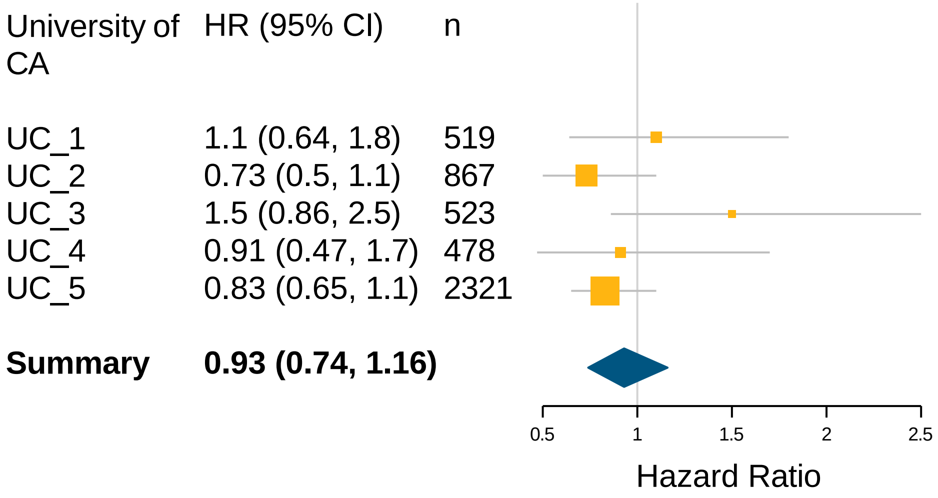

The table below shows the Leave-One-UC-Out diagnostics. The DFFITS value, Cook's distance, Covariance ratio, leave-one-out amount of heteroginity, indicator for influential estimates, comparator and treated groups are provided for each Leave-One-UC-Out analysis. The influential estimate from one UC with respect to pooled estimate are marked as Yes or No, with Yes indicating an influential UC and No otherwise.

eTable 152: Leave-One-UC-Out Sensitivity Analysis

DFFITs	Cook's Dist	Residual Heterogeneity	Influential	Comparator	Treated	UC
0.1854618	0.0384872	0.0294264	No	GLP1ra	SGLT2i	UC_1
-0.6182585	0.3971702	0.0212190	No	GLP1ra	SGLT2i	UC_2
0.8966565	0.6406074	0.0000000	Yes	GLP1ra	SGLT2i	UC_3
-0.1393196	0.0220663	0.0362093	No	GLP1ra	SGLT2i	UC_4
-0.4991432	0.3996782	0.0412044	No	GLP1ra	SGLT2i	UC_5

© 2023 Vashisht R et al. *JAMA Network Open.*

The forest plot illustrate the effect size of the comparison between Sulfonylurea and DPP4i at each UC along with the effect size obtained from the random effect meta-analysis across all the UC for outcome Neuropathy

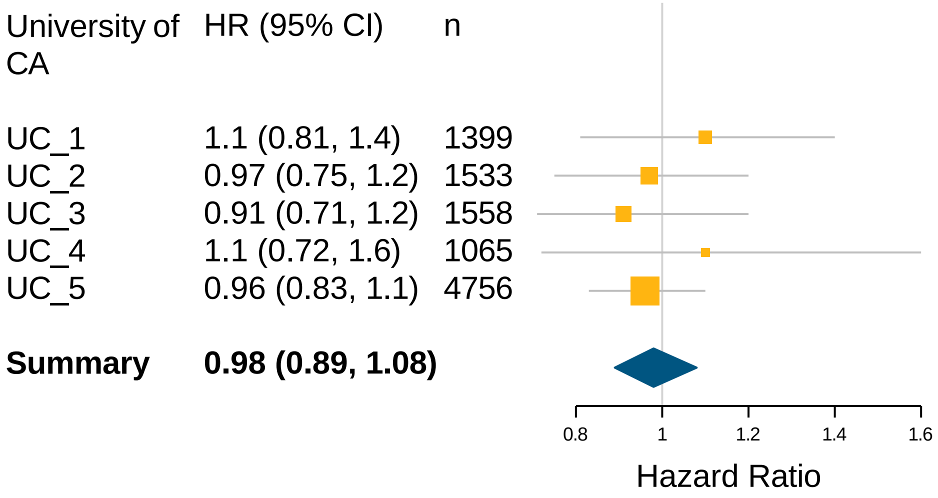

The table below shows the Leave-One-UC-Out diagnostics. The DFFITS value, Cook's distance, Covariance ratio, leave-one-out amount of heteroginity, indicator for influential estimates, comparator and treated groups are provided for each Leave-One-UC-Out analysis. The influential estimate from one UC with respect to pooled estimate are marked as Yes or No, with Yes indicating an influential UC and No otherwise.

eTable 153: Leave-One-UC-Out Sensitivity Analysis

DFFITs	Cook's Dist	Residual Heterogeneity	Influential	Comparator	Treated	UC
0.3440794	0.1183907	0	No	Sulfonylurea	DPP4i	UC_1
-0.0422038	0.0017812	0	No	Sulfonylurea	DPP4i	UC_2
-0.2413672	0.0582581	0	No	Sulfonylurea	DPP4i	UC_3
0.1497035	0.0224111	0	No	Sulfonylurea	DPP4i	UC_4
-0.3888163	0.1511781	0	No	Sulfonylurea	DPP4i	UC_5

© 2023 Vashisht R et al. *JAMA Network Open.*

The forest plot illustrate the effect size of the comparison between Sulfonylurea and GLP1ra at each UC along with the effect size obtained from the random effect meta-analysis across all the UC for outcome Neuropathy

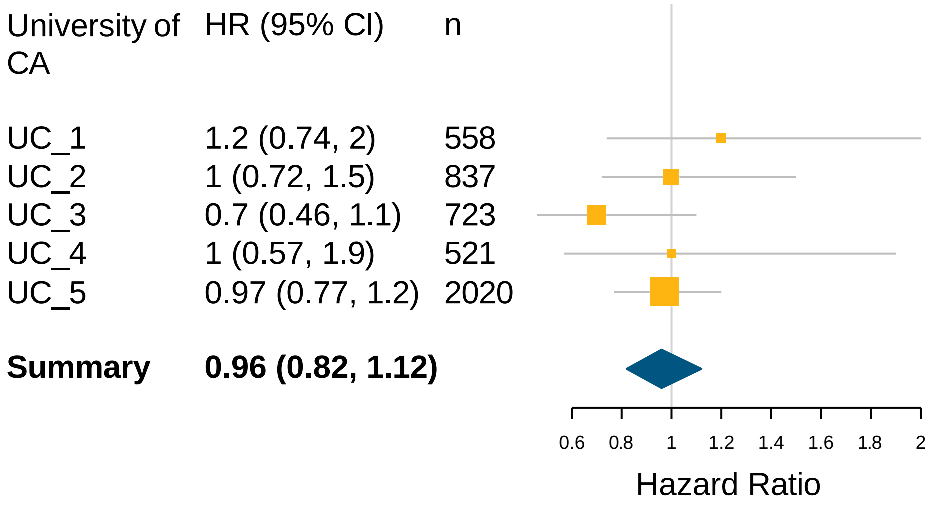

The table below shows the Leave-One-UC-Out diagnostics. The DFFITS value, Cook's distance, Covariance ratio, leave-one-out amount of heteroginity, indicator for influential estimates, comparator and treated groups are provided for each Leave-One-UC-Out analysis. The influential estimate from one UC with respect to pooled estimate are marked as Yes or No, with Yes indicating an influential UC and No otherwise.

eTable 154: Leave-One-UC-Out Sensitivity Analysis

DFFITs	Cook's Dist	Residual Heterogeneity	Influential	Comparator	Treated	UC
0.3168730	0.1004085	0	No	Sulfonylurea	GLP1ra	UC_1
0.1252042	0.0156761	0	No	Sulfonylurea	GLP1ra	UC_2
-0.5886609	0.3465217	0	No	Sulfonylurea	GLP1ra	UC_3
0.0406718	0.0016542	0	No	Sulfonylurea	GLP1ra	UC_4
0.1766198	0.0311945	0	No	Sulfonylurea	GLP1ra	UC_5

© 2023 Vashisht R et al. *JAMA Network Open.*

The forest plot illustrate the effect size of the comparison between Sulfonylurea and SGLT2i at each UC along with the effect size obtained from the random effect meta-analysis across all the UC for outcome Neuropathy

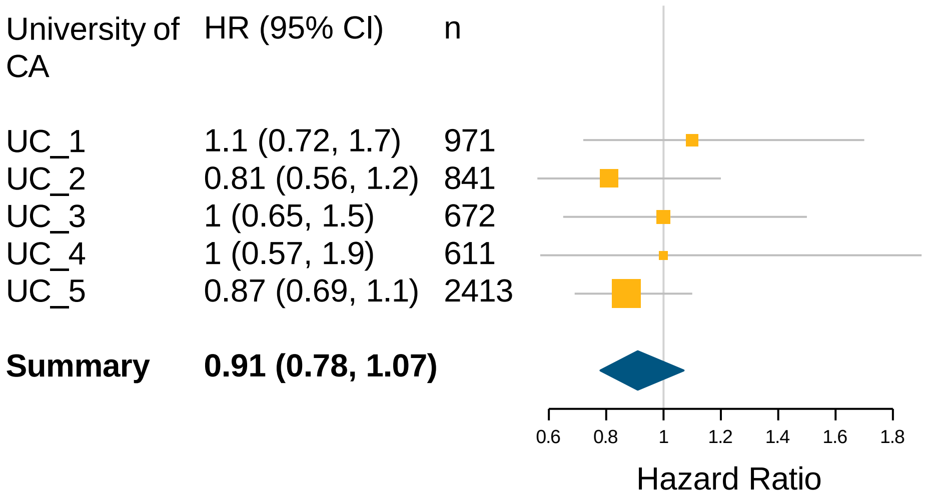

The table below shows the Leave-One-UC-Out diagnostics. The DFFITS value, Cook's distance, Covariance ratio, leave-one-out amount of heteroginity, indicator for influential estimates, comparator and treated groups are provided for each Leave-One-UC-Out analysis. The influential estimate from one UC with respect to pooled estimate are marked as Yes or No, with Yes indicating an influential UC and No otherwise.

eTable 155: Leave-One-UC-Out Sensitivity Analysis

DFFITs	Cook's Dist	Residual Heterogeneity	Influential	Comparator	Treated	UC
0.3634426	0.1320906	0	No	Sulfonylurea	SGLT2i	UC_1
-0.3178550	0.1010318	0	No	Sulfonylurea	SGLT2i	UC_2
0.1871521	0.0350259	0	No	Sulfonylurea	SGLT2i	UC_3
0.0829483	0.0068804	0	No	Sulfonylurea	SGLT2i	UC_4
-0.5430405	0.2948930	0	No	Sulfonylurea	SGLT2i	UC_5

© 2023 Vashisht R et al. *JAMA Network Open.*

4.23 Photosensitivity

4.23.1 eTable: Drug comparision table

Effect size of each drug comparison at each UC health site is tabulated.

eTable 156: Hazard ratios of drug class comparison at each UC

Comparator	Treated	UC	N	Hazard Ratio (95% CI)	P-value	Adjusted P-Value
DPP4i	GLP1ra	UC_1	681	0.94 (0.36-2.4)	9.05476e-01	9.701529e-01
DPP4i	GLP1ra	UC_2	999	1.3 (0.66-2.6)	4.34400e-01	7.240000e-01
DPP4i	GLP1ra	UC_3	780	1.3 (0.65-2.6)	4.71335e-01	7.442132e-01
DPP4i	GLP1ra	UC_4	535	0.25 (0.05-1.2)	7.63404e-02	3.517337e-01
DPP4i	GLP1ra	UC_5	2588	1.1 (0.64-1.9)	7.26705e-01	9.083813e-01
DPP4i	SGLT2i	UC_1	1041	0.29 (0.08-1)	5.81879e-02	3.517337e-01
DPP4i	SGLT2i	UC_2	1007	0.73 (0.33-1.6)	4.30808e-01	7.240000e-01
DPP4i	SGLT2i	UC_3	632	0.52 (0.16-1.7)	2.79837e-01	7.240000e-01
DPP4i	SGLT2i	UC_4	546	0.76 (0.17-3.4)	7.15080e-01	9.083813e-01
DPP4i	SGLT2i	UC_5	2887	1 (0.58-1.9)	8.87317e-01	9.701529e-01
GLP1ra	SGLT2i	UC_1	698	0.11 (0.01-0.89)	3.81749e-02	3.517337e-01
GLP1ra	SGLT2i	UC_2	1144	0.56 (0.25-1.3)	1.66458e-01	5.218980e-01
GLP1ra	SGLT2i	UC_3	732	0.44 (0.18-1.1)	7.01758e-02	3.517337e-01
GLP1ra	SGLT2i	UC_4	639	0.33 (0.03-3.2)	3.34303e-01	7.240000e-01
GLP1ra	SGLT2i	UC_5	2972	0.92 (0.53-1.6)	7.68655e-01	9.223860e-01
Sulfonylurea	DPP4i	UC_1	1720	1.3 (0.77-2.3)	3.03918e-01	7.240000e-01
Sulfonylurea	DPP4i	UC_2	1960	0.85 (0.57-1.3)	4.06579e-01	7.240000e-01
Sulfonylurea	DPP4i	UC_3	1954	1.5 (1-2.3)	4.05296e-02	3.517337e-01
Sulfonylurea	DPP4i	UC_4	1249	2.1 (0.91-5)	8.20712e-02	3.517337e-01
Sulfonylurea	DPP4i	UC_5	5623	1.1 (0.83-1.6)	4.15299e-01	7.240000e-01
Sulfonylurea	GLP1ra	UC_1	733	1.5 (0.54-4.3)	4.30205e-01	7.240000e-01
Sulfonylurea	GLP1ra	UC_2	1070	1.2 (0.66-2.2)	5.31367e-01	7.590957e-01
Sulfonylurea	GLP1ra	UC_3	974	2.1 (1-4.6)	4.68796e-02	3.517337e-01
Sulfonylurea	GLP1ra	UC_4	644	1 (0.21-5.2)	9.62091e-01	9.952666e-01
Sulfonylurea	GLP1ra	UC_5	2523	1 (0.59-1.8)	9.03147e-01	9.701529e-01
Sulfonylurea	SGLT2i	UC_1	1203	0.4 (0.11-1.5)	1.73966e-01	5.218980e-01
Sulfonylurea	SGLT2i	UC_2	1055	0.78 (0.36-1.7)	5.20694e-01	7.590957e-01
Sulfonylurea	SGLT2i	UC_3	881	2.2 (0.72-6.6)	1.65768e-01	5.218980e-01
Sulfonylurea	SGLT2i	UC_4	752	6.1e+08 (0-Inf)	9.98854e-01	9.988540e-01
Sulfonylurea	SGLT2i	UC_5	2895	1.1 (0.64-2)	6.70523e-01	9.083813e-01

4.23.2 eFigure: Individual effect size, meta analysis and sensitivity analysis

The forest plot illustrate the effect size of the comparison between DPP4i and GLP1ra at each UC along with the effect size obtained from the random effect meta-analysis across all the UC for outcome Photosensitivity

© 2023 Vashisht R et al. *JAMA Network Open.*

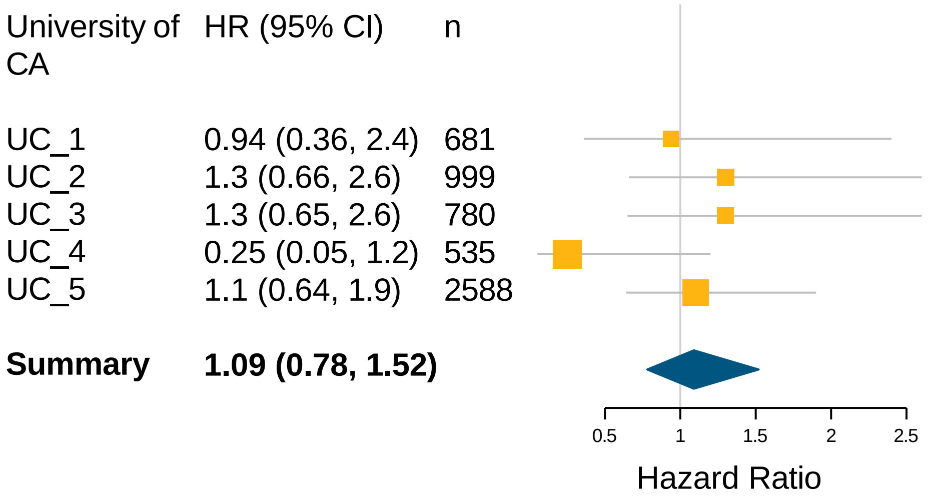

The table below shows the Leave-One-UC-Out diagnostics. The DFFITS value, Cook's distance, Covariance ratio, leave-one-out amount of heteroginity, indicator for influential estimates, comparator and treated groups are provided for each Leave-One-UC-Out analysis. The influential estimate from one UC with respect to pooled estimate are marked as Yes or No, with Yes indicating an influential UC and No otherwise.

eTable 157: Leave-One-UC-Out Sensitivity Analysis

DFFITs	Cook's Dist	Residual	Heterogeneity	Influential	Comparator	Treated	UC
-0.0067619	0.0000539		0.0416514	No	DPP4i	GLP1ra	UC_1
0.4063128	0.2157007		0.0375021	No	DPP4i	GLP1ra	UC_2
0.4009469	0.2092733		0.0377448	No	DPP4i	GLP1ra	UC_3
-0.4067404	0.1654377		0.0000000	No	DPP4i	GLP1ra	UC_4
0.2062602	0.0782181		0.0646168	No	DPP4i	GLP1ra	UC_5

© 2023 Vashisht R et al. *JAMA Network Open.*

The forest plot illustrate the effect size of the comparison between DPP4i and SGLT2i at each UC along with the effect size obtained from the random effect meta-analysis across all the UC for outcome Photosensitivity

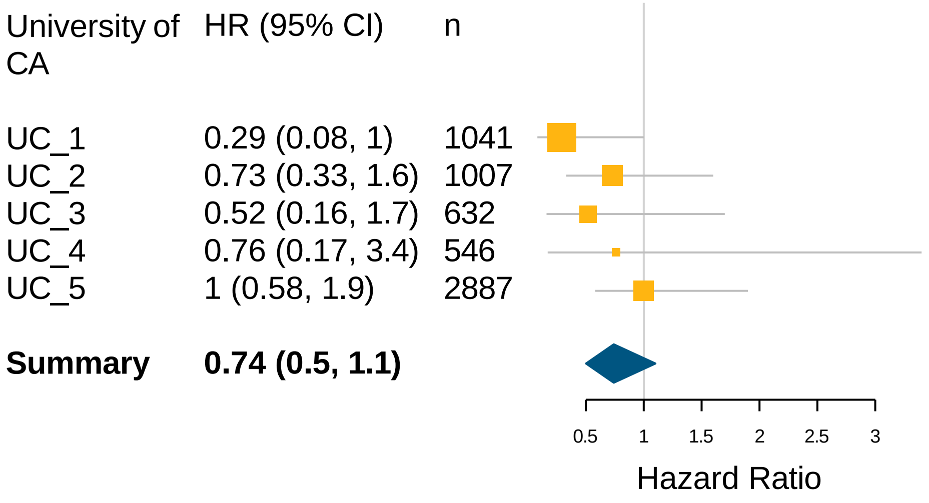

The table below shows the Leave-One-UC-Out diagnostics. The DFFITS value, Cook's distance, Covariance ratio, leave-one-out amount of heteroginity, indicator for influential estimates, comparator and treated groups are provided for each Leave-One-UC-Out analysis. The influential estimate from one UC with respect to pooled estimate are marked as Yes or No, with Yes indicating an influential UC and No otherwise.

eTable 158: Leave-One-UC-Out Sensitivity Analysis

DFFITs	Cook's Dist	Residual Heterogeneity	Influential	Comparator	Treated	UC
-0.5179037	0.2682242	0.0000000	No	DPP4i	SGLT2i	UC_1
0.2173852	0.0606095	0.0458298	No	DPP4i	SGLT2i	UC_2
-0.1918152	0.0374142	0.0061352	No	DPP4i	SGLT2i	UC_3
0.1693378	0.0302480	0.0320326	No	DPP4i	SGLT2i	UC_4
1.2321340	1.5181542	0.0000000	Yes	DPP4i	SGLT2i	UC_5

© 2023 Vashisht R et al. *JAMA Network Open.*

The forest plot illustrate the effect size of the comparison between GLP1ra and SGLT2i at each UC along with the effect size obtained from the random effect meta-analysis across all the UC for outcome Photosensitivity

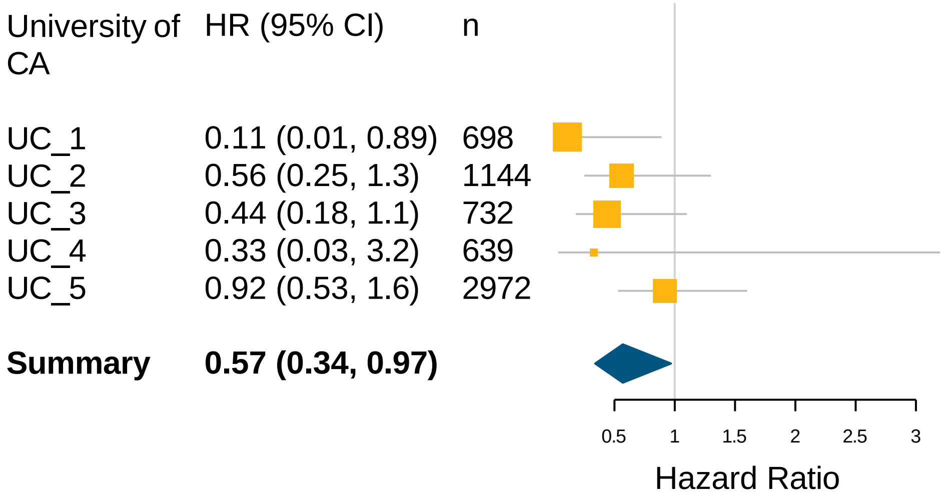

The table below shows the Leave-One-UC-Out diagnostics. The DFFITS value, Cook's distance, Covariance ratio, leave-one-out amount of heteroginity, indicator for influential estimates, comparator and treated groups are provided for each Leave-One-UC-Out analysis. The influential estimate from one UC with respect to pooled estimate are marked as Yes or No, with Yes indicating an influential UC and No otherwise.

eTable 159: Leave-One-UC-Out Sensitivity Analysis

DFFITs	Cook's Dist	Residual Heterogeneity	Influential	Comparator	Treated	UC
-0.6831692	0.4302853	0.0000000	No	GLP1ra	SGLT2i	UC_1
0.3267882	0.1698499	0.2555613	No	GLP1ra	SGLT2i	UC_2
-0.0795196	0.0077731	0.1656770	No	GLP1ra	SGLT2i	UC_3
0.0111888	0.0001296	0.1459710	No	GLP1ra	SGLT2i	UC_4
1.5265730	1.0612829	0.0000000	Yes	GLP1ra	SGLT2i	UC_5

© 2023 Vashisht R et al. *JAMA Network Open.*

The forest plot illustrate the effect size of the comparison between Sulfonylurea and DPP4i at each UC along with the effect size obtained from the random effect meta-analysis across all the UC for outcome Photosensitivity

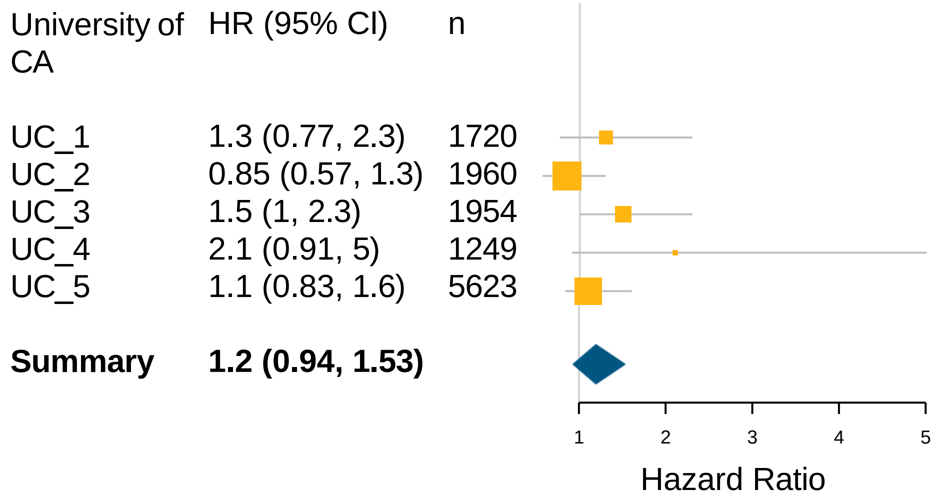

The table below shows the Leave-One-UC-Out diagnostics. The DFFITS value, Cook's distance, Covariance ratio, leave-one-out amount of heteroginity, indicator for influential estimates, comparator and treated groups are provided for each Leave-One-UC-Out analysis. The influential estimate from one UC with respect to pooled estimate are marked as Yes or No, with Yes indicating an influential UC and No otherwise.

eTable 160: Leave-One-UC-Out Sensitivity Analysis

DFFITs	Cook's Dist	Residual Heterogeneity	Influential	Comparator	Treated	UC
0.0259246	0.0008070	0.0444111	No	Sulfonylurea	DPP4i	UC_1
-0.7690867	0.3836789	0.0000000	No	Sulfonylurea	DPP4i	UC_2
0.5606378	0.3019211	0.0212366	No	Sulfonylurea	DPP4i	UC_3
0.3867970	0.1420150	0.0131527	No	Sulfonylurea	DPP4i	UC_4
-0.3365653	0.1856860	0.0571993	No	Sulfonylurea	DPP4i	UC_5

© 2023 Vashisht R et al. *JAMA Network Open.*

The forest plot illustrate the effect size of the comparison between Sulfonylurea and GLP1ra at each UC along with the effect size obtained from the random effect meta-analysis across all the UC for outcome Photosensitivity

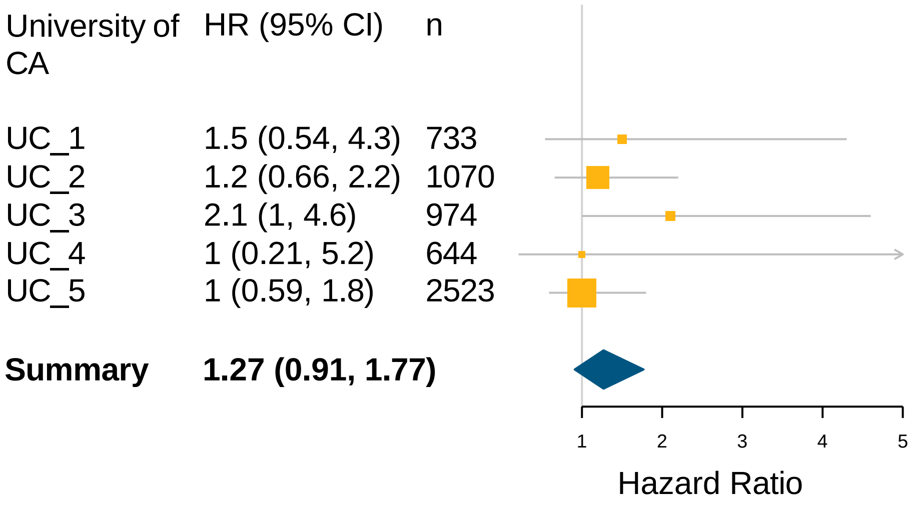

The table below shows the Leave-One-UC-Out diagnostics. The DFFITS value, Cook's distance, Covariance ratio, leave-one-out amount of heteroginity, indicator for influential estimates, comparator and treated groups are provided for each Leave-One-UC-Out analysis. The influential estimate from one UC with respect to pooled estimate are marked as Yes or No, with Yes indicating an influential UC and No otherwise.

eTable 161: Leave-One-UC-Out Sensitivity Analysis

DFFITs	Cook's Dist	Residual Heterogeneity	Influential	Comparator	Treated	UC
0.1125471	0.0126668	0	No	Sulfonylurea	GLP1ra	UC_1
-0.1474773	0.0217495	0	No	Sulfonylurea	GLP1ra	UC_2
0.6967835	0.4855073	0	Yes	Sulfonylurea	GLP1ra	UC_3
-0.0633649	0.0040151	0	No	Sulfonylurea	GLP1ra	UC_4
-0.7804427	0.6090908	0	Yes	Sulfonylurea	GLP1ra	UC_5

© 2023 Vashisht R et al. *JAMA Network Open.*

The forest plot illustrate the effect size of the comparison between Sulfonylurea and SGLT2i at each UC along with the effect size obtained from the random effect meta-analysis across all the UC for outcome Photosensitivity

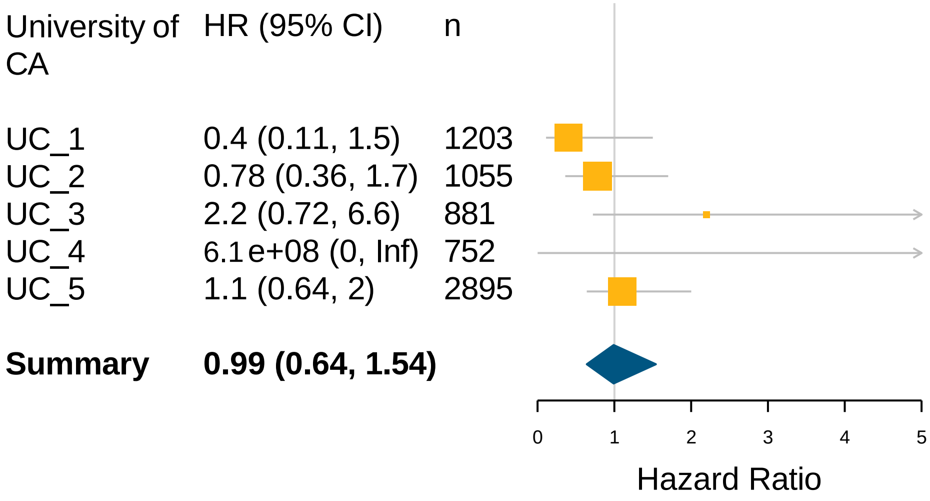

The table below shows the Leave-One-UC-Out diagnostics. The DFFITS value, Cook's distance, Covariance ratio, leave-one-out amount of heteroginity, indicator for influential estimates, comparator and treated groups are provided for each Leave-One-UC-Out analysis. The influential estimate from one UC with respect to pooled estimate are marked as Yes or No, with Yes indicating an influential UC and No otherwise.

eTable 162: Leave-One-UC-Out Sensitivity Analysis

DFFITs	Cook's Dist	Residual Heterogeneity	Influential	Comparator	Treated	UC
-0.4669006	0.2077026	0.0000000	No	Sulfonylurea	SGLT2i	UC_1
-0.3063626	0.1338319	0.0981806	No	Sulfonylurea	SGLT2i	UC_2
0.5351299	0.2679003	0.0000000	No	Sulfonylurea	SGLT2i	UC_3
NaN	0.0019722	0.0873645	No	Sulfonylurea	SGLT2i	UC_4
0.2600178	0.1539026	0.1579621	No	Sulfonylurea	SGLT2i	UC_5

© 2023 Vashisht R et al. *JAMA Network Open.*

4.24 Renal Failure

4.24.1 eTable: Drug comparision table

Effect size of each drug comparison at each UC health site is tabulated.

eTable 163: Hazard ratios of drug class comparison at each UC

Comparator	Treated	UC	N	Hazard Ratio (95% CI)	P-value	Adjusted P-Value
DPP4i	GLP1ra	UC_1	636	0.78 (0.38-1.6)	4.86447e-01	6.919964e-01
DPP4i	GLP1ra	UC_2	930	0.69 (0.42-1.1)	1.49065e-01	3.587423e-01
DPP4i	GLP1ra	UC_3	765	0.49 (0.28-0.87)	1.41068e-02	1.410680e-01
DPP4i	GLP1ra	UC_4	480	0.86 (0.42-1.8)	6.79924e-01	7.860789e-01
DPP4i	GLP1ra	UC_5	2481	1.2 (0.83-1.6)	3.84483e-01	6.919964e-01
DPP4i	SGLT2i	UC_1	926	0.57 (0.34-0.97)	3.92498e-02	2.001694e-01
DPP4i	SGLT2i	UC_2	889	0.62 (0.39-1)	4.93183e-02	2.001694e-01
DPP4i	SGLT2i	UC_3	604	0.79 (0.43-1.5)	4.58635e-01	6.919964e-01
DPP4i	SGLT2i	UC_4	457	0.81 (0.4-1.7)	5.69788e-01	7.432017e-01
DPP4i	SGLT2i	UC_5	2747	0.97 (0.71-1.3)	8.64377e-01	8.941831e-01
GLP1ra	SGLT2i	UC_1	639	0.76 (0.35-1.7)	4.88507e-01	6.919964e-01
GLP1ra	SGLT2i	UC_2	1092	1 (0.61-1.7)	9.36638e-01	9.366380e-01
GLP1ra	SGLT2i	UC_3	719	3.6 (1.4-9)	5.85023e-03	1.233184e-01
GLP1ra	SGLT2i	UC_4	574	0.78 (0.37-1.6)	5.07464e-01	6.919964e-01
GLP1ra	SGLT2i	UC_5	2851	0.7 (0.5-0.99)	4.14175e-02	2.001694e-01
Sulfonylurea	DPP4i	UC_1	1550	0.83 (0.61-1.1)	2.48634e-01	4.972680e-01
Sulfonylurea	DPP4i	UC_2	1767	1 (0.79-1.4)	7.79204e-01	8.348614e-01
Sulfonylurea	DPP4i	UC_3	1882	0.9 (0.69-1.2)	4.36620e-01	6.919964e-01
Sulfonylurea	DPP4i	UC_4	1079	0.93 (0.63-1.4)	7.07471e-01	7.860789e-01
Sulfonylurea	DPP4i	UC_5	5140	0.96 (0.8-1.2)	6.87575e-01	7.860789e-01
Sulfonylurea	GLP1ra	UC_1	689	0.64 (0.35-1.2)	1.55455e-01	3.587423e-01
Sulfonylurea	GLP1ra	UC_2	1024	0.7 (0.44-1.1)	1.32551e-01	3.587423e-01
Sulfonylurea	GLP1ra	UC_3	973	0.6 (0.35-1)	5.33785e-02	2.001694e-01
Sulfonylurea	GLP1ra	UC_4	563	0.44 (0.24-0.81)	8.22123e-03	1.233184e-01
Sulfonylurea	GLP1ra	UC_5	2391	0.87 (0.63-1.2)	3.97527e-01	6.919964e-01
Sulfonylurea	SGLT2i	UC_1	1091	0.64 (0.38-1.1)	9.16209e-02	2.748627e-01
Sulfonylurea	SGLT2i	UC_2	957	0.6 (0.38-0.97)	3.71866e-02	2.001694e-01
Sulfonylurea	SGLT2i	UC_3	831	0.9 (0.53-1.5)	7.00865e-01	7.860789e-01
Sulfonylurea	SGLT2i	UC_4	630	0.7 (0.39-1.3)	2.36351e-01	4.972680e-01
Sulfonylurea	SGLT2i	UC_5	2731	0.75 (0.54-1)	7.79175e-02	2.597250e-01

4.24.2 eFigure: Individual effect size, meta analysis and sensitivity analysis

The forest plot illustrate the effect size of the comparison between DPP4i and GLP1ra at each UC along with the effect size obtained from the random effect meta-analysis across all the UC for outcome Renal Failure

© 2023 Vashisht R et al. *JAMA Network Open.*

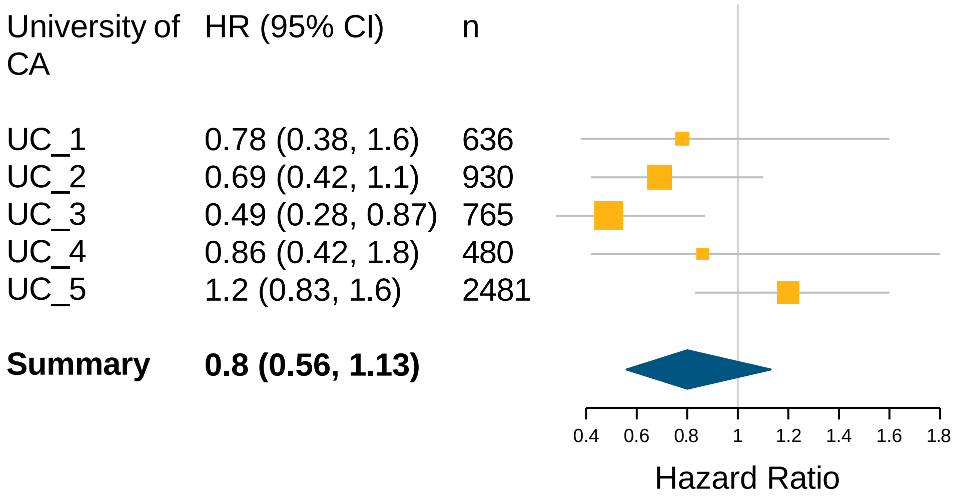

The table below shows the Leave-One-UC-Out diagnostics. The DFFITS value, Cook's distance, Covariance ratio, leave-one-out amount of heteroginity, indicator for influential estimates, comparator and treated groups are provided for each Leave-One-UC-Out analysis. The influential estimate from one UC with respect to pooled estimate are marked as Yes or No, with Yes indicating an influential UC and No otherwise.

eTable 164: Leave-One-UC-Out Sensitivity Analysis

DFFITs	Cook's Dist	Residual Heterogeneity	Influential	Comparator	Treated	UC
0.0309443	0.0011122	0.1171518	No	DPP4i	GLP1ra	UC_1
-0.1493713	0.0280428	0.1187556	No	DPP4i	GLP1ra	UC_2
-1.0368507	0.6947249	0.0234992	Yes	DPP4i	GLP1ra	UC_3
0.1253125	0.0183073	0.1186379	No	DPP4i	GLP1ra	UC_4
2.0098063	1.0276702	0.0000000	Yes	DPP4i	GLP1ra	UC_5

© 2023 Vashisht R et al. *JAMA Network Open.*

The forest plot illustrate the effect size of the comparison between DPP4i and SGLT2i at each UC along with the effect size obtained from the random effect meta-analysis across all the UC for outcome Renal Failure

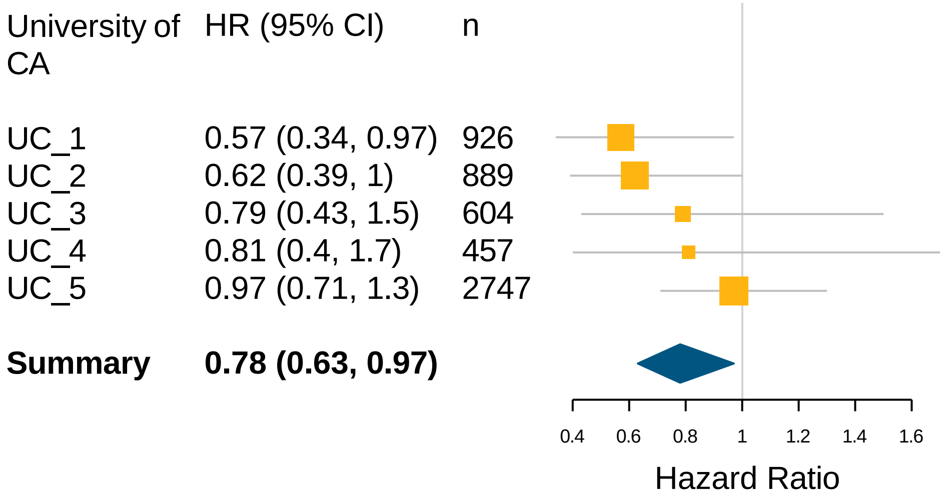

The table below shows the Leave-One-UC-Out diagnostics. The DFFITS value, Cook's distance, Covariance ratio, leave-one-out amount of heteroginity, indicator for influential estimates, comparator and treated groups are provided for each Leave-One-UC-Out analysis. The influential estimate from one UC with respect to pooled estimate are marked as Yes or No, with Yes indicating an influential UC and No otherwise.

eTable 165: Leave-One-UC-Out Sensitivity Analysis

DFFITs	Cook's Dist	Residual Heterogeneity	Influential	Comparator	Treated	UC
-0.6444205	0.3911900	0.0000000	No	DPP4i	SGLT2i	UC_1
-0.6169738	0.3561256	0.0004019	No	DPP4i	SGLT2i	UC_2
0.2493651	0.0740636	0.0246562	No	DPP4i	SGLT2i	UC_3
0.2592265	0.0762196	0.0232866	No	DPP4i	SGLT2i	UC_4
1.6457909	2.2858050	0.0000000	Yes	DPP4i	SGLT2i	UC_5

© 2023 Vashisht R et al. *JAMA Network Open.*

The forest plot illustrate the effect size of the comparison between GLP1ra and SGLT2i at each UC along with the effect size obtained from the random effect meta-analysis across all the UC for outcome Renal Failure

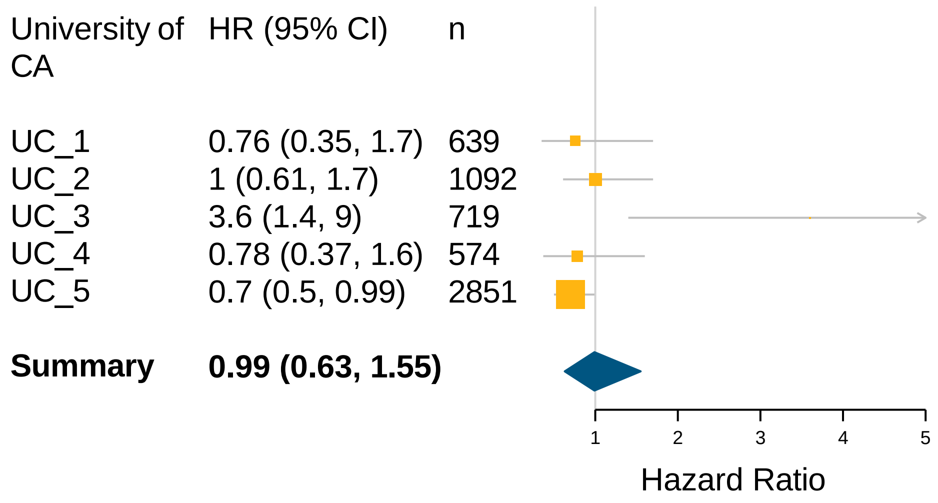

The table below shows the Leave-One-UC-Out diagnostics. The DFFITS value, Cook's distance, Covariance ratio, leave-one-out amount of heteroginity, indicator for influential estimates, comparator and treated groups are provided for each Leave-One-UC-Out analysis. The influential estimate from one UC with respect to pooled estimate are marked as Yes or No, with Yes indicating an influential UC and No otherwise.

eTable 166: Leave-One-UC-Out Sensitivity Analysis

DFFITs	Cook's Dist	Residual Heterogeneity	Influential	Comparator	Treated	UC
-0.2898931	0.0994301	0.2186304	No	GLP1ra	SGLT2i	UC_1
-0.1047145	0.0167279	0.2794392	No	GLP1ra	SGLT2i	UC_2
1.3421757	1.0544665	0.0000000	Yes	GLP1ra	SGLT2i	UC_3
-0.2888163	0.1021660	0.2268695	No	GLP1ra	SGLT2i	UC_4
-0.5418259	0.3940960	0.2246738	No	GLP1ra	SGLT2i	UC_5

The forest plot illustrate the effect size of the comparison between Sulfonylurea and DPP4i at each UC along with the effect size obtained from the random effect meta-analysis across all the UC for outcome Renal Failure

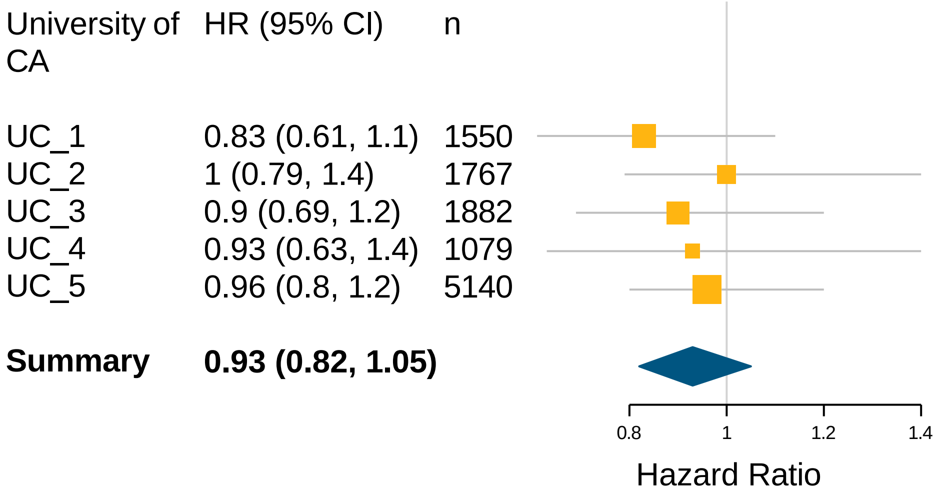

The table below shows the Leave-One-UC-Out diagnostics. The DFFITS value, Cook's distance, Covariance ratio, leave-one-out amount of heteroginity, indicator for influential estimates, comparator and treated groups are provided for each Leave-One-UC-Out analysis. The influential estimate from one UC with respect to pooled estimate are marked as Yes or No, with Yes indicating an influential UC and No otherwise.

eTable 167: Leave-One-UC-Out Sensitivity Analysis

DFFITs	Cook's Dist	Residual Heterogeneity	Influential	Comparator	Treated	UC
-0.3730213	0.1391449	0	No	Sulfonylurea	DPP4i	UC_1
0.2626902	0.0690061	0	No	Sulfonylurea	DPP4i	UC_2
-0.1223567	0.0149712	0	No	Sulfonylurea	DPP4i	UC_3
0.0019155	0.0000037	0	No	Sulfonylurea	DPP4i	UC_4
0.2991879	0.0895134	0	No	Sulfonylurea	DPP4i	UC_5

© 2023 Vashisht R et al. *JAMA Network Open.*

The forest plot illustrate the effect size of the comparison between Sulfonylurea and GLP1ra at each UC along with the effect size obtained from the random effect meta-analysis across all the UC for outcome Renal Failure

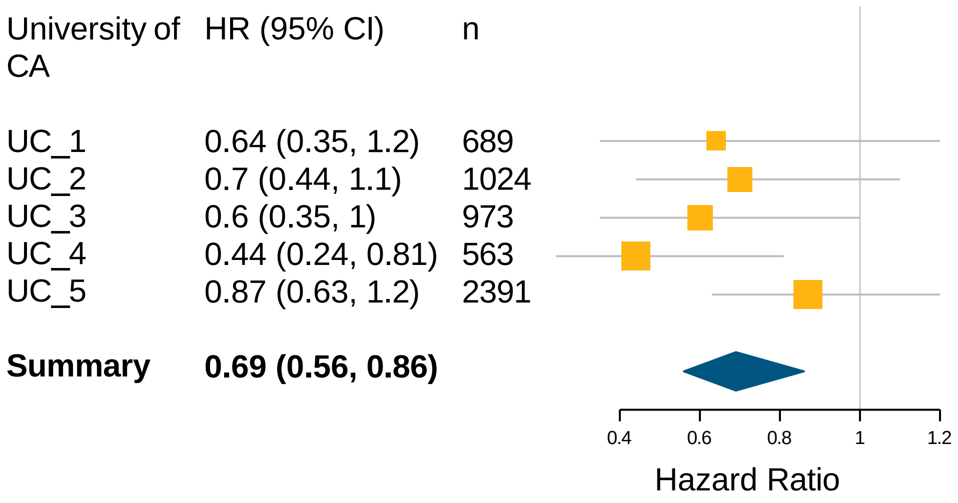

The table below shows the Leave-One-UC-Out diagnostics. The DFFITS value, Cook's distance, Covariance ratio, leave-one-out amount of heteroginity, indicator for influential estimates, comparator and treated groups are provided for each Leave-One-UC-Out analysis. The influential estimate from one UC with respect to pooled estimate are marked as Yes or No, with Yes indicating an influential UC and No otherwise.

eTable 168: Leave-One-UC-Out Sensitivity Analysis

DFFITs	Cook's Dist	Residual Heterogeneity	Influential	Comparator	Treated	UC
0.1109637	0.0144184	0.0239707	No	Sulfonylurea	GLP1ra	UC_1
0.2865192	0.1145472	0.0300326	No	Sulfonylurea	GLP1ra	UC_2
-0.0657324	0.0050823	0.0197505	No	Sulfonylurea	GLP1ra	UC_3
-0.6683122	0.4202675	0.0000000	No	Sulfonylurea	GLP1ra	UC_4
1.3679308	1.5292420	0.0000000	Yes	Sulfonylurea	GLP1ra	UC_5

© 2023 Vashisht R et al. *JAMA Network Open.*

The forest plot illustrate the effect size of the comparison between Sulfonylurea and SGLT2i at each UC along with the effect size obtained from the random effect meta-analysis across all the UC for outcome Renal Failure

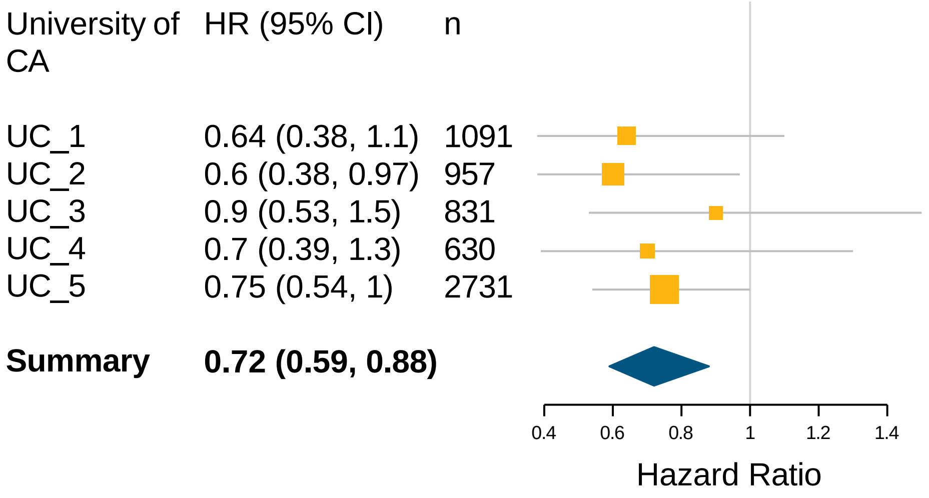

The table below shows the Leave-One-UC-Out diagnostics. The DFFITS value, Cook's distance, Covariance ratio, leave-one-out amount of heteroginity, indicator for influential estimates, comparator and treated groups are provided for each Leave-One-UC-Out analysis. The influential estimate from one UC with respect to pooled estimate are marked as Yes or No, with Yes indicating an influential UC and No otherwise.

eTable 169: Leave-One-UC-Out Sensitivity Analysis

DFFITs	Cook's Dist	Residual Heterogeneity	Influential	Comparator	Treated	UC
-0.1854799	0.0344028	0	No	Sulfonylurea	SGLT2i	UC_1
-0.3909443	0.1528374	0	No	Sulfonylurea	SGLT2i	UC_2
0.3832431	0.1468752	0	No	Sulfonylurea	SGLT2i	UC_3
-0.0307731	0.0009470	0	No	Sulfonylurea	SGLT2i	UC_4
0.3100990	0.0961614	0	No	Sulfonylurea	SGLT2i	UC_5

© 2023 Vashisht R et al. *JAMA Network Open.*

4.25 Stroke

4.25.1 eTable: Drug comparision table

Effect size of each drug comparison at each UC health site is tabulated.

eTable 170: Hazard ratios of drug class comparison at each UC

Comparator	Treated	UC	N	Hazard Ratio (95% CI)	P-value	Adjusted P-Value
DPP4i	GLP1ra	UC_1	682	0.12 (0.01-0.99)	4.88270e-02	3.316470e-01
DPP4i	GLP1ra	UC_2	1053	1 (0.33-3.2)	9.77734e-01	9.778260e-01
DPP4i	GLP1ra	UC_3	843	2.1 (0.55-8.2)	2.77821e-01	4.876300e-01
DPP4i	GLP1ra	UC_4	543	0.77 (0.21-2.9)	7.03390e-01	8.440680e-01
DPP4i	GLP1ra	UC_5	2648	0.71 (0.35-1.5)	3.48356e-01	5.196657e-01
DPP4i	SGLT2i	UC_1	1011	0.3 (0.1-0.92)	3.52775e-02	3.316470e-01
DPP4i	SGLT2i	UC_2	1037	1.9 (0.8-4.7)	1.40233e-01	3.505825e-01
DPP4i	SGLT2i	UC_3	673	0.53 (0.05-5.8)	6.02422e-01	7.662700e-01
DPP4i	SGLT2i	UC_4	543	0.54 (0.05-5.9)	6.13016e-01	7.662700e-01
DPP4i	SGLT2i	UC_5	2964	0.53 (0.24-1.2)	1.26331e-01	3.445391e-01
GLP1ra	SGLT2i	UC_1	688	5.5 (0.65-47)	1.18569e-01	3.445391e-01
GLP1ra	SGLT2i	UC_2	1251	3.2 (1-10)	4.72670e-02	3.316470e-01
GLP1ra	SGLT2i	UC_3	793	0.45 (0.12-1.7)	2.45243e-01	4.598306e-01
GLP1ra	SGLT2i	UC_4	641	1.1 (0.21-5.3)	9.40099e-01	9.778260e-01
GLP1ra	SGLT2i	UC_5	3068	0.53 (0.2-1.4)	1.99023e-01	4.264779e-01
Sulfonylurea	DPP4i	UC_1	1654	1.2 (0.69-1.9)	5.79877e-01	7.662700e-01
Sulfonylurea	DPP4i	UC_2	1969	0.73 (0.45-1.2)	2.20301e-01	4.406020e-01
Sulfonylurea	DPP4i	UC_3	2060	0.65 (0.38-1.1)	1.11056e-01	3.445391e-01
Sulfonylurea	DPP4i	UC_4	1225	0.91 (0.48-1.7)	7.68084e-01	8.862508e-01
Sulfonylurea	DPP4i	UC_5	5643	1 (0.71-1.4)	9.77826e-01	9.778260e-01
Sulfonylurea	GLP1ra	UC_1	723	0.17 (0.02-1.4)	9.61554e-02	3.445391e-01
Sulfonylurea	GLP1ra	UC_2	1120	0.45 (0.17-1.2)	1.03289e-01	3.445391e-01
Sulfonylurea	GLP1ra	UC_3	1058	0.41 (0.17-1)	5.22829e-02	3.316470e-01
Sulfonylurea	GLP1ra	UC_4	651	0.57 (0.17-1.9)	3.63766e-01	5.196657e-01
Sulfonylurea	GLP1ra	UC_5	2563	1.5 (0.65-3.6)	3.28093e-01	5.180416e-01
Sulfonylurea	SGLT2i	UC_1	1155	0.34 (0.11-1)	5.77723e-02	3.316470e-01
Sulfonylurea	SGLT2i	UC_2	1089	0.91 (0.44-1.9)	8.00212e-01	8.891244e-01
Sulfonylurea	SGLT2i	UC_3	919	0.41 (0.11-1.5)	1.86746e-01	4.264779e-01
Sulfonylurea	SGLT2i	UC_4	734	0.52 (0.16-1.7)	2.92578e-01	4.876300e-01
Sulfonylurea	SGLT2i	UC_5	2934	0.41 (0.16-1.1)	6.63294e-02	3.316470e-01

4.25.2 eFigure: Individual effect size, meta analysis and sensitivity analysis

The forest plot illustrate the effect size of the comparison between DPP4i and GLP1ra at each UC along with the effect size obtained from the random effect meta-analysis across all the UC for outcome Stroke

© 2023 Vashisht R et al. *JAMA Network Open.*

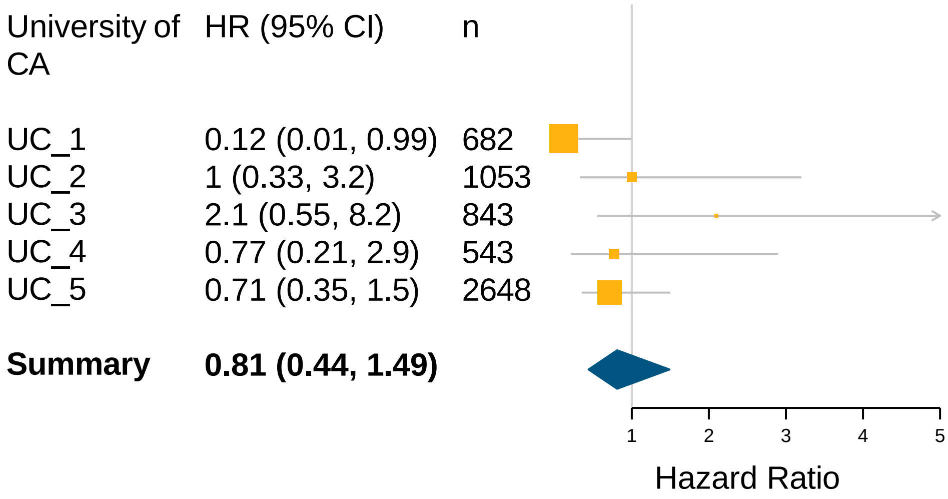

The table below shows the Leave-One-UC-Out diagnostics. The DFFITS value, Cook's distance, Covariance ratio, leave-one-out amount of heteroginity, indicator for influential estimates, comparator and treated groups are provided for each Leave-One-UC-Out analysis. The influential estimate from one UC with respect to pooled estimate are marked as Yes or No, with Yes indicating an influential UC and No otherwise.

eTable 171: Leave-One-UC-Out Sensitivity Analysis

DFFITs	Cook's Dist	Residual Heterogeneity	Influential	Comparator	Treated	UC
-0.3774578	0.1282800	0.0000000	No	DPP4i	GLP1ra	UC_1
0.2098127	0.0600724	0.2949620	No	DPP4i	GLP1ra	UC_2
0.5558067	0.2514987	0.0145918	No	DPP4i	GLP1ra	UC_3
0.0133355	0.0002282	0.2890930	No	DPP4i	GLP1ra	UC_4
-0.0649713	0.0080671	0.3671355	No	DPP4i	GLP1ra	UC_5

© 2023 Vashisht R et al. *JAMA Network Open.*

The forest plot illustrate the effect size of the comparison between DPP4i and SGLT2i at each UC along with the effect size obtained from the random effect meta-analysis across all the UC for outcome Stroke

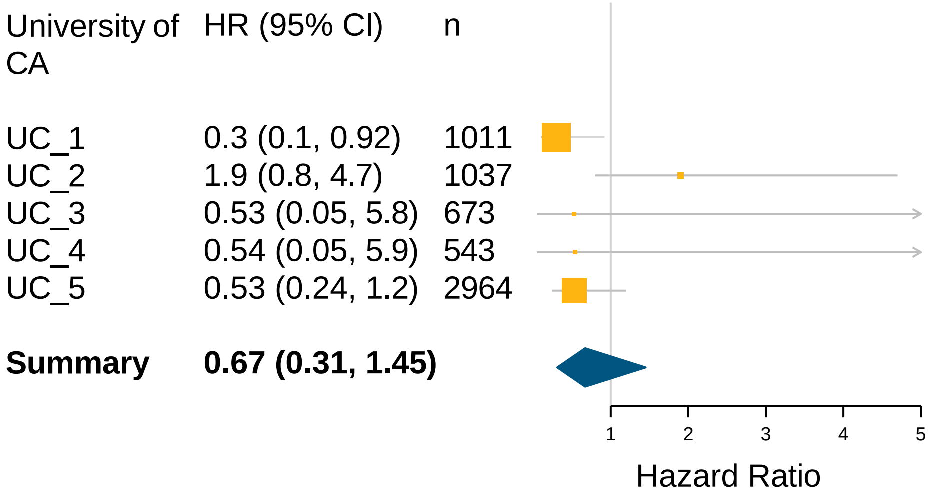

The table below shows the Leave-One-UC-Out diagnostics. The DFFITS value, Cook's distance, Covariance ratio, leave-one-out amount of heteroginity, indicator for influential estimates, comparator and treated groups are provided for each Leave-One-UC-Out analysis. The influential estimate from one UC with respect to pooled estimate are marked as Yes or No, with Yes indicating an influential UC and No otherwise.

eTable 172: Leave-One-UC-Out Sensitivity Analysis

DFFITs	Cook's Dist	Residual Heterogeneity	Influential	Comparator	Treated	UC
-0.7115844	0.4301928	0.2432244	No	DPP4i	SGLT2i	UC_1
1.6653200	1.0343920	0.0000000	Yes	DPP4i	SGLT2i	UC_2
-0.0305908	0.0009992	0.4675649	No	DPP4i	SGLT2i	UC_3
-0.0257138	0.0007063	0.4683671	No	DPP4i	SGLT2i	UC_4
-0.1115550	0.0206190	0.6790836	No	DPP4i	SGLT2i	UC_5

© 2023 Vashisht R et al. *JAMA Network Open.*

The forest plot illustrate the effect size of the comparison between GLP1ra and SGLT2i at each UC along with the effect size obtained from the random effect meta-analysis across all the UC for outcome Stroke

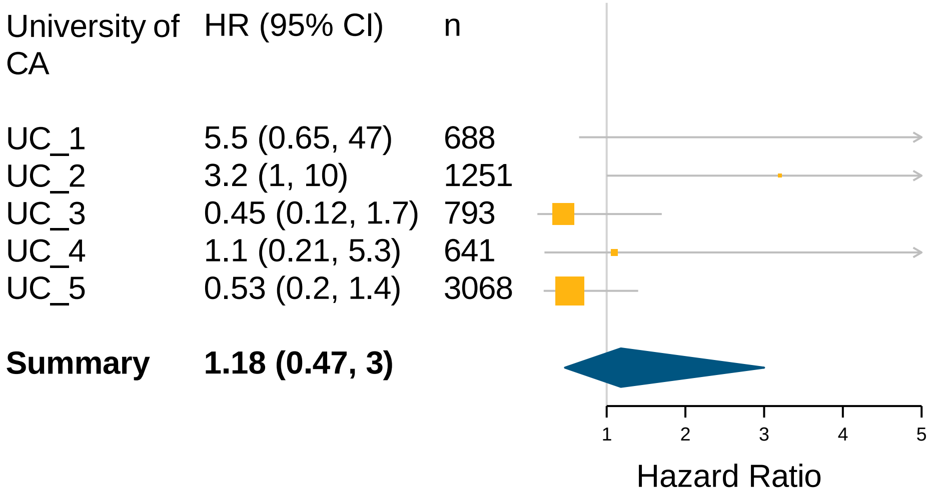

The table below shows the Leave-One-UC-Out diagnostics. The DFFITS value, Cook's distance, Covariance ratio, leave-one-out amount of heteroginity, indicator for influential estimates, comparator and treated groups are provided for each Leave-One-UC-Out analysis. The influential estimate from one UC with respect to pooled estimate are marked as Yes or No, with Yes indicating an influential UC and No otherwise.

eTable 173: Leave-One-UC-Out Sensitivity Analysis

DFFITs	Cook's Dist	Residual Heterogeneity	Influential	Comparator	Treated	UC
0.4764983	0.2131916	0.5121250	No	GLP1ra	SGLT2i	UC_1
0.9862512	0.6259646	0.2778971	Yes	GLP1ra	SGLT2i	UC_2
-0.5368613	0.3092222	0.7016677	No	GLP1ra	SGLT2i	UC_3
-0.0821525	0.0082744	0.9170426	No	GLP1ra	SGLT2i	UC_4
-0.5867883	0.3541239	0.6477020	No	GLP1ra	SGLT2i	UC_5

© 2023 Vashisht R et al. *JAMA Network Open.*

The forest plot illustrate the effect size of the comparison between Sulfonylurea and DPP4i at each UC along with the effect size obtained from the random effect meta-analysis across all the UC for outcome Stroke

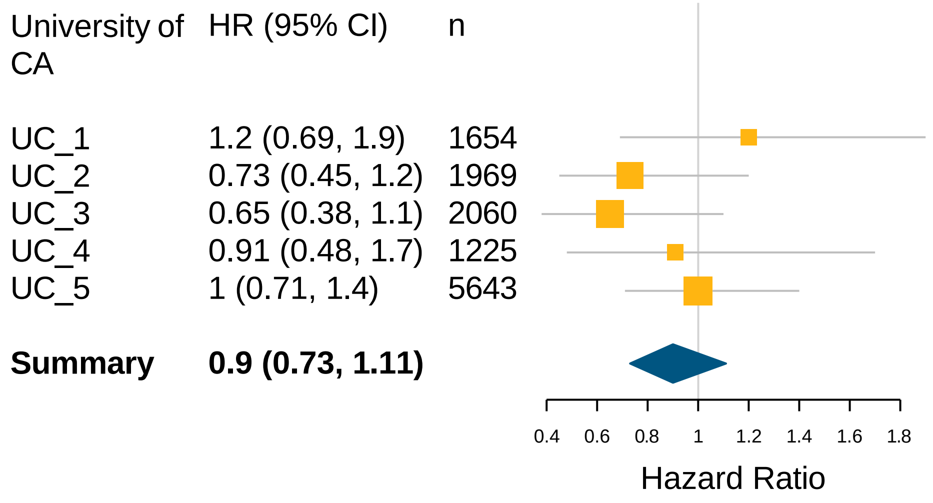

The table below shows the Leave-One-UC-Out diagnostics. The DFFITS value, Cook's distance, Covariance ratio, leave-one-out amount of heteroginity, indicator for influential estimates, comparator and treated groups are provided for each Leave-One-UC-Out analysis. The influential estimate from one UC with respect to pooled estimate are marked as Yes or No, with Yes indicating an influential UC and No otherwise.

eTable 174: Leave-One-UC-Out Sensitivity Analysis

DFFITs	Cook's Dist	Residual Heterogeneity	Influential	Comparator	Treated	UC
0.5522915	0.3050259	0.0000000	No	Sulfonylurea	DPP4i	UC_1
-0.4410660	0.1945392	0.0000000	No	Sulfonylurea	DPP4i	UC_2
-0.5633830	0.3174004	0.0000000	No	Sulfonylurea	DPP4i	UC_3
0.0871301	0.0085846	0.0136122	No	Sulfonylurea	DPP4i	UC_4
0.5567410	0.3544768	0.0043087	No	Sulfonylurea	DPP4i	UC_5

© 2023 Vashisht R et al. *JAMA Network Open.*

The forest plot illustrate the effect size of the comparison between Sulfonylurea and GLP1ra at each UC along with the effect size obtained from the random effect meta-analysis across all the UC for outcome Stroke

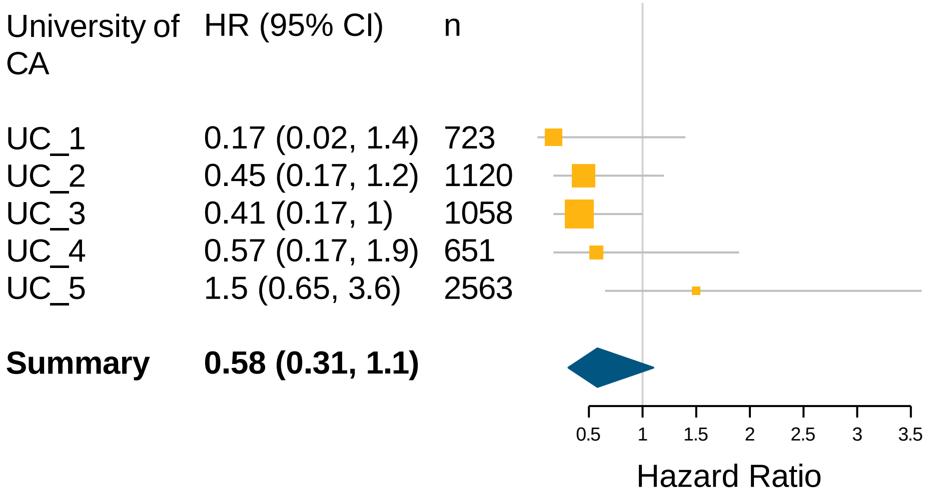

The table below shows the Leave-One-UC-Out diagnostics. The DFFITS value, Cook's distance, Covariance ratio, leave-one-out amount of heteroginity, indicator for influential estimates, comparator and treated groups are provided for each Leave-One-UC-Out analysis. The influential estimate from one UC with respect to pooled estimate are marked as Yes or No, with Yes indicating an influential UC and No otherwise.

eTable 175: Leave-One-UC-Out Sensitivity Analysis

DFFITs	Cook's Dist	Residual Heterogeneity	Influential	Comparator	Treated	UC
-0.3196477	0.1004786	0.1853542	No	Sulfonylurea	GLP1ra	UC_1
-0.1243867	0.0202800	0.3502795	No	Sulfonylurea	GLP1ra	UC_2
-0.2650419	0.0860875	0.3013606	No	Sulfonylurea	GLP1ra	UC_3
0.0693315	0.0060485	0.3600635	No	Sulfonylurea	GLP1ra	UC_4
1.4041747	0.9422927	0.0000000	Yes	Sulfonylurea	GLP1ra	UC_5

The forest plot illustrate the effect size of the comparison between Sulfonylurea and SGLT2i at each UC along with the effect size obtained from the random effect meta-analysis across all the UC for outcome Stroke

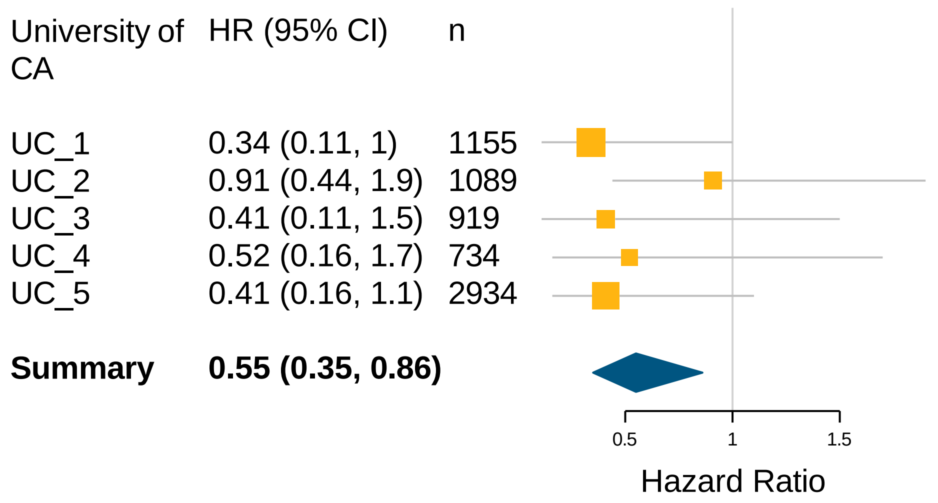

The table below shows the Leave-One-UC-Out diagnostics. The DFFITS value, Cook's distance, Covariance ratio, leave-one-out amount of heteroginity, indicator for influential estimates, comparator and treated groups are provided for each Leave-One-UC-Out analysis. The influential estimate from one UC with respect to pooled estimate are marked as Yes or No, with Yes indicating an influential UC and No otherwise.

eTable 176: Leave-One-UC-Out Sensitivity Analysis

DFFITs	Cook's Dist	Residual Heterogeneity	Influential	Comparator	Treated	UC
-0.4132260	0.1707558	0.0000000	No	Sulfonylurea	SGLT2i	UC_1
1.2890297	1.6615975	0.0000000	Yes	Sulfonylurea	SGLT2i	UC_2
-0.1714757	0.0294039	0.0000000	No	Sulfonylurea	SGLT2i	UC_3
-0.0144329	0.0002131	0.0083832	No	Sulfonylurea	SGLT2i	UC_4
-0.3536139	0.1250428	0.0000000	No	Sulfonylurea	SGLT2i	UC_5

© 2023 Vashisht R et al. *JAMA Network Open.*

4.26 Thrombocytopenia

4.26.1 eTable: Drug comparision table

Effect size of each drug comparison at each UC health site is tabulated.

eTable 177: Hazard ratios of drug class comparison at each UC

Comparator	Treated	UC	N	Hazard Ratio (95% CI)	P-value	Adjusted P-Value
DPP4i	GLP1ra	UC_1	687	1 (0.06-16)	9.98235e-01	9.982350e-01
DPP4i	GLP1ra	UC_2	1054	0.75 (0.3-1.9)	5.44411e-01	8.595963e-01
DPP4i	GLP1ra	UC_3	861	0.95 (0.36-2.6)	9.23789e-01	9.556438e-01
DPP4i	GLP1ra	UC_4	539	0.45 (0.14-1.5)	1.87879e-01	4.696975e-01
DPP4i	GLP1ra	UC_5	2664	0.49 (0.25-0.96)	3.64620e-02	4.696975e-01
DPP4i	SGLT2i	UC_1	1027	4.2 (0.88-20)	7.15607e-02	4.696975e-01
DPP4i	SGLT2i	UC_2	1036	1.1 (0.51-2.4)	7.95133e-01	8.834811e-01
DPP4i	SGLT2i	UC_3	693	0.28 (0.06-1.4)	1.16082e-01	4.696975e-01
DPP4i	SGLT2i	UC_4	547	0.42 (0.11-1.6)	2.13847e-01	4.934931e-01
DPP4i	SGLT2i	UC_5	2946	0.8 (0.45-1.4)	4.49652e-01	8.227000e-01
GLP1ra	SGLT2i	UC_1	701	6.2 (0.75-52)	9.05348e-02	4.696975e-01
GLP1ra	SGLT2i	UC_2	1272	1.3 (0.53-3.1)	5.88830e-01	8.616729e-01
GLP1ra	SGLT2i	UC_3	811	0.41 (0.08-2.1)	2.91494e-01	5.829880e-01
GLP1ra	SGLT2i	UC_4	645	1.5 (0.25-8.9)	6.63288e-01	8.651583e-01
GLP1ra	SGLT2i	UC_5	3061	1.6 (0.82-3)	1.74823e-01	4.696975e-01
Sulfonylurea	DPP4i	UC_1	1701	0.57 (0.31-1)	7.12945e-02	4.696975e-01
Sulfonylurea	DPP4i	UC_2	2000	1.7 (1-2.9)	3.48327e-02	4.696975e-01
Sulfonylurea	DPP4i	UC_3	2089	1.2 (0.7-2.1)	4.79819e-01	8.227000e-01
Sulfonylurea	DPP4i	UC_4	1238	0.91 (0.51-1.6)	7.48312e-01	8.834811e-01
Sulfonylurea	DPP4i	UC_5	5678	0.95 (0.69-1.3)	7.66049e-01	8.834811e-01
Sulfonylurea	GLP1ra	UC_1	740	0.22 (0.03-2)	1.78507e-01	4.696975e-01
Sulfonylurea	GLP1ra	UC_2	1129	0.86 (0.37-2)	7.29812e-01	8.834811e-01
Sulfonylurea	GLP1ra	UC_3	1072	1.4 (0.51-4)	4.93620e-01	8.227000e-01
Sulfonylurea	GLP1ra	UC_4	634	0.43 (0.13-1.4)	1.63244e-01	4.696975e-01
Sulfonylurea	GLP1ra	UC_5	2573	0.6 (0.28-1.3)	1.74708e-01	4.696975e-01
Sulfonylurea	SGLT2i	UC_1	1188	1.1 (0.38-3.1)	8.81373e-01	9.443282e-01
Sulfonylurea	SGLT2i	UC_2	1099	0.83 (0.36-1.9)	6.48526e-01	8.651583e-01
Sulfonylurea	SGLT2i	UC_3	946	1.4 (0.4-4.9)	6.03171e-01	8.616729e-01
Sulfonylurea	SGLT2i	UC_4	743	0.55 (0.19-1.6)	2.87625e-01	5.829880e-01
Sulfonylurea	SGLT2i	UC_5	2924	0.63 (0.36-1.1)	1.16369e-01	4.696975e-01

4.26.2 eFigure: Individual effect size, meta analysis and sensitivity analysis

The forest plot illustrate the effect size of the comparison between DPP4i and GLP1ra at each UC along with the effect size obtained from the random effect meta-analysis across all the UC for outcome Thrombocytopenia

© 2023 Vashisht R et al. *JAMA Network Open.*

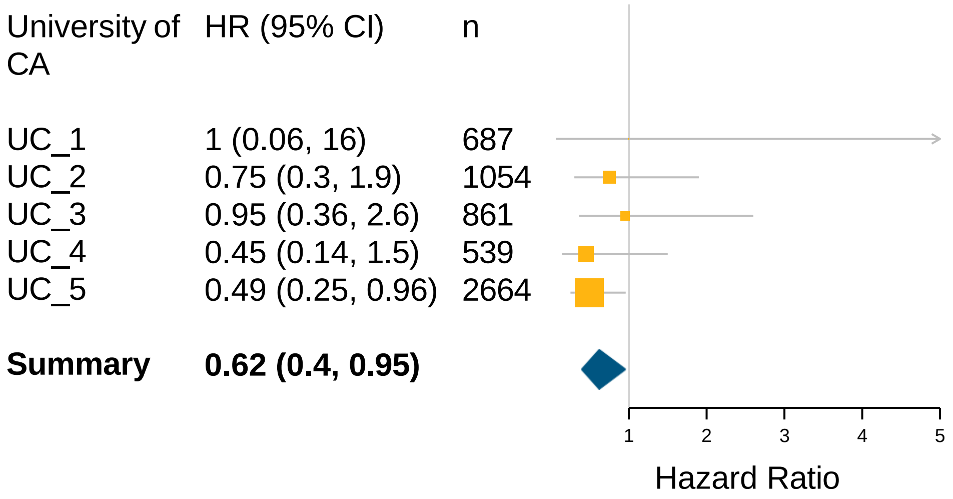

The table below shows the Leave-One-UC-Out diagnostics. The DFFITS value, Cook's distance, Covariance ratio, leave-one-out amount of heteroginity, indicator for influential estimates, comparator and treated groups are provided for each Leave-One-UC-Out analysis. The influential estimate from one UC with respect to pooled estimate are marked as Yes or No, with Yes indicating an influential UC and No otherwise.

eTable 178: Leave-One-UC-Out Sensitivity Analysis

DFFITs	Cook's Dist	Residual Heterogeneity	Influential	Comparator	Treated	UC
0.0549532	0.0030199	0	No	DPP4i	GLP1ra	UC_1
0.2526521	0.0638331	0	No	DPP4i	GLP1ra	UC_2
0.4694165	0.2203519	0	No	DPP4i	GLP1ra	UC_3
-0.2221215	0.0493380	0	No	DPP4i	GLP1ra	UC_4
-0.7523875	0.5660869	0	Yes	DPP4i	GLP1ra	UC_5

© 2023 Vashisht R et al. *JAMA Network Open.*

The forest plot illustrate the effect size of the comparison between DPP4i and SGLT2i at each UC along with the effect size obtained from the random effect meta-analysis across all the UC for outcome Thrombocytopenia

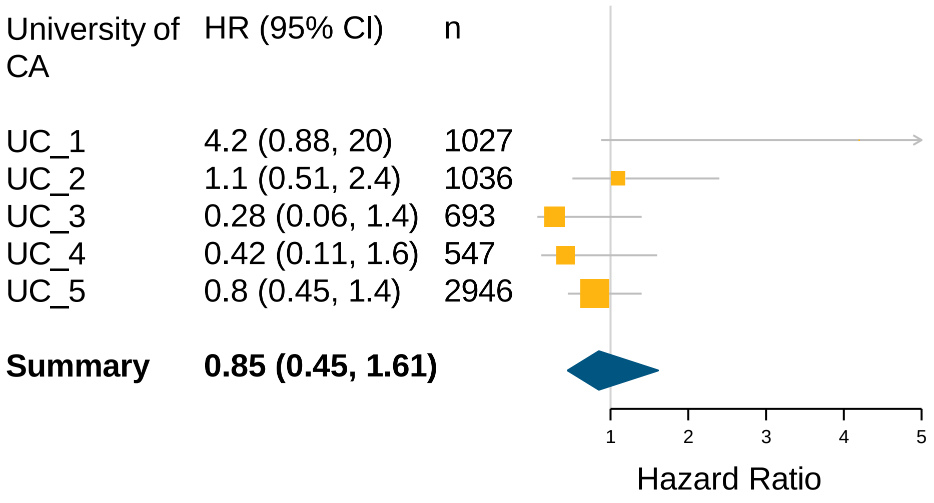

The table below shows the Leave-One-UC-Out diagnostics. The DFFITS value, Cook's distance, Covariance ratio, leave-one-out amount of heteroginity, indicator for influential estimates, comparator and treated groups are provided for each Leave-One-UC-Out analysis. The influential estimate from one UC with respect to pooled estimate are marked as Yes or No, with Yes indicating an influential UC and No otherwise.

eTable 179: Leave-One-UC-Out Sensitivity Analysis

DFFITs	Cook's Dist	Residual Heterogeneity	Influential	Comparator	Treated	UC
0.4259942	0.1372182	0.0140251	No	DPP4i	SGLT2i	UC_1
0.2240723	0.0824909	0.4672880	No	DPP4i	SGLT2i	UC_2
-0.4513846	0.1931494	0.1777568	No	DPP4i	SGLT2i	UC_3
-0.3901302	0.1593179	0.2555739	No	DPP4i	SGLT2i	UC_4
-0.0359930	0.0028413	0.5898196	No	DPP4i	SGLT2i	UC_5

© 2023 Vashisht R et al. *JAMA Network Open.*

The forest plot illustrate the effect size of the comparison between GLP1ra and SGLT2i at each UC along with the effect size obtained from the random effect meta-analysis across all the UC for outcome Thrombocytopenia

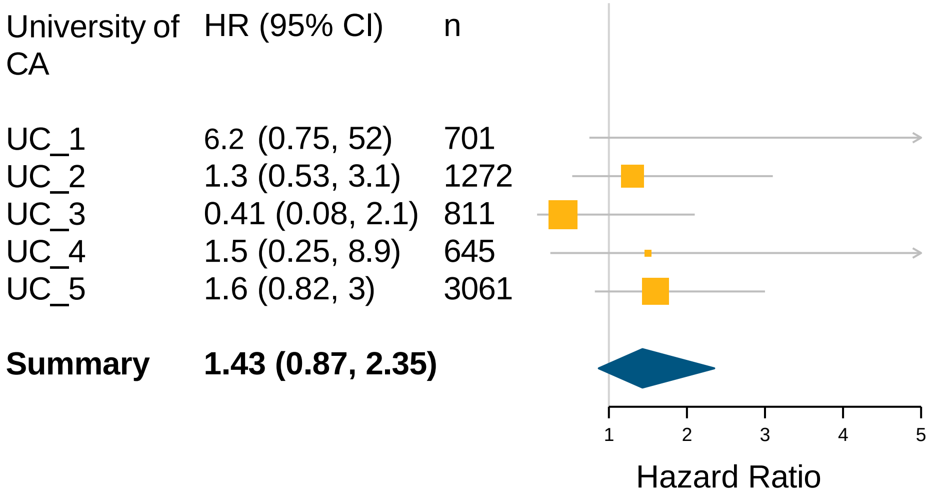

The table below shows the Leave-One-UC-Out diagnostics. The DFFITS value, Cook's distance, Covariance ratio, leave-one-out amount of heteroginity, indicator for influential estimates, comparator and treated groups are provided for each Leave-One-UC-Out analysis. The influential estimate from one UC with respect to pooled estimate are marked as Yes or No, with Yes indicating an influential UC and No otherwise.

eTable 180: Leave-One-UC-Out Sensitivity Analysis

DFFITs	Cook's Dist	Residual Heterogeneity	Influential	Comparator	Treated	UC
0.2746164	0.0740081	0.0000000	No	GLP1ra	SGLT2i	UC_1
-0.0445517	0.0036175	0.2074948	No	GLP1ra	SGLT2i	UC_2
-0.4692485	0.2133721	0.0000000	No	GLP1ra	SGLT2i	UC_3
0.0758192	0.0064740	0.1298275	No	GLP1ra	SGLT2i	UC_4
0.2427542	0.1400157	0.2034417	No	GLP1ra	SGLT2i	UC_5

© 2023 Vashisht R et al. *JAMA Network Open.*

The forest plot illustrate the effect size of the comparison between Sulfonylurea and DPP4i at each UC along with the effect size obtained from the random effect meta-analysis across all the UC for outcome Thrombocytopenia

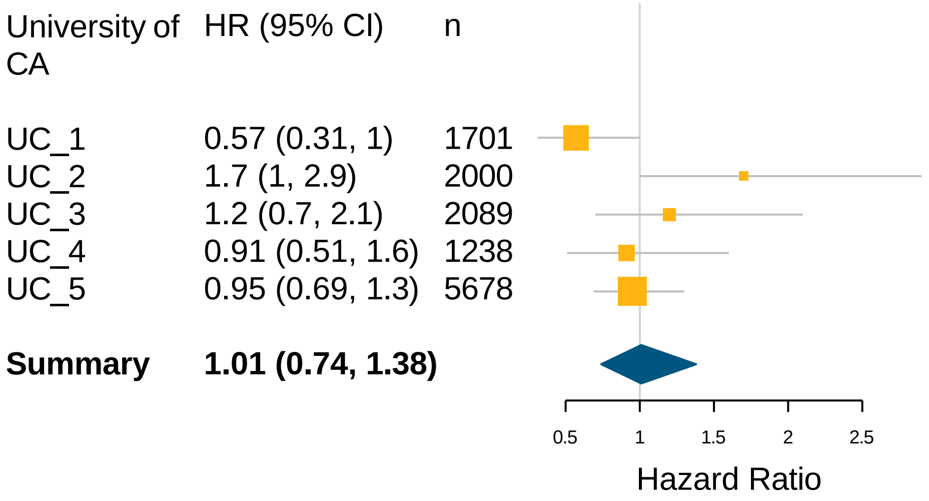

The table below shows the Leave-One-UC-Out diagnostics. The DFFITS value, Cook's distance, Covariance ratio, leave-one-out amount of heteroginity, indicator for influential estimates, comparator and treated groups are provided for each Leave-One-UC-Out analysis. The influential estimate from one UC with respect to pooled estimate are marked as Yes or No, with Yes indicating an influential UC and No otherwise.

eTable 181: Leave-One-UC-Out Sensitivity Analysis

DFFITs	Cook's Dist	Residual Heterogeneity	Influential	Comparator	Treated	UC
-0.7246063	0.3704220	0.0177545	No	Sulfonylurea	DPP4i	UC_1
0.8587807	0.4550141	0.0102592	Yes	Sulfonylurea	DPP4i	UC_2
0.2308301	0.0642257	0.0913849	No	Sulfonylurea	DPP4i	UC_3
-0.1218077	0.0182919	0.0967773	No	Sulfonylurea	DPP4i	UC_4
-0.0866348	0.0132361	0.1300326	No	Sulfonylurea	DPP4i	UC_5

© 2023 Vashisht R et al. *JAMA Network Open.*

The forest plot illustrate the effect size of the comparison between Sulfonylurea and GLP1ra at each UC along with the effect size obtained from the random effect meta-analysis across all the UC for outcome Thrombocytopenia

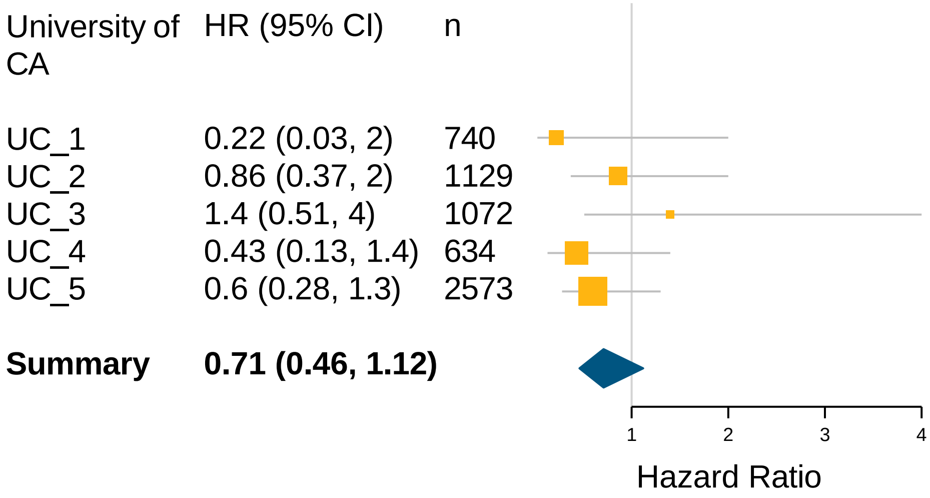

The table below shows the Leave-One-UC-Out diagnostics. The DFFITS value, Cook's distance, Covariance ratio, leave-one-out amount of heteroginity, indicator for influential estimates, comparator and treated groups are provided for each Leave-One-UC-Out analysis. The influential estimate from one UC with respect to pooled estimate are marked as Yes or No, with Yes indicating an influential UC and No otherwise.

eTable 182: Leave-One-UC-Out Sensitivity Analysis

DFFITs	Cook's Dist	Residual Heterogeneity	Influential	Comparator	Treated	UC
-0.2251279	0.0506826	0.0000000	No	Sulfonylurea	GLP1ra	UC_1
0.3134420	0.1319627	0.0635933	No	Sulfonylurea	GLP1ra	UC_2
0.6904665	0.4767440	0.0000000	Yes	Sulfonylurea	GLP1ra	UC_3
-0.3676817	0.1358845	0.0018889	No	Sulfonylurea	GLP1ra	UC_4
-0.2437572	0.0838179	0.0629982	No	Sulfonylurea	GLP1ra	UC_5

© 2023 Vashisht R et al. *JAMA Network Open.*

The forest plot illustrate the effect size of the comparison between Sulfonylurea and SGLT2i at each UC along with the effect size obtained from the random effect meta-analysis across all the UC for outcome Thrombocytopenia

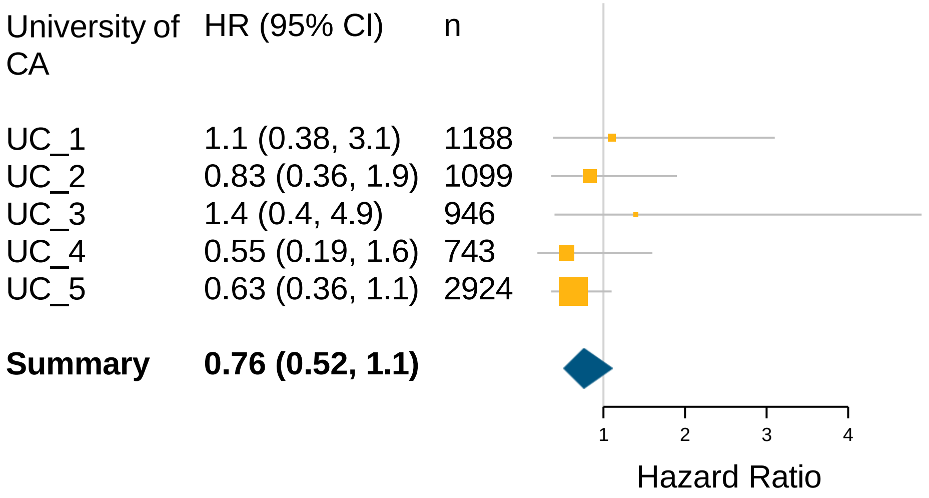

The table below shows the Leave-One-UC-Out diagnostics. The DFFITS value, Cook's distance, Covariance ratio, leave-one-out amount of heteroginity, indicator for influential estimates, comparator and treated groups are provided for each Leave-One-UC-Out analysis. The influential estimate from one UC with respect to pooled estimate are marked as Yes or No, with Yes indicating an influential UC and No otherwise.

eTable 183: Leave-One-UC-Out Sensitivity Analysis

DFFITs	Cook's Dist	Residual Heterogeneity	Influential	Comparator	Treated	UC
0.2874003	0.0825989	0	No	Sulfonylurea	SGLT2i	UC_1
0.1243511	0.0154632	0	No	Sulfonylurea	SGLT2i	UC_2
0.3176290	0.1008882	0	No	Sulfonylurea	SGLT2i	UC_3
-0.2361889	0.0557852	0	No	Sulfonylurea	SGLT2i	UC_4
-0.7892625	0.6229352	0	Yes	Sulfonylurea	SGLT2i	UC_5

© 2023 Vashisht R et al. *JAMA Network Open.*

4.27 Urinary Tract Infectious

4.27.1 eTable: Drug comparision table

Effect size of each drug comparison at each UC health site is tabulated.

eTable 184: Hazard ratios of drug class comparison at each UC

Comparator	Treated	UC	N	Hazard Ratio (95% CI)	P-value	Adjusted P-Value
DPP4i	GLP1ra	UC_1	618	1.1 (0.54-2.1)	8.34374e-01	9.287721e-01
DPP4i	GLP1ra	UC_2	908	0.73 (0.44-1.2)	2.19344e-01	6.094007e-01
DPP4i	GLP1ra	UC_3	752	1.5 (0.86-2.6)	1.56802e-01	6.094007e-01
DPP4i	GLP1ra	UC_4	507	0.86 (0.43-1.7)	6.70708e-01	9.287721e-01
DPP4i	GLP1ra	UC_5	2380	0.83 (0.6-1.2)	2.61052e-01	6.094007e-01
DPP4i	SGLT2i	UC_1	933	0.64 (0.35-1.2)	1.48851e-01	6.094007e-01
DPP4i	SGLT2i	UC_2	924	0.88 (0.54-1.5)	6.30035e-01	9.287721e-01
DPP4i	SGLT2i	UC_3	604	0.87 (0.4-1.9)	7.21859e-01	9.287721e-01
DPP4i	SGLT2i	UC_4	521	1.1 (0.44-2.7)	8.66854e-01	9.287721e-01
DPP4i	SGLT2i	UC_5	2736	0.78 (0.55-1.1)	1.51400e-01	6.094007e-01
GLP1ra	SGLT2i	UC_1	635	1.1 (0.52-2.5)	7.50975e-01	9.287721e-01
GLP1ra	SGLT2i	UC_2	1076	1 (0.59-1.7)	9.92320e-01	9.923200e-01
GLP1ra	SGLT2i	UC_3	706	0.69 (0.35-1.4)	2.84387e-01	6.094007e-01
GLP1ra	SGLT2i	UC_4	599	0.59 (0.26-1.4)	2.16778e-01	6.094007e-01
GLP1ra	SGLT2i	UC_5	2780	0.98 (0.69-1.4)	9.26893e-01	9.588548e-01
Sulfonylurea	DPP4i	UC_1	1553	0.79 (0.56-1.1)	1.75831e-01	6.094007e-01
Sulfonylurea	DPP4i	UC_2	1787	1.2 (0.89-1.5)	2.76250e-01	6.094007e-01
Sulfonylurea	DPP4i	UC_3	1856	0.97 (0.72-1.3)	8.35910e-01	9.287721e-01
Sulfonylurea	DPP4i	UC_4	1189	0.89 (0.56-1.4)	6.06020e-01	9.287721e-01
Sulfonylurea	DPP4i	UC_5	5312	0.97 (0.8-1.2)	7.27899e-01	9.287721e-01
Sulfonylurea	GLP1ra	UC_1	655	0.68 (0.35-1.3)	2.35273e-01	6.094007e-01
Sulfonylurea	GLP1ra	UC_2	984	0.9 (0.57-1.4)	6.60971e-01	9.287721e-01
Sulfonylurea	GLP1ra	UC_3	949	1.6 (0.94-2.6)	8.40574e-02	6.094007e-01
Sulfonylurea	GLP1ra	UC_4	605	0.85 (0.43-1.7)	6.44656e-01	9.287721e-01
Sulfonylurea	GLP1ra	UC_5	2359	0.87 (0.61-1.2)	4.11600e-01	8.232000e-01
Sulfonylurea	SGLT2i	UC_1	1098	0.55 (0.32-0.97)	3.77380e-02	6.094007e-01
Sulfonylurea	SGLT2i	UC_2	988	1.1 (0.62-1.8)	8.26632e-01	9.287721e-01
Sulfonylurea	SGLT2i	UC_3	849	1.1 (0.58-2.1)	7.69192e-01	9.287721e-01
Sulfonylurea	SGLT2i	UC_4	718	0.61 (0.29-1.3)	1.97947e-01	6.094007e-01
Sulfonylurea	SGLT2i	UC_5	2769	0.78 (0.55-1.1)	1.72645e-01	6.094007e-01

4.27.2 eFigure: Individual effect size, meta analysis and sensitivity analysis

The forest plot illustrate the effect size of the comparison between DPP4i and GLP1ra at each UC along with the effect size obtained from the random effect meta-analysis across all the UC for outcome Urinary Tract Infectious

© 2023 Vashisht R et al. *JAMA Network Open.*

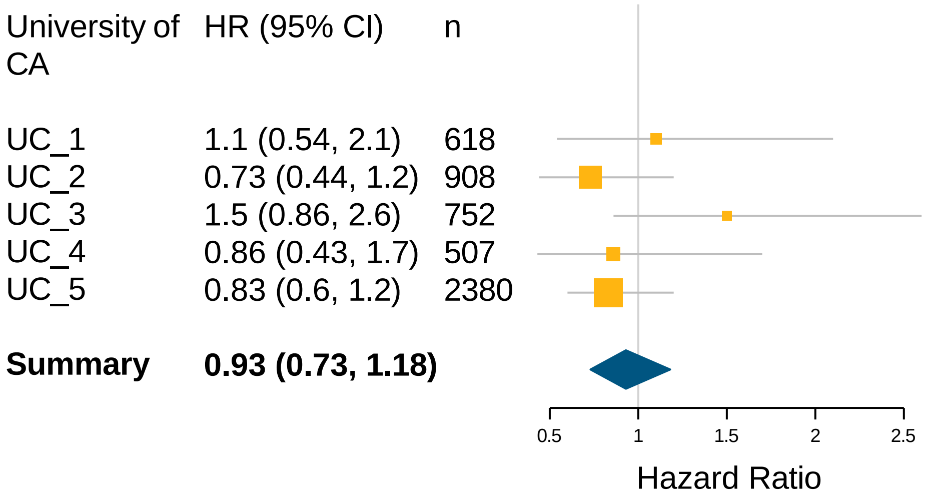

The table below shows the Leave-One-UC-Out diagnostics. The DFFITS value, Cook's distance, Covariance ratio, leave-one-out amount of heteroginity, indicator for influential estimates, comparator and treated groups are provided for each Leave-One-UC-Out analysis. The influential estimate from one UC with respect to pooled estimate are marked as Yes or No, with Yes indicating an influential UC and No otherwise.

eTable 185: Leave-One-UC-Out Sensitivity Analysis

DFFITs	Cook's Dist	Residual Heterogeneity	Influential	Comparator	Treated	UC
0.1083271	0.0133064	0.0253594	No	DPP4i	GLP1ra	UC_1
-0.5223261	0.2807539	0.0103278	No	DPP4i	GLP1ra	UC_2
0.8763738	0.6964581	0.0000000	Yes	DPP4i	GLP1ra	UC_3
-0.1947083	0.0443505	0.0304624	No	DPP4i	GLP1ra	UC_4
-0.4981778	0.3589909	0.0258015	No	DPP4i	GLP1ra	UC_5

© 2023 Vashisht R et al. *JAMA Network Open.*

The forest plot illustrate the effect size of the comparison between DPP4i and SGLT2i at each UC along with the effect size obtained from the random effect meta-analysis across all the UC for outcome Urinary Tract Infectious

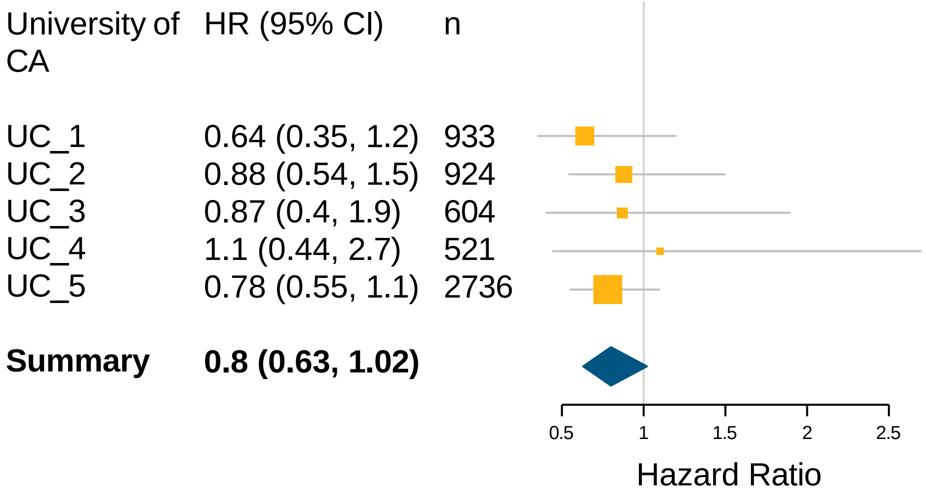

The table below shows the Leave-One-UC-Out diagnostics. The DFFITS value, Cook's distance, Covariance ratio, leave-one-out amount of heteroginity, indicator for influential estimates, comparator and treated groups are provided for each Leave-One-UC-Out analysis. The influential estimate from one UC with respect to pooled estimate are marked as Yes or No, with Yes indicating an influential UC and No otherwise.

eTable 186: Leave-One-UC-Out Sensitivity Analysis

DFFITs	Cook's Dist	Residual Heterogeneity	Influential	Comparator	Treated	UC
-0.3298523	0.1088025	0	No	DPP4i	SGLT2i	UC_1
0.2057427	0.0423301	0	No	DPP4i	SGLT2i	UC_2
0.0666944	0.0044481	0	No	DPP4i	SGLT2i	UC_3
0.1907030	0.0363676	0	No	DPP4i	SGLT2i	UC_4
-0.2242496	0.0502879	0	No	DPP4i	SGLT2i	UC_5

© 2023 Vashisht R et al. *JAMA Network Open.*

The forest plot illustrate the effect size of the comparison between GLP1ra and SGLT2i at each UC along with the effect size obtained from the random effect meta-analysis across all the UC for outcome Urinary Tract Infectious

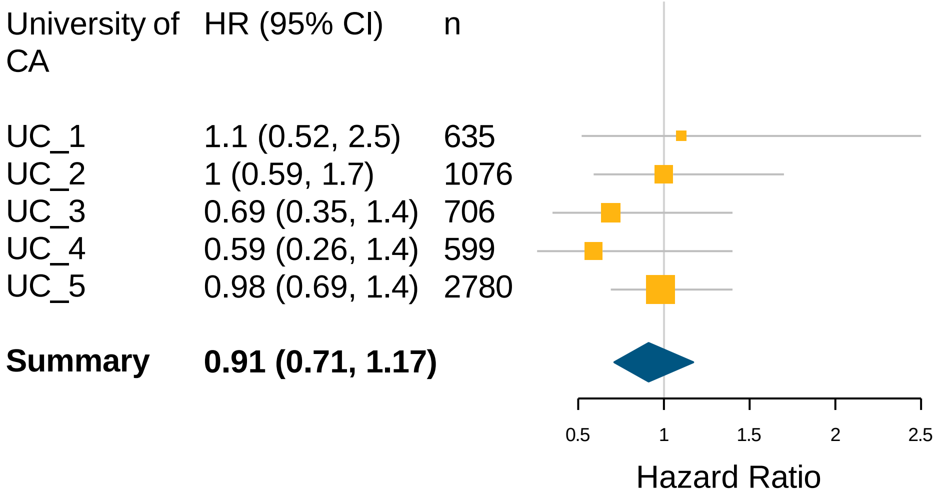

The table below shows the Leave-One-UC-Out diagnostics. The DFFITS value, Cook's distance, Covariance ratio, leave-one-out amount of heteroginity, indicator for influential estimates, comparator and treated groups are provided for each Leave-One-UC-Out analysis. The influential estimate from one UC with respect to pooled estimate are marked as Yes or No, with Yes indicating an influential UC and No otherwise.

eTable 187: Leave-One-UC-Out Sensitivity Analysis

DFFITs	Cook's Dist	Residual Heterogeneity	Influential	Comparator	Treated	UC
0.1610172	0.0259265	0	No	GLP1ra	SGLT2i	UC_1
0.1993116	0.0397251	0	No	GLP1ra	SGLT2i	UC_2
-0.3192408	0.1019147	0	No	GLP1ra	SGLT2i	UC_3
-0.3228118	0.1042075	0	No	GLP1ra	SGLT2i	UC_4
0.5239490	0.2745226	0	No	GLP1ra	SGLT2i	UC_5

© 2023 Vashisht R et al. *JAMA Network Open.*

The forest plot illustrate the effect size of the comparison between Sulfonylurea and DPP4i at each UC along with the effect size obtained from the random effect meta-analysis across all the UC for outcome Urinary Tract Infectious

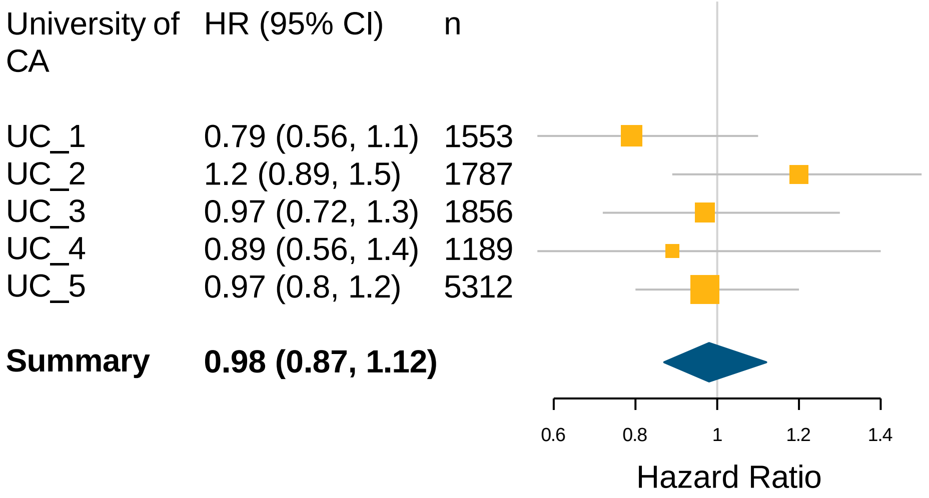

The table below shows the Leave-One-UC-Out diagnostics. The DFFITS value, Cook's distance, Covariance ratio, leave-one-out amount of heteroginity, indicator for influential estimates, comparator and treated groups are provided for each Leave-One-UC-Out analysis. The influential estimate from one UC with respect to pooled estimate are marked as Yes or No, with Yes indicating an influential UC and No otherwise.

eTable 188: Leave-One-UC-Out Sensitivity Analysis

DFFITs	Cook's Dist	Residual Heterogeneity	Influential	Comparator	Treated	UC
-0.5484650	0.2975772	0.0000000	No	Sulfonylurea	DPP4i	UC_1
0.9215846	0.8341419	0.0000000	Yes	Sulfonylurea	DPP4i	UC_2
0.0416951	0.0022976	0.0077337	No	Sulfonylurea	DPP4i	UC_3
-0.0859231	0.0080539	0.0053191	No	Sulfonylurea	DPP4i	UC_4
0.0301737	0.0016620	0.0094202	No	Sulfonylurea	DPP4i	UC_5

© 2023 Vashisht R et al. *JAMA Network Open.*

The forest plot illustrate the effect size of the comparison between Sulfonylurea and GLP1ra at each UC along with the effect size obtained from the random effect meta-analysis across all the UC for outcome Urinary Tract Infectious

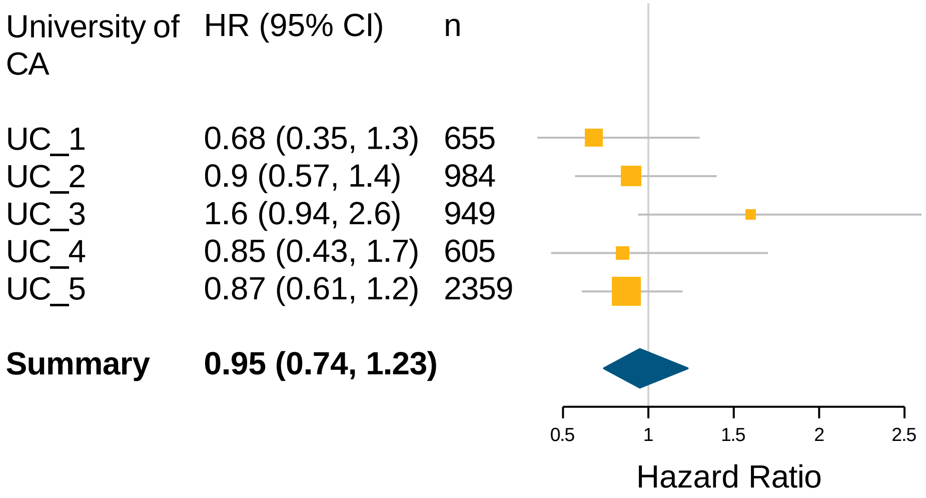

The table below shows the Leave-One-UC-Out diagnostics. The DFFITS value, Cook's distance, Covariance ratio, leave-one-out amount of heteroginity, indicator for influential estimates, comparator and treated groups are provided for each Leave-One-UC-Out analysis. The influential estimate from one UC with respect to pooled estimate are marked as Yes or No, with Yes indicating an influential UC and No otherwise.

eTable 189: Leave-One-UC-Out Sensitivity Analysis

DFFITs	Cook's Dist	Residual Heterogeneity	Influential	Comparator	Treated	UC
-0.3812624	0.1481881	0.0257763	No	Sulfonylurea	GLP1ra	UC_1
-0.1028206	0.0151302	0.0557832	No	Sulfonylurea	GLP1ra	UC_2
1.0150618	0.7668482	0.0000000	Yes	Sulfonylurea	GLP1ra	UC_3
-0.1233638	0.0174105	0.0441910	No	Sulfonylurea	GLP1ra	UC_4
-0.2160928	0.0752037	0.0554669	No	Sulfonylurea	GLP1ra	UC_5

© 2023 Vashisht R et al. *JAMA Network Open.*

The forest plot illustrate the effect size of the comparison between Sulfonylurea and SGLT2i at each UC along with the effect size obtained from the random effect meta-analysis across all the UC for outcome Urinary Tract Infectious

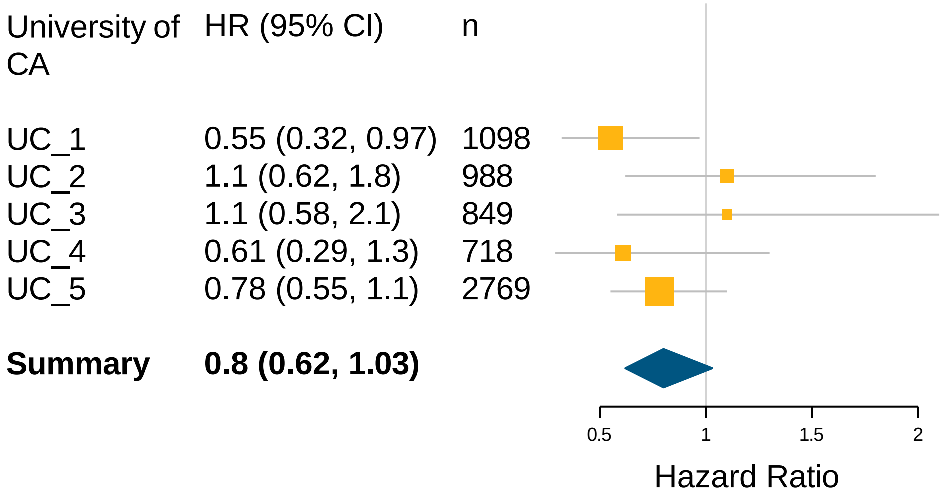

The table below shows the Leave-One-UC-Out diagnostics. The DFFITS value, Cook's distance, Covariance ratio, leave-one-out amount of heteroginity, indicator for influential estimates, comparator and treated groups are provided for each Leave-One-UC-Out analysis. The influential estimate from one UC with respect to pooled estimate are marked as Yes or No, with Yes indicating an influential UC and No otherwise.

eTable 190: Leave-One-UC-Out Sensitivity Analysis

DFFITs	Cook's Dist	Residual Heterogeneity	Influential	Comparator	Treated	UC
-0.6239731	0.3425647	0.0000000	No	Sulfonylurea	SGLT2i	UC_1
0.6063954	0.3203563	0.0000000	No	Sulfonylurea	SGLT2i	UC_2
0.3984265	0.1598364	0.0117459	No	Sulfonylurea	SGLT2i	UC_3
-0.2651187	0.0755655	0.0227480	No	Sulfonylurea	SGLT2i	UC_4
-0.0680461	0.0089529	0.0503222	No	Sulfonylurea	SGLT2i	UC_5

© 2023 Vashisht R et al. *JAMA Network Open.*

4.28 Valvular Heart Disease

4.28.1 eTable: Drug comparision table

Effect size of each drug comparison at each UC health site is tabulated.

eTable 191: Hazard ratios of drug class comparison at each UC

Comparator	Treated	UC	N	Hazard Ratio (95% CI)	P-value	Adjusted P-Value
DPP4i	GLP1ra	UC_1	688	1.3 (0.46-3.8)	5.92589e-01	8.287419e-01
DPP4i	GLP1ra	UC_2	1014	0.77 (0.4-1.5)	4.37393e-01	8.287419e-01
DPP4i	GLP1ra	UC_3	832	0.63 (0.31-1.3)	2.13501e-01	8.287419e-01
DPP4i	GLP1ra	UC_4	540	1 (0.21-5)	9.83123e-01	9.831230e-01
DPP4i	GLP1ra	UC_5	2618	0.72 (0.46-1.1)	1.47855e-01	8.287419e-01
DPP4i	SGLT2i	UC_1	1028	0.95 (0.42-2.1)	8.95576e-01	9.264579e-01
DPP4i	SGLT2i	UC_2	963	0.84 (0.41-1.7)	6.27301e-01	8.287419e-01
DPP4i	SGLT2i	UC_3	666	0.76 (0.32-1.8)	5.42962e-01	8.287419e-01
DPP4i	SGLT2i	UC_4	534	0.76 (0.17-3.4)	7.18243e-01	8.287419e-01
DPP4i	SGLT2i	UC_5	2896	0.61 (0.39-0.94)	2.67373e-02	4.634040e-01
GLP1ra	SGLT2i	UC_1	699	0.71 (0.22-2.2)	5.54997e-01	8.287419e-01
GLP1ra	SGLT2i	UC_2	1157	0.89 (0.43-1.9)	7.58625e-01	8.429167e-01
GLP1ra	SGLT2i	UC_3	773	0.6 (0.25-1.4)	2.50693e-01	8.287419e-01
GLP1ra	SGLT2i	UC_4	648	0.65 (0.11-3.9)	6.38109e-01	8.287419e-01
GLP1ra	SGLT2i	UC_5	2983	1.1 (0.67-1.8)	6.99297e-01	8.287419e-01
Sulfonylurea	DPP4i	UC_1	1696	0.9 (0.54-1.5)	6.86555e-01	8.287419e-01
Sulfonylurea	DPP4i	UC_2	1915	0.68 (0.48-0.97)	3.08936e-02	4.634040e-01
Sulfonylurea	DPP4i	UC_3	2000	1 (0.72-1.5)	8.24106e-01	8.829707e-01
Sulfonylurea	DPP4i	UC_4	1228	0.82 (0.33-2.1)	6.83429e-01	8.287419e-01
Sulfonylurea	DPP4i	UC_5	5606	1.2 (0.92-1.6)	1.83053e-01	8.287419e-01
Sulfonylurea	GLP1ra	UC_1	728	0.62 (0.24-1.6)	3.17423e-01	8.287419e-01
Sulfonylurea	GLP1ra	UC_2	1104	0.8 (0.43-1.5)	4.94816e-01	8.287419e-01
Sulfonylurea	GLP1ra	UC_3	1026	1.4 (0.67-2.9)	3.77179e-01	8.287419e-01
Sulfonylurea	GLP1ra	UC_4	651	1.3 (0.3-6)	7.01175e-01	8.287419e-01
Sulfonylurea	GLP1ra	UC_5	2539	0.85 (0.53-1.4)	5.20103e-01	8.287419e-01
Sulfonylurea	SGLT2i	UC_1	1171	0.75 (0.38-1.5)	4.09882e-01	8.287419e-01
Sulfonylurea	SGLT2i	UC_2	1024	0.55 (0.29-1)	6.56400e-02	6.564000e-01
Sulfonylurea	SGLT2i	UC_3	891	1.5 (0.77-2.9)	2.43091e-01	8.287419e-01
Sulfonylurea	SGLT2i	UC_4	738	0.34 (0.09-1.2)	1.03854e-01	7.789050e-01
Sulfonylurea	SGLT2i	UC_5	2884	0.84 (0.52-1.4)	4.66746e-01	8.287419e-01

4.28.2 eFigure: Individual effect size, meta analysis and sensitivity analysis

The forest plot illustrate the effect size of the comparison between DPP4i and GLP1ra at each UC along with the effect size obtained from the random effect meta-analysis across all the UC for outcome Valvular Heart Disease

© 2023 Vashisht R et al. *JAMA Network Open.*

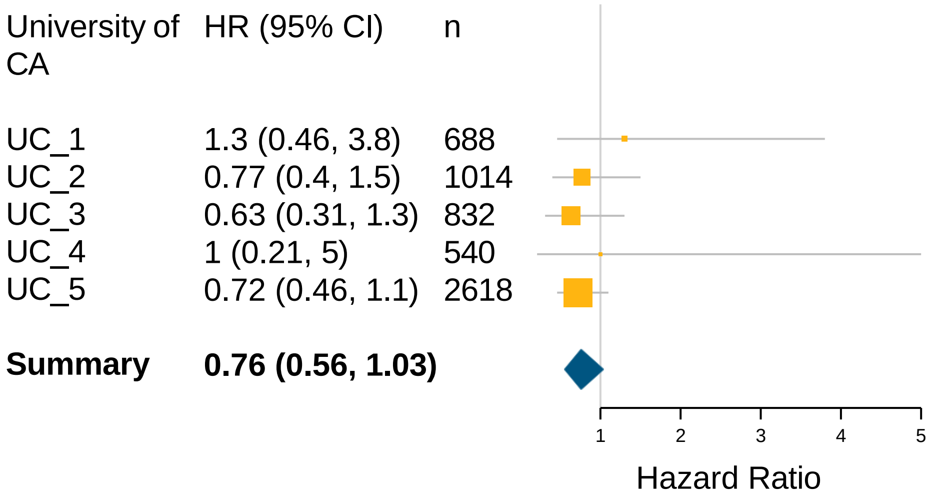

The table below shows the Leave-One-UC-Out diagnostics. The DFFITS value, Cook's distance, Covariance ratio, leave-one-out amount of heteroginity, indicator for influential estimates, comparator and treated groups are provided for each Leave-One-UC-Out analysis. The influential estimate from one UC with respect to pooled estimate are marked as Yes or No, with Yes indicating an influential UC and No otherwise.

eTable 192: Leave-One-UC-Out Sensitivity Analysis

DFFITs	Cook's Dist	Residual Heterogeneity	Influential	Comparator	Treated	UC
0.3149140	0.0991708	0	No	DPP4i	GLP1ra	UC_1
0.0273068	0.0007457	0	No	DPP4i	GLP1ra	UC_2
-0.2619658	0.0686261	0	No	DPP4i	GLP1ra	UC_3
0.0683221	0.0046679	0	No	DPP4i	GLP1ra	UC_4
-0.3148222	0.0991130	0	No	DPP4i	GLP1ra	UC_5

© 2023 Vashisht R et al. *JAMA Network Open.*

The forest plot illustrate the effect size of the comparison between DPP4i and SGLT2i at each UC along with the effect size obtained from the random effect meta-analysis across all the UC for outcome Valvular Heart Disease

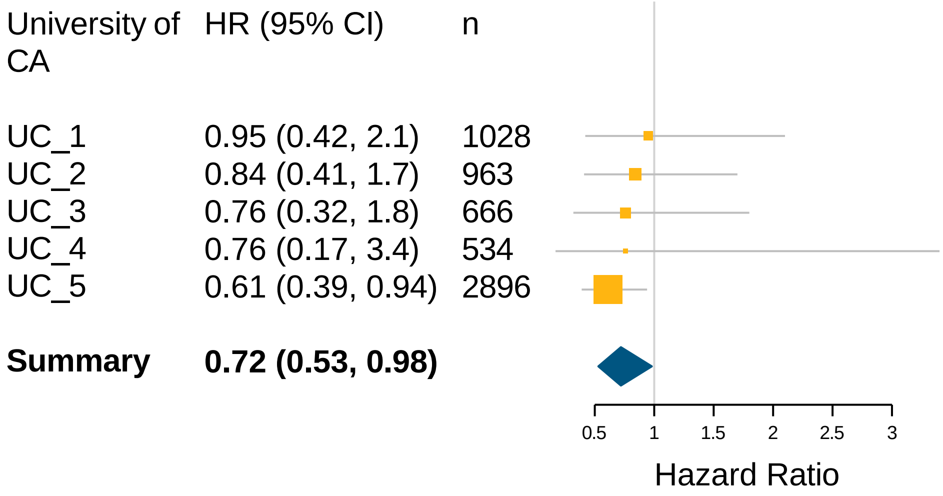

The table below shows the Leave-One-UC-Out diagnostics. The DFFITS value, Cook's distance, Covariance ratio, leave-one-out amount of heteroginity, indicator for influential estimates, comparator and treated groups are provided for each Leave-One-UC-Out analysis. The influential estimate from one UC with respect to pooled estimate are marked as Yes or No, with Yes indicating an influential UC and No otherwise.

eTable 193: Leave-One-UC-Out Sensitivity Analysis

DFFITs	Cook's Dist	Residual Heterogeneity	Influential	Comparator	Treated	UC
0.3068920	0.0941827	0	No	DPP4i	SGLT2i	UC_1
0.2313944	0.0535434	0	No	DPP4i	SGLT2i	UC_2
0.0527725	0.0027849	0	No	DPP4i	SGLT2i	UC_3
0.0159773	0.0002553	0	No	DPP4i	SGLT2i	UC_4
-1.0079280	1.0159189	0	Yes	DPP4i	SGLT2i	UC_5

The forest plot illustrate the effect size of the comparison between GLP1ra and SGLT2i at each UC along with the effect size obtained from the random effect meta-analysis across all the UC for outcome Valvular Heart Disease

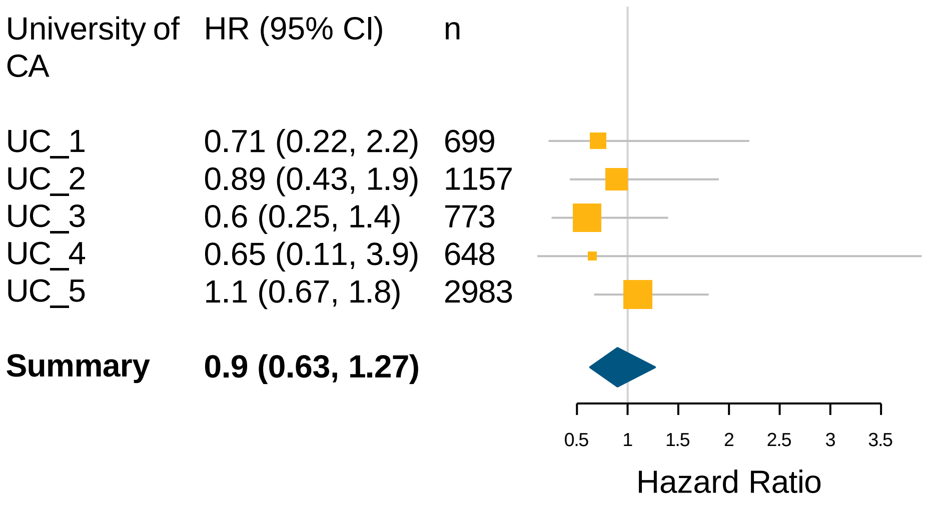

The table below shows the Leave-One-UC-Out diagnostics. The DFFITS value, Cook's distance, Covariance ratio, leave-one-out amount of heteroginity, indicator for influential estimates, comparator and treated groups are provided for each Leave-One-UC-Out analysis. The influential estimate from one UC with respect to pooled estimate are marked as Yes or No, with Yes indicating an influential UC and No otherwise.

eTable 194: Leave-One-UC-Out Sensitivity Analysis

DFFITs	Cook's Dist	Residual Heterogeneity	Influential	Comparator	Treated	UC
-0.1318896	0.0173949	0	No	GLP1ra	SGLT2i	UC_1
-0.0126681	0.0001605	0	No	GLP1ra	SGLT2i	UC_2
-0.4395455	0.1932003	0	No	GLP1ra	SGLT2i	UC_3
-0.0714837	0.0051099	0	No	GLP1ra	SGLT2i	UC_4
1.1162288	1.2459666	0	Yes	GLP1ra	SGLT2i	UC_5

© 2023 Vashisht R et al. *JAMA Network Open.*

The forest plot illustrate the effect size of the comparison between Sulfonylurea and DPP4i at each UC along with the effect size obtained from the random effect meta-analysis across all the UC for outcome Valvular Heart Disease

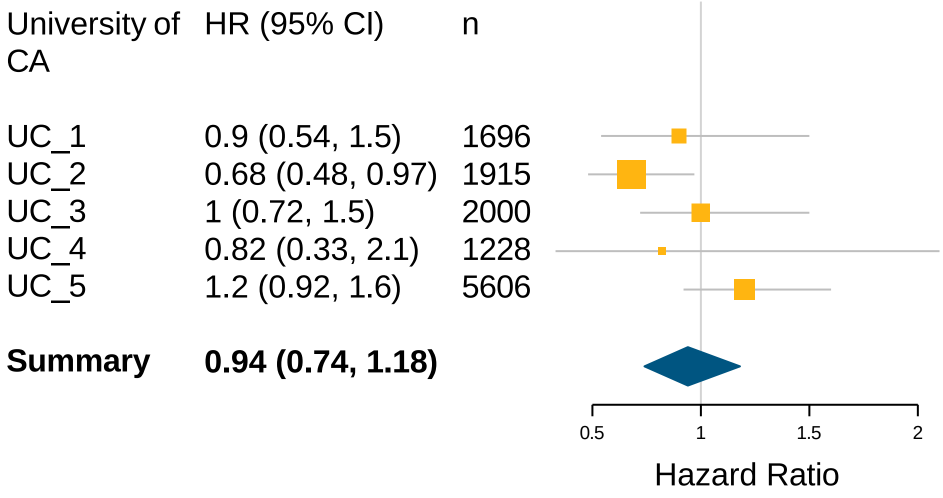

The table below shows the Leave-One-UC-Out diagnostics. The DFFITS value, Cook's distance, Covariance ratio, leave-one-out amount of heteroginity, indicator for influential estimates, comparator and treated groups are provided for each Leave-One-UC-Out analysis. The influential estimate from one UC with respect to pooled estimate are marked as Yes or No, with Yes indicating an influential UC and No otherwise.

eTable 195: Leave-One-UC-Out Sensitivity Analysis

DFFITs	Cook's Dist	Residual Heterogeneity	Influential	Comparator	Treated	UC
0.0005055	0.0000003	0.0437044	No	Sulfonylurea	DPP4i	UC_1
-1.5037867	1.2578410	0.0000000	Yes	Sulfonylurea	DPP4i	UC_2
0.2202881	0.0701956	0.0528272	No	Sulfonylurea	DPP4i	UC_3
-0.0347901	0.0012671	0.0373529	No	Sulfonylurea	DPP4i	UC_4
1.4945591	0.9756686	0.0000000	Yes	Sulfonylurea	DPP4i	UC_5

© 2023 Vashisht R et al. *JAMA Network Open.*

The forest plot illustrate the effect size of the comparison between Sulfonylurea and GLP1ra at each UC along with the effect size obtained from the random effect meta-analysis across all the UC for outcome Valvular Heart Disease

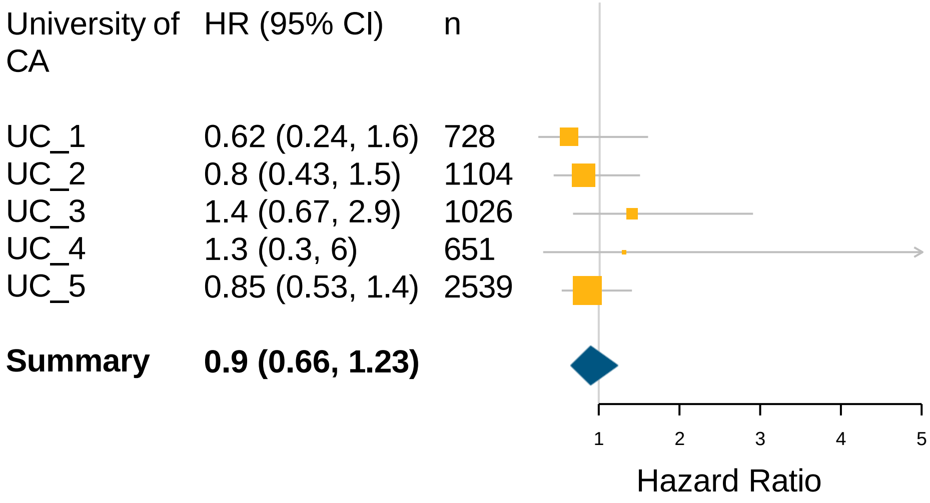

The table below shows the Leave-One-UC-Out diagnostics. The DFFITS value, Cook's distance, Covariance ratio, leave-one-out amount of heteroginity, indicator for influential estimates, comparator and treated groups are provided for each Leave-One-UC-Out analysis. The influential estimate from one UC with respect to pooled estimate are marked as Yes or No, with Yes indicating an influential UC and No otherwise.

eTable 196: Leave-One-UC-Out Sensitivity Analysis

DFFITs	Cook's Dist	Residual Heterogeneity	Influential	Comparator	Treated	UC
-0.2870399	0.0823919	0	No	Sulfonylurea	GLP1ra	UC_1
-0.2528112	0.0639135	0	No	Sulfonylurea	GLP1ra	UC_2
0.6132257	0.3760458	0	No	Sulfonylurea	GLP1ra	UC_3
0.1042488	0.0108678	0	No	Sulfonylurea	GLP1ra	UC_4
-0.2663392	0.0709365	0	No	Sulfonylurea	GLP1ra	UC_5

© 2023 Vashisht R et al. *JAMA Network Open.*

The forest plot illustrate the effect size of the comparison between Sulfonylurea and SGLT2i at each UC along with the effect size obtained from the random effect meta-analysis across all the UC for outcome Valvular Heart Disease

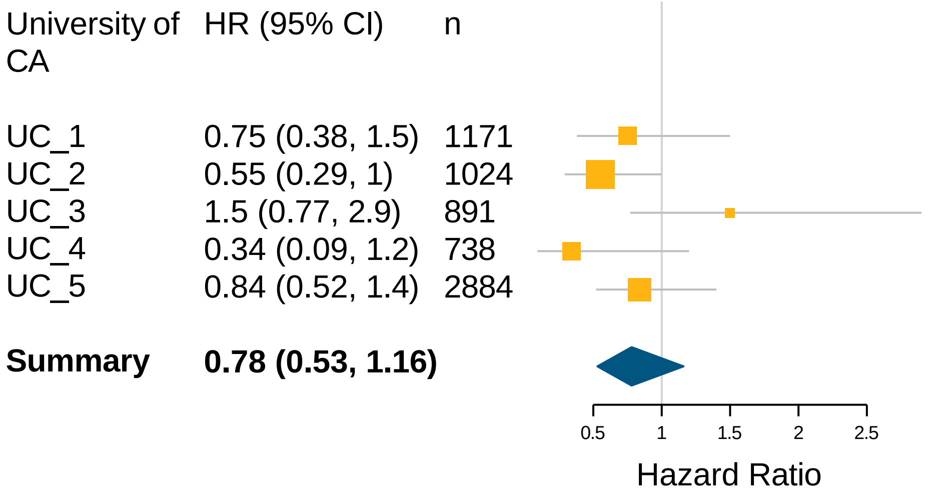

The table below shows the Leave-One-UC-Out diagnostics. The DFFITS value, Cook's distance, Covariance ratio, leave-one-out amount of heteroginity, indicator for influential estimates, comparator and treated groups are provided for each Leave-One-UC-Out analysis. The influential estimate from one UC with respect to pooled estimate are marked as Yes or No, with Yes indicating an influential UC and No otherwise.

eTable 197: Leave-One-UC-Out Sensitivity Analysis

DFFITs	Cook's Dist	Residual Heterogeneity	Influential	Comparator	Treated	UC
0.0185588	0.0004568	0.1426900	No	Sulfonylurea	SGLT2i	UC_1
-0.5211147	0.2706390	0.0768108	No	Sulfonylurea	SGLT2i	UC_2
0.8541685	0.4352111	0.0000000	No	Sulfonylurea	SGLT2i	UC_3
-0.3586014	0.1239988	0.0591715	No	Sulfonylurea	SGLT2i	UC_4
0.1821251	0.0554081	0.1721112	No	Sulfonylurea	SGLT2i	UC_5

© 2023 Vashisht R et al. *JAMA Network Open.*

4.29 Vomiting

4.29.1 eTable: Drug comparision table

Effect size of each drug comparison at each UC health site is tabulated.

eTable 198: Hazard ratios of drug class comparison at each UC

Comparator	Treated	UC	N	Hazard Ratio (95% CI)	P-value	Adjusted P-Value
DPP4i	GLP1ra	UC_1	666	1 (0.47-2.3)	9.18508e-01	9.555250e-01
DPP4i	GLP1ra	UC_2	1023	1.4 (0.75-2.5)	3.08265e-01	8.407227e-01
DPP4i	GLP1ra	UC_3	828	1.1 (0.5-2.2)	8.83610e-01	9.555250e-01
DPP4i	GLP1ra	UC_4	535	0.85 (0.35-2.1)	7.21746e-01	9.555250e-01
DPP4i	GLP1ra	UC_5	2608	1.4 (0.84-2.2)	2.14127e-01	8.043833e-01
DPP4i	SGLT2i	UC_1	1014	1.1 (0.51-2.6)	7.45207e-01	9.555250e-01
DPP4i	SGLT2i	UC_2	1027	0.51 (0.24-1.1)	8.45464e-02	5.907000e-01
DPP4i	SGLT2i	UC_3	667	0.82 (0.34-2)	6.68311e-01	9.555250e-01
DPP4i	SGLT2i	UC_4	533	0.63 (0.15-2.6)	5.22158e-01	9.555250e-01
DPP4i	SGLT2i	UC_5	2926	1 (0.62-1.6)	9.55525e-01	9.555250e-01
GLP1ra	SGLT2i	UC_1	679	0.57 (0.22-1.5)	2.41315e-01	8.043833e-01
GLP1ra	SGLT2i	UC_2	1229	0.73 (0.4-1.3)	3.03269e-01	8.407227e-01
GLP1ra	SGLT2i	UC_3	768	0.9 (0.4-2)	8.00778e-01	9.555250e-01
GLP1ra	SGLT2i	UC_4	625	0.35 (0.1-1.3)	1.18140e-01	5.907000e-01
GLP1ra	SGLT2i	UC_5	2988	0.65 (0.39-1.1)	1.02843e-01	5.907000e-01
Sulfonylurea	DPP4i	UC_1	1675	0.82 (0.49-1.4)	4.31462e-01	9.245614e-01
Sulfonylurea	DPP4i	UC_2	1962	1.4 (0.93-2.1)	1.08167e-01	5.907000e-01
Sulfonylurea	DPP4i	UC_3	2023	0.99 (0.63-1.5)	9.51467e-01	9.555250e-01
Sulfonylurea	DPP4i	UC_4	1241	1.4 (0.68-2.7)	3.82641e-01	9.245614e-01
Sulfonylurea	DPP4i	UC_5	5641	0.74 (0.56-0.99)	4.42942e-02	5.907000e-01
Sulfonylurea	GLP1ra	UC_1	710	1.2 (0.51-2.6)	7.35572e-01	9.555250e-01
Sulfonylurea	GLP1ra	UC_2	1104	1.4 (0.81-2.5)	2.25665e-01	8.043833e-01
Sulfonylurea	GLP1ra	UC_3	1022	1.3 (0.66-2.7)	4.21684e-01	9.245614e-01
Sulfonylurea	GLP1ra	UC_4	628	1.1 (0.44-3)	7.79511e-01	9.555250e-01
Sulfonylurea	GLP1ra	UC_5	2524	1.6 (0.99-2.7)	5.27023e-02	5.907000e-01
Sulfonylurea	SGLT2i	UC_1	1167	0.93 (0.46-1.9)	8.33061e-01	9.555250e-01
Sulfonylurea	SGLT2i	UC_2	1090	0.79 (0.38-1.7)	5.36509e-01	9.555250e-01
Sulfonylurea	SGLT2i	UC_3	919	0.96 (0.46-2)	9.17224e-01	9.555250e-01
Sulfonylurea	SGLT2i	UC_4	727	0.82 (0.22-3)	7.64176e-01	9.555250e-01
Sulfonylurea	SGLT2i	UC_5	2912	1.1 (0.69-1.9)	5.91332e-01	9.555250e-01

4.29.2 eFigure: Individual effect size, meta analysis and sensitivity analysis

The forest plot illustrate the effect size of the comparison between DPP4i and GLP1ra at each UC along with the effect size obtained from the random effect meta-analysis across all the UC for outcome Vomiting

© 2023 Vashisht R et al. *JAMA Network Open.*

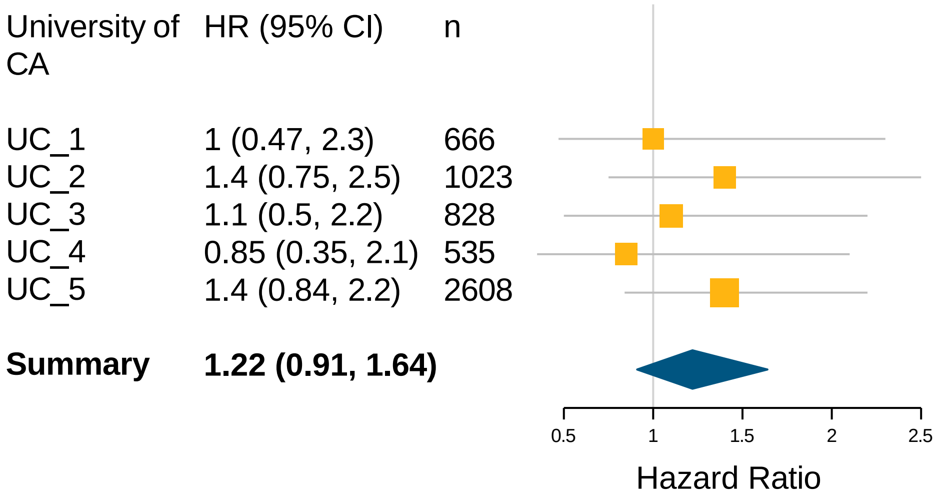

The table below shows the Leave-One-UC-Out diagnostics. The DFFITS value, Cook's distance, Covariance ratio, leave-one-out amount of heteroginity, indicator for influential estimates, comparator and treated groups are provided for each Leave-One-UC-Out analysis. The influential estimate from one UC with respect to pooled estimate are marked as Yes or No, with Yes indicating an influential UC and No otherwise.

eTable 199: Leave-One-UC-Out Sensitivity Analysis

DFFITs	Cook's Dist	Residual Heterogeneity	Influential	Comparator	Treated	UC
-0.2104836	0.0443033	0	No	DPP4i	GLP1ra	UC_1
0.2808139	0.0788564	0	No	DPP4i	GLP1ra	UC_2
-0.1298602	0.0168637	0	No	DPP4i	GLP1ra	UC_3
-0.2896081	0.0838729	0	No	DPP4i	GLP1ra	UC_4
0.5312341	0.2822097	0	No	DPP4i	GLP1ra	UC_5

© 2023 Vashisht R et al. *JAMA Network Open.*

The forest plot illustrate the effect size of the comparison between DPP4i and SGLT2i at each UC along with the effect size obtained from the random effect meta-analysis across all the UC for outcome Vomiting

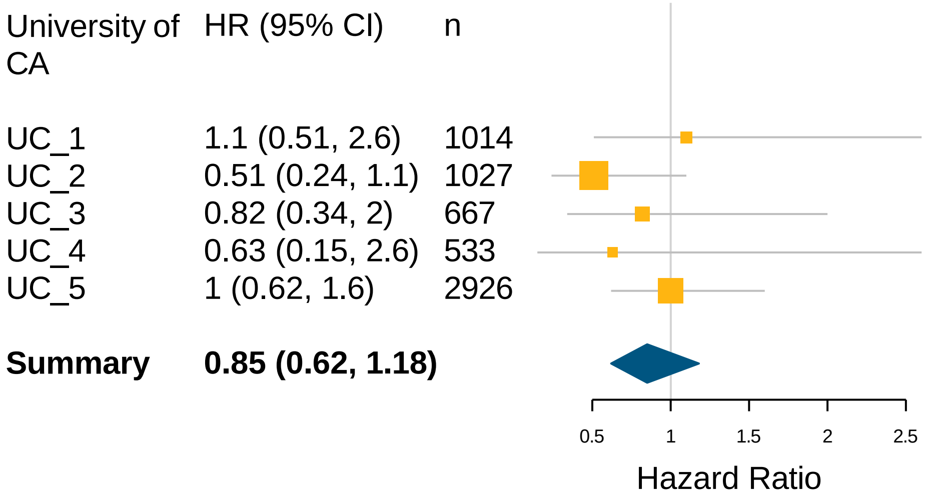

The table below shows the Leave-One-UC-Out diagnostics. The DFFITS value, Cook's distance, Covariance ratio, leave-one-out amount of heteroginity, indicator for influential estimates, comparator and treated groups are provided for each Leave-One-UC-Out analysis. The influential estimate from one UC with respect to pooled estimate are marked as Yes or No, with Yes indicating an influential UC and No otherwise.

eTable 200: Leave-One-UC-Out Sensitivity Analysis

DFFITs	Cook's Dist	Residual Heterogeneity	Influential	Comparator	Treated	UC
0.2903604	0.0843091	0	No	DPP4i	SGLT2i	UC_1
-0.6930159	0.4802710	0	Yes	DPP4i	SGLT2i	UC_2
-0.0374522	0.0014027	0	No	DPP4i	SGLT2i	UC_3
-0.1003321	0.0100665	0	No	DPP4i	SGLT2i	UC_4
0.8504957	0.7233430	0	Yes	DPP4i	SGLT2i	UC_5

© 2023 Vashisht R et al. *JAMA Network Open.*

The forest plot illustrate the effect size of the comparison between GLP1ra and SGLT2i at each UC along with the effect size obtained from the random effect meta-analysis across all the UC for outcome Vomiting

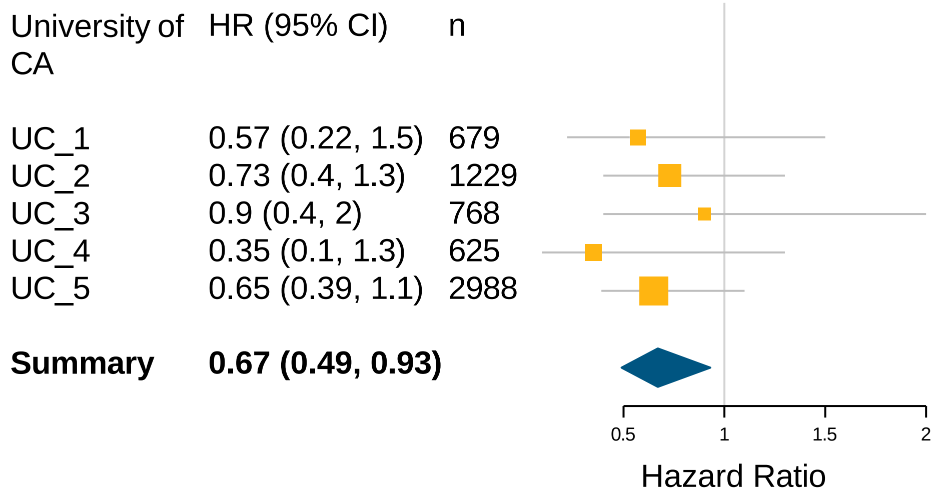

The table below shows the Leave-One-UC-Out diagnostics. The DFFITS value, Cook's distance, Covariance ratio, leave-one-out amount of heteroginity, indicator for influential estimates, comparator and treated groups are provided for each Leave-One-UC-Out analysis. The influential estimate from one UC with respect to pooled estimate are marked as Yes or No, with Yes indicating an influential UC and No otherwise.

eTable 201: Leave-One-UC-Out Sensitivity Analysis

DFFITs	Cook's Dist	Residual Heterogeneity	Influential	Comparator	Treated	UC
-0.1261574	0.0159157	0	No	GLP1ra	SGLT2i	UC_1
0.2095651	0.0439175	0	No	GLP1ra	SGLT2i	UC_2
0.3342823	0.1117447	0	No	GLP1ra	SGLT2i	UC_3
-0.2538361	0.0644327	0	No	GLP1ra	SGLT2i	UC_4
-0.1269708	0.0161216	0	No	GLP1ra	SGLT2i	UC_5

The forest plot illustrate the effect size of the comparison between Sulfonylurea and DPP4i at each UC along with the effect size obtained from the random effect meta-analysis across all the UC for outcome Vomiting

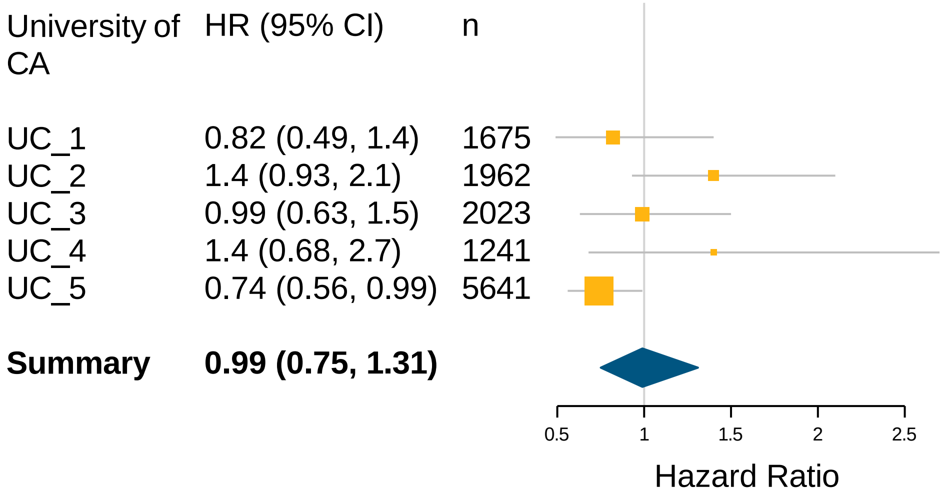

The table below shows the Leave-One-UC-Out diagnostics. The DFFITS value, Cook's distance, Covariance ratio, leave-one-out amount of heteroginity, indicator for influential estimates, comparator and treated groups are provided for each Leave-One-UC-Out analysis. The influential estimate from one UC with respect to pooled estimate are marked as Yes or No, with Yes indicating an influential UC and No otherwise.

eTable 202: Leave-One-UC-Out Sensitivity Analysis

DFFITs	Cook's Dist	Residual Heterogeneity	Influential	Comparator	Treated	UC
-0.3178759	0.1198938	0.0707311	No	Sulfonylurea	DPP4i	UC_1
1.3040085	0.9365233	0.0072245	Yes	Sulfonylurea	DPP4i	UC_2
-0.0802393	0.0086932	0.0824164	No	Sulfonylurea	DPP4i	UC_3
0.3188681	0.1025740	0.0498535	No	Sulfonylurea	DPP4i	UC_4
-1.4212370	0.7511205	0.0047030	Yes	Sulfonylurea	DPP4i	UC_5

© 2023 Vashisht R et al. *JAMA Network Open.*

The forest plot illustrate the effect size of the comparison between Sulfonylurea and GLP1ra at each UC along with the effect size obtained from the random effect meta-analysis across all the UC for outcome Vomiting

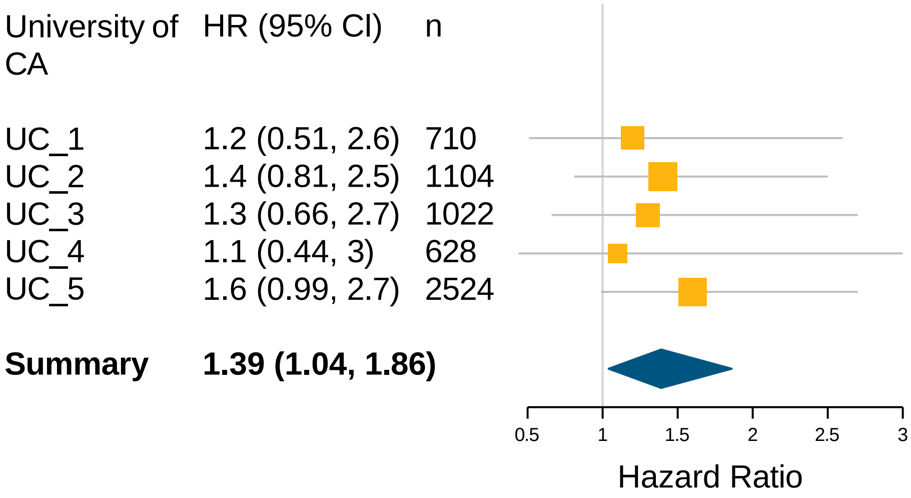

The table below shows the Leave-One-UC-Out diagnostics. The DFFITS value, Cook's distance, Covariance ratio, leave-one-out amount of heteroginity, indicator for influential estimates, comparator and treated groups are provided for each Leave-One-UC-Out analysis. The influential estimate from one UC with respect to pooled estimate are marked as Yes or No, with Yes indicating an influential UC and No otherwise.

eTable 203: Leave-One-UC-Out Sensitivity Analysis

DFFITs	Cook's Dist	Residual Heterogeneity	Influential	Comparator	Treated	UC
-0.1430676	0.0204683	0	No	Sulfonylurea	GLP1ra	UC_1
0.0237143	0.0005624	0	No	Sulfonylurea	GLP1ra	UC_2
-0.0897942	0.0080630	0	No	Sulfonylurea	GLP1ra	UC_3
-0.1584880	0.0251184	0	No	Sulfonylurea	GLP1ra	UC_4
0.4922627	0.2423226	0	No	Sulfonylurea	GLP1ra	UC_5

© 2023 Vashisht R et al. *JAMA Network Open.*

The forest plot illustrate the effect size of the comparison between Sulfonylurea and SGLT2i at each UC along with the effect size obtained from the random effect meta-analysis across all the UC for outcome Vomiting

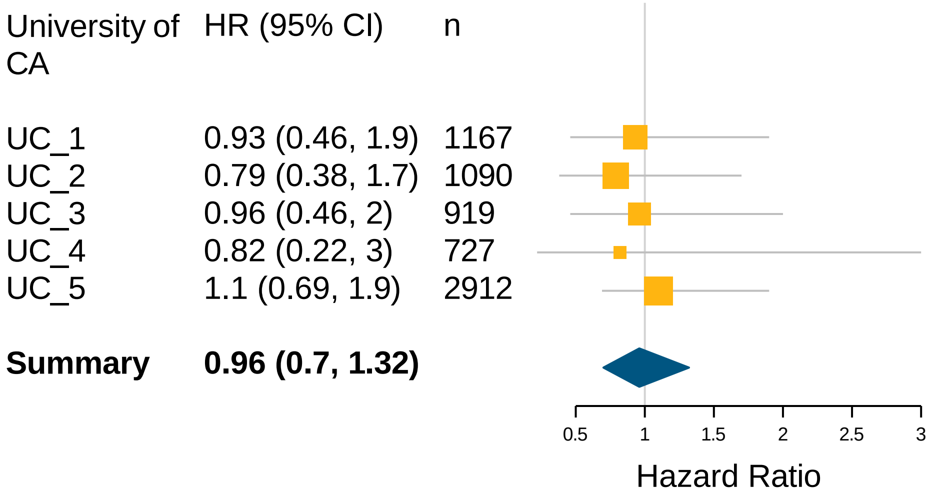

The table below shows the Leave-One-UC-Out diagnostics. The DFFITS value, Cook's distance, Covariance ratio, leave-one-out amount of heteroginity, indicator for influential estimates, comparator and treated groups are provided for each Leave-One-UC-Out analysis. The influential estimate from one UC with respect to pooled estimate are marked as Yes or No, with Yes indicating an influential UC and No otherwise.

eTable 204: Leave-One-UC-Out Sensitivity Analysis

DFFITs	Cook's Dist	Residual Heterogeneity	Influential	Comparator	Treated	UC
-0.0526641	0.0027735	0	No	Sulfonylurea	SGLT2i	UC_1
-0.2636467	0.0695096	0	No	Sulfonylurea	SGLT2i	UC_2
-0.0038620	0.0000149	0	No	Sulfonylurea	SGLT2i	UC_3
-0.0615078	0.0037832	0	No	Sulfonylurea	SGLT2i	UC_4
0.5218680	0.2723462	0	No	Sulfonylurea	SGLT2i	UC_5

© 2023 Vashisht R et al. *JAMA Network Open.*

eFigure. Forest Plot of Summary Estimates of Secondary Outcomes

The figure illustrate the summary hazard ratios of the comparative safety of T2D treatment across UC Health. Each forest plot illustrates the drug pair comparison (matched Cc-Tc pair), number of patients (N) in Cc and Tc across UC Health, the summary hazard ratios (sHR) and corresponding 95% confidence intervals obtained from the random effect meta-analysis across UC Health, the heterogeneity I2 of the sHR and the stability of sHR based on leave-one-medical-center-out influence analysis. Detailed forest plots of each drug pair comparison at each UC Health site along with the random effect meta-analysis summary estimates across UC Health including leave-one-medical-center-out influence analysis id provided in eSection-4.2-4.29

© 2023 Vashisht R et al. *JAMA Network Open.*

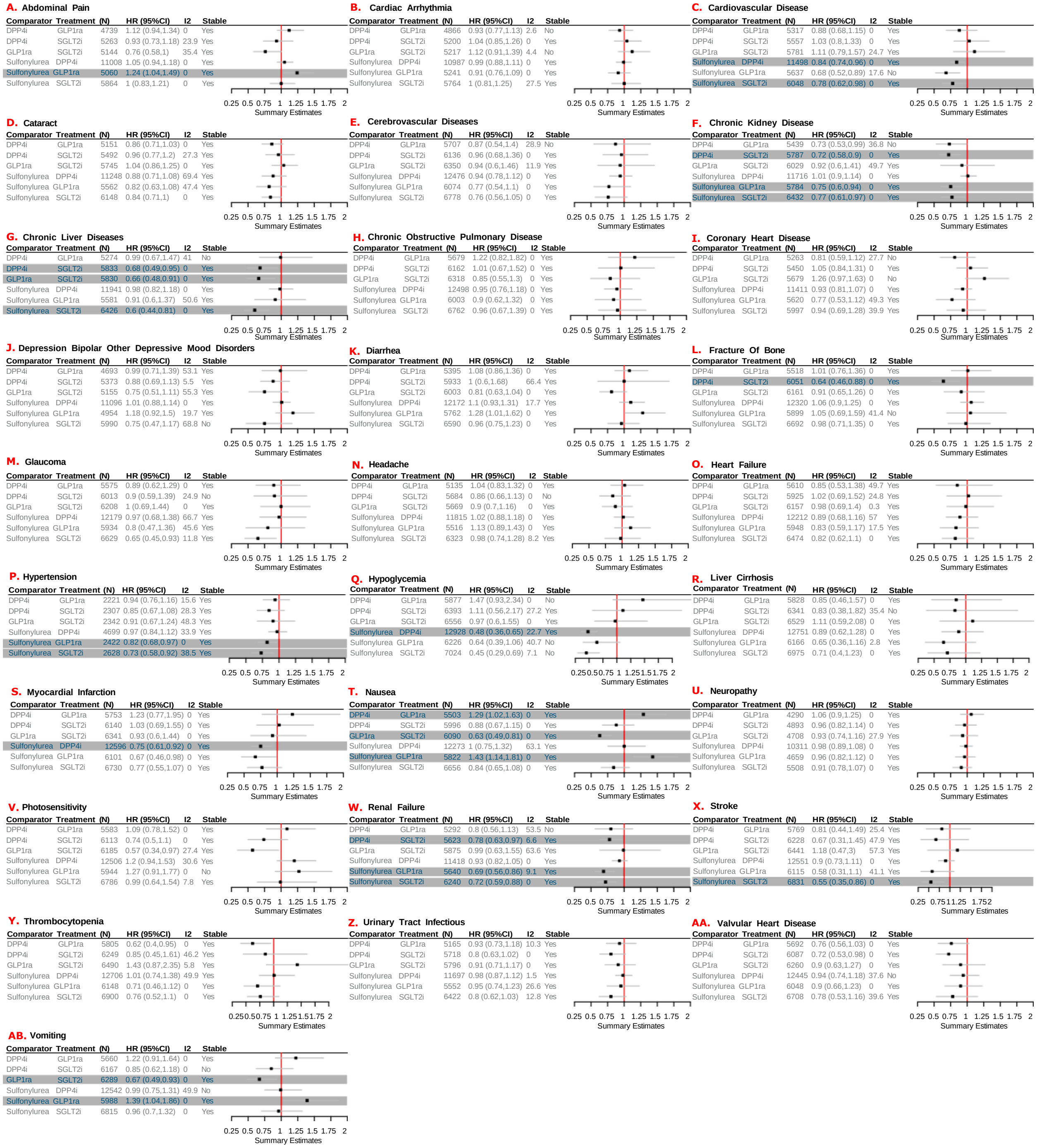

A. Abdominal Pain

Comparator	Treatment	(N)	HR (95%CI)	I2	Stable
DPP4i	GLP1ra	4739	1.12 (0.94,1.34)	0	Yes
DPP4i	SGLT2i	5263	0.93 (0.73,1.18)	23.9	Yes
GLP1ra	SGLT2i	5144	0.76 (0.58,1)	35.4	Yes
Sulfonylurea	DPP4i	11008	1.05 (0.94,1.18)	0	Yes
Sulfonylurea	GLP1ra	5060	1.24 (1.04,1.49)	0	Yes
Sulfonylurea	SGLT2i	5864	1 (0.83,1.21)	0	Yes

B. Cardiac Arrhythmia

Comparator	Treatment	(N)	HR (95%CI)	I2	Stable
DPP4i	GLP1ra	4866	0.93 (0.77,1.13)	2.6	No
DPP4i	SGLT2i	5200	1.04 (0.85,1.26)	0	Yes
GLP1ra	SGLT2i	5217	1.12 (0.91,1.39)	4.4	No
Sulfonylurea	DPP4i	10987	0.99 (0.88,1.11)	0	Yes
Sulfonylurea	GLP1ra	5241	0.91 (0.76,1.09)	0	Yes
Sulfonylurea	SGLT2i	5764	1 (0.81,1.25)	27.5	Yes

C. Cardiovascular Disease

Comparator	Treatment	(N)	HR (95%CI)	I2	Stable
DPP4i	GLP1ra	5317	0.88 (0.68,1.15)	0	Yes
DPP4i	SGLT2i	5557	1.03 (0.8,1.33)	0	Yes
GLP1ra	SGLT2i	5781	1.11 (0.79,1.57)	24.7	Yes
Sulfonylurea	DPP4i	11498	0.84 (0.74,0.96)	0	Yes
Sulfonylurea	GLP1ra	5637	0.68 (0.52,0.89)	17.6	No
Sulfonylurea	SGLT2i	6048	0.78 (0.62,0.98)	0	Yes

D. Cataract

Comparator	Treatment	(N)	HR (95%CI)	I2	Stable
DPP4i	GLP1ra	5151	0.86 (0.71,1.03)	0	Yes
DPP4i	SGLT2i	5492	0.96 (0.77,1.2)	27.3	Yes
GLP1ra	SGLT2i	5745	1.04 (0.86,1.25)	0	Yes
Sulfonylurea	DPP4i	11248	0.88 (0.71,1.08)	69.4	Yes
Sulfonylurea	GLP1ra	5562	0.82 (0.63,1.08)	47.4	Yes
Sulfonylurea	SGLT2i	6148	0.84 (0.71,1)	0	Yes

E. Cerebrovascular Diseases

Comparator	Treatment	(N)	HR (95%CI)	I2	Stable
DPP4i	GLP1ra	5707	0.87 (0.54,1.4)	28.9	No
DPP4i	SGLT2i	6136	0.96 (0.68,1.36)	0	Yes
GLP1ra	SGLT2i	6350	0.94 (0.6,1.46)	11.9	Yes
Sulfonylurea	DPP4i	12476	0.94 (0.78,1.12)	0	Yes
Sulfonylurea	GLP1ra	6074	0.77 (0.54,1.1)	0	Yes
Sulfonylurea	SGLT2i	6778	0.76 (0.56,1.05)	0	Yes

F. Chronic Kidney Disease

Comparator	Treatment	(N)	HR (95%CI)	I2	Stable
DPP4i	GLP1ra	5439	0.73 (0.53,0.99)	36.8	No
DPP4i	SGLT2i	5787	0.72 (0.58,0.9)	0	Yes
GLP1ra	SGLT2i	6029	0.92 (0.6,1.41)	49.7	Yes
Sulfonylurea	DPP4i	11716	1.01 (0.9,1.14)	0	Yes
Sulfonylurea	GLP1ra	5784	0.75 (0.6,0.94)	0	Yes
Sulfonylurea	SGLT2i	6432	0.77 (0.61,0.97)	0	Yes

G. Chronic Liver Diseases

Comparator	Treatment	(N)	HR (95%CI)	I2	Stable
DPP4i	GLP1ra	5274	0.99 (0.67,1.47)	41	No
DPP4i	SGLT2i	5833	0.68 (0.49,0.95)	0	Yes
GLP1ra	SGLT2i	5830	0.66 (0.48,0.91)	0	Yes
Sulfonylurea	DPP4i	11941	0.98 (0.82,1.18)	0	Yes
Sulfonylurea	GLP1ra	5581	0.91 (0.6,1.37)	50.6	Yes
Sulfonylurea	SGLT2i	6426	0.6 (0.44,0.81)	0	Yes

H. Chronic Obstructive Pulmonary Disease

Comparator	Treatment	(N)	HR (95%CI)	I2	Stable
DPP4i	GLP1ra	5679	1.22 (0.82,1.82)	0	Yes
DPP4i	SGLT2i	6162	1.01 (0.67,1.52)	0	Yes
GLP1ra	SGLT2i	6318	0.85 (0.55,1.3)	0	Yes
Sulfonylurea	DPP4i	12498	0.95 (0.76,1.18)	0	Yes
Sulfonylurea	GLP1ra	6003	0.9 (0.62,1.32)	0	Yes
Sulfonylurea	SGLT2i	6762	0.96 (0.67,1.39)	0	Yes

I. Coronary Heart Disease

Comparator	Treatment	(N)	HR (95%CI)	I2	Stable
DPP4i	GLP1ra	5263	0.81 (0.59,1.12)	27.7	No
DPP4i	SGLT2i	5450	1.05 (0.84,1.31)	0	Yes
GLP1ra	SGLT2i	5679	1.26 (0.97,1.63)	0	No
Sulfonylurea	DPP4i	11411	0.93 (0.81,1.07)	0	Yes
Sulfonylurea	GLP1ra	5620	0.77 (0.53,1.12)	49.3	Yes
Sulfonylurea	SGLT2i	5997	0.94 (0.69,1.28)	39.9	Yes

J. Depression Bipolar Other Depressive Mood Disorders

Comparator	Treatment	(N)	HR (95%CI)	I2	Stable
DPP4i	GLP1ra	4693	0.99 (0.71,1.39)	53.1	Yes
DPP4i	SGLT2i	5373	0.88 (0.69,1.13)	5.5	Yes
GLP1ra	SGLT2i	5155	0.75 (0.51,1.11)	55.3	Yes
Sulfonylurea	DPP4i	11096	1.01 (0.88,1.14)	0	Yes
Sulfonylurea	GLP1ra	4954	1.18 (0.92,1.5)	19.7	Yes
Sulfonylurea	SGLT2i	5990	0.75 (0.47,1.17)	68.8	No

K. Diarrhea

Comparator	Treatment	(N)	HR (95%CI)	I2	Stable
DPP4i	GLP1ra	5395	1.08 (0.86,1.36)	0	Yes
DPP4i	SGLT2i	5933	1 (0.6,1.68)	66.4	Yes
GLP1ra	SGLT2i	6003	0.81 (0.63,1.04)	0	Yes
Sulfonylurea	DPP4i	12172	1.1 (0.93,1.31)	17.7	Yes
Sulfonylurea	GLP1ra	5762	1.28 (1.01,1.62)	0	Yes
Sulfonylurea	SGLT2i	6590	0.96 (0.75,1.23)	0	Yes

L. Fracture Of Bone

Comparator	Treatment	(N)	HR (95%CI)	I2	Stable
DPP4i	GLP1ra	5518	1.01 (0.76,1.36)	0	Yes
DPP4i	SGLT2i	6051	0.64 (0.46,0.88)	0	Yes
GLP1ra	SGLT2i	6161	0.91 (0.65,1.26)	0	Yes
Sulfonylurea	DPP4i	12320	1.06 (0.9,1.25)	0	Yes
Sulfonylurea	GLP1ra	5899	1.05 (0.69,1.59)	41.4	No
Sulfonylurea	SGLT2i	6692	0.98 (0.71,1.35)	0	Yes

M. Glaucoma

Comparator	Treatment	(N)	HR (95%CI)	I2	Stable
DPP4i	GLP1ra	5575	0.89 (0.62,1.29)	0	Yes
DPP4i	SGLT2i	6013	0.9 (0.59,1.39)	24.9	No
GLP1ra	SGLT2i	6208	1 (0.69,1.44)	0	Yes
Sulfonylurea	DPP4i	12179	0.97 (0.68,1.38)	66.7	Yes
Sulfonylurea	GLP1ra	5934	0.8 (0.47,1.36)	45.6	Yes
Sulfonylurea	SGLT2i	6629	0.65 (0.45,0.93)	11.8	Yes

N. Headache

Comparator	Treatment	(N)	HR (95%CI)	I2	Stable
DPP4i	GLP1ra	5135	1.04 (0.83,1.32)	0	Yes
DPP4i	SGLT2i	5684	0.86 (0.66,1.13)	0	No
GLP1ra	SGLT2i	5669	0.9 (0.7,1.16)	0	Yes
Sulfonylurea	DPP4i	11815	1.02 (0.88,1.18)	0	Yes
Sulfonylurea	GLP1ra	5516	1.13 (0.89,1.43)	0	Yes
Sulfonylurea	SGLT2i	6323	0.98 (0.74,1.28)	8.2	Yes

O. Heart Failure

Comparator	Treatment	(N)	HR (95%CI)	I2	Stable
DPP4i	GLP1ra	5610	0.85 (0.53,1.38)	49.7	Yes
DPP4i	SGLT2i	5925	1.02 (0.69,1.52)	24.8	Yes
GLP1ra	SGLT2i	6157	0.98 (0.69,1.4)	0.3	Yes
Sulfonylurea	DPP4i	12212	0.89 (0.68,1.16)	57	Yes
Sulfonylurea	GLP1ra	5948	0.83 (0.59,1.17)	17.5	Yes
Sulfonylurea	SGLT2i	6474	0.82 (0.62,1.1)	0	Yes

P. Hypertension

Comparator	Treatment	(N)	HR (95%CI)	I2	Stable
DPP4i	GLP1ra	2221	0.94 (0.76,1.16)	15.6	Yes
DPP4i	SGLT2i	2307	0.85 (0.67,1.08)	28.3	Yes
GLP1ra	SGLT2i	2342	0.91 (0.67,1.24)	48.3	Yes
Sulfonylurea	DPP4i	4699	0.97 (0.84,1.12)	33.9	Yes
Sulfonylurea	GLP1ra	2422	0.82 (0.68,0.97)	0	Yes
Sulfonylurea	SGLT2i	2628	0.73 (0.58,0.92)	38.5	Yes

Q. Hypoglycemia

Comparator	Treatment	(N)	HR (95%CI)	I2	Stable
DPP4i	GLP1ra	5877	1.47 (0.93,2.34)	0	No
DPP4i	SGLT2i	6393	1.11 (0.56,2.17)	27.2	Yes
GLP1ra	SGLT2i	6556	0.97 (0.6,1.55)	0	Yes
Sulfonylurea	DPP4i	12928	0.48 (0.36,0.65)	22.7	Yes
Sulfonylurea	GLP1ra	6226	0.64 (0.39,1.06)	40.7	No
Sulfonylurea	SGLT2i	7024	0.45 (0.29,0.69)	7.1	No

R. Liver Cirrhosis

Comparator	Treatment	(N)	HR (95%CI)	I2	Stable
DPP4i	GLP1ra	5828	0.85 (0.46,1.57)	0	Yes
DPP4i	SGLT2i	6341	0.83 (0.38,1.82)	35.4	No
GLP1ra	SGLT2i	6529	1.11 (0.59,2.08)	0	Yes
Sulfonylurea	DPP4i	12751	0.89 (0.62,1.28)	0	Yes
Sulfonylurea	GLP1ra	6166	0.65 (0.36,1.16)	2.8	Yes
Sulfonylurea	SGLT2i	6975	0.71 (0.4,1.23)	0	Yes

S. Myocardial Infarction

Comparator	Treatment	(N)	HR (95%CI)	I2	Stable
DPP4i	GLP1ra	5753	1.23 (0.77,1.95)	0	Yes
DPP4i	SGLT2i	6140	1.03 (0.69,1.55)	0	Yes
GLP1ra	SGLT2i	6341	0.93 (0.6,1.44)	0	Yes
Sulfonylurea	DPP4i	12596	0.75 (0.61,0.92)	0	Yes
Sulfonylurea	GLP1ra	6101	0.67 (0.46,0.98)	0	Yes
Sulfonylurea	SGLT2i	6730	0.77 (0.55,1.07)	0	Yes

T. Nausea

Comparator	Treatment	(N)	HR (95%CI)	I2	Stable
DPP4i	GLP1ra	5503	1.29 (1.02,1.63)	0	Yes
DPP4i	SGLT2i	5996	0.88 (0.67,1.15)	0	Yes
GLP1ra	SGLT2i	6090	0.63 (0.49,0.81)	0	Yes
Sulfonylurea	DPP4i	12273	1 (0.75,1.32)	63.1	Yes
Sulfonylurea	GLP1ra	5822	1.43 (1.14,1.81)	0	Yes
Sulfonylurea	SGLT2i	6656	0.84 (0.65,1.08)	0	Yes

U. Neuropathy

Comparator	Treatment	(N)	HR (95%CI)	I2	Stable
DPP4i	GLP1ra	4290	1.06 (0.9,1.25)	0	Yes
DPP4i	SGLT2i	4893	0.96 (0.82,1.14)	0	Yes
GLP1ra	SGLT2i	4708	0.93 (0.74,1.16)	27.9	Yes
Sulfonylurea	DPP4i	10311	0.98 (0.89,1.08)	0	Yes
Sulfonylurea	GLP1ra	4659	0.96 (0.82,1.12)	0	Yes
Sulfonylurea	SGLT2i	5508	0.91 (0.78,1.07)	0	Yes

V. Photosensitivity

Comparator	Treatment	(N)	HR (95%CI)	I2	Stable
DPP4i	GLP1ra	5583	1.09 (0.78,1.52)	0	Yes
DPP4i	SGLT2i	6113	0.74 (0.5,1.1)	0	Yes
GLP1ra	SGLT2i	6185	0.57 (0.34,0.97)	27.4	Yes
Sulfonylurea	DPP4i	12506	1.2 (0.94,1.53)	30.6	Yes
Sulfonylurea	GLP1ra	5944	1.27 (0.91,1.77)	0	No
Sulfonylurea	SGLT2i	6786	0.99 (0.64,1.54)	7.8	Yes

W. Renal Failure

Comparator	Treatment	(N)	HR (95%CI)	I2	Stable
DPP4i	GLP1ra	5292	0.8 (0.56,1.13)	53.5	No
DPP4i	SGLT2i	5623	0.78 (0.63,0.97)	6.6	Yes
GLP1ra	SGLT2i	5875	0.99 (0.63,1.55)	63.6	Yes
Sulfonylurea	DPP4i	11418	0.93 (0.82,1.05)	0	Yes
Sulfonylurea	GLP1ra	5640	0.69 (0.56,0.86)	9.1	Yes
Sulfonylurea	SGLT2i	6240	0.72 (0.59,0.88)	0	Yes

X. Stroke

Comparator	Treatment	(N)	HR (95%CI)	I2	Stable
DPP4i	GLP1ra	5769	0.81 (0.44,1.49)	25.4	Yes
DPP4i	SGLT2i	6228	0.67 (0.31,1.45)	47.9	Yes
GLP1ra	SGLT2i	6441	1.18 (0.47,3)	57.3	Yes
Sulfonylurea	DPP4i	12551	0.9 (0.73,1.11)	0	Yes
Sulfonylurea	GLP1ra	6115	0.58 (0.31,1.1)	41.1	Yes
Sulfonylurea	SGLT2i	6831	0.55 (0.35,0.86)	0	Yes

Y. Thrombocytopenia

Comparator	Treatment	(N)	HR (95%CI)	I2	Stable
DPP4i	GLP1ra	5805	0.62 (0.4,0.95)	0	Yes
DPP4i	SGLT2i	6249	0.85 (0.45,1.61)	46.2	Yes
GLP1ra	SGLT2i	6490	1.43 (0.87,2.35)	5.8	Yes
Sulfonylurea	DPP4i	12706	1.01 (0.74,1.38)	49.9	Yes
Sulfonylurea	GLP1ra	6148	0.71 (0.46,1.12)	0	Yes
Sulfonylurea	SGLT2i	6900	0.76 (0.52,1.1)	0	Yes

Z. Urinary Tract Infectious

Comparator	Treatment	(N)	HR (95%CI)	I2	Stable
DPP4i	GLP1ra	5165	0.93 (0.73,1.18)	10.3	Yes
DPP4i	SGLT2i	5718	0.8 (0.63,1.02)	0	Yes
GLP1ra	SGLT2i	5796	0.91 (0.71,1.17)	0	Yes
Sulfonylurea	DPP4i	11697	0.98 (0.87,1.12)	1.5	Yes
Sulfonylurea	GLP1ra	5552	0.95 (0.74,1.23)	26.6	Yes
Sulfonylurea	SGLT2i	6422	0.8 (0.62,1.03)	12.8	Yes

AA. Valvular Heart Disease

Comparator	Treatment	(N)	HR (95%CI)	I2	Stable
DPP4i	GLP1ra	5692	0.76 (0.56,1.03)	0	Yes
DPP4i	SGLT2i	6087	0.72 (0.53,0.98)	0	Yes
GLP1ra	SGLT2i	6260	0.9 (0.63,1.27)	0	Yes
Sulfonylurea	DPP4i	12445	0.94 (0.74,1.18)	37.6	No
Sulfonylurea	GLP1ra	6048	0.9 (0.66,1.23)	0	Yes
Sulfonylurea	SGLT2i	6708	0.78 (0.53,1.16)	39.6	Yes

AB. Vomiting

Comparator	Treatment	(N)	HR (95%CI)	I2	Stable
DPP4i	GLP1ra	5660	1.22 (0.91,1.64)	0	Yes
DPP4i	SGLT2i	6167	0.85 (0.62,1.18)	0	No
GLP1ra	SGLT2i	6289	0.67 (0.49,0.93)	0	Yes
Sulfonylurea	DPP4i	12542	0.99 (0.75,1.31)	49.9	No
Sulfonylurea	GLP1ra	5988	1.39 (1.04,1.86)	0	Yes
Sulfonylurea	SGLT2i	6815	0.96 (0.7,1.32)	0	Yes

eFigure 47: Forest Plot of Summary Estimates of Secondary Outcomes

© 2023 Vashisht R et al. *JAMA Network Open.*

eAppendix 4. Secondary Outcomes

Following definitions for the secondary outcomes were utilized in this study. Highlighted are the parent phenotype and sub phenotype SNOMED codes.

4.30 eTable: Abdominal Pain

eTable 205: Phenotype and Sub Phenotype SNOMEDs

Phenotype	SNOMED
Abdominal Pain	301715003; 43478001; 301717006; 14700001000004102; 247362001; 371102005; 438506002; 74704000; 83132003; 1119217009; 838411007; 162046002; 116290004; 285388000; 71850005; 9991008; 301754002; 301716002; 102614006; 21522001; 102613000; 304542004; 271858001; 60043000; 439469002; 707597009; 1119218004; 162042000; 162038003; 285387005; 54586004; 45979003

4.31 eTable: Cardiac Arrhythmia

eTable 206: Phenotype and Sub Phenotype SNOMEDs

Phenotype	SNOMED
Cardiac Arrhythmia	4973001; 6374002; 12026006; 270492004; 5370000; 46619002; 233917008; 29717002; 30667004; 44808001; 240298005; 67198005; 195042002; 233916004; 86014007; 20143001; 66657009; 413341007; 871686004; 129575004; 16797001; 17366009; 251094002; 251177002; 253528005; 195060002; 195069001; 425615007; 427665004; 49260003; 420002000; 276512006; 50799005; 43906007; 71792006; 41863008; 82838007; 422348008; 421869004; 88412007; 440028005; 440059007; 6180003; 75532003; 764732004; 10701000087104; 723866006; 789693005; 789039008; 442917000; 442559009; 450919004; 284941000119107; 284951000119109; 472809000; 698251009; 699256006; 698247007; 698249005; 49436004; 17869006; 28189009; 59118001; 9651007; 240299002; 80313002; 6285003; 63467002; 60423000; 27885002; 46319007; 55475008; 71908006; 63593006; 413342000; 3424008; 871832005; 10626002; 13395001; 13640000; 17338001; 204384007; 204383001; 251155001; 251167004; 251168009; 251174009; 251178007; 251183004; 26950008; 27337007; 300997008; 29894000; 195070000; 195080001; 425582007; 32425009; 32758004; 426749004; 33413000; 309809007; 276796006; 49710005; 276513001; 49982000; 44103008; 39357005; 81898007; 81681009; 4006006; 40593004; 36083008; 46220003; 62026008; 63232000; 6456007; 65778007; 66568003; 69730002; 698250005; 698272007; 706923002; 1142041008; 1142068008; 1142069000; 1142086006; 1142090008; 1142093005; 1142098001; 1142105006; 1142110005; 1142117008; 1142120000; 1142124009; 1142204001; 10164001; 11157007; 20852007; 251093008; 251120003; 251124007; 251161003; 251164006; 251092003; 251125008; 251165007; 251173003; 251180001; 251181002; 251186007; 251162005; 251170000; 251187003; 251172008; 251175005; 284470004; 195046004; 195105007; 195039008; 233910005; 233911009; 233915000; 233923003; 233922008; 233896004; 233897008; 233914001; 29320008; 49044005; 314208002; 54016002; 406461004; 61277005; 74390002; 429243003; 764457005; 723860000; 725145002; 442946007; 444605001; 443478002; 472810005; 473006003; 462170003; 462169004; 120041000119109; 21421000119109; 5761000119100; 698252002; 698270004; 698271000; 1010405004; 1142040009; 1142057008; 1142064005; 1142066007; 1142067003; 1142082008; 1142103004; 1142111009; 1142114001; 1142118003; 1142119006; 1142121001; 1142122008; 1142123003; 1156821008; 16415081000119104; 14718009; 2374000; 11849007; 19092004; 278482008; 184004; 251114004; 251123001; 251152003; 251163000; 251176006; 251188008; 251166008; 251171001; 251179004; 251182009; 74615001; 37760005; 76887001; 419752005; 82226007; 418341009; 771179007; 773587008; 719907006; 720507006; 721013001; 733125004; 733454004; 715971003; 715865008; 735682000; 735685003; 735684004; 195071001; 195072008; 195083004; 287057009; 300996004; 233902009; 233913007; 233918003; 233891009; 233900001; 233901002; 233892002; 233895000; 233899006; 233919006; 234172002; 233893007; 233894001; 233898003; 233903004; 233904005; 283645003; 282825002; 373905003; 47830009; 4554005; 39260000; 315027009; 5609005; 38274001; 38566003; 59272004; 72654001; 73459006; 74021003; 44602002; 77221000; 418493005; 419671004; 418818005; 419400008; 766883006; 770784003; 719823007; 720448006; 721010003; 715395008; 715560009; 715535009; 6624005; 735683005; 762534000; 762247006; 102451000119107; 710878005

4.32 eTable: Cardiovascular Disease

eTable 207: Phenotype and Sub Phenotype SNOMEDs

Phenotype	SNOMED
Cardiovascular Disease	22298006; 82523003; 410429000; 75543006; 21454007; 1386000; 432504007; 1755008; 84114007; 20059004; 274100004; 71908006; 71444005

© 2023 Vashisht R et al. *JAMA Network Open.*

4.33 eTable: Cataract

eTable 208: Phenotype and Sub Phenotype SNOMEDs

Phenotype	SNOMED
Cataract	5368009; 766834007; 8801005; 76309006; 8656007; 193570009; 34361001; 43959009; 79410001; 39450006; 34533008; 193589009; 193609000; 421920002; 264443002; 95722004; 445213003; 1412008; 53889007; 111515007; 11422002; 193600001; 193598007; 43972005; 78875003; 5318001; 65720003; 420756003

4.34 eTable: Cerebrovascular Diseases

© 2023 Vashisht R et al. *JAMA Network Open.*

eTable 209: Phenotype and Sub Phenotype SNOMEDs

Phenotype	SNOMED
Cerebrovascular Diseases	1153543002; 329501000119102; 1078223005; 1142056004; 1153545009; 1153611009; 1153612002; 1153631008; 1153638002; 1155688007; 1155689004; 1155697006; 1155699009; 1156017002; 1156027008; 1156029006; 11842891000119102; 11844791000119100; 16528221000119104; 346674811000119104; 38595071000119104; 111296006; 111297002; 14246007; 14977000; 230223008; 230716006; 230720005; 230225001; 230691006; 230714009; 230715005; 230724001; 230732009; 230482003; 230717002; 230721009; 230723007; 230735006; 15742000; 206576006; 276221006; 24624008; 276220007; 277316004; 277319006; 277322008; 18322005; 277324009; 186317009; 195373009; 18058007; 195212005; 195229008; 195233001; 195234007; 195206000; 195216008; 195232006; 195235008; 254775002; 286742002; 302881009; 234005004; 234142008; 371041009; 371040005; 371121002; 371158002; 45639009; 312586003; 67992007; 56384000; 90099008; 57981008; 37943007; 590005; 92997002; 93054001; 93396008; 384993003; 403775003; 78569004; 422504002; 86003009; 95459002; 771476007; 778060000; 15648361000119108; 15648401000119104; 15648561000119102; 718551002; 721979005; 721328009; 722643003; 722930000; 723082006; 713081000; 713265001; 15710641000119100; 284861000119104; 723857007; 724097003; 724425005; 724428007; 724429004; 724993002; 16709811000119106; 347011000119102; 788883008; 788882003; 788881005; 788880006; 788455001; 444657001; 444869007; 445349004; 15988391000119106; 16002111000119106; 16024031000119100; 16218291000119100; 292621000119100; 292631000119102; 292671000119104; 292691000119103; 329361000119107; 329421000119107; 329571000119107; 329651000119102; 734326000; 734383005; 734879002; 734880004; 734961002; 734965006; 735131004; 762630002; 762649003; 762651004; 734384004; 734963004; 762632005; 330791000119108; 734327009; 735132006; 449903008; 128171000119104; 140921000119102; 9901000119100; 99451000119105; 126011000119107; 699706000; 698363002; 9611000119107; 702374000; 702575003; 703166003; 703207000; 703226008; 705129007; 700467001; 266254007; 200332008; 55382008; 6594005; 65587001; 1055001; 287731003; 70936005; 56267009; 230690007; 230730001; 38742007; 80820004; 195183002; 195182007; 93468003; 71444005; 54519002; 64586002; 266253001; 25772007; 14070001; 10349009; 140251000119101; 15707881000119106; 15707921000119104; 16002191000119102; 16002231000119106; 21000119103; 27820001000004104; 291401000119102; 329391000119100; 429231000124101; 429811000124106; 429851000124107; 429861000124109; 430731000124103; 430831000124106; 430851000124104; 431421000124103; 432261000124102; 432481000124106; 433821000124102; 433971000124107; 434821000124104; 434831000124101; 434951000124104; 435271000124103; 436031000124102; 436041000124107; 436781000124104; 437911000124106; 454031000124106; 329541000119100; 16891111000119104; 496369931000119104; 1153544008; 1153546005; 1153607003; 1153608008; 1153630009; 1153632001; 1153633006; 1153634000; 1155698001; 1156016006; 1156018007; 1156019004; 11844831000119106; 152148641000119104; 16520041000119104; 204501003; 204493007; 110997000; 111298007; 111028009; 111299004; 10878002; 23808003; 16418006; 230221005; 230222003; 230220006; 230725000; 230713003; 230722002; 230738008; 230224002; 230731002; 15705007; 277328007; 277330009; 277299009; 277315000; 277320000; 276706004; 277325005; 277329004; 24654003; 195209007; 195211003; 195217004; 195236009; 195154000; 195160000; 195213000; 195200006; 302879007; 455791000124106; 455841000124109; 455851000124106; 5431000124100; 5451000124107; 5461000124109; 5471000124102; 5481000124104; 5501000124109; 5511000124107; 5531000124101; 5581000124100; 5591000124102; 5601000124105; 5651000124109; 429841000124105; 435281000124100; 436791000124101; 430841000124101; 838275008; 840422007; 840437006; 840438001; 840439009; 860820000; 870544005; 870566003; 870579007; 870637009; 871637001; 16279401000119108; 128218002; 23819000; 200260008; 281240008; 2495006; 26954004; 192771002; 192772009; 300920004; 192760003; 192765008; 192770001; 192761004; 195205001; 253699002; 307362003; 425932008; 35386004; 30400005; 48601002; 50751005; 428089008; 297157005; 54265003; 302880005; 234006003; 234149004; 233983001; 302878004; 302909007; 233964008; 233988005; 371160000; 29322000; 48248005; 275434003; 390936003; 302213007; 56453003; 413758000; 89980009; 408664007; 408665008; 92962004; 61091005; 93312006; 73173006; 73390009; 76402003; 423462008; 95458005; 95235009; 95644001; 457551000124104; 15648201000119100; 721411005; 721412003; 722004001; 722642008; 722929005; 16371781000119100; 713035000; 15708001000119106; 15708041000119108; 15710721000119104; 329671000119106; 720626009; 724424009; 724426006; 724779000; 724994008; 725132001; 16661931000119102; 16644681000119102; 788884002; 788454002; 445109004; 443929000; 444172003; 55734000; 373606000; 80758005; 80901002; 8166000; 8269002; 40276003; 88755007; 89142007; 36179005; 60706008; 65084004; 3681008; 65312002; 6729006; 95461006; 75138007; 88174006; 87937009; 88032003; 15648281000119108; 15648441000119102; 720809000; 724427002; 783161005; 783258000; 783415001; 783417009; 783418004; 783419007; 783422009; 783423004; 783629005; 783731008; 783733006; 336191000119105; 341801000119101; 16476641000119100; 16661971000119104; 16662331000119106; 16023911000119108; 716051003; 717003001; 285161000119105; 285171000119104; 285191000119103; 460312001; 460880006; 461326001; 284871000119105; 285201000119100; 472746006; 446712002; 15978431000119106; 16000391000119104; 16002031000119102; 16023991000119104; 291351000119109; 291371000119100; 291411000119104; 292661000119105; 292681000119101; 329371000119101; 329431000119105; 329451000119104; 329461000119102; 329491000119109; 329641000119104; 734396006; 734397002; 734959006; 734960001; 735114006; 735115007; 737159004; 737160009; 762629007; 762633000; 762648006; 762652006; 291481000119105; 329481000119106; 734374000; 734382000; 734964005; 329561000119101; 140911000119109; 9631000119102; 7931000119101; 703176000; 703180005; 703193000; 703205008; 703206009; 703208005; 703218000; 703219008; 703266007; 703267003; 703268008; 703311009; 703312002; 703313007; 703184001; 705066004; 703163006; 703221003; 710575003; 200333003; 42970005; 6956001; 20059004; 266257000; 64009001; 75543006; 69116000; 90520006; 28366008; 186893003; 73192008; 62914000; 34781003; 19474002; 51723007; 195180004; 6715005; 200331001; 12720001000004100; 16001671000119100; 16002071000119104; 16002391000119100; 16002471000119108; 16002511000119104; 291471000119107; 329401000119103; 329621000119105; 329631000119108; 330111000119103; 429241000124106; 429251000124108; 429821000124103; 429831000124100; 430721000124101; 433951000124102; 434191000124107; 434881000124100; 436021000124100; 436591000124109; 455801000124107; 455811000124105; 455821000124102; 455831000124104; 455901000124101; 455911000124103; 455921000124106; 455931000124109; 5571000124103; 5611000124108; 21290001000004104; 430781000124102; 434141000124103; 434891000124102; 430861000124102; 429221000124104; 433941000124104; 838308007; 838309004; 840419005; 840420004; 840434004; 840436002; 840441005; 213044006; 116288000; 128608001; 237867001; 128609009; 16061002; 21258007; 200259003; 262717000; 253194008; 25133001; 192769002; 192759008; 28790007; 192755002; 192764007; 195210002; 195155004; 262940009; 12853006; 262942001; 425420004; 32112006; 426814001; 427020007; 426651005; 276594006; 275363001; 4262001; 277196008; 297176007; 297138001; 43658003; 80606009; 416792008; 39925003; 40450001; 88922007; 61687004; 62702001; 64775002; 69798007; 70607008; 95455008; 95456009; 87555007; 232036006; 724357007; 787044009; 783413008; 783416000; 783420001; 783421002; 783630000; 783707003; 783716004; 783787000; 461431000124108; 106021000119105; 16026951000119102; 716745004; 284811000119102; 284821000119109; 284881000119108; 462035067002; 460890003; 460899002; 79341000119107

© 2023 Vashisht R et al. *JAMA Network Open.*

4.35 eTable: Chronic Kidney Disease

eTable 210: Phenotype and Sub Phenotype SNOMEDs

Phenotype	SNOMED
Chronic Kidney Disease	46177005; 90688005; 433144002; 431856006; 433146000; 431857002; 431855005; 120261000119101; 285061000119106; 285081000119102; 285911000119109; 285921000119102; 434431000124103; 449631000124102; 285041000119107; 285101000119109; 236435004; 57557005; 751000119104; 711000119100; 741000119101; 721000119107; 771000119108; 731000119105; 700378005; 700379002; 90731000119103; 90751000119109; 90761000119106; 90771000119100; 90791000119104; 90741000119107; 704667004; 90721000119101; 96441000119101; 284961000119106; 284971000119100; 284981000119102; 285011000119108; 709044004; 712487000; 285001000119105; 284991000119104; 368421000119108; 368431000119106; 368441000119102; 368451000119100; 368461000119103; 368471000119109; 236436003; 236433006; 236434000; 425369003; 776416004; 722098007; 722467000; 723190009; 722150000; 722149000; 714152005; 714153000; 111411000119103; 153851000119106; 285841000119104; 285871000119106; 285881000119109; 96731000119100; 96741000119109; 285831000119108; 285851000119102; 285861000119100; 286371000119107; 104931000119100; 129151000119102; 129161000119100; 129171000119106; 129181000119109; 117681000119102; 153891000119101; 8501000119104; 96701000119107; 96711000119105; 96721000119103; 96751000119106

4.36 eTable: Chronic Liver Diseases

eTable 211: Phenotype and Sub Phenotype SNOMEDs

Phenotype	SNOMED
Chronic Liver Diseases	235869004; 61977001; 420054005; 34736002; 79720007; 186639003; 838305005; 838377003; 838380002; 863957008; 870517000; 10295004; 197286002; 197291001; 197300007; 197303009; 197360009; 197305002; 197362001; 197359004; 197361008; 190823004; 427022004; 425413006; 266471006; 307757001; 266469006; 79607001; 44553005; 432908002; 536002; 5667009; 89580002; 58282009; 74669004; 74162007; 38662009; 78208005; 419728003; 774204006; 719454003; 721847002; 713181003; 713966008; 725938001; 782771007; 716203000; 717047007; 717187000; 715401008; 735451005; 700463002; 708248004; 702969000; 153091000119109; 1010616001; 1155841005; 109819003; 238033007; 12368000; 123716002; 31155007; 371139006; 29291001; 328383001; 88518009; 62484002; 66870002; 66937008; 76301009; 70737009; 235880004; 235915002; 235901004; 235909002; 235889003; 235898000; 235903001; 768006009; 768125005; 768126006; 768127002; 768288001; 768289009; 723360007; 723583009; 723829000; 725416005; 444707001; 347891000119103; 30188007; 1761006; 266468003; 72925005; 128302006; 41889008; 76783007; 435101000124104; 871619002; 197284004; 16070004; 197293003; 197294009; 197296006; 197299004; 197301006; 197310003; 19943007; 27156006; 30102006; 31712002; 33144001; 266470007; 50167007; 43634002; 43904005; 314963000; 57339008; 6075009; 60037002; 73146005; 424340000; 86454000; 773726000; 773737004; 771149000; 773415005; 774151000; 721710005; 783734000; 784346006; 715864007; 737202006; 735733008; 450880008; 831000119103; 703866000; 708198006; 103611000119102; 1092801000119102; 1155913007; 11179002; 1116000; 123604002; 123607009; 123605001; 123717006; 123606000; 15999000; 21861000; 238035000; 271440004; 371067004; 45256007; 41527003; 7265005; 89789003; 6183001; 63246000; 37666005; 37688005; 9843006; 235886005; 235895002; 235896001; 235897005; 240792005; 764962002; 767809001; 767810006; 724766009; 725939009; 725940006; 699189004

4.37 eTable: Chronic Obstructive Pulmonary Disease

eTable 212: Phenotype and Sub Phenotype SNOMEDs

Phenotype	SNOMED
Chronic Obstructive Pulmonary Disease	13645005; 195949008; 74417001; 57686001; 61937009; 77690003; 135836000; 233675009; 16003001; 195957006; 195953005; 196026004; 49691004; 60805002; 86680006; 442025000; 1010334009; 16846004; 31898008; 266355005; 47895001; 313296004; 313299006; 66987001; 70756004; 708030004; 63480004; 195951007; 185086009; 87433001; 33325001; 451981000124108; 233674008; 233677001; 23958009; 195958001; 195959009; 285381006; 4981000; 84409004; 785736001; 1751000119100; 1010333003; 836477007; 2912004; 425748003; 266356006; 313297008; 52571006; 89099002; 68328006; 735465007; 293241000119100

4.38 eTable: Coronary Heart Disease

© 2023 Vashisht R et al. *JAMA Network Open.*

eTable 213: Phenotype and Sub Phenotype SNOMEDs

Phenotype	SNOMED
Coronary Heart Disease	53741008; 414024009

4.39 eTable: Depression Bipolar Other Depressive Mood Disorders

eTable 214: Phenotype and Sub Phenotype SNOMEDs

Phenotype	SNOMED
Depression Bipolar Other Depressive Mood Disorders	15639000; 31446002; 77486005; 765176007; 46229002; 68019004; 191613003; 22121000; 5703000; 191630001; 430852001; 191639000; 191629006; 191623007; 35489007; 76441001; 57194009; 162004; 191616006; 49512000; 46244001; 66344007; 75837004; 111485001; 18818009; 79298009; 83225003; 78667006; 191621009; 36474008; 192362008; 13746004; 36583000; 191641004; 191638008; 191627008; 191618007; 76105009; 191620005; 63249007; 19527009; 33135002; 40379007; 36923009; 70747007; 782501005

4.40 eTable: Diarrhea

eTable 215: Phenotype and Sub Phenotype SNOMEDs

Phenotype	SNOMED
Diarrhea	62315008; 2919008; 267060006; 236077008; 249519007; 128333008; 409966000; 409587002

4.41 eTable: Fracture Of Bone

eTable 216: Phenotype and Sub Phenotype SNOMEDs

Phenotype	SNOMED
Fracture Of Bone	125605004

4.42 eTable: Glaucoma

eTable 217: Phenotype and Sub Phenotype SNOMEDs

Phenotype	SNOMED
Glucoma	33647009; 77075001; 30041005; 66747002; 46168003; 111514006; 29369005; 370504007; 84494001; 19144002; 204113001; 1003530008; 93435005; 4210003; 34623005; 392288006; 37155002; 53667005; 65460003; 23986001; 1207009; 68241007; 50485007; 392291006; 1003614009

4.43 eTable: Headache

eTable 218: Phenotype and Sub Phenotype SNOMEDs

Phenotype	SNOMED
Headache	162299003; 162311003; 162304002; 162307009; 279016001; 38823002; 712826000; 162211001; 267096005; 41413006; 162308004; 162310002; 162301005; 162309007; 44538002; 86925001; 712831003; 25064002; 330007; 4969004; 735938006

© 2023 Vashisht R et al. *JAMA Network Open.*

4.44 eTable: Heart Failure

eTable 219: Phenotype and Sub Phenotype SNOMEDs

Phenotype	SNOMED
Heart Failure	84114007

4.45 eTable: Hypertension

eTable 220: Phenotype and Sub Phenotype SNOMEDs

Phenotype	SNOMED
Hypertension	14973001; 123800009; 23130000; 206596003; 194783001; 194788005; 371125006; 48146000; 52698002; 56218007; 59720008; 73410007; 74451002; 39018007; 766937004; 712832005; 443482000; 132721000119104; 5501000119106; 706882009; 71421000119105; 71701000119105; 704667004; 1201005; 194785008; 38341003; 123799005; 78975002; 89242004; 31992008; 59621000; 28119000; 434711000124103; 871642009; 10725009; 19769006; 169465000; 194791005; 428575007; 429198000; 429457004; 57684003; 84094009; 397748008; 46481004; 65518004; 70272006; 427889009; 16229371000119106; 720568003; 461301000124109; 762463000; 697929007; 697930002; 127991000119101; 128001000119105; 140101000119109; 140111000119107; 140121000119100; 140131000119102

4.46 eTable: Hypoglycemia

eTable 221: Phenotype and Sub Phenotype SNOMEDs

Phenotype	SNOMED
Hypoglycemia	52767006; 237633009; 84371000119108; 230796005; 421725003; 719216001; 302866003; 237637005; 267384006; 190448007; 421437000; 120731000119103

4.47 eTable: Liver Cirrhosis

eTable 222: Phenotype and Sub Phenotype SNOMEDs

Phenotype	SNOMED
Liver Cirrhosis	871619002; 197291001; 197303009; 197296006; 197299004; 197310003; 27156006; 33144001; 425413006; 266469006; 6183001; 76301009; 235896001; 725938001; 699189004; 1761006; 420054005; 123604002; 123605001; 123606000; 15999000; 271440004; 45256007; 89580002; 78208005; 86454000; 725939009; 715864007; 715401008; 103611000119102; 1010616001; 16070004; 197293003; 197294009; 197305002; 197301006; 19943007; 31712002; 266470007; 266471006; 43904005; 37688005; 235895002; 235897005; 831000119103; 266468003; 109819003; 12368000; 123717006; 123716002; 21861000; 371139006; 536002; 74669004; 419728003; 725416005; 725940006; 716203000; 735733008

4.48 eTable: Myocardial Infarction

eTable 223: Phenotype and Sub Phenotype SNOMEDs

Phenotype	SNOMED
Myocardial Infarction	1755008; 233838001; 73795002; 65547006; 194802003; 76593002; 233826005; 233827001; 233825009; 233828006; 233830008; 233832000; 233835003; 233839009; 233841005; 233829003; 233834004; 233840006; 233837006; 233843008; 304914007; 282006; 311796008; 311793000; 79009004; 703165004; 703210007; 703212004; 703213009; 703360004; 703209002; 15712841000119100; 15712961000119108; 15713081000119108; 15713161000119100; 879955009; 836293000; 836295007; 846683001; 840312002; 840316004; 846668006; 868224003; 868225002; 194856005; 194809007; 194857001; 42531007; 22298006; 12238111000119106; 16837681000119104; 57054005; 54329005; 58612006; 70211005; 70422006; 896689003; 896691006; 896696001; 896697005; 15962541000119106; 380001000004106; 233836002; 233831007; 233842003; 233833005; 314207007; 311792005; 59063002; 401303003; 285981000119103; 23311000119105; 17531000119105; 703164000; 703211006; 703252002; 703253007; 703251009; 836294006; 840309000; 840609007; 840680009; 868214006; 868217004; 868220007; 868226001; 129574000; 15990001; 194858006; 32574007; 307140009; 418044006; 52035003; 394710008; 62695002; 401314000; 64627002; 428196007; 70998009

4.49 eTable: Neuropathy

eTable 224: Phenotype and Sub Phenotype SNOMEDs

Phenotype	SNOMED
Neuropathy	386033004; 230572002

4.50 eTable: Nausea

eTable 225: Phenotype and Sub Phenotype SNOMEDs

Phenotype	SNOMED
Nausea	73335002; 422587007; 698861005; 16932000; 2919008

4.51 eTable: Photosensitivity

eTable 226: Phenotype and Sub Phenotype SNOMEDs

Phenotype	SNOMED
Photosensitivity	201101007; 201015007; 41495000; 52636001; 79144000; 238522003; 238526000; 238528004; 238529007; 200999007; 200836002; 201024003; 254667001; 302836005; 304524009; 79372000; 54116000; 90128006; 90386003; 69231004; 58306008; 58419006; 402177006; 403197009; 403201009; 402480004; 402173005; 402175003; 403198004; 403210001; 403199007; 403205000; 403206004; 403208003; 403626007; 418686001; 200837006; 95342006; 773769008; 720820000; 721007005; 724551009; 724873006; 789051005; 449732002; 449733007; 111200005; 238712007; 238518008; 109251008; 109252001; 21543000; 238525001; 238530002; 51048002; 43982006; 72100002; 84036008; 89019003; 46795000; 402176002; 6618004; 402165001; 402166000; 402174004; 402179009; 402318000; 403200005; 403202002; 403203007; 403204001; 403207008; 403365004; 733209003; 733210008; 737249005; 737250005; 737251009; 762664003

4.52 eTable: Renal Failure

eTable 227: Phenotype and Sub Phenotype SNOMEDs

Phenotype	SNOMED
Renal Failure	434431000124103; 109477002; 236436003; 236424009; 236428007; 236431008; 236432001; 23697004; 236433006; 236434000; 31005002; 269257004; 269301005; 373422007; 430535006; 45646000; 301814009; 438783006; 57557005; 78209002; 422593004; 213231008; 423533009; 776416004; 722095005; 722096006; 722278006; 723188008; 723189000; 723190009; 722721004; 733097003; 724093004; 285841000119104; 286371000119107; 153891000119101; 698591006; 90771000119100; 90791000119104; 129561000119108; 704667004; 712487000; 42399005; 46177005; 51292008; 111407006; 14669001; 429224003; 90688005; 298015003; 194781004; 194780003; 49220004; 368471000119109; 870589006; 236423003; 236429004; 236435004; 307309005; 268854008; 275408006; 424114000; 429489008; 363287001; 43258006; 373421000; 88380005; 36225005; 62216007; 36568005; 425369003; 368951000119105; 713453003; 713696000; 111411000119103; 789660001; 444976001; 445236007; 127991000119101; 128001000119105; 129721000119106; 140031000119103; 145681000119101

© 2023 Vashisht R et al. *JAMA Network Open.*

4.53 eTable: Stroke

eTable 228: Phenotype and Sub Phenotype SNOMEDs

Phenotype	SNOMED
Stroke	75543006; 21454007; 71444005; 270907008; 20059004; 274100004; 1386000; 432504007; 291571000119106

4.54 eTable: Thrombocytopenia

eTable 229: Phenotype and Sub Phenotype SNOMEDs

Phenotype	SNOMED
Thrombocytopenia	13172003; 302215000; 73397007; 75331009; 267535004; 74576004; 36070007; 267534000; 1156746003; 1156838007; 19307009; 111588002; 302873008; 234482009; 234485006; 234487003; 234483004; 234484005; 234486007; 323079008; 371074009; 373420004; 439007008; 441134009; 60628003; 63444004; 37492005; 402654005; 87902006; 717769007; 720521008; 721304007; 721882001; 722475006; 719021005; 789660001; 737221003; 699208000; 711407000; 866152006; 128094006; 128091003; 191322006; 191323001; 2897005; 128092005; 128093000; 33183004; 34395002; 30182008; 48788004; 49886003; 54569005; 417626001; 416902009; 82190001; 421766003; 359531004; 438476003; 359536009; 438492008; 73162004; 441322009; 402653004; 403837005; 78345002; 78129009; 85589009; 86635005; 771075004; 712922002; 713388002; 97571000119109; 724637001; 733096007; 783194008; 783251006; 716336002; 154826009

4.55 eTable: Urinary Tract Infectious

eTable 230: Phenotype and Sub Phenotype SNOMEDs

Phenotype	SNOMED
Urinary Tract Infectious	267204006; 68566005; 275742001; 236629009; 236624004; 87696004; 720406004; 67277002; 48278001; 8725005; 84619001; 15628003; 197845000; 199206009; 4800001; 67224007; 12301009; 236626002; 236379002; 236625003; 61373006; 9713002; 38822007; 80375002; 11251000; 17322007; 56717001; 59530001; 17121006

4.56 eTable: Valvular Heart Disease

eTable 231: Phenotype and Sub Phenotype SNOMEDs

Phenotype	SNOMED
Valvular Heart Disease	274097009; 368009

4.57 eTable: Vomiting

eTable 232: Phenotype and Sub Phenotype SNOMEDs

Phenotype	SNOMED
Vomiting	16932000; 73335002; 765480005; 146291000119108; 2919008; 698861005; 23971007; 424580008; 444673007; 422400008; 332982000

© 2023 Vashisht R et al. *JAMA Network Open.*

eAppendix 5. Discussion and Limitations

We took a statistically rigorous approach to minimize the impact on significant findings. Firstly, we conducted our study independently at each of the UC Health sites. The reason for this was to ensure that we factor in the medical practices specific to each UC, as these practices are known to vary across different geographic areas and policies. Secondly, we utilized a wealth of pre-baseline clinical patient history to address observable confounding factors as thoroughly as possible, aiming to match patients fairly for meaningful comparisons. Thirdly, we collected evidence from each UC and evaluated the heterogeneity of the evidence using a random-effects meta-analysis approach. We only considered evidence that was likely to be consistent and stable. Fourthly, to assess the stability and robustness of the evidence, we conducted a leave-one-medical-center-out influence analysis. We retained evidence that appeared stable across the UC Health system despite variations in medical practices and analytical considerations. Finally, we accounted for multiple hypothesis testing by applying the false discovery rate approach. We compare the evidence gathered from the clinical data presented in this study to that with the evidence and knowledgebase from the randomized controlled trials. A brief table comparing evidence from clinical data and evidence from randomized controlled trials or their meta-analysis is provided in the table below.

eTable 233: Evidence from clinical data and from RCTs or network-meta analysis of RCTs

Evidence.from.clinical.data	Evidence.from.RCTs.or.Their.Meta.Analysis..PubMed.Id.
GLP1RA is effective in glycemic controls compared with DPP4i	PMID:24499291; PMID:19515413; PMID:23627775
High risk of metabolic failure among those treated with SGLT2i versus GLP1ra, albeit unstable estimate	PMID:27350752 and reference therein suggest both SGLT2i and GLP1ra are likely to be equally effective in glycemic control.
Treatment with SGLT2i or DPP4i had lower risk of adverse cardiovascular outcomes	PMID:35296336 network meta-analysis of 23 CVOTs
GLP1RA and SGLT2i have potential for cardiovascular (GLP1RA) and cardiac (SGLT2i) benefit	PMID:35296336 network meta-analysis of 23 CVOTs
DPP4i compared with SU could have cardiovascular benefits in addition to superior glycemic control and less liklehood of hypoglycemia	PMID: 31536101 CAROLINA trial. Favorable attributes of DPP4i such as lower risk of hypoglycemia, weight gain make it potentially suitable choice.
Adding GLP1RA or SGLT2i to metformin also showed lower risks of new chronic kidney disease and renal failure, compared to SU	PMID:28131656; PMID: 31497854; PMID: 34180939; PMID: 30424892; PMID: 33441402
Better effectiveness and safety of GLP1RA and SGLT2i compared with SU	PMID: 36129997; PMID: 36129996 - GRADE trial, our findings indicated better effectiveness of GLP1RA compared with SU when added to metformin monotherapy in maintaining glycemic control in addition to their benefits against cardio-, renal-, liver-disorders as well as hypertension. GRADE trial, our findings indicated better effectiveness of GLP1RA compared with SU when added to metformin monotherapy in maintaining glycemic control in addition to their benefits against cardio-, renal-, liver-disorders as well as hypertension

We acknowledge that, despite our diligent endeavors, the chance of bias arising from unobserved confounding remains. For example, we did not account for treatment costs in our analysis, which could potentially muddle treatment choices and persist as an unobserved variable in our study. We binarized (i.e. present or absent) medical procedures, diagnoses, medications, and laboratory measurements to use as a surrogate marker of the clinical state of a patient to adjust for potentially observed confounders. As an example, we abstained from incorporating the precise numerical values of platelet counts. Instead, we opted for a binary representation denoting the presence or absence of the test ordered to assess platelet count. This approach has the potential to obscure the underlying rationale for treatment decisions established at the baseline. Likewise, we did not take into account the patients' race, ethnicity, or socioeconomic status, all of which could impact treatment determinations and results. We also concede the possibility of lingering confounding in the evidence concerning the efficacy and safety of T2D treatments. Furthermore, our analysis is restricted to second-line treatments. We censor patient time in the event that a patient requires a third medication. For instance, if a patient was prescribed an SGLT2 inhibitor after their DPP4 inhibitor treatment, in addition

to metformin, we would stop recording data at the time of the SGLT2 inhibitor prescription. This could potentially make it harder to interpret the results when considering a third treatment. Additionally, there is a possibility of survivorship bias. It is challenging to specifically know why some patients who stopped a given medication did so, and so the bias towards the patients who took a given medicine for longer periods (and the side effect associated with such long-term use) could have been accentuated in our study.

We required each patient to have at-least 90 days of continuous enrollment within the UC system. Currently, there exists no established empirical framework outlining the ideal duration for such a monitoring window. Our decision to opt for a 90-day interval was primarily driven by the intention to encompass an adequate observation period for patients within the UC system. This duration is long enough to account for potential prescription refills and ensures a comprehensive assessment. Indeed, the matching process was designed to accommodate the imperative 90-day prerequisite. Consequently, only those patients who possessed a minimum of 90 days' worth of observational data preceding the index date were eligible for inclusion in the matching procedure. In the future, we could monitor the specific EHRs for even longer to get an even more accurate understanding of the length of pre-index observation window. But in truth, even extending the monitoring period for years will not preclude the possibility that someone could receive a medication from an unaccounted source. Still, even in our study the only way that a patient could receive a medication from an external provider prior to an index treatment made from within a UC Health site is if that patient receives at least one diabetes medication from one doctor and at least another diabetes medication from an entirely different doctor in a completely different health system. Although this type of disorganized care is the bane of healthcare in the US, it is thankfully such fractured diabetes medication management is very rare. Usually, one doctor prescribes and makes treatment decisions for all a patient's diabetes medications. As such, the possibility of this confounding issue is likely quite low.

© 2023 Vashisht R et al. *JAMA Network Open.*
