# Supplementary material for: Second-Line Pharmaceutical Treatments for Patients with Type 2 Diabetes
Source: JAMA Netw Open. 2023 Oct 2;6(10):e2336613. doi: 10.1001/jamanetworkopen.2023.36613 (PMC10546239; doi:10.1001/jamanetworkopen.2023.36613)
Supplement: Supplement 2. — Data Sharing Statement [file jamanetwopen-e2336613-s002.pdf]

Data Sharing Statement

Vashisht. Second-Line Pharmaceutical Treatments for Patients with Type 2 Diabetes. *JAMA Netw Open*. Published October 02, 2023. doi:10.1001/jamanetworkopen.2023.36613

Data
Data available: No

Additional Information
Explanation for why data not available: The data used for this analysis was derived from the de-identified electronic health records of patients receiving care across the University of California Health. Although de-identified, the individual-level nature of the data used risks individuals being identified, or being able to self-identify, if the data are released publicly.
